# miR-9 utilizes precursor pathways in adaptation to alcohol in mouse striatal neurons

**DOI:** 10.3389/adar.2023.11323

**Published:** 2023-06-06

**Authors:** Edward Andrew Mead, Yongping Wang, Sunali Patel, Austin P. Thekkumthala, Rebecca Kepich, Elizabeth Benn-Hirsch, Victoria Lee, Azra Basaly, Susan Bergeson, Hava T. Siegelmann, Andrzej Zbigniew Pietrzykowski

**Affiliations:** ^1^ Laboratory of Adaptation, Reward and Addiction, Department of Animal Sciences, Rutgers, The State University of New Jersey, New Brunswick, NJ, United States; ^2^ Thermo Fisher Scientific Inc., Austin, TX, United States; ^3^ Department of Cell Biology and Biochemistry, School of Medicine, Texas Tech University Health Sciences Center, Lubbock, TX, United States; ^4^ Department of Machine Learning, Mohamed bin Zayed University of Artificial Intelligence, Abu Dhabi, United Arab Emirates; ^5^ Biologically Inspired Neural & Dynamical Systems Laboratory, The Manning College of Information and Computer Sciences, University of Massachusetts, Amherst, MA, United States

**Keywords:** addiction, digital PCR, alcohol adaptation, microRNA miR-9, medium spiny neurons

## Abstract

microRNA-9 (miR-9) is one of the most abundant microRNAs in the mammalian brain, essential for its development and normal function. In neurons, it regulates the expression of several key molecules, ranging from ion channels to enzymes, to transcription factors broadly affecting the expression of many genes. The neuronal effects of alcohol, one of the most abused drugs in the world, seem to be at least partially dependent on regulating the expression of miR-9. We previously observed that molecular mechanisms of the development of alcohol tolerance are miR-9 dependent. Since a critical feature of alcohol action is temporal exposure to the drug, we decided to better understand the time dependence of alcohol regulation of miR-9 biogenesis and expression. We measured the effect of intoxicating concentration of alcohol (20 mM ethanol) on the expression of all major elements of miR-9 biogenesis: three pri-precursors (pri-mir-9-1, pri-mir-9-2, pri-mir-9-3), three pre-precursors (pre-mir-9-1, pre-mir-9-2, pre-mir-9-3), and two mature microRNAs: miR-9-5p and miR-9-3p, using digital PCR and RT-qPCR, and murine primary medium spiny neurons (MSN) cultures. We subjected the neurons to alcohol based on an exposure/withdrawal matrix of different exposure times (from 15 min to 24 h) followed by different withdrawal times (from 0 h to 24 h). We observed that a short exposure increased mature miR-9-5p expression, which was followed by a gradual decrease and subsequent increase of the expression, returning to pre-exposure levels within 24 h. Temporal changes of miR-9-3p expression were complementing miR-9-5p changes. Interestingly, an extended, continuous presence of the drug caused a similar pattern. These results suggest the presence of the adaptive mechanisms of miR-9 expression in the presence and absence of alcohol. Measurement of miR-9 pre- and pri-precursors showed further that the primary effect of alcohol on miR-9 is through the mir-9-2 precursor pathway with a smaller contribution of mir-9-1 and mir-9-3 precursors. Our results provide new insight into the adaptive mechanisms of neurons to alcohol exposure. It would be of interest to determine next which microRNA-based mechanisms are involved in a transition from the acute, intoxicating effects of alcohol to the chronic, addictive effects of the drug.

## Introduction

Alcohol Use Disorder (AUD) is a chronic, incurable disease affecting people worldwide regardless of their social or economic status. AUD leads to an estimated 132.6 million disability-adjusted life years (DALYs), and an estimated 3 million deaths per year [1]. In the United States AUD is one of the largest drug problems, and alcohol abuse costs the country hundreds of billions of dollars each year in lost revenue, treatments, and mortality [2, 3]. Development of alcohol addiction takes place over time through the complex actions of alcohol on the brain’s reward system. Temporal characteristics of alcohol actions are critical yet poorly understood.

In recent years, many studies have focused on the epigenetic underpinnings of addiction to better understand the development of AUD [4]. MicroRNAs (miRNAs), small (∼21 nt long) endogenous RNA molecules are powerful epigenetic modulators regulating gene expression on a genome-wide scale [5]. It has been estimated that microRNAs modify the expression of approximately 60% of the transcripts in humans [6] and play a fundamental role in the development and maintenance of neurons in the brain [7]. microRNAs are also key elements of the development of drug [8–10] and alcohol addiction [11–13].

One particular microRNA involved in brain development [14], function [15], and malfunction [16] is miR-9 (specifically miR-9-5p). Dysregulation of miR-9-5p by alcohol has a broad impact on the brain, and several downstream targets of miR-9-5p have been well-established ([17], reviewed in depth in [18]). However, effects of upstream changes in miR-9 biogenesis on mature miR-9 are less studied*.* Understanding alcohol regulation of miR-9 biogenesis could help to uncover new mechanisms of alcohol action, and ultimately may lead to discovery of novel therapeutic options in addiction.

**FIGURE 1:**
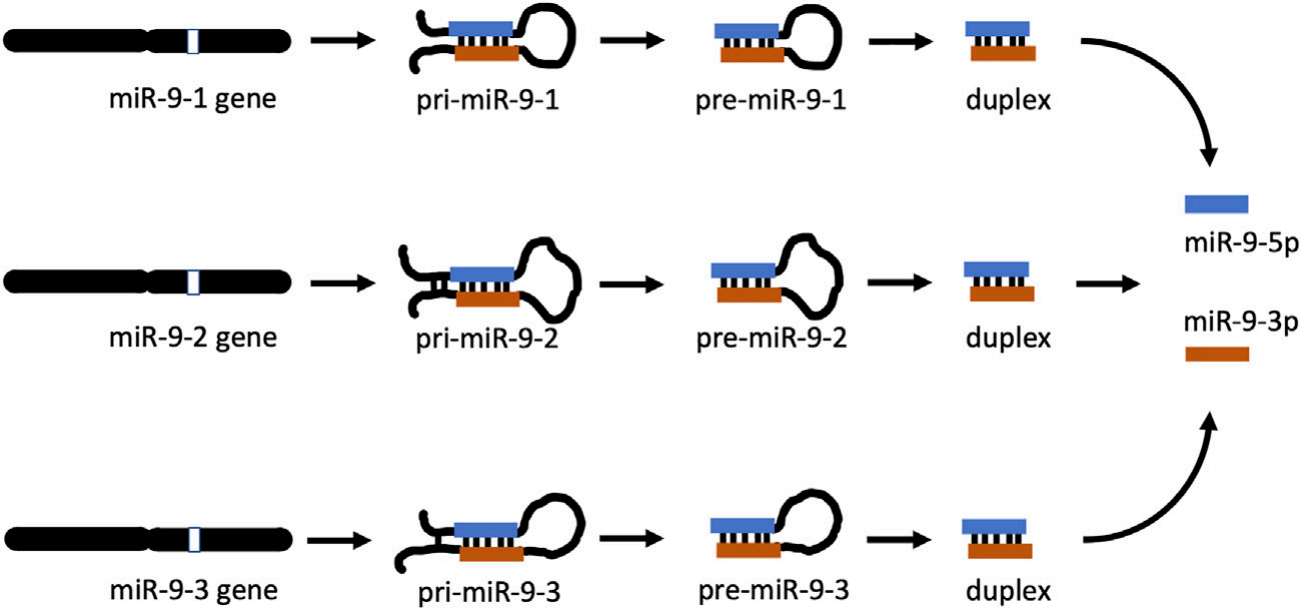
miR-9 biogenesis pathways. In apes (including humans) and rodents (including mice), there are 3 mir-9 genes (mir-9-1, mir-9-2, and mir-9-3) located on different chromosomes. Each gene produces its own primary RNA precursor (pri-mir-9-1, pri-mir-9-2, and pri-mir-9-3), which is cleaved to a pre-precursor (pre-mir-9-1, pre-mir-9-2, and pre-mir-9-3). Each precursor is further processed to yield a duplex containing both miR-9-5p and miR-9-3p. Separation of each duplex into single-stranded RNA sequences generates two final forms of miR-9, which are physiologically active: miR-9-5p and miR-9-3p. All miR-9-5p produced via 3 separate biogenesis pathways are identical. Similarly, all final miR-9-3p are indistinguishable.

miR-9 is an ancient microRNA found from invertebrates to mammals [19, 20] and has a complex biogenesis. In many species there are three distinct miR-9 genes located on three different chromosomes. In humans, miR-9 genes are located on chromosomes 1, 5, and 15, while their equivalents in mice are on chromosomes 3, 7, and 13, respectively [21]. In both species, each gene gives rise to a separate, long, primary precursor, pri-mir-9-1, pri-mir-9-2, and pri-mir-9-3 (Figure 1). Each pri-precursor is subsequently trimmed to a shorter pre-precursor of a characteristic hairpin loop structure (Figure 1). The next step produces an even shorter, small, double-stranded duplex consisting mostly of two complementarily bound miRNA strands. Ultimate processing of the duplex separates the strands yielding two short, single-stranded, distinct mature microRNAs: miR-9-5p and miR-9-3p. Importantly, in the case of miR-9, all final mature miR-9-5p products of the 3 biogenesis pathways are identical [22]. Similarly, all mature miR-9-3p end products are indistinguishable (Figure 1). Both mature miR-9 strands execute biological action by interacting through complementarity with multiple targets (RNA transcripts), which usually leads to suppression of expression of the targets.

miR-9-3p has been shown to be biologically active and play an important role in carcinogenesis [23] such as in Burkitt’s lymphoma [24] and breast cancer [23, 25] as well as brain pathologies. Decreased expression of miR-9-3p has been linked to neurological disorders including Alzheimer’s and Huntington’s diseases [26].

Thus, understanding temporal regulation of the expression of various miR-9 precursors as well as both forms of mature miR-9 by alcohol is critical in enhancing our understanding of the mechanisms involved in the development of alcohol addiction and adaptation to alcohol exposure.

## Materials and methods

### Striatal culture

C57BL6/J mice (Jackson Laboratory, Bar Harbor, ME) were maintained under 12h:12h light:dark cycles at standard temperature and humidity with food and water provided *ad libitum* at the Bartlett Animal Facility (Rutgers-New Brunswick). Mice were monitored daily, and cages were routinely changed. Mice were bred for litters to use in generating cultures. All animal experiments were approved by the Rutgers Institutional Animal Care and Use Committee (IACUC Protocol # 10-024).

Cultures of Medium Spiny Neurons (MSN) at ∼95% purity [27, 28], were prepared following well-established protocols [29–31]. At day 5 after birth (P5), pups were decapitated, and brains were immediately removed and placed into a 60 mm plate containing ice-cold CMF-HBSS (100 mL of final solution made with 10 mL 10x HBSS (Life Technologies), 0.7 mL 5% NaHCO_3_ (Sigma), final pH 7.1, brought to final volume with ultrapure water, then filter sterilized and stored at room temperature). The Nucleus Accumbens (NAc) was removed using a mouse brain atlas for visual reference [32]. Equal numbers of male and female pups were used for each preparation to limit the bias of using a single gender. Striatal tissue was diced into smaller fragments, ∼1 mm in diameter in 3.15 mL cold CMF-HBSS, and trypsinized with the addition of 0.35 mL of 2.5% trypsin at 37°C. After 10 min, 8 mL DMEM-FBS medium (178 mL DMEM (high glucose, no sodium pyruvate, no glutamine (Irvine Scientist), with 20 mL FBS, 0.5 mL 10,000 u Pen/Strep and 2 mL 200 mM glutamine (Life Technologies), stored at 4°C in the dark) was added, and the solution was centrifuged at 300 x g for 5 min to pellet the cells. The medium was aspirated from the tube, and 5 mL of Growth Medium (100 mL DMEM/F12 (with Glutamax; Life Technologies), with 2 mL FBS (1.9% v/v), 2 mL B-27 (1.9% v/v; Life Technologies), and 1 mL penicillin/streptomycin (0.95% v/v), stored at 4°C in the dark) was added. Trituration to further break apart aggregates was conducted using a fire-polished Pasteur pipet, and the tube was spun again as above. Excess media was removed, and the cells were resuspended in 10 mL of Growth Medium. Preparations were conducted under a sterile hood to help maintain sterility, except for centrifugation. The concentration of live cells/mL was estimated by hemocytometer counts of live:dead cells using trypan blue, and the cell stock solution was diluted to a final concentration of 0.5 × 10^6^ cells/mL. Plates of striatal cells were prepared by seeding 2 mL of the cell stock solution onto 35 mm cell culture plates that had been coated with ornithine (Sigma-Aldrich, St. Louis, MO) and laminin (Life Technologies, Carlsbad, CA) for cell adherence and enrichment for neurons. Preparations were rapidly conducted as speed was critical for cell viability. Twenty-four hours post-seeding, after allowing cells to adhere, the media was replaced with a 2 mL Neurobasal Medium (NB)/plate (100 mL Neurobasal A Medium supplemented to 2.0 mM glutamine final concentration; Life Technologies), with 2 mL FBS (1.9% v/v), 2 mL B-27 (1.9% v/v; Life Technologies), and 1 mL penicillin/streptomycin (0.95% v/v) stored at 4°C in the dark). Cultures were maintained at 37°C/5% CO2 in a cell culture incubator with saturated humidity for another week before starting exposures, and as a result, the neurons were nearly 2 weeks old since birth (5 days *in vivo* + 8 days *in vitro* = 13 days total) at the start of the experiments. Sometimes in microRNA studies alpha-amanitin is added to cultured cells to inhibit RNA polymerases II and III, which process microRNA. Since alpha-amanitin can also cause widespread transcriptional stress and apoptosis [33, 34] we did not add it to our cultures.

### Ethanol exposure

We chose 20 mM ethanol for alcohol exposures as it represents a physiologically relevant dose of alcohol while maintaining cell viability. 20 mM ethanol corresponds to a 0.092% Blood Alcohol Content (BAC), which can be achieved in humans by a quick (30–60 min) consumption of 3–4 standard drinks of alcohol by a 150-pound individual [35] causing disinhibition, impaired thinking, and potential DWI/DUI in the US [35]. Previously we have shown that 20 mM ethanol can upregulate the expression of miR-9-5p within 15 min after exposure of the rat brain organotypic cultures containing supraoptic nucleus (SON) neurons leading to alcohol tolerance [17]. Importantly, 20 mM causes minimal neuronal cell death in culture as shown by us [12] and others [36].

Seven days after seeding neurons the cells were subjected to the alcohol exposure and withdrawal with the following collection time points: Control = 0 min exposure +0 h withdrawal, 15 min 20 mM ethanol exposure +0 h withdrawal, 15 min 20 mM ethanol exposure +1 h withdrawal, 15 min 20 mM ethanol exposure +6 h withdrawal, 15 min 20 mM ethanol exposure +12 h withdrawal, 15 min 20 mM ethanol exposure +24 h withdrawal, 6 h 20 mM ethanol exposure +0 h withdrawal, 6 h 20 mM ethanol exposure +6 h withdrawal, 6 h 20 mM ethanol exposure +24 h withdrawal. Collection at each time point was conducted in triplicate. For each control, 5–7 plates were prepared. Cells were treated by aspirating off media and replacing with either a neurobasal medium (“media only” control) or a neurobasal medium with alcohol (NBE with 20 mM final ethanol concentration). Ethanol evaporation was minimized by maintaining NBE plates in a semi-sealed container in the incubator with saturated humidity and additional plates of medium containing the same concentration of ethanol, based upon the methods of Pietrzykowski [12, 17].

After a defined length of exposure, NB or NBE media were removed. For plates without a withdrawal period, cells were collected immediately. For cells with a withdrawal period, the NB medium replaced the NBE medium for a defined length of time after which cells were collected.

Cell collection was carried out by quickly rinsing plates with 2 mL ice-cold PBS followed by scraping cells from the plate with a cell scraper in 200 uL PBS. Cells were immediately flash-frozen in liquid nitrogen and stored at −80°C until processed for total RNA isolation as described previously [16].

### Alcohol concentration verification

Media samples were gathered at each collection point in the experimental process (before and after ethanol addition, during exposure and withdrawal) to verify alcohol concentration. Alcohol measurements were conducted using an AMI Analyzer according to the manufacturer’s instructions (Analox Instruments Ltd., Lunenburg, MA). 10, 20, and 50 mM ethanol standards in media were used to calibrate the instrument prior to reads to ensure accuracy. Alcohol measurements confirmed that ethanol loss was minimized using our methodology as previously described in more detail [12, 17].

### RNA isolation

Total RNA or Small RNA (for precursor assays) was isolated by miRVana kit according to the manufacturer’s instructions (Life Technologies). Concentration and purity were analyzed by a Nanodrop 1000 Spectrophotometer (Thermo Fisher Scientific Inc., Wilmington, DE), and aliquots of each sample were used to prepare 10ng/ul dilutions in nuclease-free water for RT-qPCR. All samples were kept at −80°C.

### RT-qPCR

#### miRNA

We conducted RT and qPCR steps to assess mature miR-9-5p and miR-9-3p based upon the manufacturer’s protocols for TaqMan Small RNA Assays (Applied Biosystems, Inc., Foster City, CA). Using the TaqMan MicroRNA Reverse Transcription kit (Applied Biosystems, Inc., Foster City, CA), mature miRNA was converted into cDNA using a Veriti Thermal Cycler (Applied Biosystems, Inc., Foster City, CA). A working stock of 10 ng/ul of total RNA was prepared and used for RT with each experimental sample in a total volume of 15 μL. RT consisted of 16°C 30 min, 42°C 30 min, 85°C 5 min, and hold at 4°C. The two-step process of RT followed by qPCR permitted finer control/greater accuracy for the final RT-qPCR reaction by allowing us to equalize the quantities of cDNA. cDNAs were amplified with the Taqman Small RNA Assay kit (Applied Biosystems, Inc., Foster City, CA) using an ABI Step One Plus Thermocycler (Applied Biosystems, Inc., Foster City, CA). 1.33 μL of RT sample was used for Taqman qPCR in a total volume of 20 μL. TaqMan reactions were carried out using Universal Master Mix II, no UNG from Applied Biosystems, and 1 µL of TaqMan MicroRNA assay primers. Triplicates of each sample were used in the 96-well plate (except for controls, where *n* = 5, or *n* = 7) to ensure greater accuracy. The average was taken as the value for each. For normalization and quality assessment we followed absolute quantification methods which can provide better accuracy without the need of a separate housekeeping gene, as described by Iguchi [37], Arabkari [38], and Wang [39]. We used 7-log dilution range (10 fmol–10^−4^ fmol) of synthetic miR-9-5p and miR-9-3p oligos (amplification efficiency, *R* ^2^ = 0.9993). The cycling protocol consisted of 95°C for 10 min, followed by 40 cycles of (95°C for 15 s and 60°C for 1 min), in an ABI Step One Plus Thermocycler (Applied Biosystems, Inc., Foster City, CA). Data collection occurred at the 60°C step.

#### Pre- and Pri-miRNA precursors

We used the Ambion miRVana kit following the manufacturer’s instructions, to separate small RNA molecules including pre-precursor miRNAs (∼100 nt in length) from the much larger (over 1,000 nt in length) pri-precursors for subsequent studies.

##### Pre- precursors

RT was conducted using a miScript II RT kit (Qiagen). miR-9 precursors (pre-mir-9-1, pre-mir-9-2, pre-mir-9-3) were pre-amplified using stock primers for RT-qPCR from Qiagen. After this, a 1:20 dilution of the pre-amp product was used for normal qPCR. Standard curves were prepared from 100 fmol using stocks of 9-1, 9-2, and 9-3 oligos. Pre-amplification was carried out using Qiagen miScript Precursor assay kits for pre-mir-9-1, -9-2, and -9-3 respectively, along with a miScript PreAMP PCR Kit (Qiagen).

##### Pri-precursors

Cell cultures were obtained as indicated above. RT was carried out with SuperScript VILO Master Mix (Invitrogen) using the manufacturer’s recommendations. cDNA samples were sent to Life Technologies for subsequent digital PCR.

##### Digital PCR

30 ng/μL of each alcohol exposure sample was tested with three TaqMan Pri-miRNA assays (Mm04227702 pri-mmu-mir-9-1, Mm03306269 pri-mmu-mir-9-2, and Mm03307250 pri-mmu-mir-9-3) (Thermo Fisher Scientific Inc.). 1μL of each sample was added to 10 μL QuantStudio 3D Digital PCR Master Mix, 1 μL of TaqMan Assay (20X), and 8 μL of nuclease-free water for 20 μL of the reaction mix. 14.5 μL of reaction mix was loaded on each QuantStudio 3D Digital PCR 20K Chip (Thermo Fisher Scientific Inc.) using QuantStudio 3D Digital PCR Chip Loader (Thermo Fisher Scientific Inc.) according to manufacturer’s instruction. The digital PCR was performed on Proflex 2x Flat PCR System (Thermo Fisher Scientific Inc.) with thermal cycling of 10 min at 96°C, followed by 39 cycles at 60°C for 2 min and 98°C for 30 s, followed by holding at 60°C for 2 min and 10°C for long term. Each chip fluorescence intensity was read using QuantStudio 3D Digital PCR instrument (Thermo Fisher Scientific Inc.) and analyzed copies/μL based on Poisson distribution using QuantStudio 3D Analysis Suite Cloud Software (Thermo Fisher Scientific Inc.).

### Statistical analysis

Expression data for statistical analysis were obtained using oligos in a standard curve method for mature miR-9-5p and miR-9-3p, 2^−ΔΔCT^ method for pre-precursors, and Poisson distribution for pri-precursors. The data were analyzed using unpaired, two-tailed t-tests. Data were expressed as fold-change to visualize the relationship between exposure condition and molecule expression. *p*-value below 0.05 (*p* < 0.05) was set as statistically significant.

## Results

### Regulation of miR-9-5p and miR-9-3p expression by short exposure to alcohol

**FIGURE 2:**
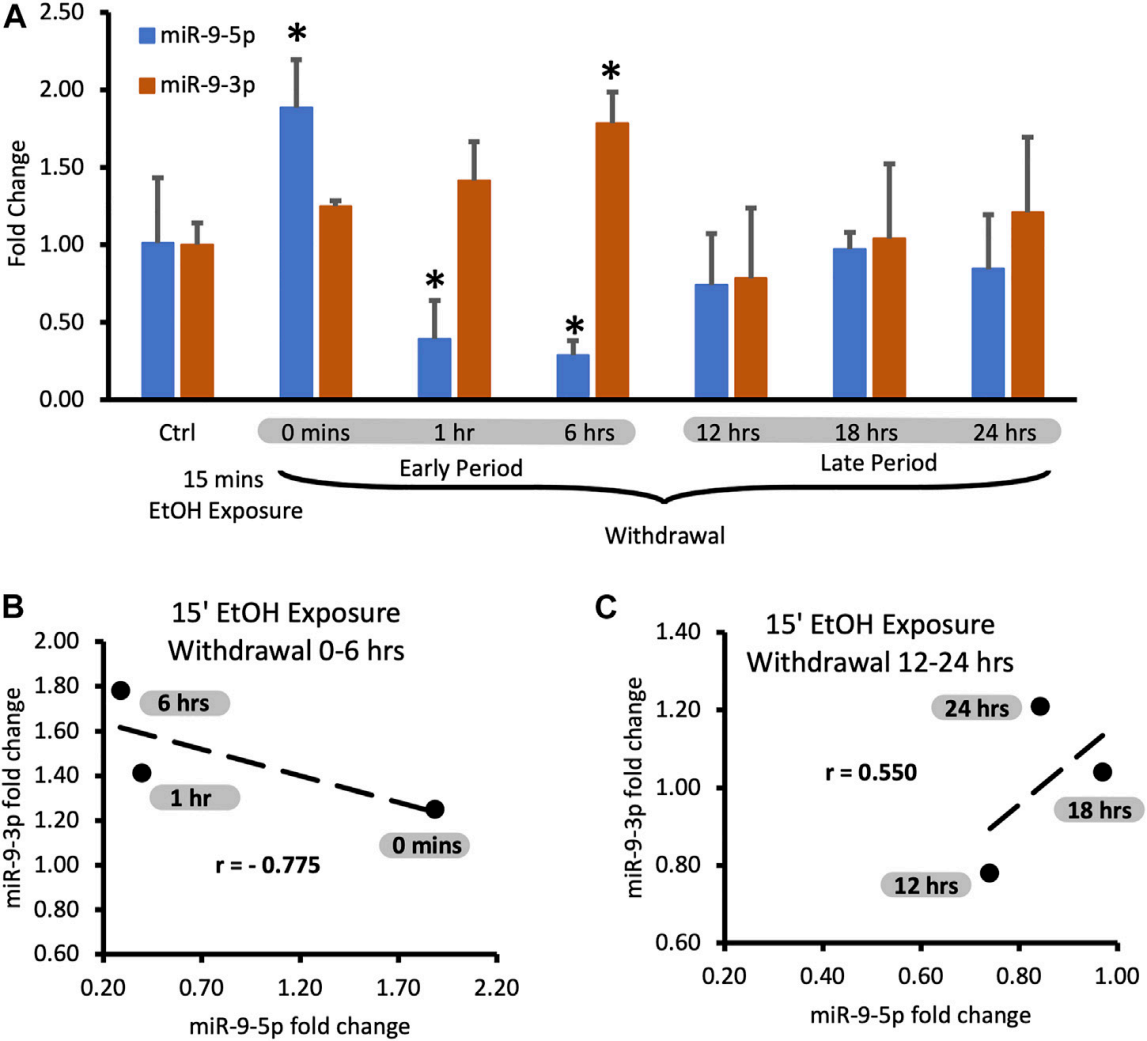
Expression levels of miR-9-5p and -3p during 24-hr long withdrawal after 15 min exposure to 20 mM ethanol. **(A)** Temporal expression profile of miR-9-5p (left bars) and miR-9-3p (right bars) measured by Taqman-based RT-qPCR. Data expressed as mean fold change ± SD. *n* = 3, except *n* = 7 in miR-9-5p control group, *n* = 5 in miR-9-3p control group). Asterisks indicate statistically significant differences comparing to the control, *p* < 0.05. **(B)** Strong, negative correlation of miR-9-5p and miR-9-3p expression levels during the first 6 h of alcohol withdrawal. Correlation coefficient r = −0.775. **(C)** Moderate, positive correlation of miR-9-5p and miR-93p expression levels during 12–24 h of alcohol withdrawal. The correlation coefficient r = 0.550.

miR-9-5p is a prominent brain microRNA regulated by alcohol. Some reports describe the stimulatory effect of alcohol on miR-9-5p expression [17, 40], while others report the opposite effects [41]. To better understand the intricacies of miR-9-5p regulation by alcohol, we first exposed murine primary neuronal cultures to physiologically relevant 20 mM ethanol for 15 min (the short exposure) and measured its expression at various times after alcohol withdrawal up to 24 h post-exposure (Figure 2A).

We observed that after the short exposure expression levels of miR-9-5p increased almost two-fold (Figure 2A, left bars), in accordance with previously published findings [17]. Alcohol withdrawal caused a fast decrease of the elevated levels of miR-9-5p even below the pre-exposure, normal levels within 1 h after the start of the exposure (Figure 2A, left bars). In the alcohol-free environment, miR-9-5p levels decreased even further with time, reaching the lowest levels of around 40% of the pre-exposure levels at the 6 h post-exposure mark. Somewhere between 6 h and 12 h of the withdrawal miR-9-5p levels started to rebound from their nadir point and went back to the pre-exposure levels (Figure 2A, left bars). They reached the pre-exposure levels 12 h after the exposure and maintained normal levels up to 24 h after the exposure (Figure 2A, left bars).

Although miR-9-5p is the most recognized final product of miR-9 biogenesis, miR-9-3p also plays an important role in neural development [42] and neuronal differentiation [43] with more predicted targets then miR-9-5p (Supplementary Table S1, miR-9-5p: 1242 targets; Supplementary Table S2, miR-9-3p: 4334 targets). Interestingly, there is a quite large overlap of targets between these two microRNAs: over 34% of miR-9-5p targets are also targeted by miR-9-3p (425 targets, Supplementary Table S3).

We observed that short alcohol exposure also regulates the expression of miR-9-3p. The short exposure increased expression of miR-9-3p (Figure 2A, right bars) similar to its effect on the miR-9-5p expression. In contrast to miR-9-5p however, after the removal of alcohol, miR-9-3p levels continue to rise, reaching significantly higher levels 6 h post-exposure (Figure 2A, right bars).

After reaching the peak of expression, miR-9-3p levels return down to pre-exposure levels at the 12 h post-exposure timepoint and maintain that normal level up to the 24 h post-exposure, mimicking temporal dynamics of miR-9-5p expression changes within 12–24 h post-exposure time interval (Figure 2A, right bars).

It seems that, based on changes in the expression of both microRNAs, two withdrawal periods triggered by short alcohol exposure could be distinguished: the early period starting immediately after alcohol withdrawal and lasting around 6 h, and the late period following the early one and lasting up to the 24-hour post-exposure timepoint (Figure 2A).

During both time periods, the expression of miR-9-5p and miR-9-3p seems to be tightly associated with each other as determined by correlation analysis. During the early withdrawal period changes in miR-9-3p and miR-9-5p expression are strongly and negatively correlated (Figure 2B; Table 1, correlation coefficient r = −0.775). During the late withdrawal period, changes in the expression of miR-9-3p and miR-9-5p are moderately and positively correlated (Figure 2C; Table 2, correlation coefficient r = 0.55).

**TABLE 1:** Correlation between expression fold change of miR-9-5p and miR-9-3p during the early period of alcohol withdrawal.

EtOH WD time [hrs]	miR-9-5p fold change	miR-9-3p fold change	r
0	1.89	1.25	- 0.775
1	0.39	1.41	
6	0.29	1.78	

EtOH WD—ethanol withdrawal, r—correlation coefficient.

**TABLE 2:** Correlation between expression fold change of miR-9-5p and miR-9-3p during the late period of alcohol withdrawal.

EtOH WD time [hrs]	miR-9-5p fold change	miR-9-3p fold change	r
12	0.74	0.78	0.550
18	0.97	1.04	
24	0.84	1.21	

EtOH WD—ethanol withdrawal, r—correlation coefficient.

### Regulation of miR-9-5p and miR-9-3p expression by continuous exposure to alcohol

We compared the short exposure results with the expression of miR-9-5p under the continuous presence of the drug for up to 24 h (continuous exposure).

We assumed that the continuous presence of the drug would maintain the elevated plateau of miR-9-5p since exposure to alcohol increased miR-9-5p levels in the first place. However, it was not the case. We observed that despite alcohol presence, after the initial increase, miR-9-5p levels dropped within 6 h post-exposure (Figure 2B, left bars) and then increased (Figure 2B, left bars) with a similar temporal dynamic seen with the short exposure. Interestingly, in the continuing presence of alcohol beyond 6 h the miR-9-5p expression pattern shifted upwards above the pre-exposure levels presumably trying to set a new, higher equilibrium (Figure 2B, left bars).

**FIGURE 3:**
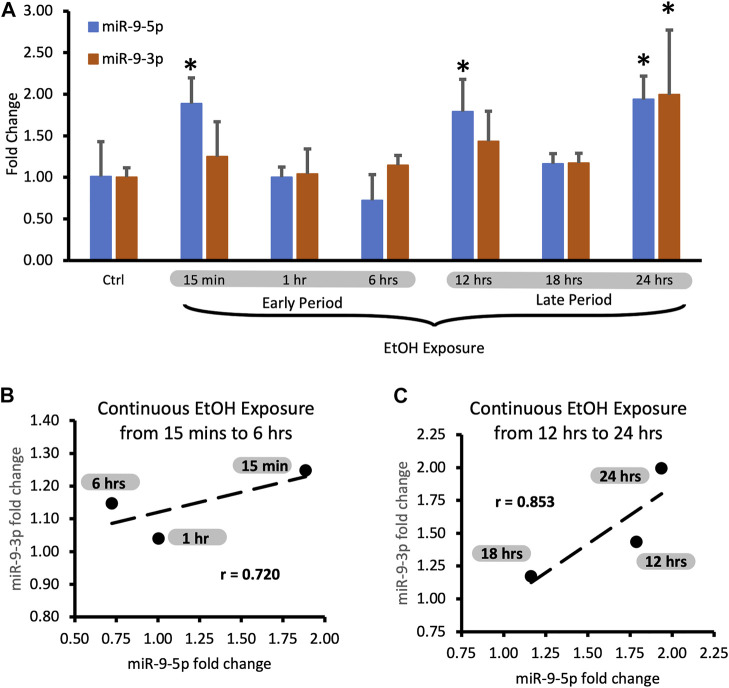
Expression levels of miR-9-5p and -3p during continuous, 24-hr long exposure to 20 mM ethanol. **(A)** Temporal expression profile of miR-9-5p (left bars) and miR-9-3p (right bars) measured by Taqman RT-qPCR. Data expressed as mean fold change ± SD. *n* = 3, except *n* = 7 in miR-9-5p control group, *n* = 5 in miR-9-5p control group). Asterisks indicate statistically significant differences comparing to the control, *p* < 0.05. **(B)** Strong, positive correlation of miR-9-5p and miR-9-3p expression levels during the first 6 h of alcohol exposure. The correlation coefficient r = 0.720. **(C)** Strong, positive correlation of miR-9-5p and miR-93p expression levels during 12–24 h of alcohol exposure. The correlation coefficient r = 0.853.

During the continuous exposure to alcohol, the miR-9-3p expression did not change sufficiently to achieve standard statistical significance (*p* < 0.05) except for the last timepoint (Figure 3A, 24 h exposure). However, the changes of the miR-9-3p expression tightly followed the changes of the miR-9-5p expression, showing a strong and positive correlation at each timepoint studied (Figure 3A). We think that two periods with similar time frames can be distinguished here as well based on changes in the expression pattern: the early exposure period starting soon after alcohol addition and lasting about 6 h (Figure 3A) with a correlation coefficient r = 0.720 (Figure 3B; Table 3), and the late exposure period following the first one up to the 24-hour of alcohol exposure (Figure 3C; Table 4) with the correlation coefficient r = 0.853.

**TABLE 3:** Correlation between expression fold change of miR-9-5p and miR-9-3p during the early period of continuous alcohol exposure.

EtOH ex time [hrs]	miR-9-5p fold change	miR-9-3p fold change	r
0.25	1.89	1.25	0.720
1	1.00	1.04	
6	0.72	1.15	

EtOH ex—ethanol exposure, r—correlation coefficient.

**TABLE 4:** Correlation between expression fold change of miR-9-5p and miR-9-3p during the late period of continuous alcohol exposure.

EtOH ex time [hrs]	miR-9-5p fold change	miR-9-3p fold change	r
12	1.79	1.43	0.853
18	1.16	1.17	
24	1.94	1.99	

EtOH ex—ethanol exposure, r—correlation coefficient.

### Regulation of expression of miR-9 precursors by short alcohol exposure

Both miR-9-5p and miR-9-3p are final products of miR-9 biogenesis (Figure 1, and ref 19). Three separate biogenesis pathways of the miR-9-5p/miR-9-3p pair start with each miR-9 gene generating its own pri-mir-9 precursor, and subsequently pre-mir-9 precursor, which ultimately contributes to the mature miR-9-5p and the mature miR-9-3p pools (Figure 1). We decided to determine the effects of both, the short and the continuous alcohol exposure, on the expression of all of these precursors.

**FIGURE 4:**
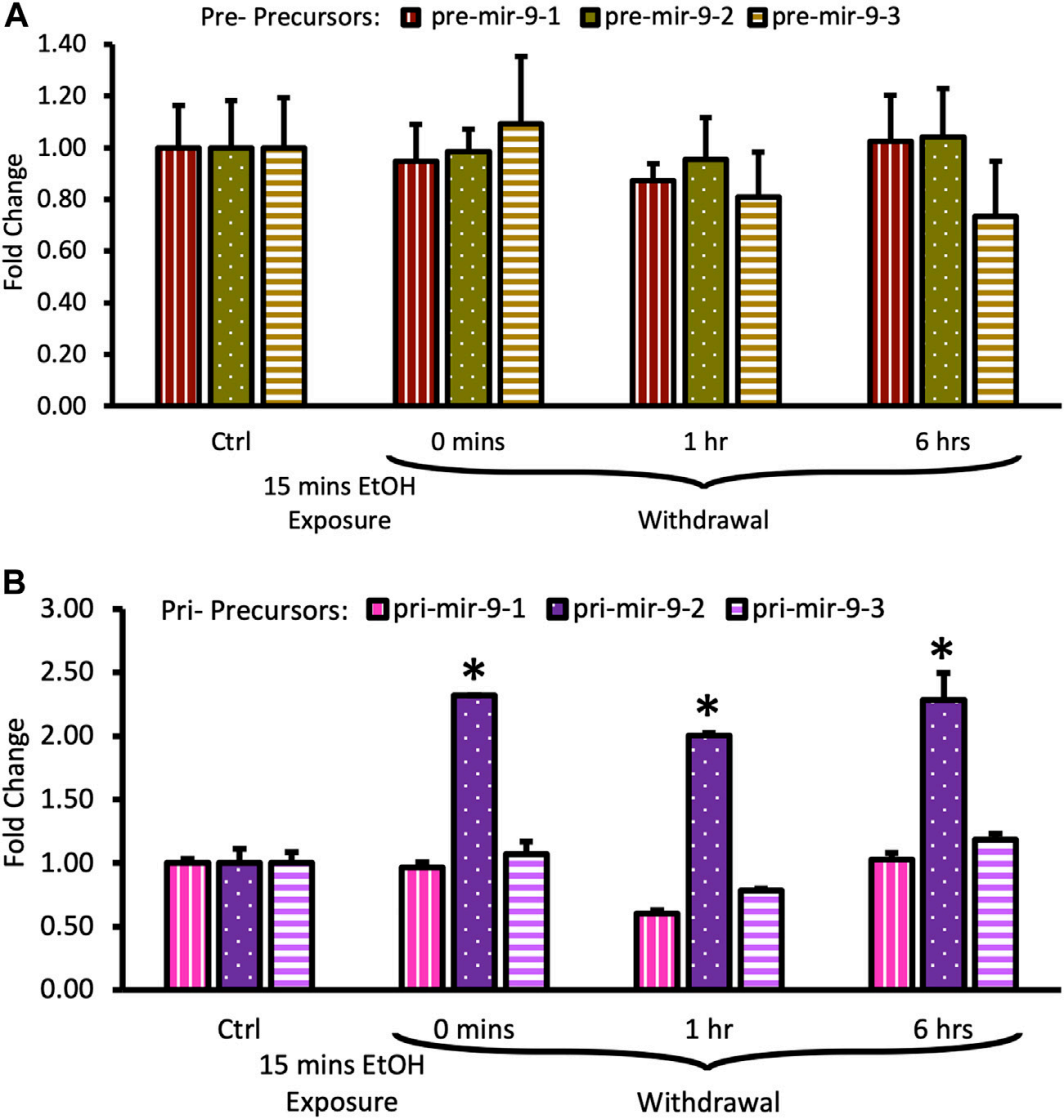
Expression levels of pre- and pri-precursors during the first 6 hours of withdrawal after 15 min exposure to 20 mM ethanol. **(A)** Temporal expression profile of pre-mir-9-1 (left bars), pre-mir-9-2 (middle bars), and pre-mir-9-3 (right bars) measured by miScript RT-PCR. **(B)** Temporal expression profile of pri-mir-9-1 (left bars), pri-mir-9-2 (middle bars), and pri-mir-9-3 (right bars) measured by QuantStudio 3D Digital RT-PCR. Data expressed as mean fold change ± SD. *n* = 3 (pre-precursors), *n* = 2 (pri-precursors). Asterisks indicate statistically significant differences comparing to the control, *p* < 0.05.

We observed that the short alcohol exposure (15 min) had no effect on the expression of all three pre-mir-9 precursors (Figure 4A). Expression levels of none of the precursors changed immediately after the alcohol exposure. Since they remained consistently at the same, unchanged level for 6 h following the alcohol withdrawal (Figure 4A) we did not explore further time points.

In contrast, within the same timeframe of the early period of withdrawal, we observed a robust, over 2-fold upregulation of pri-mir-9-2 precursor expression by short alcohol exposure immediately following the exposure (Figure 4B). The pri-mir-9-2 precursor expression upregulation was sustained for at least 6 h after the alcohol withdrawal (Figure 4B). This effect was not observable for the other two pri-precursors: pri-mir-9-1 and the pri-mir-9-3 (Figure 4B).

Overall, it seems that a short, 15 min alcohol exposure elicited changes in the expression of pri-miR-9-2 precursor only and that these changes were quick, robust, and unceasing in alcohol absence.

### Regulation of expression of miR-9 precursors by long alcohol exposure

The long (6 h) alcohol exposure affected the expression of both, pre- and pri-mir-9 precursors.

**FIGURE 5:**
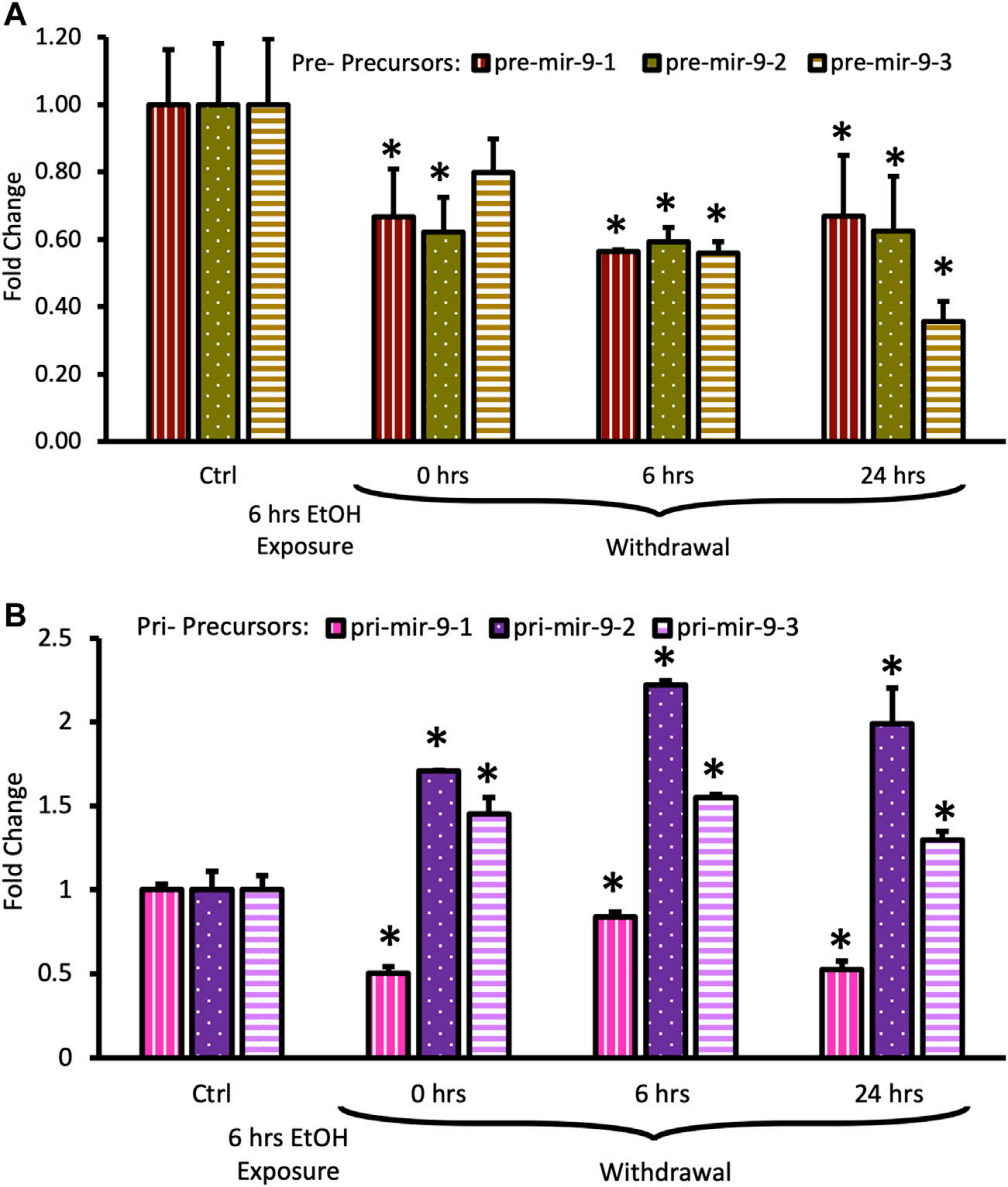
Expression levels of pre- and pri-precursors during 24 h withdrawal after 6 h of exposure to 20 mM ethanol. **(A)** Temporal expression profile of pre-mir-9-1 (left bars), pre-mir-9-2 (middle bars), and pre-mir-9-3 (right bars) measured by miScript RT-PCR. **(B)** Temporal expression profile of pri-mir-9-1 (left bars), pri-mir-9-2 (middle bars), and pri-mir-9-3 (right bars) measured by QuantStudio 3D Digital RT-PCR. Data expressed as mean fold change ± SD. *n* = 3 (pre-precursors), *n* = 2 (pri-precursors). Asterisks indicate statistically significant differences comparing to the control, *p* < 0.05.

Both, pre-mir-9-1, and pre-mir-9-2 were significantly downregulated after 6 h of alcohol exposure, with pre-mir-9-3 following this trend but not reaching a statistical significance at *p* < 0.05 yet (Figure 5A). Withdrawal of alcohol for 6 h after the 6 hr-long exposure to the drug did not restore expression levels of any of the pre-mir-9 precursors with all of them being decreased. The decreased expressions of all three pre-miR-9 precursors continued in the absence of alcohol for up to 24 h after alcohol withdrawal (Figure 5A).

The effects of the long (6 h) alcohol exposure on the expression levels of pri-miR-9 precursors also affected all of these precursors but each in a different way (Figure 5B). The expression of the pri-mir-9-1 precursor was consistently downregulated to about 50% of its pre-exposure levels, and this downregulation persisted in the absence of alcohol for up to 24 h after alcohol withdrawal (Figure 5B). In contrast, the expression of the remaining two pri-precursors (pri-mir-9-2, pri-mir-9-3) was significantly upregulated by the long (6 h) alcohol exposure to about 1.5-fold above their pre-exposure levels. After alcohol withdrawal, the upregulated levels of both pri-precursors were sustained (Figure 5B). The pri-miR-9-3 precursor maintained its 1.5-fold upregulation at both, 6 h and 24 h after alcohol withdrawal (Figure 5B), while the pri-miR-9-2 precursor expression levels 6 h after alcohol withdrawal went even further up, reaching above 2-fold upregulation, and maintaining their higher expression levels 24 h post-exposure (Figure 5B).

Overall, it seems that longer alcohol exposure elicited wider changes in the expression of miR-9 precursors, affecting the expression of all precursors. Nevertheless, it seems that the miR-9-2 biogenesis pathway responded in the most striking way.

## Discussion

Alcohol Use Disorder (AUD) is a very complex disease involving an array of biomolecules, multiple biological pathways, and several organismal systems. Time is a fundamental factor of alcohol-triggered changes in the brain’s function as the development of AUD is happening progressively over time. We have attempted to shed some light on the temporal regulation of the biogenesis of miR-9, one of the key master regulators of gene expression in the brain [19], which is affected by alcohol in both, brain development [44, 45] and mature brain function [17, 46] and exists in two biologically active forms: miR-9-5p and miR-9-3p. We measured changes of both mature miR-9 forms in murine, primary cell culture consisting of Medium Spiny Neurons (MSN) derived from the Nucleus Accumbens (NAc), which is a part of the brain reward system integrating information from the cortex and subcortical regions [47–49] and highjacked by alcohol in AUD [50]. Alcohol affects the activity of MSN [51], disrupts information integration, and causes behavioral effects [52].

Although during intoxication, neurons in the brain can be exposed to a wide range of alcohol concentrations from around 10 mM to over 100 mM, 20 mM ethanol concentration has a low apoptotic effect [17, 36] yet significant effects on the CNS neurobiology (e.g., ion channel conductivity, neuronal excitability, neuronal network activity), morphology (e.g., synaptic shape and mitochondrial density [53], and behavior (e.g., sedation, motor incoordination, inability to operate motor vehicles, consistent with intoxication) [54]. We reported previously that exposure of the rat neurohypophysial brain explant to 20 mM alcohol for a short time (15 min) caused an upregulation of miR-9-5p expression and observable changes in expression of some of miR-9-5p targets, including the rearrangement of BK channel splice variants consistent with neuroadaptation [17]. Here, we extended our studies to determine temporal characteristics of miR-9 adaptation to alcohol using murine primary neuronal cultures of medium spiny neurons harvested from the striatum, allowing precise control over alcohol exposure and withdrawal of the pivotal element of the brain reward system.

### miR-9-5p homeostatic response to short alcohol exposure and withdrawal

After observing previously the biological effects of a short exposure to 20 mM alcohol [17], we questioned whether the upregulated miR-9-5p levels persist after alcohol withdrawal and for how long. We determined here that the short alcohol exposure triggered changes in miR-9-5p expression observable during withdrawal. These changes could be divided into two, subsequent phases: 1/downregulation below the pre-exposure level, and 2/upregulation to the pre-exposure level. These phases seem to follow a pattern of homeostatic regulation, during which miR-9-5p levels thrown off of the steady state equilibrium by alcohol exposure would undergo changes after alcohol withdrawal to return eventually to the pre-exposure *status quo*. Based on our collection time points we observed that MSN neurons need roughly around 6–12 h of the drug withdrawal to return miR-9-5p levels to normal (i.e., the pre-exposure steady-state equilibrium). It would be of interest, in the next studies, to further narrow down the time window of this homeostatic adaptation.

### miR-9-3p upregulation attenuates the effects of the miR-9-5p downregulation

Recently, miR-9-3p, the passenger strand derived from the same duplex as miR-9-5p, gained recognition as biologically active on its own [43, 55]. Martinez et al. [56] showed that chronic ethanol exposure over the course of 55 days elevated miR-9-3p in the serum of rats. Balaraman proposed that the ratio between these two mature microRNAs is important in the regulation of neuronal differentiation and in the development of cancer [57]. Both microRNAs impact the differentiation of neural stem cells through the co-regulation of a transcription factor, REST (RE1 silencing transcription factor/neuron-restrictive silencer factor). miR-9-5p targets REST directly, while miR-9-3p regulates the expression of coREST, a cofactor of REST [42, 57]. Therefore, miR-9-5p and miR-9-3p working in tandem can create various combinations of REST:coREST, thus influencing neuronal differentiation [58, 59].

Our results postulate an even tighter, joint effect of miR-9-5p and miR-9-3p on gene expression. Simultaneous downregulation of miR-9-5p and upregulation of miR-9-3p observed at some points, and their convergence on a large number of targets (425 transcripts, over 34% of miR-9-5p targets) could be a neuronal attempt to attenuate, at least some acute alcohol effects on miR-9-5p targets, consistent with a homeostatic response and preservation of pre-exposure equilibrium. We also would like to propose that any future studies focused on the regulation of miR-9-5p and its targets by alcohol or other factors should include miR-9-3p and its targets as well.

### Coordinated miR-9-5p/-3p allostatic response to continuous alcohol exposure

We expected that in the continuous presence of alcohol, upregulated levels of miR-9-5p and miR-9-3p would be maintained. However, that was not the case. We observed that despite the continuous presence of alcohol, both miR-9-5p and miR-9-3p levels followed a response pattern, similar to one observed in a short exposure/withdrawal experiment, which could be also divided into two phases: 1/initial downregulation, 2/subsequent upregulation, with a demarcation line between these two phases happening after 6–12 h of alcohol exposure. Interestingly, the final outcome after 24 h of alcohol exposure was a significant upregulation of both miR-9-5p and miR-9-3p. One could interpret these results as a neuronal adaptation at the molecular level to the continuous presence of alcohol by attempting to set up a new, overcorrected set-point of miR-9-5p and miR-9-3p expression despite the continued presence of the drug. This is consistent with achieving new stability through change—a tenet of allostasis [60, 61] and the allostatic model of addiction [62].

### Regulation of miR-9 precursors and biogenesis pathways by alcohol

Since we observed the presence of the initial phase triggered by a short or continuous exposure lasting about 6 h, we decided to determine whether alcohol differently affects upstream elements of the miR-9 biogenesis pathway (precursors) during that window using two scenarios. First, we used this window as a withdrawal window preceded by the short exposure, second, we used it as an exposure window followed by a 24-hour withdrawal period. As we observed no measurable effect on all three miR-9 pre-precursors’ expression levels during the first scenario, we concluded that the likelihood of alcohol affecting the steps of microRNA biogenesis responsible for the production of pre-precursors from pri-precursors is rather low. However, in scenario 2, alcohol downregulated all three miR-9 pre-precursors suggesting a possibility that alcohol could interfere with one or some of the steps producing pre-precursors from pri-precursors. Production of microRNA pre-precursors starts with pri-precursors cleavage by the Microprocessor machinery, followed by export from the nucleus to the cytoplasm by the exportin5 complex, and capture by Dicer for further processing by the RISC complex [63]. There are many proteins involved in microRNA biogenesis as each microRNA processing complex consists of several proteins. The microprocessor contains Drosha, DGCR8, RIIa and RIIb proteins, and Exportin-5, a mediator of nuclear export that needs a cofactor RanGTP protein [63], while Dicer works with auxiliary proteins TRBP and members of the Argonaute protein family (AGO) to form the RISC complex [63]. It is possible that some of the regulation of precursors by alcohol reported here is due to an alcohol effect on some of these proteins. Indeed, Mulligan [64] showed an association between Drosha and Dicer expression and response to alcohol, while Prins [65] determined that, in the rat hippocampus, alcohol alters Drosha and Dicer expression (also see 18). Moreover, Gedik [66] reported a genetic association of DGCR8, AGO1, and AGO2 alleles with alcohol dependence risk. It would be of great interest to gain a full picture of alcohol regulation of activity of the key elements of the microRNA biogenesis complexes which process precursors.

In order to better understand the temporal regulation of miR-9 expression by alcohol we should also focus our future efforts on the initial steps of the biogenesis, namely, the production of the miR-9 pri-precursors from their respective genes. Our results revealed that even the short alcohol exposure triggered upregulation of pri-mir-9-2, while longer exposure affected the expression of all three miR-9 pri-precursors. At this point we cannot rule out any mechanisms regulating miR-9 gene expression; however, we suspect epigenetic control of the mir-9-2 gene expression by alcohol. Pappalardo-Carter et al. [67] showed that alcohol increases CpG dinucleotide methylation of the mir-9-2 gene promoter. We believe that further, comprehensive studies of the epigenetic regulation of all miR-9 genes by alcohol are fully warranted.

Further studies should also shed some light on the differential regulation of miR-9 expression not only by the temporal aspect of alcohol exposure but also by alcohol concentration. Pappalardo-Carter [67] reported that a high alcohol concentration (130 mM) suppressed miR-9 expression, while Tapocik [68] showed that an alcohol concentration of 70 mM inhibited the expression of miR-9, creating a lower steady-state level in alcohol-dependent rats.

AUD is known to have a genetic component [69, 70]. Because human miR-9-1 and miR-9-3 genes are located near or within the AUD susceptibility loci [8], we believe that exploring the differential effects of alcohol on each miR-9 biogenesis pathway is also of great importance in understanding the genetic predisposition to AUD. We would hypothesize that the first response to alcohol exposure is mostly through the mir-9-2 biogenesis pathway. However, with continuous exposure (longer than 6 h) or possibly multiple exposures (mimicking frequent drinking characteristic of the AUD) the mir-9-2 gene may be eventually substantially turned down, with the remaining contribution shifting to mir-9-1 and mir-9-3 genes. As these two genes combined produce less miR-9 than mir-9-2, this hypothesis would explain lower levels of miR-9 observed in chronic alcohol exposure experiments [67, 68]. This hypothesis would also rationalize the presence of miR-9-1 and miR-9-3 genes in the AUD susceptibility loci.

It is worth mentioning that a deeper understanding of alcohol regulation of miR-9 biogenesis would also benefit research focused on cancer and neurodegenerative diseases. Aberrant levels of miR-9 (either miR-9-5p, miR-9-3p, or both) have been reported in many types of cancer [71]: breast cancer [25, 72, 73], Burkitt’s Lymphoma [24], hepatocarcinoma [74], prostate cancer [75], gastric cancer [76], colorectal cancer [77], as well as Alzheimer’s and Huntington’s diseases [78]. Chronic heavy alcohol consumption increases the risk of all of these cancers and neurodegenerative diseases [79–86].

## Summary

AUD is a progressive brain disease. Understanding the temporal effects of alcohol on gene expression in neurons is of great importance. Using murine primary cultures of medium spiny neurons, we attempted to deepen our understanding of temporal regulation by alcohol of expression and biogenesis of miR-9-5p and miR-9-3p, key regulators of gene expression. Based on miR-9-5p and miR-9-3p responses to short alcohol exposure, we concluded that changes in expression of these two microRNAs seem to be consistent with the homeostatic model of addiction, while longer, continuous alcohol exposure evoked possibly allostatic changes. Finally, our results point out that the sensitivity of mir-9 genes to alcohol varies among genes and is also time-dependent. The mir-9-2 gene produces pri-mir-9-2 precursor almost immediately after alcohol exposure, while mir-9-1 and mir-9-3 genes need longer exposure to alcohol. Our studies may help us to understand better mechanisms of addiction, carcinogenesis, and neurodegenerative disorders.

## Limitations and future directions

There are several limitations to consider when interpreting the results. We used the primary neuronal culture of the medium spiny neurons harvested from young mice pups’ striatum. One needs to remember that neurons harvested in such a way are taken away from their natural environment of the whole brain “connected” to the whole animal. To preserve more “natural” conditions we could use brain striatal slices, however, their viability over 24 h is poor; we could also consider using whole animals, however, in this model, it is impossible to precisely control alcohol exposure and withdrawal. Thus, with its inherited limitations, this model provides us with precise control over alcohol exposure and withdrawal, as well as direct access to neurons derived from the striatum - a pivotal element of the reward system, which plays a fundamental role in the development of addiction.

Another limiting factor is that neurons harvested from newborn pups are not mature yet and for about 2 weeks correspond to the final *in utero* period of human development. However, we waited 5 days to harvest the neurons from the striata of P5 pups and then cultivated them on a dish for 8 days before starting alcohol exposure, thus likely passing the period corresponding to the *in utero* human development.

Another factor to remember is that neurons during a few days after plating undergo proliferation on the culture dish. It has been shown in another model that in the proliferating neurons of the retina miR-9 levels (presumably miR-9-5p) oscillate with a rhythmicity of 3 h [87] meaning the expression of miR-9 follows a sinusoid with the same levels observed every 3 hours. This rhythmicity is transient and stabilizes once the neurons mature. Cultivating neurons on a dish for about a week yields mostly mature neurons. However, it is possible that there are some proliferating neurons still present. Since most of our collection time points were multiplications of three, they were in sync with miR-9 oscillations, thus any miR-9 rhythmicity should have a minimal effect. Therefore, by harvesting MSN from the P5 pups and allowing them for a few days to mature before starting alcohol exposure, we think that we were able to circumnavigate at least some of the shortcomings of this model. Future collections with time intervals shorter than 3 h (or not in sync with 3 h) will require though additional controls.

We measured the expression of miR-9 precursors which are products of mir-9 genes and biogenesis machinery but did not directly study the regulation of gene expression or the machinery activity. Future studies could focus on a systematic approach of determining the alcohol sensitivity of individual elements of microRNA biogenesis (e.g., using antisense oligonucleotides targeting each precursor individually) including temporal characteristics of epigenetic regulation of gene expression by alcohol.

Lastly, we used a single, low-dose alcohol concentration to minimize cellular death. Since higher alcohol concentrations have been shown to also regulate miR-9 expression [67, 68] determination of their effects on miR-9 biogenesis would be of interest.

## Data Availability

The raw data supporting the conclusion of this article will be made available by the authors, without undue reservation.
