# Supplementary material for: miR-9 utilizes precursor pathways in adaptation to alcohol in mouse striatal neurons
Source: Adv Drug Alcohol Res. Author manuscript; Available in PMC 2023 Dec 19. (PMC10730111; doi:10.3389/adar.2023.11323)
Supplement: Mead et al SM Table 3 [file NIHMS1906815-supplement-Mead_et_al_SM_Table_3.pdf]

Supplementary Materials (SM), Table 3
List of overlapping targets of both, miR-9-5p and miR-9-3p

#	Target gene	Representative transcript	Gene name
1	1110002E22Rik	ENSMUST00000163080.2	RIKEN cDNA 1110002E22 gene
2	1200014J11Rik	ENSMUST00000021135.3	RIKEN cDNA 1200014J11 gene
3	2310035C23Rik	ENSMUST00000086721.4	RIKEN cDNA 2310035C23 gene
4	4933426M11Rik	ENSMUST00000068519.5	RIKEN cDNA 4933426M11 gene
5	Aatk	ENSMUST00000064307.4	apoptosis-associated tyrosine kinase
6	Abi1	ENSMUST00000140164.2	abl-interactor 1
7	Acot7	ENSMUST00000167926.2	acyl-CoA thioesterase 7
8	Acvr1b	ENSMUST00000000544.10	activin A receptor, type 1B
9	Adam10	ENSMUST00000067880.7	a disintegrin and metallopeptidase domain 10
10	Adamts5	ENSMUST00000023611.5	a disintegrin-like and metallopeptidase (reprolysin type) with thrombospondin type 1 motif, 5 (aggrecanase-2)
11	Adcy9	ENSMUST00000117801.2	adenylate cyclase 9
12	Aff1	ENSMUST00000054979.4	AF4/FMR2 family, member 1
13	Agfg1	ENSMUST00000189220.1	ArfGAP with FG repeats 1
14	Ahdc1	ENSMUST00000105916.2	AT hook, DNA binding motif, containing 1
15	AI118078	ENSMUST00000085754.4	expressed sequence AI118078
16	Ak4	ENSMUST00000102780.2	adenylate kinase 4
17	Akr1b3	ENSMUST00000102980.5	aldo-keto reductase family 1, member B3 (aldose reductase)
18	Amer1	ENSMUST00000084535.5	APC membrane recruitment 1
19	Amigo2	ENSMUST00000053106.5	adhesion molecule with Ig like domain 2
20	Ammecr1	ENSMUST00000041317.2	Alport syndrome, mental retardation, midface hypoplasia and elliptocytosis chromosomal region gene 1
21	Amotl1	ENSMUST00000160770.2	angiomotin-like 1
22	Ank	ENSMUST00000022875.6	progressive ankylosis
23	Ankrd40	ENSMUST00000051221.7	ankyrin repeat domain 40
24	Ar	ENSMUST00000052837.7	androgen receptor
25	Arhgap31	ENSMUST00000023487.4	Rho GTPase activating protein 31
26	Armcx2	ENSMUST00000119010.2	armadillo repeat containing, X-linked 2
27	Asxl3	ENSMUST00000097655.3	additional sex combs like 3 (Drosophila)
28	Atp7a	ENSMUST00000055941.6	ATPase, Cu++ transporting, alpha polypeptide
29	Axl	ENSMUST00000002677.5	AXL receptor tyrosine kinase
30	B230219D22Rik	ENSMUST00000057844.8	RIKEN cDNA B230219D22 gene
31	B3galnt2	ENSMUST00000099747.3	UDP-GalNAc:betaGlcNAc beta 1,3-galactosaminyltransferase, polypeptide 2
32	Bag4	ENSMUST00000038498.8	BCL2-associated athanogene 4
33	Bahd1	ENSMUST00000036578.6	bromo adjacent homology domain containing 1
34	Baz2b	ENSMUST00000112550.2	bromodomain adjacent to zinc finger domain, 2B
35	BC007180	ENSMUST00000189969.1	cDNA sequence BC007180
36	Bcl6	ENSMUST00000023151.5	B cell leukemia/lymphoma 6
37	Bend4	ENSMUST00000169190.1	BEN domain containing 4
38	Bicc1	ENSMUST00000143791.2	bicaudal C homolog 1 (Drosophila)
39	Bnc2	ENSMUST00000176612.2	basonuclin 2
40	Btbd7	ENSMUST00000045652.6	BTB (POZ) domain containing 7
41	Btg2	ENSMUST00000020692.6	B cell translocation gene 2, anti-proliferative
42	Cacna1e	ENSMUST00000187541.1	calcium channel, voltage-dependent, R type, alpha 1E subunit
43	Calml4	ENSMUST00000034777.7	calmodulin-like 4
44	Camta1	ENSMUST00000097774.3	calmodulin binding transcription activator 1
45	Capza1	ENSMUST00000094028.5	capping protein (actin filament) muscle Z-line, alpha 1

46	Cbfa2t2	ENSMUST00000109725.2	core-binding factor, runt domain, alpha subunit 2, translocated to, 2 (human)
47	Cbfa2t3	ENSMUST00000127984.2	core-binding factor, runt domain, alpha subunit 2, translocated to, 3 (human)
48	Cbx5	ENSMUST00000118152.2	chromobox 5
49	Ccdc50	ENSMUST00000100026.4	coiled-coil domain containing 50
50	Ccne1	ENSMUST00000108023.4	cyclin E1
51	Ccnt2	ENSMUST00000112570.1	cyclin T2
52	Ccr6	ENSMUST00000164411.3	chemokine (C-C motif) receptor 6
53	Ccser1	ENSMUST00000126214.2	coiled-coil serine rich 1
54	Ccser2	ENSMUST00000090024.5	coiled-coil serine rich 2
55	Cdc14b	ENSMUST00000109770.1	CDC14 cell division cycle 14B
56	Cdc73	ENSMUST00000018337.8	cell division cycle 73, Paf1/RNA polymerase II complex component
57	Cdh10	ENSMUST00000166873.3	cadherin 10
58	Cdh11	ENSMUST00000075190.3	cadherin 11
59	Cdk13	ENSMUST00000042365.7	cyclin-dependent kinase 13
60	Cdk8	ENSMUST00000031640.9	cyclin-dependent kinase 8
61	Cdyl2	ENSMUST00000109102.2	chromodomain protein, Y chromosome-like 2
62	Cep170b	ENSMUST00000101018.4	centrosomal protein 170B
63	Ces1c	ENSMUST00000034189.9	carboxylesterase 1C
64	Clmn	ENSMUST00000109937.2	calmin
65	Clock	ENSMUST00000075159.1	circadian locomotor output cycles kaput
66	Cmah	ENSMUST00000167746.2	cytidine monophospho-N-acetylneuraminic acid hydroxylase
67	Cmtm6	ENSMUST00000035007.8	CKLF-like MARVEL transmembrane domain containing 6
68	Cmtr2	ENSMUST00000056972.5	cap methyltransferase 2
69	Cnot6l	ENSMUST00000155901.2	CCR4-NOT transcription complex, subunit 6-like
70	Cnot7	ENSMUST00000034012.4	CCR4-NOT transcription complex, subunit 7
71	Colec12	ENSMUST00000040069.8	collectin sub-family member 12
72	Cpeb3	ENSMUST00000079754.5	cytoplasmic polyadenylation element binding protein 3
73	Cpeb4	ENSMUST00000109412.3	cytoplasmic polyadenylation element binding protein 4
74	Crebrf	ENSMUST00000062519.8	CREB3 regulatory factor
75	Csnk1a1	ENSMUST00000165123.2	casein kinase 1, alpha 1
76	Ctdsp2	ENSMUST00000105256.3	CTD (carboxy-terminal domain, RNA polymerase II, polypeptide A) small phosphatase 2
77	Ctnnal1	ENSMUST00000045142.9	catenin (cadherin associated protein), alpha-like 1
78	Ctps2	ENSMUST00000033727.8	cytidine 5'-triphosphate synthase 2
79	Cttnbp2nl	ENSMUST00000077548.6	CTTNBP2 N-terminal like
80	Cxcl2	ENSMUST00000075433.6	chemokine (C-X-C motif) ligand 2
81	Cyhr1	ENSMUST00000081291.7	cysteine and histidine rich 1
82	D15Ertd621e	ENSMUST00000037270.3	DNA segment, Chr 15, ERATO Doi 621, expressed
83	D16Ertd472e	ENSMUST00000114220.2	DNA segment, Chr 16, ERATO Doi 472, expressed
84	D1Ertd622e	ENSMUST00000053033.8	DNA segment, Chr 1, ERATO Doi 622, expressed
85	D230025D16Rik	ENSMUST00000034361.4	RIKEN cDNA D230025D16 gene
86	D430041D05Rik	ENSMUST00000089726.4	RIKEN cDNA D430041D05 gene
87	Dcbld2	ENSMUST00000046663.7	discoidin, CUB and LCCL domain containing 2
88	Dcc	ENSMUST00000114943.4	deleted in colorectal carcinoma
89	Dcp1a	ENSMUST00000022535.7	DCP1 decapping enzyme homolog A (S. cerevisiae)
90	Dcp1b	ENSMUST00000112777.3	DCP1 decapping enzyme homolog B (S. cerevisiae)
91	Dcun1d4	ENSMUST00000063882.6	DCN1, defective in cullin neddylation 1, domain containing 4 (S. cerevisiae)
92	Ddhd2	ENSMUST00000033975.6	DDHD domain containing 2

93	Ddi2	ENSMUST00000102484.4	DNA-damage inducible protein 2
94	Ddx3x	ENSMUST00000000804.6	DEAD/H (Asp-Glu-Ala-Asp/His) box polypeptide 3, X-linked
95	Dennd1a	ENSMUST00000102787.4	DENN/MADD domain containing 1A
96	Dgkb	ENSMUST00000040500.7	diacylglycerol kinase, beta
97	Dhx40	ENSMUST00000018569.8	DEAH (Asp-Glu-Ala-His) box polypeptide 40
98	Dicer1	ENSMUST00000041987.6	dicer 1, ribonuclease type III
99	Dio2	ENSMUST00000082432.3	deiodinase, iodothyronine, type II
100	Dlgap2	ENSMUST00000133298.2	discs, large (Drosophila) homolog-associated protein 2
101	Dnaja4	ENSMUST00000070070.7	DnaJ (Hsp40) homolog, subfamily A, member 4
102	Dock9	ENSMUST00000100299.4	dedicator of cytokinesis 9
103	E130308A19Rik	ENSMUST00000070150.5	RIKEN cDNA E130308A19 gene
104	Egr3	ENSMUST00000035908.1	early growth response 3
105	Eif4e	ENSMUST00000029803.7	eukaryotic translation initiation factor 4E
106	Eif4e3	ENSMUST00000032151.2	eukaryotic translation initiation factor 4E member 3
107	Eif5	ENSMUST00000166123.2	eukaryotic translation initiation factor 5
108	Elmod2	ENSMUST00000177594.2	ELMO/CED-12 domain containing 2
109	Emb	ENSMUST00000022242.7	embigin
110	Enah	ENSMUST00000078719.7	enabled homolog (Drosophila)
111	Enpep	ENSMUST00000029658.8	glutamyl aminopeptidase
112	Epha7	ENSMUST00000029964.6	Eph receptor A7
113	Esyt2	ENSMUST00000100986.2	extended synaptotagmin-like protein 2
114	Ets1	ENSMUST00000034534.7	E26 avian leukemia oncogene 1, 5' domain
115	Fam126b	ENSMUST00000161600.2	family with sequence similarity 126, member B
116	Fam135b	ENSMUST00000022953.8	family with sequence similarity 135, member B
117	Fam199x	ENSMUST00000047852.7	family with sequence similarity 199, X-linked
118	Fam46a	ENSMUST00000034802.9	family with sequence similarity 46, member A
119	Fam46c	ENSMUST00000061455.8	family with sequence similarity 46, member C
120	Fam46d	ENSMUST00000101292.3	family with sequence similarity 46, member D
121	Fbn2	ENSMUST00000025497.6	fibrillin 2
122	Fbxl16	ENSMUST00000045692.7	F-box and leucine-rich repeat protein 16
123	Fbxl17	ENSMUST00000024761.7	F-box and leucine-rich repeat protein 17
124	Fbxl3	ENSMUST00000022720.9	F-box and leucine-rich repeat protein 3
125	Fbxo28	ENSMUST00000051431.4	F-box protein 28
126	Fbxo33	ENSMUST00000043204.7	F-box protein 33
127	Fbxw2	ENSMUST00000028220.4	F-box and WD-40 domain protein 2
128	Fgf12	ENSMUST00000100024.1	fibroblast growth factor 12
129	Fgf14	ENSMUST00000095529.4	fibroblast growth factor 14
130	Fgf9	ENSMUST00000165526.2	fibroblast growth factor 9
131	Fignl2	ENSMUST00000178140.1	fidgetin-like 2
132	Fktn	ENSMUST00000128667.2	fukutin
133	Fli1	ENSMUST00000016231.8	Friend leukemia integration 1
134	Flrt1	ENSMUST00000113383.2	fibronectin leucine rich transmembrane protein 1
135	Flrt3	ENSMUST00000110057.2	fibronectin leucine rich transmembrane protein 3
136	Foxi3	ENSMUST00000069634.5	forkhead box I3
137	Foxp2	ENSMUST00000115477.2	forkhead box P2
138	Frem2	ENSMUST00000091137.4	Fras1 related extracellular matrix protein 2
139	Fry	ENSMUST00000087204.5	furry homolog (Drosophila)
140	Fsd1l	ENSMUST00000132151.2	fibronectin type III and SPRY domain containing 1-like
141	Fst	ENSMUST00000022287.5	follistatin
142	Fstl1	ENSMUST00000114763.2	follistatin-like 1
143	Fxr1	ENSMUST00000001620.8	fragile X mental retardation gene 1, autosomal homolog

144	Fyco1	ENSMUST00000167595.3	FYVE and coiled-coil domain containing 1
145	Fzd7	ENSMUST00000114246.3	frizzled homolog 7 (Drosophila)
146	Gabrb2	ENSMUST00000007797.4	gamma-aminobutyric acid (GABA) A receptor, subunit beta 2
147	Gabrg3	ENSMUST00000068911.7	gamma-aminobutyric acid (GABA) A receptor, subunit gamma 3
148	Gls	ENSMUST00000114510.2	glutaminase
149	Gm12353	ENSMUST00000108250.2	predicted gene 12353
150	Gm9804	ENSMUST00000056234.3	predicted gene 9804
151	Gnai1	ENSMUST00000074694.5	guanine nucleotide binding protein (G protein), alpha inhibiting 1
152	Gnpnat1	ENSMUST00000046191.7	glucosamine-phosphate N-acetyltransferase 1
153	Gopc	ENSMUST00000105475.3	golgi associated PDZ and coiled-coil motif containing
154	Gpalpp1	ENSMUST00000022585.3	GPALPP motifs containing 1
155	Gpatch8	ENSMUST00000143842.1	G patch domain containing 8
156	Gpc6	ENSMUST00000078849.5	glypican 6
157	Gpr137c	ENSMUST00000146150.1	G protein-coupled receptor 137C
158	Gprasp2	ENSMUST00000173804.2	G protein-coupled receptor associated sorting protein 2
159	Grhl1	ENSMUST00000020985.8	grainyhead-like 1 (Drosophila)
160	Grin2a	ENSMUST00000115835.2	glutamate receptor, ionotropic, NMDA2A (epsilon 1)
161	Gtpbp2	ENSMUST00000024748.8	GTP binding protein 2
162	Gucy1a2	ENSMUST00000115733.1	guanylate cyclase 1, soluble, alpha 2
163	Gzf1	ENSMUST00000028928.7	GDNF-inducible zinc finger protein 1
164	Hiat1	ENSMUST00000029570.6	hippocampus abundant gene transcript 1
165	Hic2	ENSMUST00000090190.6	hypermethylated in cancer 2
166	Hipk1	ENSMUST00000118317.2	homeodomain interacting protein kinase 1
167	Hipk2	ENSMUST00000161779.2	homeodomain interacting protein kinase 2
168	Hipk3	ENSMUST00000028600.8	homeodomain interacting protein kinase 3
169	Hk2	ENSMUST00000000642.5	hexokinase 2
170	Hs3st3b1	ENSMUST00000094103.3	heparan sulfate (glucosamine) 3-O-sulfotransferase 3B1
171	Hspa13	ENSMUST00000114244.1	heat shock protein 70 family, member 13
172	Htr1f	ENSMUST00000063076.4	5-hydroxytryptamine (serotonin) receptor 1F
173	Hyal4	ENSMUST00000031691.2	hyaluronoglucosaminidase 4
174	Id4	ENSMUST00000021810.1	inhibitor of DNA binding 4
175	Ift52	ENSMUST00000018002.7	intraflagellar transport 52
176	Ift80	ENSMUST00000107812.2	intraflagellar transport 80
177	Igf2bp1	ENSMUST00000013559.2	insulin-like growth factor 2 mRNA binding protein 1
178	Ikzf4	ENSMUST00000133342.2	IKAROS family zinc finger 4
179	Ikzf5	ENSMUST00000046306.9	IKAROS family zinc finger 5
180	Il1rapl1	ENSMUST00000113966.2	interleukin 1 receptor accessory protein-like 1
181	Ildr2	ENSMUST00000111416.1	immunoglobulin-like domain containing receptor 2
182	Inhbb	ENSMUST00000038765.5	inhibin beta-B
183	Itga6	ENSMUST00000028522.4	integrin alpha 6
184	Itm2b	ENSMUST00000022704.7	integral membrane protein 2B
185	Jup	ENSMUST00000001592.9	junction plakoglobin
186	Kcna4	ENSMUST00000037012.2	potassium voltage-gated channel, shaker-related subfamily, member 4
187	Kcnj2	ENSMUST00000042970.2	potassium inwardly-rectifying channel, subfamily J, member 2
188	Kcnj3	ENSMUST00000112632.1	potassium inwardly-rectifying channel, subfamily J, member 3

189	Kcnn3	ENSMUST00000000811.7	potassium intermediate/small conductance calcium-activated channel, subfamily N, member 3
190	Kcnq2	ENSMUST00000149964.3	potassium voltage-gated channel, subfamily Q, member 2
191	Kctd10	ENSMUST00000102581.5	potassium channel tetramerisation domain containing 10
192	Kctd12	ENSMUST00000184744.1	potassium channel tetramerisation domain containing 12
193	Kdm7a	ENSMUST00000002305.8	lysine (K)-specific demethylase 7A
194	Kif1b	ENSMUST00000030806.5	kinesin family member 1B
195	Kif26b	ENSMUST00000160789.1	kinesin family member 26B
196	Kirrel	ENSMUST00000159976.2	kin of IRRE like (Drosophila)
197	Kitl	ENSMUST00000105283.2	kit ligand
198	Klf13	ENSMUST00000063694.8	Kruppel-like factor 13
199	Klhdc10	ENSMUST00000068259.6	kelch domain containing 10
200	Klhl42	ENSMUST00000036003.7	kelch-like 42
201	Kpnb1	ENSMUST00000001479.4	karyopherin (importin) beta 1
202	Krt222	ENSMUST00000103132.4	keratin 222
203	Lancl3	ENSMUST00000069763.2	LanC lantibiotic synthetase component C-like 3 (bacterial)
204	Lepre1	ENSMUST00000030393.7	leprecan 1
205	Lifr	ENSMUST00000171588.1	leukemia inhibitory factor receptor
206	Lpp	ENSMUST00000038053.7	LIM domain containing preferred translocation partner in lipoma
207	Lrig2	ENSMUST00000046316.6	leucine-rich repeats and immunoglobulin-like domains 2
208	Lrrc1	ENSMUST00000183873.2	leucine rich repeat containing 1
209	Man1a2	ENSMUST00000008907.8	mannosidase, alpha, class 1A, member 2
210	Map3k2	ENSMUST00000096575.3	mitogen-activated protein kinase kinase kinase 2
211	Marcks	ENSMUST00000092584.5	myristoylated alanine rich protein kinase C substrate
212	Mbnl1	ENSMUST00000099087.2	muscleblind-like 1 (Drosophila)
213	Mcmbp	ENSMUST00000057557.8	MCM (minichromosome maintenance deficient) binding protein
214	Mctp2	ENSMUST00000079323.6	multiple C2 domains, transmembrane 2
215	Mdga2	ENSMUST00000113942.2	MAM domain containing glycosylphosphatidylinositol anchor 2
216	Megf10	ENSMUST00000075770.7	multiple EGF-like-domains 10
217	Memo1	ENSMUST00000078459.6	mediator of cell motility 1
218	Mfap3l	ENSMUST00000160719.2	microfibrillar-associated protein 3-like
219	Mical2	ENSMUST00000050149.6	microtubule associated monooxygenase, calponin and LIM domain containing 2
220	Mier3	ENSMUST00000109272.3	mesoderm induction early response 1, family member 3
221	Mkl2	ENSMUST00000149359.1	MKL/myocardin-like 2
222	Mllt3	ENSMUST00000078090.6	myeloid/lymphoid or mixed-lineage leukemia (trithorax homolog, Drosophila); translocated to, 3
223	Mmp16	ENSMUST00000029881.4	matrix metallopeptidase 16
224	Mpp3	ENSMUST00000100400.3	membrane protein, palmitoylated 3 (MAGUK p55 subfamily member 3)
225	Mtf2	ENSMUST00000081567.5	metal response element binding transcription factor 2
226	Mum1l1	ENSMUST00000113045.3	melanoma associated antigen (mutated) 1-like 1
227	Mycbp	ENSMUST00000030400.8	c-myc binding protein
228	Mypn	ENSMUST00000095580.2	myopalladin
229	Myrf	ENSMUST00000088013.6	myelin regulatory factor
230	Nabp1	ENSMUST00000027279.6	nucleic acid binding protein 1
231	Nap1l1	ENSMUST00000171797.1	nucleosome assembly protein 1-like 1
232	Ncoa3	ENSMUST00000088095.5	nuclear receptor coactivator 3
233	Ndrg1	ENSMUST00000005256.7	N-myc downstream regulated gene 1

234	Nelfa	ENSMUST00000030993.6	negative elongation factor complex member A, Whsc2
235	Nell1	ENSMUST00000081872.6	NEL-like 1
236	Nfia	ENSMUST00000092532.7	nuclear factor I/A
237	Nhlrc2	ENSMUST00000071423.5	NHL repeat containing 2
238	Nop9	ENSMUST00000019441.8	NOP9 nucleolar protein
239	Nox4	ENSMUST00000032781.8	NADPH oxidase 4
240	Nrp1	ENSMUST00000026917.8	neuropilin 1
241	Ntng1	ENSMUST00000156177.3	netrin G1
242	Ntrk3	ENSMUST00000039431.8	neurotrophic tyrosine kinase, receptor, type 3
243	Nxpe3	ENSMUST00000099705.3	neurexophilin and PC-esterase domain family, member 3
244	Nyap2	ENSMUST00000123285.1	neuronal tyrosine-phophorylated phosphoinositide 3-kinase adaptor 2
245	Onecut1	ENSMUST00000056006.10	one cut domain, family member 1
246	Onecut2	ENSMUST00000175965.3	one cut domain, family member 2
247	Osbpl3	ENSMUST00000114468.3	oxysterol binding protein-like 3
248	Otud4	ENSMUST00000173078.2	OTU domain containing 4
249	Otud7a	ENSMUST00000058476.8	OTU domain containing 7A
250	Otud7b	ENSMUST00000090785.3	OTU domain containing 7B
251	Oxsr1	ENSMUST00000040853.5	oxidative-stress responsive 1
252	P2ry4	ENSMUST00000053373.1	pyrimidinergic receptor P2Y, G-protein coupled, 4
253	P4ha2	ENSMUST00000174616.2	procollagen-proline, 2-oxoglutarate 4-dioxygenase (proline 4-hydroxylase), alpha II polypeptide
254	Pabpc4l	ENSMUST00000166505.1	poly(A) binding protein, cytoplasmic 4-like
255	Pak2	ENSMUST00000023467.8	p21 protein (Cdc42/Rac)-activated kinase 2
256	Pak3	ENSMUST00000172330.2	p21 protein (Cdc42/Rac)-activated kinase 3
257	Pcdh1	ENSMUST00000160721.2	protocadherin 1
258	Pcgf5	ENSMUST00000071267.7	polycomb group ring finger 5
259	Pcm1	ENSMUST00000045218.7	pericentriolar material 1
260	Pddc1	ENSMUST00000106008.1	Parkinson disease 7 domain containing 1
261	Pdk4	ENSMUST00000019721.4	pyruvate dehydrogenase kinase, isoenzyme 4
262	Pex5l	ENSMUST00000108225.4	peroxisomal biogenesis factor 5-like
263	Pgap1	ENSMUST00000097739.4	post-GPI attachment to proteins 1
264	Phactr1	ENSMUST00000148891.2	phosphatase and actin regulator 1
265	Phf20l1	ENSMUST00000048188.8	PHD finger protein 20-like 1
266	Phip	ENSMUST00000034787.6	pleckstrin homology domain interacting protein
267	Phtf2	ENSMUST00000118174.2	putative homeodomain transcription factor 2
268	Pik3c2a	ENSMUST00000170430.1	phosphatidylinositol 3-kinase, C2 domain containing, alpha polypeptide
269	Pik3r3	ENSMUST00000030464.8	phosphatidylinositol 3 kinase, regulatory subunit, polypeptide 3 (p55)
270	Pip4k2b	ENSMUST00000018691.8	phosphatidylinositol-5-phosphate 4-kinase, type II, beta
271	Pitpnc1	ENSMUST00000103064.4	phosphatidylinositol transfer protein, cytoplasmic 1
272	Plbd2	ENSMUST00000031597.6	phospholipase B domain containing 2
273	Plekha1	ENSMUST00000120441.2	pleckstrin homology domain containing, family A (phosphoinositide binding specific) member 1
274	Plxna4	ENSMUST00000115096.3	plexin A4
275	Polr3g	ENSMUST00000048993.6	polymerase (RNA) III (DNA directed) polypeptide G
276	POU2F1	ENSMUST00000111427.3	POU domain, class 2, transcription factor 1 isoform B
277	Pou2f1	ENSMUST00000111429.5	POU domain, class 2, transcription factor 1
278	Pou2f2	ENSMUST00000108418.5	POU domain, class 2, transcription factor 2
279	Pou3f2	ENSMUST00000178174.2	POU domain, class 3, transcription factor 2
280	Ppip5k2	ENSMUST00000042509.7	diphosphoinositol pentakisphosphate kinase 2

281	Ppm1a	ENSMUST00000021514.8	protein phosphatase 1A, magnesium dependent, alpha isoform
282	Ppp2r2a	ENSMUST00000089230.5	protein phosphatase 2, regulatory subunit B, alpha
283	Ppp2r5d	ENSMUST00000002839.8	protein phosphatase 2, regulatory subunit B', delta
284	Prdm1	ENSMUST00000039174.5	PR domain containing 1, with ZNF domain
285	Prkca	ENSMUST00000059595.5	protein kinase C, alpha
286	Prrx1	ENSMUST00000075805.7	paired related homeobox 1
287	Prtg	ENSMUST00000055535.8	protogenin homolog (Gallus gallus)
288	Psd3	ENSMUST00000038959.10	pleckstrin and Sec7 domain containing 3
289	Psen1	ENSMUST00000101225.1	presenilin 1
290	Ptbp2	ENSMUST00000029780.7	polypyrimidine tract binding protein 2
291	Ptchd1	ENSMUST00000038665.5	patched domain containing 1
292	Ptchd4	ENSMUST00000048691.4	patched domain containing 4
293	Pxdn	ENSMUST00000122328.2	peroxidasin homolog (Drosophila)
294	Rab3il1	ENSMUST00000121418.2	RAB3A interacting protein (rabin3)-like 1
295	Rap2a	ENSMUST00000062117.7	RAS related protein 2a
296	Rasa2	ENSMUST00000034984.7	RAS p21 protein activator 2
297	Rasgrf2	ENSMUST00000099326.4	RAS protein-specific guanine nucleotide-releasing factor 2
298	Rassf3	ENSMUST00000026902.7	Ras association (RalGDS/AF-6) domain family member 3
299	Rbm24	ENSMUST00000037923.3	RNA binding motif protein 24
300	Rbms3	ENSMUST00000111773.4	RNA binding motif, single stranded interacting protein
301	Rc3h1	ENSMUST00000161609.2	RING CCCH (C3H) domains 1
302	Rcor1	ENSMUST00000084968.8	REST corepressor 1
303	Rell1	ENSMUST00000154169.1	RELT-like 1
304	Rest	ENSMUST00000080359.6	RE1-silencing transcription factor
305	Rfx3	ENSMUST00000165566.2	regulatory factor X, 3 (influences HLA class II expression)
306	Rfx5	ENSMUST00000107254.2	regulatory factor X, 5 (influences HLA class II expression)
307	Rnf11	ENSMUST00000030284.4	ring finger protein 11
308	Rnf128	ENSMUST00000113026.1	ring finger protein 128
309	Rnf150	ENSMUST00000078525.5	ring finger protein 150
310	Rnf169	ENSMUST00000080817.4	ring finger protein 169
311	Rnf19a	ENSMUST00000022890.8	ring finger protein 19A
312	Rnf24	ENSMUST00000059372.5	ring finger protein 24
313	Rora	ENSMUST00000034766.8	RAR-related orphan receptor alpha
314	Rsc1a1	ENSMUST00000105782.1	regulatory solute carrier protein, family 1, member 1
315	Rtn4rl1	ENSMUST00000102514.3	reticulon 4 receptor-like 1
316	Rundc3b	ENSMUST00000047485.9	RUN domain containing 3B
317	Runx1t1	ENSMUST00000006761.4	runt-related transcription factor 1; translocated to, 1 (cyclin D-related)
318	Rybp	ENSMUST00000101118.2	RING1 and YY1 binding protein
319	Sall4	ENSMUST00000075044.4	sal-like 4 (Drosophila)
320	Scn2b	ENSMUST00000170998.2	sodium channel, voltage-gated, type II, beta
321	Scrib	ENSMUST00000002603.6	scribbled homolog (Drosophila)
322	Scube1	ENSMUST00000171496.2	signal peptide, CUB domain, EGF-like 1
323	Scyl3	ENSMUST00000027876.5	SCY1-like 3 (S. cerevisiae)
324	Sdc1	ENSMUST00000020911.8	syndecan 1
325	Sema3a	ENSMUST00000030714.7	sema domain, immunoglobulin domain (Ig), short basic domain, secreted, (semaphorin) 3A
326	Senp6	ENSMUST00000037484.9	SUMO/sentrin specific peptidase 6
327	Serinc5	ENSMUST00000049488.7	serine incorporator 5
328	Sgcd	ENSMUST00000077221.5	sarcoglycan, delta (dystrophin-associated glycoprotein)
329	Sh2b3	ENSMUST00000086310.2	SH2B adaptor protein 3

330	Sh3bgrl2	ENSMUST00000113215.4	SH3 domain binding glutamic acid-rich protein like 2
331	Sh3glb1	ENSMUST00000163279.1	SH3-domain GRB2-like B1 (endophilin)
332	Shc3	ENSMUST00000021898.5	src homology 2 domain-containing transforming protein C3
333	Shroom4	ENSMUST00000089520.2	shroom family member 4
334	Sin3a	ENSMUST00000167715.2	transcriptional regulator, SIN3A (yeast)
335	Six4	ENSMUST00000043208.7	sine oculis-related homeobox 4
336	Slain2	ENSMUST00000144843.2	SLAIN motif family, member 2
337	Slc12a5	ENSMUST00000099092.4	solute carrier family 12, member 5
338	Slc1a1	ENSMUST00000025875.4	solute carrier family 1 (neuronal/epithelial high affinity glutamate transporter, system Xag), member 1
339	Slc22a23	ENSMUST00000040336.6	solute carrier family 22, member 23
340	Slc25a35	ENSMUST00000018884.5	solute carrier family 25, member 35
341	Slc25a36	ENSMUST00000085206.5	solute carrier family 25, member 36
342	Slc30a5	ENSMUST00000067246.4	solute carrier family 30 (zinc transporter), member 5
343	Slc30a7	ENSMUST00000067485.3	solute carrier family 30 (zinc transporter), member 7
344	Slc44a1	ENSMUST00000107651.3	solute carrier family 44, member 1
345	Slc5a3	ENSMUST00000113975.2	solute carrier family 5 (inositol transporters), member 3
346	Slc6a2	ENSMUST00000072939.6	solute carrier family 6 (neurotransmitter transporter, noradrenalin), member 2
347	Slc6a20b	ENSMUST00000026273.8	solute carrier family 6 (neurotransmitter transporter), member 20B
348	Slc6a6	ENSMUST00000032185.7	solute carrier family 6 (neurotransmitter transporter, taurine), member 6
349	Slc7a11	ENSMUST00000029297.4	solute carrier family 7 (cationic amino acid transporter, y+ system), member 11
350	Slc8a1	ENSMUST00000163680.3	solute carrier family 8 (sodium/calcium exchanger), member 1
351	Slc9a7	ENSMUST00000072451.5	solute carrier family 9 (sodium/hydrogen exchanger), member 7
352	Slitrk2	ENSMUST00000166241.1	SLIT and NTRK-like family, member 2
353	Slitrk3	ENSMUST00000059407.7	SLIT and NTRK-like family, member 3
354	Slitrk4	ENSMUST00000069926.8	SLIT and NTRK-like family, member 4
355	Smarcd2	ENSMUST00000106843.2	SWI/SNF related, matrix associated, actin dependent regulator of chromatin, subfamily d, member 2
356	Smarce1	ENSMUST00000103133.3	SWI/SNF related, matrix associated, actin dependent regulator of chromatin, subfamily e, member 1
357	Smim20	ENSMUST00000121042.2	small integral membrane protein 20
358	Smurf2	ENSMUST00000103067.4	SMAD specific E3 ubiquitin protein ligase 2
359	Snrk	ENSMUST00000120173.2	SNF related kinase
360	Soat1	ENSMUST00000189661.1	sterol O-acyltransferase 1
361	Sord	ENSMUST00000110551.3	sorbitol dehydrogenase
362	Sp4	ENSMUST00000026367.9	trans-acting transcription factor 4
363	Spag9	ENSMUST00000041956.8	sperm associated antigen 9
364	Specc1l	ENSMUST00000105421.3	sperm antigen with calponin homology and coiled-coil domains 1-like
365	Spock1	ENSMUST00000185502.1	sparc/osteonectin, cwcv and kazal-like domains proteoglycan 1
366	Sptlc1	ENSMUST00000021920.6	serine palmitoyltransferase, long chain base subunit 1
367	Sptssb	ENSMUST00000171529.2	serine palmitoyltransferase, small subunit B
368	Srgn	ENSMUST00000160987.2	serglycin
369	Srpk1	ENSMUST00000130643.2	serine/arginine-rich protein specific kinase 1
370	Srsf1	ENSMUST00000079866.5	serine/arginine-rich splicing factor 1

371	Srsf6	ENSMUST00000130411.1	serine/arginine-rich splicing factor 6
372	Ssx2ip	ENSMUST00000106153.3	synovial sarcoma, X breakpoint 2 interacting protein
373	St8sia4	ENSMUST00000043336.5	ST8 alpha-N-acetyl-neuraminide alpha-2,8-sialyltransferase 4
374	Synj1	ENSMUST00000170853.2	synaptojanin 1
375	Syt9	ENSMUST00000073459.6	synaptotagmin IX
376	Taf1d	ENSMUST00000164079.2	TATA box binding protein (Tbp)-associated factor, RNA polymerase I, D
377	Taf4b	ENSMUST00000169862.1	TAF4B RNA polymerase II, TATA box binding protein (TBP)-associated factor
378	Tbpl1	ENSMUST00000127698.2	TATA box binding protein-like 1
379	Tenm1	ENSMUST00000115059.2	teneurin transmembrane protein 1
380	Tgfbr1	ENSMUST00000007757.9	transforming growth factor, beta receptor I
381	Tgfbr2	ENSMUST00000061101.5	transforming growth factor, beta receptor II
382	Thbs2	ENSMUST00000170872.1	thrombospondin 2
383	Timm23	ENSMUST00000170331.2	translocase of inner mitochondrial membrane 23
384	Tlk1	ENSMUST00000038584.8	tousled-like kinase 1
385	Tmem170b	ENSMUST00000129449.1	transmembrane protein 170B
386	Tmem212	ENSMUST00000058077.3	transmembrane protein 212
387	Tmem33	ENSMUST00000037918.6	transmembrane protein 33
388	Tnrc6b	ENSMUST00000067689.7	trinucleotide repeat containing 6b
389	Traf4	ENSMUST00000017530.3	TNF receptor associated factor 4
390	Trim2	ENSMUST00000107692.2	tripartite motif-containing 2
391	Trim66	ENSMUST00000106739.2	tripartite motif-containing 66
392	Trim71	ENSMUST00000111816.2	tripartite motif-containing 71
393	Tsc22d2	ENSMUST00000099090.2	TSC22 domain family, member 2
394	Tspan15	ENSMUST00000047883.9	tetraspanin 15
395	Tspan9	ENSMUST00000112173.2	tetraspanin 9
396	Twsg1	ENSMUST00000024906.4	twisted gastrulation homolog 1 (Drosophila)
397	Ube2h	ENSMUST00000102993.4	ubiquitin-conjugating enzyme E2H
398	Ubr5	ENSMUST00000110336.2	ubiquitin protein ligase E3 component n-recognin 5
399	Ubxn7	ENSMUST00000115151.3	UBX domain protein 7
400	Uhmk1	ENSMUST00000123399.1	U2AF homology motif (UHM) kinase 1
401	Unc13a	ENSMUST00000030170.9	unc-13 homolog A (C. elegans)
402	Unc80	ENSMUST00000061620.10	unc-80 homolog (C. elegans)
403	Usp15	ENSMUST00000020334.7	ubiquitin specific peptidase 15
404	Usp31	ENSMUST00000046929.6	ubiquitin specific peptidase 31
405	Vav3	ENSMUST00000046864.8	vav 3 oncogene
406	Vcl	ENSMUST00000022369.7	vinculin
407	Wasf2	ENSMUST00000084241.6	WAS protein family, member 2
408	Wdtc1	ENSMUST00000105906.1	WD and tetratricopeptide repeats 1
409	Wwp2	ENSMUST00000166615.1	WW domain containing E3 ubiquitin protein ligase 2
410	Xpo4	ENSMUST00000174545.2	exportin 4
411	Xrcc2	ENSMUST00000030773.7	X-ray repair complementing defective repair in Chinese hamster cells 2
412	Xylt1	ENSMUST00000032892.5	xylosyltransferase 1
413	Yap1	ENSMUST00000086580.6	yes-associated protein 1
414	Ypel2	ENSMUST00000018571.4	yippee-like 2 (Drosophila)
415	Zbtb1	ENSMUST00000042779.3	zinc finger and BTB domain containing 1
416	Zbtb20	ENSMUST00000114694.3	zinc finger and BTB domain containing 20
417	Zbtb26	ENSMUST00000067043.4	zinc finger and BTB domain containing 26
418	Zbtb41	ENSMUST00000039867.7	zinc finger and BTB domain containing 41 homolog

419	Zbtb44	ENSMUST00000115222.3	zinc finger and BTB domain containing 44
420	Zbtb7a	ENSMUST00000048128.9	zinc finger and BTB domain containing 7a
421	Zdbf2	ENSMUST00000114132.2	zinc finger, DBF-type containing 2
422	Zdhhc20	ENSMUST00000089473.3	zinc finger, DHHC domain containing 20
423	Zdhhc21	ENSMUST00000030110.9	zinc finger, DHHC domain containing 21
424	Zdhhc23	ENSMUST00000036321.8	zinc finger, DHHC domain containing 23
425	Zdhhc7	ENSMUST00000034280.7	zinc finger, DHHC domain containing 7
426	Zfhx3	ENSMUST00000043896.9	zinc finger homeobox 3
427	Zfhx4	ENSMUST00000176383.2	zinc finger homeodomain 4
428	Zfp266	ENSMUST00000174462.2	zinc finger protein 266
429	Zfp365	ENSMUST00000064656.7	zinc finger protein 365
430	Zfp395	ENSMUST00000066994.6	zinc finger protein 395
431	Zfp618	ENSMUST00000107415.2	zinc finger protein 618
432	Zkscan1	ENSMUST00000019660.5	zinc finger with KRAB and SCAN domains 1
433	Znrf2	ENSMUST00000079869.7	zinc and ring finger 2
434	Zzz3	ENSMUST00000106100.3	zinc finger, ZZ domain containing 3
