# Supplementary material for: miR-9 utilizes precursor pathways in adaptation to alcohol in mouse striatal neurons
Source: Adv Drug Alcohol Res. Author manuscript; Available in PMC 2023 Dec 19. (PMC10730111; doi:10.3389/adar.2023.11323)
Supplement: Mead et al SM Table 1 [file NIHMS1906815-supplement-Mead_et_al_SM_Table_1.pdf]

Supplementary Materials (SM), Table 1
List of murine miR-9-5p predicted targets by TargetScan 7.1

#	Target gene	Representative transcript	Gene name
1	Onecut2	ENSMUST00000175965.3	one cut domain, family member 2
2	Erg	ENSMUST00000113846.4	avian erythroblastosis virus E-26 (v-ets) oncogene related
3	Onecut1	ENSMUST00000056006.10	one cut domain, family member 1
4	Gm6970	ENSMUST00000169692.1	predicted gene 6970
5	Ybx3	ENSMUST00000032309.7	Y box protein 3
6	Slc39a1	ENSMUST00000015467.8	solute carrier family 39 (zinc transporter), member 1
7	Sord	ENSMUST00000110551.3	sorbitol dehydrogenase
8	Onecut3	ENSMUST00000051773.8	one cut domain, family member 3
9	Lifr	ENSMUST00000171588.1	leukemia inhibitory factor receptor
10	Ift52	ENSMUST00000018002.7	intraflagellar transport 52
11	Csnk1a1	ENSMUST00000165123.2	casein kinase 1, alpha 1
12	Tgfbi	ENSMUST00000045173.8	transforming growth factor, beta induced
13	Foxg1	ENSMUST00000179669.1	forkhead box G1
14	Traf3	ENSMUST00000117269.2	TNF receptor-associated factor 3
15	Cnnm1	ENSMUST00000165311.1	cyclin M1
16	Slc19a2	ENSMUST00000044021.6	solute carrier family 19 (thiamine transporter), member 2
17	Ccnt1	ENSMUST00000169707.2	cyclin T1
18	Slc5a3	ENSMUST00000113975.2	solute carrier family 5 (inositol transporters), member 3
19	Prdm6	ENSMUST00000115399.2	PR domain containing 6
20	Rims2	ENSMUST00000082054.6	regulating synaptic membrane exocytosis 2
21	Zfp266	ENSMUST00000174462.2	zinc finger protein 266
22	Slc20a2	ENSMUST00000067786.7	solute carrier family 20, member 2
23	Pak4	ENSMUST00000108283.2	p21 protein (Cdc42/Rac)-activated kinase 4
24	Tubgcp4	ENSMUST00000110658.2	tubulin, gamma complex associated protein 4
25	Slc2a2	ENSMUST00000029240.8	solute carrier family 2 (facilitated glucose transporter), member 2
26	Trpm7	ENSMUST00000103224.4	transient receptor potential cation channel, subfamily M, member 7
27	Rab34	ENSMUST00000002128.8	RAB34, member RAS oncogene family
28	P4ha2	ENSMUST00000174616.2	procollagen-proline, 2-oxoglutarate 4-dioxygenase (proline 4-hydroxylase), alpha II polypeptide
29	Kcnmb2	ENSMUST00000119970.2	potassium large conductance calcium-activated channel, subfamily M, beta member 2
30	Bend3	ENSMUST00000167488.2	BEN domain containing 3
31	Evi5l	ENSMUST00000176072.2	ecotropic viral integration site 5 like
32	Fry	ENSMUST00000087204.5	furry homolog (Drosophila)
33	Map7	ENSMUST00000116259.3	microtubule-associated protein 7
34	Enpep	ENSMUST00000029658.8	glutamyl aminopeptidase
35	Ldlrap1	ENSMUST00000037828.6	low density lipoprotein receptor adaptor protein 1
36	Slc27a4	ENSMUST00000080065.2	solute carrier family 27 (fatty acid transporter), member 4
37	Rgs8	ENSMUST00000041776.6	regulator of G-protein signaling 8
38	1110007C09Rik	ENSMUST00000048946.6	RIKEN cDNA 1110007C09 gene
39	Kcnj2	ENSMUST00000042970.2	potassium inwardly-rectifying channel, subfamily J, member 2
40	Kcna2	ENSMUST00000038695.1	potassium voltage-gated channel, shaker-related subfamily, member 2
41	Lin28b	ENSMUST00000079390.6	lin-28 homolog B (C. elegans)
42	Mesdc1	ENSMUST00000094216.4	mesoderm development candidate 1
43	POU2F1	ENSMUST00000111427.3	POU domain, class 2, transcription factor 1 isoform B
44	Prtg	ENSMUST00000055535.8	protogenin homolog (Gallus gallus)
45	Ap1s2	ENSMUST00000069041.9	adaptor-related protein complex 1, sigma 2 subunit
46	Syap1	ENSMUST00000033723.3	synapse associated protein 1
47	Mga	ENSMUST00000079934.6	MAX gene associated
48	Etl4	ENSMUST00000114606.2	enhancer trap locus 4
49	Utrn	ENSMUST00000076817.4	utrophin
50	Foxi3	ENSMUST00000069634.5	forkhead box I3

51	Calb2	ENSMUST00000003754.6	calbindin 2
52	Stk3	ENSMUST00000018476.8	serine/threonine kinase 3
53	Tesk2	ENSMUST00000045542.7	testis-specific kinase 2
54	Slc31a2	ENSMUST00000084530.3	solute carrier family 31, member 2
55	Snrk	ENSMUST00000120173.2	SNF related kinase
56	Maea	ENSMUST00000114449.3	macrophage erythroblast attacher
57	Snx25	ENSMUST00000110378.3	sorting nexin 25
58	Sfxn2	ENSMUST00000026011.6	sideroflexin 2
59	Ipo4	ENSMUST00000047131.10	importin 4
60	Mdga1	ENSMUST00000171691.2	MAM domain containing glycosylphosphatidylinositol anchor 1
61	Adam11	ENSMUST00000068150.5	a disintegrin and metallopeptidase domain 11
62	Ano1	ENSMUST00000121758.2	anoctamin 1, calcium activated chloride channel
63	Greb1	ENSMUST00000162112.2	gene regulated by estrogen in breast cancer protein
64	Gna14	ENSMUST00000025602.2	guanine nucleotide binding protein, alpha 14
65	Zfp236	ENSMUST00000171071.3	zinc finger protein 236
66	Lect1	ENSMUST00000165835.2	leukocyte cell derived chemotaxin 1
67	Ehd4	ENSMUST00000028755.7	EH-domain containing 4
68	Lepre1	ENSMUST00000030393.7	leprecan 1
69	Arid3c	ENSMUST00000171251.2	AT rich interactive domain 3C (BRIGHT-like)
70	Znrf2	ENSMUST00000079869.7	zinc and ring finger 2
71	Prrx1	ENSMUST00000075805.7	paired related homeobox 1
72	Napb	ENSMUST00000028926.7	N-ethylmaleimide sensitive fusion protein attachment protein beta
73	Cthrc1	ENSMUST00000067072.3	collagen triple helix repeat containing 1
74	Cdc73	ENSMUST00000018337.8	cell division cycle 73, Paf1/RNA polymerase II complex component
75	Cxcl11	ENSMUST00000077820.5	chemokine (C-X-C motif) ligand 11
76	Slc50a1	ENSMUST00000107460.2	solute carrier family 50 (sugar transporter), member 1
77	Cxcl2	ENSMUST00000075433.6	chemokine (C-X-C motif) ligand 2
78	Tbpl1	ENSMUST00000127698.2	TATA box binding protein-like 1
79	Scrib	ENSMUST00000002603.6	scribbled homolog (Drosophila)
80	Fam19a5	ENSMUST00000068088.6	family with sequence similarity 19, member A5
81	Ap3b1	ENSMUST00000022196.3	adaptor-related protein complex 3, beta 1 subunit
82	Frmd6	ENSMUST00000057859.7	FERM domain containing 6
83	Zbtb20	ENSMUST00000114694.3	zinc finger and BTB domain containing 20
84	Slc10a3	ENSMUST00000114147.1	solute carrier family 10 (sodium/bile acid cotransporter family), member 3
85	Galnt3	ENSMUST00000028378.3	UDP-N-acetyl-alpha-D-galactosamine:polypeptide N-acetylgalactosaminyltransferase 3
86	Vav3	ENSMUST00000046864.8	vav 3 oncogene
87	Spg20	ENSMUST00000044116.8	spastic paraplegia 20, spartin (Troyer syndrome) homolog (human)
88	Ube3c	ENSMUST00000049453.4	ubiquitin protein ligase E3C
89	Csgalnact1	ENSMUST00000078350.7	chondroitin sulfate N-acetylgalactosaminyltransferase 1
90	Cdc14b	ENSMUST00000109770.1	CDC14 cell division cycle 14B
91	Magt1	ENSMUST00000151689.2	magnesium transporter 1
92	Ccdc43	ENSMUST00000092569.7	coiled-coil domain containing 43
93	Col18a1	ENSMUST00000081654.7	collagen, type XVIII, alpha 1
94	Nxpe3	ENSMUST00000099705.3	neurexophilin and PC-esterase domain family, member 3
95	Timm23	ENSMUST00000170331.2	translocase of inner mitochondrial membrane 23
96	Pigz	ENSMUST00000052174.2	phosphatidylinositol glycan anchor biosynthesis, class Z
97	Vdac3	ENSMUST00000009036.4	voltage-dependent anion channel 3
98	Atl2	ENSMUST00000112437.2	atlastin GTPase 2
99	Shroom4	ENSMUST00000089520.2	shroom family member 4
100	AI118078	ENSMUST00000085754.4	expressed sequence AI118078
101	Ap4e1	ENSMUST00000002063.9	adaptor-related protein complex AP-4, epsilon 1
102	Kcnk4	ENSMUST00000025908.6	potassium channel, subfamily K, member 4
103	Acot7	ENSMUST00000167926.2	acyl-CoA thioesterase 7
104	Foxp2	ENSMUST00000115477.2	forkhead box P2

105	Emb	ENSMUST00000022242.7	embigin
106	Pou2f3	ENSMUST00000034513.7	POU domain, class 2, transcription factor 3
107	Smarcd2	ENSMUST00000106843.2	SWI/SNF related, matrix associated, actin dependent regulator of chromatin, subfamily d, member 2
108	Wasf2	ENSMUST00000084241.6	WAS protein family, member 2
109	Col15a1	ENSMUST00000102917.5	collagen, type XV, alpha 1
110	Foxn2	ENSMUST00000112238.3	forkhead box N2
111	Nr5a2	ENSMUST00000027649.8	nuclear receptor subfamily 5, group A, member 2
112	Grhl1	ENSMUST00000020985.8	grainyhead-like 1 (Drosophila)
113	Rnf150	ENSMUST00000078525.5	ring finger protein 150
114	Tnc	ENSMUST00000030056.6	tenascin C
115	Exd2	ENSMUST00000038185.8	exonuclease 3'-5' domain containing 2
116	Tmem109	ENSMUST00000038128.9	transmembrane protein 109
117	Sptssa	ENSMUST00000056228.6	serine palmitoyltransferase, small subunit A
118	BC007180	ENSMUST00000189969.1	cDNA sequence BC007180
119	Dnajc14	ENSMUST00000026410.1	DnaJ (Hsp40) homolog, subfamily C, member 14
120	Snx7	ENSMUST00000029639.6	sorting nexin 7
121	Pcgf6	ENSMUST00000026032.5	polycomb group ring finger 6
122	Nhsl1	ENSMUST00000037341.8	NHS-like 1
123	Celf6	ENSMUST00000118549.2	CUGBP, Elav-like family member 6
124	Armcx2	ENSMUST00000119010.2	armadillo repeat containing, X-linked 2
125	Klf5	ENSMUST00000005279.6	Kruppel-like factor 5
126	Zbtb14	ENSMUST00000112674.2	zinc finger and BTB domain containing 14
127	Twsg1	ENSMUST00000024906.4	twisted gastrulation homolog 1 (Drosophila)
128	Dse	ENSMUST00000048010.7	dermatan sulfate epimerase
129	Snx16	ENSMUST00000099223.5	sorting nexin 16
130	Mum1l1	ENSMUST00000113045.3	melanoma associated antigen (mutated) 1-like 1
131	Rell1	ENSMUST00000154169.1	RELT-like 1
132	Nox4	ENSMUST00000032781.8	NADPH oxidase 4
133	Hltf	ENSMUST00000002502.6	helicase-like transcription factor
134	Fam107b	ENSMUST00000027965.5	family with sequence similarity 107, member B
135	St6gal1	ENSMUST00000178797.2	beta galactoside alpha 2,6 sialyltransferase 1
136	Itm2c	ENSMUST00000027425.10	integral membrane protein 2C
137	Zc3h12a	ENSMUST00000036188.7	zinc finger CCCH type containing 12A
138	Capza1	ENSMUST00000094028.5	capping protein (actin filament) muscle Z-line, alpha 1
139	Myh1	ENSMUST00000124516.2	myosin, heavy polypeptide 1, skeletal muscle, adult
140	Hmga2	ENSMUST00000159699.1	high mobility group AT-hook 2
141	Pbrm1	ENSMUST00000112095.2	polybromo 1
142	Socs5	ENSMUST00000041369.6	suppressor of cytokine signaling 5
143	Bcat2	ENSMUST00000033098.8	branched chain aminotransferase 2, mitochondrial
144	Igf2bp3	ENSMUST00000031838.7	insulin-like growth factor 2 mRNA binding protein 3
145	Hyal4	ENSMUST00000031691.2	hyaluronoglucosaminidase 4
146	Slc17a2	ENSMUST00000006786.5	solute carrier family 17 (sodium phosphate), member 2
147	Dram2	ENSMUST00000067630.7	DNA-damage regulated autophagy modulator 2
148	Ank2	ENSMUST00000182704.2	ankyrin 2, brain
149	Phf13	ENSMUST00000055688.9	PHD finger protein 13
150	Ankrd13a	ENSMUST00000102578.5	ankyrin repeat domain 13a
151	Zfp324	ENSMUST00000038701.7	zinc finger protein 324
152	Fgf18	ENSMUST00000109363.2	fibroblast growth factor 18
153	Mtmr2	ENSMUST00000034396.8	myotubularin related protein 2
154	Atp1b1	ENSMUST00000027863.7	ATPase, Na+/K+ transporting, beta 1 polypeptide
155	Klhl18	ENSMUST00000111933.2	kelch-like 18
156	Tcf7	ENSMUST00000086844.4	transcription factor 7, T cell specific
157	Phldb2	ENSMUST00000036355.7	pleckstrin homology-like domain, family B, member 2
158	Cass4	ENSMUST00000103073.3	Cas scaffolding protein family member 4
159	Ces1c	ENSMUST00000034189.9	carboxylesterase 1C

160	Eif4e3	ENSMUST00000032151.2	eukaryotic translation initiation factor 4E member 3
161	Ddhd2	ENSMUST00000033975.6	DDHD domain containing 2
162	Sirt1	ENSMUST00000120239.2	sirtuin 1
163	Cngb3	ENSMUST00000102999.1	cyclic nucleotide gated channel beta 3
164	Plscr3	ENSMUST00000108633.3	phospholipid scramblase 3
165	Cpeb2	ENSMUST00000169035.2	cytoplasmic polyadenylation element binding protein 2
166	Hist1h4h	ENSMUST00000102972.4	histone cluster 1, H4h
167	Ctnna1	ENSMUST00000042345.6	catenin (cadherin associated protein), alpha 1
168	Smoc2	ENSMUST00000024660.8	SPARC related modular calcium binding 2
169	Tbc1d22a	ENSMUST00000063414.8	TBC1 domain family, member 22a
170	Atg14	ENSMUST00000042988.6	autophagy related 14
171	Arhgdia	ENSMUST00000106197.4	Rho GDP dissociation inhibitor (GDI) alpha
172	Ube2z	ENSMUST00000100528.4	ubiquitin-conjugating enzyme E2Z (putative)
173	Lurap1l	ENSMUST00000055922.3	leucine rich adaptor protein 1-like
174	Fbxl2	ENSMUST00000035090.8	F-box and leucine-rich repeat protein 2
175	Pim3	ENSMUST00000042818.9	proviral integration site 3
176	Il10rb	ENSMUST00000023691.6	interleukin 10 receptor, beta
177	Klhl1	ENSMUST00000022666.7	kelch-like 1
178	Prkg2	ENSMUST00000031277.6	protein kinase, cGMP-dependent, type II
179	Pdgfc	ENSMUST00000029652.3	platelet-derived growth factor, C polypeptide
180	Fstl1	ENSMUST00000114763.2	follistatin-like 1
181	Palmd	ENSMUST00000040097.8	palmdelphin
182	Foxp1	ENSMUST00000113326.3	forkhead box P1
183	Atp8b2	ENSMUST00000069805.8	ATPase, class I, type 8B, member 2
184	Gnpnat1	ENSMUST00000046191.7	glucosamine-phosphate N-acetyltransferase 1
185	Ldlrad3	ENSMUST00000058790.6	low density lipoprotein receptor class A domain containing 3
186	Map2k7	ENSMUST00000062686.5	mitogen-activated protein kinase kinase 7
187	Kdm5a	ENSMUST00000005108.7	lysine (K)-specific demethylase 5A
188	Fbn2	ENSMUST00000025497.6	fibrillin 2
189	Ift80	ENSMUST00000107812.2	intraflagellar transport 80
190	9130230L23Rik	ENSMUST00000067737.6	RIKEN cDNA 9130230L23 gene
191	Myo1c	ENSMUST00000102505.4	myosin IC
192	Pou2f1	ENSMUST00000111429.5	POU domain, class 2, transcription factor 1
193	Kctd12	ENSMUST00000184744.1	potassium channel tetramerisation domain containing 12
194	Fbxw2	ENSMUST00000028220.4	F-box and WD-40 domain protein 2
195	Pou2f2	ENSMUST00000108418.5	POU domain, class 2, transcription factor 2
196	Nell1	ENSMUST00000081872.6	NEL-like 1
197	Slc35b3	ENSMUST00000021870.5	solute carrier family 35, member B3
198	Akr1b3	ENSMUST00000102980.5	aldo-keto reductase family 1, member B3 (aldose reductase)
199	Auh	ENSMUST00000021913.10	AU RNA binding protein/enoyl-coenzyme A hydratase
200	Runx1t1	ENSMUST00000006761.4	runt-related transcription factor 1; translocated to, 1 (cyclin D-related)
201	Tal2	ENSMUST00000030124.3	T cell acute lymphocytic leukemia 2
202	Taf4b	ENSMUST00000169862.1	TAF4B RNA polymerase II, TATA box binding protein (TBP)-associated factor
203	Tmem260	ENSMUST00000111735.3	transmembrane protein 260
204	Esyt1	ENSMUST00000026427.6	extended synaptotagmin-like protein 1
205	Dlx3	ENSMUST00000092768.6	distal-less homeobox 3
206	Mthfd2	ENSMUST00000005810.6	methylenetetrahydrofolate dehydrogenase (NAD+ dependent), methenyltetrahydrofolate cyclohydrolase
207	Zbtb1	ENSMUST00000042779.3	zinc finger and BTB domain containing 1
208	Tsc22d2	ENSMUST00000099090.2	TSC22 domain family, member 2
209	Six5	ENSMUST00000049454.5	sine oculis-related homeobox 5
210	Map3k2	ENSMUST00000096575.3	mitogen-activated protein kinase kinase kinase 2
211	Vamp3	ENSMUST00000030797.3	vesicle-associated membrane protein 3
212	Cnnm2	ENSMUST00000099373.5	cyclin M2
213	Igf2bp2	ENSMUST00000100052.5	insulin-like growth factor 2 mRNA binding protein 2

214	St8sia4	ENSMUST00000043336.5	ST8 alpha-N-acetyl-neuraminide alpha-2,8-sialyltransferase 4
215	Cntfr	ENSMUST00000102961.4	ciliary neurotrophic factor receptor
216	Maf	ENSMUST00000109104.1	avian musculoaponeurotic fibrosarcoma (v-maf) AS42 oncogene homolog
217	Fam149b	ENSMUST00000090503.5	family with sequence similarity 149, member B
218	Inhbb	ENSMUST00000038765.5	inhibin beta-B
219	Furin	ENSMUST00000122232.2	furin (paired basic amino acid cleaving enzyme)
220	Zkscan1	ENSMUST00000019660.5	zinc finger with KRAB and SCAN domains 1
221	Atoh8	ENSMUST00000042646.7	atonal homolog 8 (Drosophila)
222	Cbfa2t3	ENSMUST00000127984.2	core-binding factor, runt domain, alpha subunit 2, translocated to, 3 (human)
223	Pcsk5	ENSMUST00000050715.8	proprotein convertase subtilisin/kexin type 5
224	Foxp4	ENSMUST00000113265.2	forkhead box P4
225	Agfg1	ENSMUST00000189220.1	ArfGAP with FG repeats 1
226	Rassf3	ENSMUST00000026902.7	Ras association (RalGDS/AF-6) domain family member 3
227	M6pr	ENSMUST00000007602.9	mannose-6-phosphate receptor, cation dependent
228	B3galnt2	ENSMUST00000099747.3	UDP-GalNAc:betaGlcNAc beta 1,3-galactosaminyltransferase, polypeptide 2
229	Arhgap24	ENSMUST00000073302.6	Rho GTPase activating protein 24
230	Kpnb1	ENSMUST00000001479.4	karyopherin (importin) beta 1
231	Nap1l1	ENSMUST00000171797.1	nucleosome assembly protein 1-like 1
232	Lamp1	ENSMUST00000033824.6	lysosomal-associated membrane protein 1
233	Col9a1	ENSMUST00000054588.9	collagen, type IX, alpha 1
234	Itga6	ENSMUST00000028522.4	integrin alpha 6
235	Ntng1	ENSMUST00000156177.3	netrin G1
236	Map3k3	ENSMUST00000002044.9	mitogen-activated protein kinase kinase kinase 3
237	Id4	ENSMUST00000021810.1	inhibitor of DNA binding 4
238	Ulk2	ENSMUST00000004920.3	unc-51 like kinase 2
239	Bclaf1	ENSMUST00000092678.4	BCL2-associated transcription factor 1
240	Icmt	ENSMUST00000048892.8	isoprenylcysteine carboxyl methyltransferase
241	Ccar2	ENSMUST00000035612.5	cell cycle activator and apoptosis regulator 2
242	Vgll4	ENSMUST00000032459.8	vestigial like 4 (Drosophila)
243	Reep4	ENSMUST00000047218.3	receptor accessory protein 4
244	Plbd2	ENSMUST00000031597.6	phospholipase B domain containing 2
245	Igfbp3	ENSMUST00000020702.5	insulin-like growth factor binding protein 3
246	Ncoa3	ENSMUST00000088095.5	nuclear receptor coactivator 3
247	Phf21a	ENSMUST00000044036.9	PHD finger protein 21A
248	Ccne2	ENSMUST00000029866.10	cyclin E2
249	Ube2q1	ENSMUST00000038356.8	ubiquitin-conjugating enzyme E2Q (putative) 1
250	Fbn1	ENSMUST00000028633.7	fibrillin 1
251	Dtd1	ENSMUST00000028917.6	D-tyrosyl-tRNA deacylase 1
252	Hormad1	ENSMUST00000090797.5	HORMA domain containing 1
253	Pdgfrb	ENSMUST00000025522.5	platelet derived growth factor receptor, beta polypeptide
254	Myocd	ENSMUST00000102635.4	myocardin
255	Pi4k2a	ENSMUST00000066778.4	phosphatidylinositol 4-kinase type 2 alpha
256	Cmtm6	ENSMUST00000035007.8	CKLF-like MARVEL transmembrane domain containing 6
257	Maml2	ENSMUST00000177755.1	mastermind like 2 (Drosophila)
258	Rbms3	ENSMUST00000111773.4	RNA binding motif, single stranded interacting protein
259	Ambra1	ENSMUST00000099712.4	autophagy/beclin 1 regulator 1
260	Cdc25a	ENSMUST00000094324.3	cell division cycle 25A
261	Lrrtm4	ENSMUST00000136421.1	leucine rich repeat transmembrane neuronal 4
262	Kcnj14	ENSMUST00000071937.5	potassium inwardly-rectifying channel, subfamily J, member 14
263	Diap2	ENSMUST00000113320.2	diaphanous homolog 2 (Drosophila)
264	Arpc1a	ENSMUST00000031625.9	actin related protein 2/3 complex, subunit 1A
265	Mtf2	ENSMUST00000081567.5	metal response element binding transcription factor 2
266	Syt9	ENSMUST00000073459.6	synaptotagmin IX

267	Hk2	ENSMUST00000000642.5	hexokinase 2
268	Slc25a24	ENSMUST00000029477.6	solute carrier family 25 (mitochondrial carrier, phosphate carrier), member 24
269	Arid3b	ENSMUST00000171444.2	AT rich interactive domain 3B (BRIGHT-like)
270	Gmeb2	ENSMUST00000049032.7	glucocorticoid modulatory element binding protein 2
271	Stmn1	ENSMUST00000030636.5	stathmin 1
272	Lzts2	ENSMUST00000039016.7	leucine zipper, putative tumor suppressor 2
273	Lrch4	ENSMUST00000031734.10	leucine-rich repeats and calponin homology (CH) domain containing 4
274	Zfp354a	ENSMUST00000109122.2	zinc finger protein 354A
275	Timm44	ENSMUST00000003029.8	translocase of inner mitochondrial membrane 44
276	Nrp1	ENSMUST00000026917.8	neuropilin 1
277	Colec12	ENSMUST00000040069.8	collectin sub-family member 12
278	Ccdc50	ENSMUST00000100026.4	coiled-coil domain containing 50
279	Gzf1	ENSMUST00000028928.7	GDNF-inducible zinc finger protein 1
280	Fbxl3	ENSMUST00000022720.9	F-box and leucine-rich repeat protein 3
281	Shc2	ENSMUST00000163867.2	SHC (Src homology 2 domain containing) transforming protein 2
282	Clock	ENSMUST00000075159.1	circadian locomotor output cycles kaput
283	Slain2	ENSMUST00000144843.2	SLAIN motif family, member 2
284	Lyve1	ENSMUST00000033050.3	lymphatic vessel endothelial hyaluronan receptor 1
285	Gdnf	ENSMUST00000022744.3	glial cell line derived neurotrophic factor
286	Gltscr1l	ENSMUST00000040624.5	GLTSCR1-like
287	Faf2	ENSMUST00000126071.2	Fas associated factor family member 2
288	Shc3	ENSMUST00000021898.5	src homology 2 domain-containing transforming protein C3
289	Anp32b	ENSMUST00000102926.4	acidic (leucine-rich) nuclear phosphoprotein 32 family, member B
290	Nek1	ENSMUST00000034065.8	NIMA (never in mitosis gene a)-related expressed kinase 1
291	Tmem136	ENSMUST00000061833.4	transmembrane protein 136
292	Ptchd4	ENSMUST00000048691.4	patched domain containing 4
293	Cntn3	ENSMUST00000032159.6	contactin 3
294	Gm9804	ENSMUST00000056234.3	predicted gene 9804
295	Dpysl5	ENSMUST00000114729.2	dihydropyrimidinase-like 5
296	Pxdn	ENSMUST00000122328.2	peroxidasin homolog (Drosophila)
297	Mdga2	ENSMUST00000113942.2	MAM domain containing glycosylphosphatidylinositol anchor 2
298	Rnf24	ENSMUST00000059372.5	ring finger protein 24
299	Ar	ENSMUST00000052837.7	androgen receptor
300	Rnf128	ENSMUST00000113026.1	ring finger protein 128
301	Cyb561	ENSMUST00000184086.2	cytochrome b-561
302	Lin28a	ENSMUST00000051674.2	lin-28 homolog A (C. elegans)
303	Nedd4	ENSMUST00000034740.9	neural precursor cell expressed, developmentally down-regulated 4
304	Slc9a1	ENSMUST00000030669.7	solute carrier family 9 (sodium/hydrogen exchanger), member 1
305	Pygo2	ENSMUST00000060061.5	pygopus 2
306	4930506M07Rik	ENSMUST00000163821.1	RIKEN cDNA 4930506M07 gene
307	Gpr123	ENSMUST00000026548.8	G protein-coupled receptor 123
308	Crebrf	ENSMUST00000062519.8	CREB3 regulatory factor
309	Zic5	ENSMUST00000039118.6	zinc finger protein of the cerebellum 5
310	Hes1	ENSMUST00000023171.7	hairy and enhancer of split 1 (Drosophila)
311	Thbs2	ENSMUST00000170872.1	thrombospondin 2
312	Dusp6	ENSMUST00000020118.4	dual specificity phosphatase 6
313	Jakmip2	ENSMUST00000082254.6	janus kinase and microtubule interacting protein 2
314	Man1a2	ENSMUST00000008907.8	mannosidase, alpha, class 1A, member 2
315	Shisa2	ENSMUST00000053949.5	shisa homolog 2 (Xenopus laevis)
316	Amigo2	ENSMUST00000053106.5	adhesion molecule with Ig like domain 2
317	C1ql1	ENSMUST00000057849.5	complement component 1, q subcomponent-like 1
318	Ppp6r3	ENSMUST00000113997.3	protein phosphatase 6, regulatory subunit 3
319	Zc3h10	ENSMUST00000040572.4	zinc finger CCCH type containing 10
320	Insig1	ENSMUST00000059155.10	insulin induced gene 1
321	Fgf12	ENSMUST00000100024.1	fibroblast growth factor 12

322	Sh2b3	ENSMUST00000086310.2	SH2B adaptor protein 3
323	Hic2	ENSMUST00000090190.6	hypermethylated in cancer 2
324	Fnbp4	ENSMUST00000013759.5	formin binding protein 4
325	Phf20l1	ENSMUST00000048188.8	PHD finger protein 20-like 1
326	Rnf144a	ENSMUST00000020971.7	ring finger protein 144A
327	Phip	ENSMUST00000034787.6	pleckstrin homology domain interacting protein
328	Cttnbp2nl	ENSMUST00000077548.6	CTTNBP2 N-terminal like
329	Crim1	ENSMUST00000112498.2	cysteine rich transmembrane BMP regulator 1 (chordin like)
330	Ypel2	ENSMUST00000018571.4	yippee-like 2 (Drosophila)
331	Trmt10b	ENSMUST00000044673.3	tRNA methyltransferase 10B
332	Grwd1	ENSMUST00000107723.3	glutamate-rich WD repeat containing 1
333	Rnf111	ENSMUST00000034739.6	ring finger 111
334	Gskip	ENSMUST00000051934.5	GSK3B interacting protein
335	Nhlh2	ENSMUST00000066187.4	nescient helix loop helix 2
336	Krt222	ENSMUST00000103132.4	keratin 222
337	Arx	ENSMUST00000113947.3	aristaless related homeobox
338	Samd11	ENSMUST00000179919.1	sterile alpha motif domain containing 11
339	Dyrk1b	ENSMUST00000085901.7	dual-specificity tyrosine-(Y)-phosphorylation regulated kinase 1b
340	Sacs	ENSMUST00000119943.2	sacsin
341	Plbd1	ENSMUST00000032336.4	phospholipase B domain containing 1
342	Grsf1	ENSMUST00000078945.6	G-rich RNA sequence binding factor 1
343	Slc18a2	ENSMUST00000026084.3	solute carrier family 18 (vesicular monoamine), member 2
344	Zfp131	ENSMUST00000177916.2	zinc finger protein 131
345	Fam13c	ENSMUST00000062883.6	family with sequence similarity 13, member C
346	8030462N17Rik	ENSMUST00000074653.4	RIKEN cDNA 8030462N17 gene
347	Mrfap1	ENSMUST00000068795.3	Morf4 family associated protein 1
348	Tgfbr2	ENSMUST00000061101.5	transforming growth factor, beta receptor II
349	Mypn	ENSMUST00000095580.2	myopalladin
350	Egln3	ENSMUST00000039516.3	egl-9 family hypoxia-inducible factor 3
351	Sort1	ENSMUST00000102632.5	sortilin 1
352	Fam46a	ENSMUST00000034802.9	family with sequence similarity 46, member A
353	Tbc1d8	ENSMUST00000054462.5	TBC1 domain family, member 8
354	Hoxa11	ENSMUST00000048026.9	homeobox A11
355	Rab3il1	ENSMUST00000121418.2	RAB3A interacting protein (rabin3)-like 1
356	Uhrf1	ENSMUST00000001258.9	ubiquitin-like, containing PHD and RING finger domains, 1
357	Dennd1a	ENSMUST00000102787.4	DENN/MADD domain containing 1A
358	Bcl6	ENSMUST00000023151.5	B cell leukemia/lymphoma 6
359	Atp11c	ENSMUST00000154051.2	ATPase, class VI, type 11C
360	2510039O18Rik	ENSMUST00000103232.1	RIKEN cDNA 2510039O18 gene
361	Aebp2	ENSMUST00000032359.9	AE binding protein 2
362	1200014J11Rik	ENSMUST00000021135.3	RIKEN cDNA 1200014J11 gene
363	Dedd	ENSMUST00000111300.2	death effector domain-containing
364	Fam46d	ENSMUST00000101292.3	family with sequence similarity 46, member D
365	Slc25a36	ENSMUST00000085206.5	solute carrier family 25, member 36
366	Arid1a	ENSMUST00000145664.3	AT rich interactive domain 1A (SWI-like)
367	Dupd1	ENSMUST00000073870.5	dual specificity phosphatase and pro isomerase domain containing 1
368	Arhgef17	ENSMUST00000107032.1	Rho guanine nucleotide exchange factor (GEF) 17
369	Adcy5	ENSMUST00000114913.1	adenylate cyclase 5
370	Kif24	ENSMUST00000108055.3	kinesin family member 24
371	Vangl1	ENSMUST00000029453.7	vang-like 1 (van gogh, Drosophila)
372	Ptbp3	ENSMUST00000030076.6	polypyrimidine tract binding protein 3
373	Otud7b	ENSMUST00000090785.3	OTU domain containing 7B
374	Rhoq	ENSMUST00000024956.9	ras homolog gene family, member Q
375	Slc38a4	ENSMUST00000023101.4	solute carrier family 38, member 4
376	Atf1	ENSMUST00000023769.5	activating transcription factor 1
377	Kcnk3	ENSMUST00000066295.2	potassium channel, subfamily K, member 3

378	Timm17a	ENSMUST00000081104.4	translocase of inner mitochondrial membrane 17a
379	Fam199x	ENSMUST00000047852.7	family with sequence similarity 199, X-linked
380	Cc2d1b	ENSMUST00000030320.7	coiled-coil and C2 domain containing 1B
381	1190002N15Rik	ENSMUST00000113028.1	RIKEN cDNA 1190002N15 gene
382	Npy1r	ENSMUST00000039303.5	neuropeptide Y receptor Y1
383	Rap1b	ENSMUST00000064667.7	RAS related protein 1b
384	Ppm1l	ENSMUST00000029355.7	protein phosphatase 1 (formerly 2C)-like
385	Gpr137c	ENSMUST00000146150.1	G protein-coupled receptor 137C
386	Ppapdc2	ENSMUST00000045674.3	phosphatidic acid phosphatase type 2 domain containing 2
387	Elavl1	ENSMUST00000098950.4	ELAV (embryonic lethal, abnormal vision)-like 1 (Hu antigen R)
388	Flrt3	ENSMUST00000110057.2	fibronectin leucine rich transmembrane protein 3
389	Ptchd1	ENSMUST00000038665.5	patched domain containing 1
390	Rnf19a	ENSMUST00000022890.8	ring finger protein 19A
391	Ammecr1	ENSMUST00000041317.2	Alport syndrome, mental retardation, midface hypoplasia and elliptocytosis chromosomal region gene 1
392	Tnfrsf21	ENSMUST00000024708.4	tumor necrosis factor receptor superfamily, member 21
393	Eif4e	ENSMUST00000029803.7	eukaryotic translation initiation factor 4E
394	Ube2h	ENSMUST00000102993.4	ubiquitin-conjugating enzyme E2H
395	Mpp3	ENSMUST00000100400.3	membrane protein, palmitoylated 3 (MAGUK p55 subfamily member 3)
396	Adamts5	ENSMUST00000023611.5	a disintegrin-like and metallopeptidase (reprolysin type) with thrombospondin type 1 motif, 5 (aggrecanase-2)
397	Itpkc	ENSMUST00000003850.7	inositol 1,4,5-trisphosphate 3-kinase C
398	Xpr1	ENSMUST00000027741.6	xenotropic and polytropic retrovirus receptor 1
399	Myh9	ENSMUST00000016771.7	myosin, heavy polypeptide 9, non-muscle
400	Arl4c	ENSMUST00000159814.1	ADP-ribosylation factor-like 4C
401	Arpp21	ENSMUST00000111872.3	cyclic AMP-regulated phosphoprotein, 21
402	En1	ENSMUST00000079721.8	engrailed 1
403	Bnc2	ENSMUST00000176612.2	basonuclin 2
404	Fam126b	ENSMUST00000161600.2	family with sequence similarity 126, member B
405	Fam46c	ENSMUST00000061455.8	family with sequence similarity 46, member C
406	Klf7	ENSMUST00000114086.2	Kruppel-like factor 7 (ubiquitous)
407	Ptbp1	ENSMUST00000165704.2	polypyrimidine tract binding protein 1
408	Cd151	ENSMUST00000106000.3	CD151 antigen
409	Zbtb38	ENSMUST00000152594.2	zinc finger and BTB domain containing 38
410	Dio2	ENSMUST00000082432.3	deiodinase, iodothyronine, type II
411	Ipo13	ENSMUST00000036156.5	importin 13
412	Olr1	ENSMUST00000032265.7	oxidized low density lipoprotein (lectin-like) receptor 1
413	Dnajb1	ENSMUST00000005620.8	DnaJ (Hsp40) homolog, subfamily B, member 1
414	Scn2b	ENSMUST00000170998.2	sodium channel, voltage-gated, type II, beta
415	Dyrk1a	ENSMUST00000023614.4	dual-specificity tyrosine-(Y)-phosphorylation regulated kinase 1a
416	Car7	ENSMUST00000159416.2	carbonic anhydrase 7
417	Fyttd1	ENSMUST00000171325.2	forty-two-three domain containing 1
418	Tmem200c	ENSMUST00000178545.1	transmembrane protein 200C
419	Cnot6l	ENSMUST00000155901.2	CCR4-NOT transcription complex, subunit 6-like
420	Tmem87b	ENSMUST00000110325.2	transmembrane protein 87B
421	Slc33a1	ENSMUST00000029402.9	solute carrier family 33 (acetyl-CoA transporter), member 1
422	Gnai1	ENSMUST00000074694.5	guanine nucleotide binding protein (G protein), alpha inhibiting 1
423	Ctnnal1	ENSMUST00000045142.9	catenin (cadherin associated protein), alpha-like 1
424	Atf2	ENSMUST00000112010.3	activating transcription factor 2
425	Nptx1	ENSMUST00000026670.4	neuronal pentraxin 1
426	Btbd10	ENSMUST00000047091.8	BTB (POZ) domain containing 10
427	Fam19a4	ENSMUST00000089295.4	family with sequence similarity 19, member A4
428	Stam	ENSMUST00000102960.5	signal transducing adaptor molecule (SH3 domain and ITAM motif) 1
429	Tmem28	ENSMUST00000096363.2	transmembrane protein 28
430	Gpt2	ENSMUST00000034136.6	glutamic pyruvate transaminase (alanine aminotransferase) 2

431	Bahd1	ENSMUST00000036578.6	bromo adjacent homology domain containing 1
432	Got1	ENSMUST00000026196.8	glutamate oxaloacetate transaminase 1, soluble
433	Zbtb41	ENSMUST00000039867.7	zinc finger and BTB domain containing 41 homolog
434	Ikzf2	ENSMUST00000027146.3	IKAROS family zinc finger 2
435	Rbfox2	ENSMUST00000171751.2	RNA binding protein, fox-1 homolog (C. elegans) 2
436	Fli1	ENSMUST00000016231.8	Friend leukemia integration 1
437	Mier3	ENSMUST00000109272.3	mesoderm induction early response 1, family member 3
438	Nabp1	ENSMUST00000027279.6	nucleic acid binding protein 1
439	Ptma	ENSMUST00000186255.1	prothymosin alpha
440	Slc44a5	ENSMUST00000089948.5	solute carrier family 44, member 5
441	Inhba	ENSMUST00000164993.1	inhibin beta-A
442	Acvr1b	ENSMUST00000000544.10	activin A receptor, type 1B
443	Htr1a	ENSMUST00000022235.4	5-hydroxytryptamine (serotonin) receptor 1A
444	Itpripl2	ENSMUST00000178344.1	inositol 1,4,5-triphosphate receptor interacting protein-like 2
445	Lpp	ENSMUST00000038053.7	LIM domain containing preferred translocation partner in lipoma
446	Snip1	ENSMUST00000052183.6	Smad nuclear interacting protein 1
447	Golph3	ENSMUST00000059680.5	golgi phosphoprotein 3
448	Pias3	ENSMUST00000064900.10	protein inhibitor of activated STAT 3
449	Sgsm1	ENSMUST00000048112.7	small G protein signaling modulator 1
450	Rab40b	ENSMUST00000106107.2	Rab40B, member RAS oncogene family
451	Opcml	ENSMUST00000115243.3	opioid binding protein/cell adhesion molecule-like
452	Hist1h2ak	ENSMUST00000074752.3	histone cluster 1, H2ak
453	Cep104	ENSMUST00000047497.9	centrosomal protein 104
454	Fam73b	ENSMUST00000077977.8	family with sequence similarity 73, member B
455	Ppara	ENSMUST00000109422.2	peroxisome proliferator activated receptor alpha
456	B230219D22Rik	ENSMUST00000057844.8	RIKEN cDNA B230219D22 gene
457	Cd47	ENSMUST00000084838.7	CD47 antigen (Rh-related antigen, integrin-associated signal transducer)
458	Srsf1	ENSMUST00000079866.5	serine/arginine-rich splicing factor 1
459	Hoxb1	ENSMUST00000019117.2	homeobox B1
460	Adamts18	ENSMUST00000093113.4	a disintegrin-like and metallopeptidase (reprolysin type) with thrombospondin type 1 motif, 18
461	Smurf2	ENSMUST00000103067.4	SMAD specific E3 ubiquitin protein ligase 2
462	Kif13a	ENSMUST00000056978.7	kinesin family member 13A
463	Jup	ENSMUST00000001592.9	junction plakoglobin
464	Anxa2	ENSMUST00000034756.9	annexin A2
465	Frem2	ENSMUST00000091137.4	Fras1 related extracellular matrix protein 2
466	Dcun1d4	ENSMUST00000063882.6	DCN1, defective in cullin neddylation 1, domain containing 4 (S. cerevisiae)
467	Hmgxb3	ENSMUST00000091884.4	HMG box domain containing 3
468	Pik3r3	ENSMUST00000030464.8	phosphatidylinositol 3 kinase, regulatory subunit, polypeptide 3 (p55)
469	Kif1c	ENSMUST00000102554.2	kinesin family member 1C
470	Dnajc3	ENSMUST00000022734.7	DnaJ (Hsp40) homolog, subfamily C, member 3
471	Kcna4	ENSMUST00000037012.2	potassium voltage-gated channel, shaker-related subfamily, member 4
472	Fxr1	ENSMUST00000001620.8	fragile X mental retardation gene 1, autosomal homolog
473	Tsnax	ENSMUST00000075896.6	translin-associated factor X
474	Nfkb1	ENSMUST00000029812.8	nuclear factor of kappa light polypeptide gene enhancer in B cells 1, p105
475	Fbrs	ENSMUST00000048896.6	fibrosin
476	Uhmk1	ENSMUST00000123399.1	U2AF homology motif (UHM) kinase 1
477	Zfp280d	ENSMUST00000098576.4	zinc finger protein 280D
478	Hn1l	ENSMUST00000024981.7	hematological and neurological expressed 1-like
479	Slc39a14	ENSMUST00000068044.8	solute carrier family 39 (zinc transporter), member 14
480	2610528J11Rik	ENSMUST00000030261.5	RIKEN cDNA 2610528J11 gene
481	Tra2b	ENSMUST00000161286.2	transformer 2 beta homolog (Drosophila)
482	Fnip2	ENSMUST00000076136.5	folliculin interacting protein 2
483	Ikzf5	ENSMUST00000046306.9	IKAROS family zinc finger 5

484	Tln1	ENSMUST00000030187.8	talin 1
485	Hlcs	ENSMUST00000163193.2	holocarboxylase synthetase (biotin- [propriony-Coenzyme A-carboxylase (ATP-hydrolysing)] ligase)
486	Stk38l	ENSMUST00000001675.8	serine/threonine kinase 38 like
487	Trim2	ENSMUST00000107692.2	tripartite motif-containing 2
488	Mef2c	ENSMUST00000163888.2	myocyte enhancer factor 2C
489	Cc2d1a	ENSMUST00000117424.3	coiled-coil and C2 domain containing 1A
490	Six4	ENSMUST00000043208.7	sine oculis-related homeobox 4
491	Adpgk	ENSMUST00000026266.7	ADP-dependent glucokinase
492	Kif21a	ENSMUST00000088614.6	kinesin family member 21A
493	Pura	ENSMUST00000051301.3	purine rich element binding protein A
494	Phf19	ENSMUST00000028232.3	PHD finger protein 19
495	Dr1	ENSMUST00000031190.4	down-regulator of transcription 1
496	Wiz	ENSMUST00000087703.6	widely-interspaced zinc finger motifs
497	Ephb4	ENSMUST00000111054.1	Eph receptor B4
498	Dolpp1	ENSMUST00000028209.9	dolichyl pyrophosphate phosphatase 1
499	Pter	ENSMUST00000028063.5	phosphotriesterase related
500	Supt7l	ENSMUST00000065388.5	suppressor of Ty 7-like
501	2310035C23Rik	ENSMUST00000086721.4	RIKEN cDNA 2310035C23 gene
502	En2	ENSMUST00000036177.8	engrailed 2
503	Prdm1	ENSMUST00000039174.5	PR domain containing 1, with ZNF domain
504	Gjd2	ENSMUST00000090275.4	gap junction protein, delta 2
505	Tlk1	ENSMUST00000038584.8	tousled-like kinase 1
506	Pabpc4l	ENSMUST00000166505.1	poly(A) binding protein, cytoplasmic 4-like
507	Sall4	ENSMUST00000075044.4	sal-like 4 (Drosophila)
508	Rps6ka4	ENSMUST00000170516.3	ribosomal protein S6 kinase, polypeptide 4
509	Stx1a	ENSMUST00000005509.5	syntaxin 1A (brain)
510	Zdhhc7	ENSMUST00000034280.7	zinc finger, DHHC domain containing 7
511	Kif1b	ENSMUST00000030806.5	kinesin family member 1B
512	Ctps2	ENSMUST00000033727.8	cytidine 5'-triphosphate synthase 2
513	Cntn4	ENSMUST00000089208.3	contactin 4
514	Mknk2	ENSMUST00000003433.6	MAP kinase-interacting serine/threonine kinase 2
515	Cog3	ENSMUST00000049168.7	component of oligomeric golgi complex 3
516	Ptbp2	ENSMUST00000029780.7	polypyrimidine tract binding protein 2
517	Wscd2	ENSMUST00000094452.3	WSC domain containing 2
518	6330408A02Rik	ENSMUST00000119558.2	RIKEN cDNA 6330408A02 gene
519	Stt3b	ENSMUST00000035010.8	STT3, subunit of the oligosaccharyltransferase complex, homolog B (S. cerevisiae)
520	Cyp2u1	ENSMUST00000106337.2	cytochrome P450, family 2, subfamily u, polypeptide 1
521	Cpeb4	ENSMUST00000109412.3	cytoplasmic polyadenylation element binding protein 4
522	Rtn4rl1	ENSMUST00000102514.3	reticulon 4 receptor-like 1
523	Ikzf4	ENSMUST00000133342.2	IKAROS family zinc finger 4
524	Specc1l	ENSMUST00000105421.3	sperm antigen with calponin homology and coiled-coil domains 1-like
525	Gm6625	ENSMUST00000177870.1	predicted gene 6625
526	Col4a2	ENSMUST00000033899.8	collagen, type IV, alpha 2
527	Nid2	ENSMUST00000022340.3	nidogen 2
528	Tmem200a	ENSMUST00000066049.6	transmembrane protein 200A
529	Soat1	ENSMUST00000189661.1	sterol O-acyltransferase 1
530	Magi2	ENSMUST00000101558.4	membrane associated guanylate kinase, WW and PDZ domain containing 2
531	Bicc1	ENSMUST00000143791.2	bicaudal C homolog 1 (Drosophila)
532	Ntrk3	ENSMUST00000039431.8	neurotrophic tyrosine kinase, receptor, type 3
533	Zfp407	ENSMUST00000125763.1	zinc finger protein 407
534	Pak2	ENSMUST00000023467.8	p21 protein (Cdc42/Rac)-activated kinase 2
535	Pskh1	ENSMUST00000049699.8	protein serine kinase H1
536	Tmem248	ENSMUST00000065329.7	transmembrane protein 248

537	Rbm24	ENSMUST00000037923.3	RNA binding motif protein 24
538	Phtf2	ENSMUST00000118174.2	putative homeodomain transcription factor 2
539	Slc30a7	ENSMUST00000067485.3	solute carrier family 30 (zinc transporter), member 7
540	Bcl2l11	ENSMUST00000110341.3	BCL2-like 11 (apoptosis facilitator)
541	Map3k1	ENSMUST00000109267.3	mitogen-activated protein kinase kinase kinase 1
542	Rap2c	ENSMUST00000053593.7	RAP2C, member of RAS oncogene family
543	Il1rapl1	ENSMUST00000113966.2	interleukin 1 receptor accessory protein-like 1
544	Fgf14	ENSMUST00000095529.4	fibroblast growth factor 14
545	Fam196b	ENSMUST00000165963.2	family with sequence similarity 196, member B
546	Slc30a3	ENSMUST00000031037.8	solute carrier family 30 (zinc transporter), member 3
547	Vamp1	ENSMUST00000100942.3	vesicle-associated membrane protein 1
548	Zdhhc21	ENSMUST00000030110.9	zinc finger, DHHC domain containing 21
549	Cmtr2	ENSMUST00000056972.5	cap methyltransferase 2
550	Col27a1	ENSMUST00000036300.7	collagen, type XXVII, alpha 1
551	Fktn	ENSMUST00000128667.2	fukutin
552	Yipf4	ENSMUST00000024873.6	Yip1 domain family, member 4
553	Lsm14a	ENSMUST00000085585.6	LSM14 homolog A (SCD6, S. cerevisiae)
554	Pcdh1	ENSMUST00000160721.2	protocadherin 1
555	Ap2m1	ENSMUST00000007216.8	adaptor-related protein complex 2, mu 1 subunit
556	Myo1d	ENSMUST00000041065.8	myosin ID
557	Sp8	ENSMUST00000063918.2	trans-acting transcription factor 8
558	Asic1	ENSMUST00000023758.7	acid-sensing (proton-gated) ion channel 1
559	Lancl3	ENSMUST00000069763.2	LanC lantibiotic synthetase component C-like 3 (bacterial)
560	Osbpl3	ENSMUST00000114468.3	oxysterol binding protein-like 3
561	Galntl6	ENSMUST00000098757.3	UDP-N-acetyl-alpha-D-galactosamine:polypeptide N-acetylgalactosaminyltransferase-like 6
562	Mapkapk2	ENSMUST00000016672.5	MAP kinase-activated protein kinase 2
563	Tram1	ENSMUST00000027068.5	translocating chain-associating membrane protein 1
564	Zfp286	ENSMUST00000108705.2	zinc finger protein 286
565	Shroom3	ENSMUST00000113054.3	shroom family member 3
566	Slc9a7	ENSMUST00000072451.5	solute carrier family 9 (sodium/hydrogen exchanger), member 7
567	Esrrb	ENSMUST00000116402.4	estrogen related receptor, beta
568	Nmt2	ENSMUST00000081932.7	N-myristoyltransferase 2
569	Lhfp	ENSMUST00000059562.8	lipoma HMGIC fusion partner
570	Celf4	ENSMUST00000025117.7	CUGBP, Elav-like family member 4
571	Slc25a37	ENSMUST00000037064.4	solute carrier family 25, member 37
572	Gsk3b	ENSMUST00000023507.7	glycogen synthase kinase 3 beta
573	Ccser2	ENSMUST00000090024.5	coiled-coil serine rich 2
574	Fndc3b	ENSMUST00000046157.4	fibronectin type III domain containing 3B
575	Btbd7	ENSMUST00000045652.6	BTB (POZ) domain containing 7
576	Ccdc126	ENSMUST00000055559.7	coiled-coil domain containing 126
577	Sptlc2	ENSMUST00000021424.4	serine palmitoyltransferase, long chain base subunit 2
578	Myh11	ENSMUST00000090287.3	myosin, heavy polypeptide 11, smooth muscle
579	Nr2e1	ENSMUST00000019938.5	nuclear receptor subfamily 2, group E, member 1
580	Epha7	ENSMUST00000029964.6	Eph receptor A7
581	Axl	ENSMUST00000002677.5	AXL receptor tyrosine kinase
582	Fignl2	ENSMUST00000178140.1	fidgetin-like 2
583	Wdtc1	ENSMUST00000105906.1	WD and tetratricopeptide repeats 1
584	Upf3b	ENSMUST00000076265.7	UPF3 regulator of nonsense transcripts homolog B (yeast)
585	Snap23	ENSMUST00000110711.3	synaptosomal-associated protein 23
586	Sin3a	ENSMUST00000167715.2	transcriptional regulator, SIN3A (yeast)
587	Rbms1	ENSMUST00000028347.7	RNA binding motif, single stranded interacting protein 1
588	Hunk	ENSMUST00000065856.6	hormonally upregulated Neu-associated kinase
589	Cmah	ENSMUST00000167746.2	cytidine monophospho-N-acetylneuraminic acid hydroxylase
590	Pptc7	ENSMUST00000053426.9	PTC7 protein phosphatase homolog (S. cerevisiae)
591	Oxsr1	ENSMUST00000040853.5	oxidative-stress responsive 1

592	Cyhr1	ENSMUST00000081291.7	cysteine and histidine rich 1
593	Fzd7	ENSMUST00000114246.3	frizzled homolog 7 (Drosophila)
594	D1Ertd622e	ENSMUST00000053033.8	DNA segment, Chr 1, ERATO Doi 622, expressed
595	Arfip2	ENSMUST00000131446.2	ADP-ribosylation factor interacting protein 2
596	Shb	ENSMUST00000061986.6	src homology 2 domain-containing transforming protein B
597	Lin7a	ENSMUST00000020057.9	lin-7 homolog A (C. elegans)
598	Hist3h2a	ENSMUST00000108817.4	histone cluster 3, H2a
599	Fgf5	ENSMUST00000031280.1	fibroblast growth factor 5
600	Fgf9	ENSMUST00000165526.2	fibroblast growth factor 9
601	Mmp16	ENSMUST00000029881.4	matrix metallopeptidase 16
602	Elmod2	ENSMUST00000177594.2	ELMO/CED-12 domain containing 2
603	Pde7b	ENSMUST00000020165.8	phosphodiesterase 7B
604	Ptprg	ENSMUST00000022264.7	protein tyrosine phosphatase, receptor type, G
605	Cxcr4	ENSMUST00000052172.6	chemokine (C-X-C motif) receptor 4
606	Ppp4r1	ENSMUST00000073104.5	protein phosphatase 4, regulatory subunit 1
607	Erbb2ip	ENSMUST00000022222.6	Erbb2 interacting protein
608	Gopc	ENSMUST00000105475.3	golgi associated PDZ and coiled-coil motif containing
609	Lhx5	ENSMUST00000031591.8	LIM homeobox protein 5
610	4933426M11Rik	ENSMUST00000068519.5	RIKEN cDNA 4933426M11 gene
611	Ubash3b	ENSMUST00000044155.9	ubiquitin associated and SH3 domain containing, B
612	Slc6a2	ENSMUST00000072939.6	solute carrier family 6 (neurotransmitter transporter, noradrenalin), member 2
613	Ccdc6	ENSMUST00000147545.2	coiled-coil domain containing 6
614	4930579G24Rik	ENSMUST00000029388.8	RIKEN cDNA 4930579G24 gene
615	Scube2	ENSMUST00000007423.6	signal peptide, CUB domain, EGF-like 2
616	Cbx5	ENSMUST00000118152.2	chromobox 5
617	Scyl3	ENSMUST00000027876.5	SCY1-like 3 (S. cerevisiae)
618	Creb3l2	ENSMUST00000041093.5	cAMP responsive element binding protein 3-like 2
619	Lgalsl	ENSMUST00000047028.8	lectin, galactoside binding-like
620	Creb5	ENSMUST00000047450.7	cAMP responsive element binding protein 5
621	Fam179b	ENSMUST00000066296.7	family with sequence similarity 179, member B
622	Tmem170b	ENSMUST00000129449.1	transmembrane protein 170B
623	Zdhhc5	ENSMUST00000035840.5	zinc finger, DHHC domain containing 5
624	Kcna1	ENSMUST00000055168.3	potassium voltage-gated channel, shaker-related subfamily, member 1
625	Mbnl1	ENSMUST00000099087.2	muscleblind-like 1 (Drosophila)
626	Tgfbr1	ENSMUST00000007757.9	transforming growth factor, beta receptor I
627	Ppip5k2	ENSMUST00000042509.7	diphosphoinositol pentakisphosphate kinase 2
628	Efna1	ENSMUST00000029566.3	ephrin A1
629	Klhl42	ENSMUST00000036003.7	kelch-like 42
630	Sos1	ENSMUST00000068714.5	son of sevenless homolog 1 (Drosophila)
631	Ubr5	ENSMUST00000110336.2	ubiquitin protein ligase E3 component n-recognin 5
632	March1	ENSMUST00000110255.2	membrane-associated ring finger (C3HC4) 1
633	Zfp202	ENSMUST00000168832.1	zinc finger protein 202
634	Lin7c	ENSMUST00000028583.7	lin-7 homolog C (C. elegans)
635	S100pbp	ENSMUST00000106061.3	S100P binding protein
636	Slc8a1	ENSMUST00000163680.3	solute carrier family 8 (sodium/calcium exchanger), member 1
637	Esyt2	ENSMUST00000100986.2	extended synaptotagmin-like protein 2
638	Thap2	ENSMUST00000020346.4	THAP domain containing, apoptosis associated protein 2
639	Ccne1	ENSMUST00000108023.4	cyclin E1
640	Rorb	ENSMUST00000040153.9	RAR-related orphan receptor beta
641	Rsc1a1	ENSMUST00000105782.1	regulatory solute carrier protein, family 1, member 1
642	Hs3st3b1	ENSMUST00000094103.3	heparan sulfate (glucosamine) 3-O-sulfotransferase 3B1
643	Pcsk6	ENSMUST00000055576.6	proprotein convertase subtilisin/kexin type 6
644	Pank3	ENSMUST00000018990.7	pantothenate kinase 3
645	Gprasp2	ENSMUST00000173804.2	G protein-coupled receptor associated sorting protein 2
646	Rab38	ENSMUST00000107256.2	RAB38, member RAS oncogene family

647	Arhgef2	ENSMUST00000176500.2	rho/rac guanine nucleotide exchange factor (GEF) 2
648	Dpp4	ENSMUST00000047812.7	dipeptidylpeptidase 4
649	A730020M07Rik	ENSMUST00000050571.4	RIKEN cDNA A730020M07 gene
650	Megf10	ENSMUST00000075770.7	multiple EGF-like-domains 10
651	Phospho1	ENSMUST00000054173.3	phosphatase, orphan 1
652	Dock9	ENSMUST00000100299.4	dedicator of cytokinesis 9
653	Tgoln1	ENSMUST00000070524.4	trans-golgi network protein
654	Pik3cb	ENSMUST00000035037.8	phosphatidylinositol 3-kinase, catalytic, beta polypeptide
655	Cnot7	ENSMUST00000034012.4	CCR4-NOT transcription complex, subunit 7
656	Hiat1	ENSMUST00000029570.6	hippocampus abundant gene transcript 1
657	Mbtps1	ENSMUST00000098362.4	membrane-bound transcription factor peptidase, site 1
658	Rasgrf2	ENSMUST00000099326.4	RAS protein-specific guanine nucleotide-releasing factor 2
659	Fyco1	ENSMUST00000167595.3	FYVE and coiled-coil domain containing 1
660	Vat1	ENSMUST00000040430.7	vesicle amine transport protein 1 homolog (T californica)
661	Chsy1	ENSMUST00000036372.6	chondroitin sulfate synthase 1
662	Foxf2	ENSMUST00000042054.2	forkhead box F2
663	Trim71	ENSMUST00000111816.2	tripartite motif-containing 71
664	Crybg3	ENSMUST00000044604.9	beta-gamma crystallin domain containing 3
665	5031439G07Rik	ENSMUST00000047144.7	RIKEN cDNA 5031439G07 gene
666	Antxr2	ENSMUST00000031281.9	anthrax toxin receptor 2
667	Entpd5	ENSMUST00000021662.6	ectonucleoside triphosphate diphosphohydrolase 5
668	Psen1	ENSMUST00000101225.1	presenilin 1
669	Pdk4	ENSMUST00000019721.4	pyruvate dehydrogenase kinase, isoenzyme 4
670	Prrt2	ENSMUST00000159916.1	proline-rich transmembrane protein 2
671	Slc6a6	ENSMUST00000032185.7	solute carrier family 6 (neurotransmitter transporter, taurine), member 6
672	Trim55	ENSMUST00000029139.7	tripartite motif-containing 55
673	Lmx1a	ENSMUST00000111377.2	LIM homeobox transcription factor 1 alpha
674	Dync1li2	ENSMUST00000041769.6	dynein, cytoplasmic 1 light intermediate chain 2
675	Tmtc1	ENSMUST00000060095.9	transmembrane and tetratricopeptide repeat containing 1
676	Adam10	ENSMUST00000067880.7	a disintegrin and metallopeptidase domain 10
677	Gpam	ENSMUST00000061856.5	glycerol-3-phosphate acyltransferase, mitochondrial
678	Sbno1	ENSMUST00000065263.6	sno, strawberry notch homolog 1 (Drosophila)
679	Sh3bgrl2	ENSMUST00000113215.4	SH3 domain binding glutamic acid-rich protein like 2
680	Fam43b	ENSMUST00000105032.3	family with sequence similarity 43, member B
681	Zfp263	ENSMUST00000162207.1	zinc finger protein 263
682	Dbnl	ENSMUST00000102928.4	drebrin-like
683	Ddi2	ENSMUST00000102484.4	DNA-damage inducible protein 2
684	Adamts9	ENSMUST00000113438.2	a disintegrin-like and metallopeptidase (reprolysin type) with thrombospondin type 1 motif, 9
685	Ralgds	ENSMUST00000028170.9	ral guanine nucleotide dissociation stimulator
686	Gabrb2	ENSMUST00000007797.4	gamma-aminobutyric acid (GABA) A receptor, subunit beta 2
687	D230025D16Rik	ENSMUST00000034361.4	RIKEN cDNA D230025D16 gene
688	Diap1	ENSMUST00000115634.2	diaphanous homolog 1 (Drosophila)
689	Ak4	ENSMUST00000102780.2	adenylate kinase 4
690	Ank	ENSMUST00000022875.6	progressive ankylosis
691	Ncor2	ENSMUST00000111398.2	nuclear receptor co-repressor 2
692	Spag9	ENSMUST00000041956.8	sperm associated antigen 9
693	Pik3c2a	ENSMUST00000170430.1	phosphatidylinositol 3-kinase, C2 domain containing, alpha polypeptide
694	Tulp4	ENSMUST00000149756.2	tubby like protein 4
695	Camkk2	ENSMUST00000111668.3	calcium/calmodulin-dependent protein kinase kinase 2, beta
696	Adamts6	ENSMUST00000065766.6	a disintegrin-like and metallopeptidase (reprolysin type) with thrombospondin type 1 motif, 6
697	Vcl	ENSMUST00000022369.7	vinculin
698	2310003H01Rik	ENSMUST00000026448.9	RIKEN cDNA 2310003H01 gene
699	Ciapin1	ENSMUST00000162538.2	cytokine induced apoptosis inhibitor 1

700	Prim1	ENSMUST00000026461.7	DNA primase, p49 subunit
701	Slc25a35	ENSMUST00000018884.5	solute carrier family 25, member 35
702	Cecr2	ENSMUST00000112686.2	cat eye syndrome chromosome region, candidate 2
703	Gdf11	ENSMUST00000026408.6	growth differentiation factor 11
704	Ccng1	ENSMUST00000020576.7	cyclin G1
705	Gpbp1l1	ENSMUST00000030460.9	GC-rich promoter binding protein 1-like 1
706	Rhoj	ENSMUST00000055390.5	ras homolog gene family, member J
707	Smap2	ENSMUST00000043200.7	small ArfGAP 2
708	Pcsk2	ENSMUST00000028905.9	proprotein convertase subtilisin/kexin type 2
709	Chd3	ENSMUST00000108661.2	chromodomain helicase DNA binding protein 3
710	Plekha1	ENSMUST00000120441.2	pleckstrin homology domain containing, family A (phosphoinositide binding specific) member 1
711	1700019D03Rik	ENSMUST00000050567.5	RIKEN cDNA 1700019D03 gene
712	Zfp395	ENSMUST00000066994.6	zinc finger protein 395
713	Parg	ENSMUST00000022470.9	poly (ADP-ribose) glycohydrolase
714	Chmp2b	ENSMUST00000004965.6	charged multivesicular body protein 2B
715	Hmg20a	ENSMUST00000034879.3	high mobility group 20A
716	Slc39a9	ENSMUST00000085245.5	solute carrier family 39 (zinc transporter), member 9
717	Fgf10	ENSMUST00000022246.8	fibroblast growth factor 10
718	Zfp319	ENSMUST00000057717.6	zinc finger protein 319
719	Lmna	ENSMUST00000029699.7	lamin A
720	Arid1b	ENSMUST00000115797.3	AT rich interactive domain 1B (SWI-like)
721	Bace1	ENSMUST00000034591.5	beta-site APP cleaving enzyme 1
722	Spock1	ENSMUST00000185502.1	sparc/osteonectin, cwcv and kazal-like domains proteoglycan 1
723	Alg6	ENSMUST00000097961.3	asparagine-linked glycosylation 6 (alpha-1,3,-glucosyltransferase)
724	Chst4	ENSMUST00000109222.2	carbohydrate (chondroitin 6/keratan) sulfotransferase 4
725	Dyrk2	ENSMUST00000004281.8	dual-specificity tyrosine-(Y)-phosphorylation regulated kinase 2
726	Srsf6	ENSMUST00000130411.1	serine/arginine-rich splicing factor 6
727	H6pd	ENSMUST00000084117.7	hexose-6-phosphate dehydrogenase (glucose 1-dehydrogenase)
728	Cpeb1	ENSMUST00000098331.4	cytoplasmic polyadenylation element binding protein 1
729	Lzts3	ENSMUST00000089561.4	leucine zipper, putative tumor suppressor family member 3
730	Olfml2a	ENSMUST00000057279.5	olfactomedin-like 2A
731	Gad1	ENSMUST00000094934.5	glutamate decarboxylase 1
732	Pak6	ENSMUST00000099557.4	p21 protein (Cdc42/Rac)-activated kinase 6
733	Afap1	ENSMUST00000064571.5	actin filament associated protein 1
734	Fbxo47	ENSMUST00000093939.3	F-box protein 47
735	Fam117b	ENSMUST00000036540.6	family with sequence similarity 117, member B
736	Cdk8	ENSMUST00000031640.9	cyclin-dependent kinase 8
737	Myo19	ENSMUST00000093969.5	myosin XIX
738	Gm12353	ENSMUST00000108250.2	predicted gene 12353
739	Nfib	ENSMUST00000107245.3	nuclear factor I/B
740	Fam222b	ENSMUST00000073705.6	family with sequence similarity 222, member B
741	Txndc5	ENSMUST00000035988.9	thioredoxin domain containing 5
742	Fsd1l	ENSMUST00000132151.2	fibronectin type III and SPRY domain containing 1-like
743	Zfhx3	ENSMUST00000043896.9	zinc finger homeobox 3
744	Vcan	ENSMUST00000109546.3	versican
745	Fbxo33	ENSMUST00000043204.7	F-box protein 33
746	Fst	ENSMUST00000022287.5	follistatin
747	Foxn3	ENSMUST00000046859.5	forkhead box N3
748	Srpk1	ENSMUST00000130643.2	serine/arginine-rich protein specific kinase 1
749	Dbt	ENSMUST00000000349.6	dihydrolipoamide branched chain transacylase E2
750	Arfgef1	ENSMUST00000088615.5	ADP-ribosylation factor guanine nucleotide-exchange factor 1(brefeldin A-inhibited)
751	Sh3pxd2b	ENSMUST00000038753.5	SH3 and PX domains 2B
752	Isl1	ENSMUST00000036060.7	ISL1 transcription factor, LIM/homeodomain
753	Lrig2	ENSMUST00000046316.6	leucine-rich repeats and immunoglobulin-like domains 2

754	Vwa7	ENSMUST00000007245.2	von Willebrand factor A domain containing 7
755	Ranbp17	ENSMUST00000102815.4	RAN binding protein 17
756	Fnip1	ENSMUST00000046835.8	folliculin interacting protein 1
757	Abl2	ENSMUST00000166172.3	v-abl Abelson murine leukemia viral oncogene 2 (arg, Abelson-related gene)
758	Abcd1	ENSMUST00000002084.8	ATP-binding cassette, sub-family D (ALD), member 1
759	Asb7	ENSMUST00000124899.2	ankyrin repeat and SOCS box-containing 7
760	Kdm2a	ENSMUST00000047898.8	lysine (K)-specific demethylase 2A
761	Cnnm4	ENSMUST00000153128.1	cyclin M4
762	Phf8	ENSMUST00000046950.7	PHD finger protein 8
763	Runx1	ENSMUST00000023673.8	runt related transcription factor 1
764	Ppm1a	ENSMUST00000021514.8	protein phosphatase 1A, magnesium dependent, alpha isoform
765	Ankrd12	ENSMUST00000038116.6	ankyrin repeat domain 12
766	Celf5	ENSMUST00000118763.2	CUGBP, Elav-like family member 5
767	Sema3a	ENSMUST00000030714.7	sema domain, immunoglobulin domain (Ig), short basic domain, secreted, (semaphorin) 3A
768	Zdbf2	ENSMUST00000114132.2	zinc finger, DBF-type containing 2
769	D10Bwg1379e	ENSMUST00000019999.5	DNA segment, Chr 10, Brigham & Women's Genetics 1379 expressed
770	Tdh	ENSMUST00000022522.9	L-threonine dehydrogenase
771	Asxl2	ENSMUST00000111215.4	additional sex combs like 2 (Drosophila)
772	Chst15	ENSMUST00000077472.4	carbohydrate (N-acetylgalactosamine 4-sulfate 6-O) sulfotransferase 15
773	Ttyh2	ENSMUST00000045779.5	tweety homolog 2 (Drosophila)
774	Agap1	ENSMUST00000027521.9	ArfGAP with GTPase domain, ankyrin repeat and PH domain 1
775	Kcnh5	ENSMUST00000042299.2	potassium voltage-gated channel, subfamily H (eag-related), member 5
776	Gabbr2	ENSMUST00000107749.2	gamma-aminobutyric acid (GABA) B receptor, 2
777	Ubfd1	ENSMUST00000033158.4	ubiquitin family domain containing 1
778	Arf6	ENSMUST00000050063.7	ADP-ribosylation factor 6
779	Dot1l	ENSMUST00000105336.3	DOT1-like, histone H3 methyltransferase (S. cerevisiae)
780	Kif13b	ENSMUST00000100473.4	kinesin family member 13B
781	Dixdc1	ENSMUST00000034566.9	DIX domain containing 1
782	Sdc1	ENSMUST00000020911.8	syndecan 1
783	Gad1-ps	ENSMUST00000167243.1	glutamate decarboxylase 1, pseudogene
784	Rnf112	ENSMUST00000060255.8	ring finger protein 112
785	Tmcc1	ENSMUST00000088896.4	transmembrane and coiled coil domains 1
786	Rnf11	ENSMUST00000030284.4	ring finger protein 11
787	Ppip5k1	ENSMUST00000052029.4	diphosphoinositol pentakisphosphate kinase 1
788	Map2k3	ENSMUST00000019076.4	mitogen-activated protein kinase kinase 3
789	Asxl1	ENSMUST00000109790.1	additional sex combs like 1
790	Zfp367	ENSMUST00000059817.5	zinc finger protein 367
791	Eif5	ENSMUST00000166123.2	eukaryotic translation initiation factor 5
792	Rspry1	ENSMUST00000060389.8	ring finger and SPRY domain containing 1
793	Wipf1	ENSMUST00000094681.5	WAS/WASL interacting protein family, member 1
794	Dis3l2	ENSMUST00000168237.2	DIS3 mitotic control homolog (S. cerevisiae)-like 2
795	Slc12a5	ENSMUST00000099092.4	solute carrier family 12, member 5
796	Micu3	ENSMUST00000068999.8	mitochondrial calcium uptake family, member 3
797	Gab2	ENSMUST00000004622.5	growth factor receptor bound protein 2-associated protein 2
798	Grik3	ENSMUST00000030676.7	glutamate receptor, ionotropic, kainate 3
799	Klf12	ENSMUST00000097079.4	Kruppel-like factor 12
800	Bend4	ENSMUST00000169190.1	BEN domain containing 4
801	Cbfa2t2	ENSMUST00000109725.2	core-binding factor, runt domain, alpha subunit 2, translocated to, 2 (human)
802	Zbtb44	ENSMUST00000115222.3	zinc finger and BTB domain containing 44
803	Atg13	ENSMUST00000076803.6	autophagy related 13
804	Ccni	ENSMUST00000058550.9	cyclin I
805	Edem3	ENSMUST00000059498.6	ER degradation enhancer, mannosidase alpha-like 3
806	Syt1	ENSMUST00000105276.2	synaptotagmin I

807	Gigyf1	ENSMUST00000031727.7	GRB10 interacting GYF protein 1
808	Pacsin1	ENSMUST00000045896.4	protein kinase C and casein kinase substrate in neurons 1
809	Mctp2	ENSMUST00000079323.6	multiple C2 domains, transmembrane 2
810	Serinc5	ENSMUST00000049488.7	serine incorporator 5
811	Zfp362	ENSMUST00000106072.3	zinc finger protein 362
812	Kitl	ENSMUST00000105283.2	kit ligand
813	Rundc3b	ENSMUST00000047485.9	RUN domain containing 3B
814	Tcf19	ENSMUST00000161012.2	transcription factor 19
815	Ankrd52	ENSMUST00000014642.4	ankyrin repeat domain 52
816	Rs1	ENSMUST00000033650.8	retinoschisis (X-linked, juvenile) 1 (human)
817	Clcn5	ENSMUST00000004428.8	chloride channel 5
818	Hapln4	ENSMUST00000007738.9	hyaluronan and proteoglycan link protein 4
819	Zbtb39	ENSMUST00000054287.7	zinc finger and BTB domain containing 39
820	Glipr2	ENSMUST00000030202.8	GLI pathogenesis-related 2
821	Chst14	ENSMUST00000099546.5	carbohydrate (N-acetylgalactosamine 4-0) sulfotransferase 14
822	Cpeb3	ENSMUST00000079754.5	cytoplasmic polyadenylation element binding protein 3
823	Unkl	ENSMUST00000039734.6	unkempt-like (Drosophila)
824	Lmbrd2	ENSMUST00000090380.4	LMBR1 domain containing 2
825	Hipk3	ENSMUST00000028600.8	homeodomain interacting protein kinase 3
826	Rora	ENSMUST00000034766.8	RAR-related orphan receptor alpha
827	Cdh10	ENSMUST00000166873.3	cadherin 10
828	Mical3	ENSMUST00000098457.3	microtubule associated monooxygenase, calponin and LIM domain containing 3
829	Mkl2	ENSMUST00000149359.1	MKL/myocardin-like 2
830	Mfap3l	ENSMUST00000160719.2	microfibrillar-associated protein 3-like
831	Klf13	ENSMUST00000063694.8	Kruppel-like factor 13
832	Dgkb	ENSMUST00000040500.7	diacylglycerol kinase, beta
833	Abi1	ENSMUST00000140164.2	abl-interactor 1
834	Ccser1	ENSMUST00000126214.2	coiled-coil serine rich 1
835	Gm996	ENSMUST00000114217.2	predicted gene 996
836	Kirrel	ENSMUST00000159976.2	kin of IRRE like (Drosophila)
837	Shisa5	ENSMUST00000026737.6	shisa homolog 5 (Xenopus laevis)
838	Peak1	ENSMUST00000188142.1	pseudopodium-enriched atypical kinase 1
839	Lrrc1	ENSMUST00000183873.2	leucine rich repeat containing 1
840	Brpf3	ENSMUST00000004985.9	bromodomain and PHD finger containing, 3
841	Myrf	ENSMUST00000088013.6	myelin regulatory factor
842	Ddx3x	ENSMUST00000000804.6	DEAD/H (Asp-Glu-Ala-Asp/His) box polypeptide 3, X-linked
843	Hipk1	ENSMUST00000118317.2	homeodomain interacting protein kinase 1
844	Actr1a	ENSMUST00000040270.4	ARP1 actin-related protein 1A, centractin alpha
845	Glcci1	ENSMUST00000064285.9	glucocorticoid induced transcript 1
846	Zfp185	ENSMUST00000164800.2	zinc finger protein 185
847	Pitpnm3	ENSMUST00000075258.7	PITPNM family member 3
848	Rfesd	ENSMUST00000167271.2	Rieske (Fe-S) domain containing
849	Rnf169	ENSMUST00000080817.4	ring finger protein 169
850	Mtpn	ENSMUST00000031866.5	myotrophin
851	Epas1	ENSMUST00000024954.9	endothelial PAS domain protein 1
852	Rfx7	ENSMUST00000093820.4	regulatory factor X, 7
853	Tspan18	ENSMUST00000111265.3	tetraspanin 18
854	Zzz3	ENSMUST00000106100.3	zinc finger, ZZ domain containing 3
855	Nyap2	ENSMUST00000123285.1	neuronal tyrosine-phophorylated phosphoinositide 3-kinase adaptor 2
856	Rcor1	ENSMUST00000084968.8	REST corepressor 1
857	Kdm4c	ENSMUST00000077851.4	lysine (K)-specific demethylase 4C
858	Atf3	ENSMUST00000027941.8	activating transcription factor 3
859	Kctd2	ENSMUST00000106533.2	potassium channel tetramerisation domain containing 2
860	Tbc1d4	ENSMUST00000161991.2	TBC1 domain family, member 4
861	Zdhhc20	ENSMUST00000089473.3	zinc finger, DHHC domain containing 20

862	Rasd2	ENSMUST00000139848.2	RASD family, member 2
863	Cdyl2	ENSMUST00000109102.2	chromodomain protein, Y chromosome-like 2
864	Ppp1r13b	ENSMUST00000054815.8	protein phosphatase 1, regulatory (inhibitor) subunit 13B
865	Wdr6	ENSMUST00000068700.5	WD repeat domain 6
866	Gpr124	ENSMUST00000033876.8	G protein-coupled receptor 124
867	Iffo2	ENSMUST00000174078.1	intermediate filament family orphan 2
868	Tnks	ENSMUST00000033929.4	tankyrase, TRF1-interacting ankyrin-related ADP-ribose polymerase
869	Slc39a13	ENSMUST00000073575.6	solute carrier family 39 (metal ion transporter), member 13
870	Kctd10	ENSMUST00000102581.5	potassium channel tetramerisation domain containing 10
871	Mme	ENSMUST00000029400.1	membrane metallo endopeptidase
872	Alcam	ENSMUST00000023312.8	activated leukocyte cell adhesion molecule
873	Nfia	ENSMUST00000092532.7	nuclear factor I/A
874	Prrt3	ENSMUST00000101059.1	proline-rich transmembrane protein 3
875	Xrn1	ENSMUST00000185633.1	5'-3' exoribonuclease 1
876	Slitrk2	ENSMUST00000166241.1	SLIT and NTRK-like family, member 2
877	Gabrg3	ENSMUST00000068911.7	gamma-aminobutyric acid (GABA) A receptor, subunit gamma 3
878	Sez6	ENSMUST00000093995.4	seizure related gene 6
879	Kcnq2	ENSMUST00000149964.3	potassium voltage-gated channel, subfamily Q, member 2
880	Senp1	ENSMUST00000044189.10	SUMO1/sentrin specific peptidase 1
881	D16Ertd472e	ENSMUST00000114220.2	DNA segment, Chr 16, ERATO Doi 472, expressed
882	Pcdh7	ENSMUST00000094783.4	protocadherin 7
883	D17Wsu92e	ENSMUST00000114863.3	DNA segment, Chr 17, Wayne State University 92, expressed
884	Aatk	ENSMUST00000064307.4	apoptosis-associated tyrosine kinase
885	Scn4b	ENSMUST00000060125.5	sodium channel, type IV, beta
886	Bicd1	ENSMUST00000086829.5	bicaudal D homolog 1 (Drosophila)
887	Notch2	ENSMUST00000079812.6	notch 2
888	Carm1	ENSMUST00000115395.4	coactivator-associated arginine methyltransferase 1
889	Ranbp2	ENSMUST00000003310.5	RAN binding protein 2
890	Fmr1	ENSMUST00000088546.6	fragile X mental retardation syndrome 1
891	Srf	ENSMUST00000015749.5	serum response factor
892	Cbln4	ENSMUST00000087950.3	cerebellin 4 precursor protein
893	Zbtb34	ENSMUST00000113158.2	zinc finger and BTB domain containing 34
894	Cbx6	ENSMUST00000109623.2	chromobox 6
895	Ajuba	ENSMUST00000054487.8	ajuba LIM protein
896	Atp7a	ENSMUST00000055941.6	ATPase, Cu++ transporting, alpha polypeptide
897	Npy2r	ENSMUST00000029633.4	neuropeptide Y receptor Y2
898	Syt14	ENSMUST00000016344.7	synaptotagmin XIV
899	Nagpa	ENSMUST00000023911.5	N-acetylglucosamine-1-phosphodiester alpha-N-acetylglucosaminidase
900	Dhx40	ENSMUST00000018569.8	DEAH (Asp-Glu-Ala-His) box polypeptide 40
901	Rab5b	ENSMUST00000000727.2	RAB5B, member RAS oncogene family
902	Pex5l	ENSMUST00000108225.4	peroxisomal biogenesis factor 5-like
903	Hipk2	ENSMUST00000161779.2	homeodomain interacting protein kinase 2
904	Sptssb	ENSMUST00000171529.2	serine palmitoyltransferase, small subunit B
905	Ets1	ENSMUST00000034534.7	E26 avian leukemia oncogene 1, 5' domain
906	Larp1	ENSMUST00000178636.1	La ribonucleoprotein domain family, member 1
907	Ccdc88a	ENSMUST00000040182.7	coiled coil domain containing 88A
908	Aak1	ENSMUST00000089519.7	AP2 associated kinase 1
909	Col12a1	ENSMUST00000071750.7	collagen, type XII, alpha 1
910	Shc1	ENSMUST00000191485.1	src homology 2 domain-containing transforming protein C1
911	Ralb	ENSMUST00000004565.9	v-ral simian leukemia viral oncogene homolog B (ras related)
912	Rab11fip2	ENSMUST00000171986.2	RAB11 family interacting protein 2 (class I)
913	Zbtb46	ENSMUST00000029106.7	zinc finger and BTB domain containing 46
914	H13	ENSMUST00000089059.3	histocompatibility 13
915	Pgap1	ENSMUST00000097739.4	post-GPI attachment to proteins 1
916	Hecw2	ENSMUST00000120904.2	HECT, C2 and WW domain containing E3 ubiquitin protein ligase 2
917	Xpo4	ENSMUST00000174545.2	exportin 4

918	Asxl3	ENSMUST00000097655.3	additional sex combs like 3 (Drosophila)
919	Sgms1	ENSMUST00000099514.4	sphingomyelin synthase 1
920	Ube3a	ENSMUST00000107537.1	ubiquitin protein ligase E3A
921	Mthfd1l	ENSMUST00000117291.2	methylenetetrahydrofolate dehydrogenase (NADP+ dependent) 1-like
922	Mtmr10	ENSMUST00000032736.5	myotubularin related protein 10
923	Nmt1	ENSMUST00000021314.7	N-myristoyltransferase 1
924	Rgl1	ENSMUST00000111859.2	ral guanine nucleotide dissociation stimulator,-like 1
925	Ctdsp2	ENSMUST00000105256.3	CTD (carboxy-terminal domain, RNA polymerase II, polypeptide A) small phosphatase 2
926	Rmnd5a	ENSMUST00000002292.9	required for meiotic nuclear division 5 homolog A (S. cerevisiae)
927	Prkcz	ENSMUST00000103178.5	protein kinase C, zeta
928	Synj2bp	ENSMUST00000163402.2	synaptojanin 2 binding protein
929	Paqr3	ENSMUST00000112969.4	progestin and adipoQ receptor family member III
930	Tns1	ENSMUST00000169786.2	tensin 1
931	Ssx2ip	ENSMUST00000106153.3	synovial sarcoma, X breakpoint 2 interacting protein
932	Prps2	ENSMUST00000026839.4	phosphoribosyl pyrophosphate synthetase 2
933	Tm9sf3	ENSMUST00000025989.8	transmembrane 9 superfamily member 3
934	Scaf11	ENSMUST00000047835.6	SR-related CTD-associated factor 11
935	Atxn1	ENSMUST00000180110.2	ataxin 1
936	Cnot1	ENSMUST00000098473.5	CCR4-NOT transcription complex, subunit 1
937	Fbxl16	ENSMUST00000045692.7	F-box and leucine-rich repeat protein 16
938	Gpatch8	ENSMUST00000143842.1	G patch domain containing 8
939	Dstyk	ENSMUST00000045110.8	dual serine/threonine and tyrosine protein kinase
940	Tab3	ENSMUST00000048250.4	TGF-beta activated kinase 1/MAP3K7 binding protein 3
941	Impa1	ENSMUST00000118410.2	inositol (myo)-1(or 4)-monophosphatase 1
942	Ankrd40	ENSMUST00000051221.7	ankyrin repeat domain 40
943	Rnf38	ENSMUST00000098098.3	ring finger protein 38
944	Sik3	ENSMUST00000126865.2	SIK family kinase 3
945	Wipi2	ENSMUST00000036872.10	WD repeat domain, phosphoinositide interacting 2
946	Mical2	ENSMUST00000050149.6	microtubule associated monooxygenase, calponin and LIM domain containing 2
947	Clvs1	ENSMUST00000038841.8	clavesin 1
948	Klhl24	ENSMUST00000023509.3	kelch-like 24
949	Foxo1	ENSMUST00000053764.5	forkhead box O1
950	Sgcd	ENSMUST00000077221.5	sarcoglycan, delta (dystrophin-associated glycoprotein)
951	Src	ENSMUST00000029175.8	Rous sarcoma oncogene
952	Srcap	ENSMUST00000084563.5	Snf2-related CREBBP activator protein
953	Pdcd6ip	ENSMUST00000035086.7	programmed cell death 6 interacting protein
954	Aff1	ENSMUST00000054979.4	AF4/FMR2 family, member 1
955	Ube4b	ENSMUST00000103212.4	ubiquitination factor E4B
956	Rc3h1	ENSMUST00000161609.2	RING CCCH (C3H) domains 1
957	Amer1	ENSMUST00000084535.5	APC membrane recruitment 1
958	Atp8a1	ENSMUST00000037380.9	ATPase, aminophospholipid transporter (APLT), class I, type 8A, member 1
959	E130309D14Rik	ENSMUST00000100866.2	RIKEN cDNA E130309D14 gene
960	Adamts3	ENSMUST00000163159.2	a disintegrin-like and metallopeptidase (reprolysin type) with thrombospondin type 1 motif, 3
961	Tspan9	ENSMUST00000112173.2	tetraspanin 9
962	Frmd4a	ENSMUST00000075767.8	FERM domain containing 4A
963	Pou3f2	ENSMUST00000178174.2	POU domain, class 3, transcription factor 2
964	Entpd1	ENSMUST00000112231.3	ectonucleoside triphosphate diphosphohydrolase 1
965	Ubxn7	ENSMUST00000115151.3	UBX domain protein 7
966	Trim66	ENSMUST00000106739.2	tripartite motif-containing 66
967	Mycbp	ENSMUST00000030400.8	c-myc binding protein
968	Csnk1g2	ENSMUST00000079773.7	casein kinase 1, gamma 2
969	Slc16a10	ENSMUST00000092566.6	solute carrier family 16 (monocarboxylic acid transporters), member 10

970	Wapal	ENSMUST00000048263.8	wings apart-like homolog (Drosophila)
971	Rfx3	ENSMUST00000165566.2	regulatory factor X, 3 (influences HLA class II expression)
972	Mlxip	ENSMUST00000068237.6	MLX interacting protein
973	Adcy9	ENSMUST00000117801.2	adenylate cyclase 9
974	Slc7a8	ENSMUST00000022787.6	solute carrier family 7 (cationic amino acid transporter, y+ system), member 8
975	Slc22a23	ENSMUST00000040336.6	solute carrier family 22, member 23
976	Ndrg1	ENSMUST00000005256.7	N-myc downstream regulated gene 1
977	Nfic	ENSMUST00000020461.9	nuclear factor I/C
978	Ralgapb	ENSMUST00000109486.3	Ral GTPase activating protein, beta subunit (non-catalytic)
979	Gtpbp1	ENSMUST00000046463.8	GTP binding protein 1
980	Rybp	ENSMUST00000101118.2	RING1 and YY1 binding protein
981	Tenm4	ENSMUST00000107162.2	teneurin transmembrane protein 4
982	Smpd3	ENSMUST00000067512.7	sphingomyelin phosphodiesterase 3, neutral
983	Rxra	ENSMUST00000077257.6	retinoid X receptor alpha
984	Sp4	ENSMUST00000026367.9	trans-acting transcription factor 4
985	Fbxo41	ENSMUST00000159062.2	F-box protein 41
986	Grasp	ENSMUST00000000543.4	GRP1 (general receptor for phosphoinositides 1)-associated scaffold protein
987	Nfatc3	ENSMUST00000109308.1	nuclear factor of activated T cells, cytoplasmic, calcineurin dependent 3
988	Prkca	ENSMUST00000059595.5	protein kinase C, alpha
989	March6	ENSMUST00000090227.4	membrane-associated ring finger (C3HC4) 6
990	Trip12	ENSMUST00000186465.1	thyroid hormone receptor interactor 12
991	Sema6d	ENSMUST00000103239.4	sema domain, transmembrane domain (TM), and cytoplasmic domain, (semaphorin) 6D
992	Jak1	ENSMUST00000102781.4	Janus kinase 1
993	Phldb1	ENSMUST00000147495.2	pleckstrin homology-like domain, family B, member 1
994	Rere	ENSMUST00000105682.3	arginine glutamic acid dipeptide (RE) repeats
995	Ccnt2	ENSMUST00000112570.1	cyclin T2
996	Gpc6	ENSMUST00000078849.5	glypican 6
997	Atp11a	ENSMUST00000033818.4	ATPase, class VI, type 11A
998	Polr3g	ENSMUST00000048993.6	polymerase (RNA) III (DNA directed) polypeptide G
999	Gls	ENSMUST00000114510.2	glutaminase
1000	Zbtb7a	ENSMUST00000048128.9	zinc finger and BTB domain containing 7a
1001	Pip4k2b	ENSMUST00000018691.8	phosphatidylinositol-5-phosphate 4-kinase, type II, beta
1002	Ppp2r5d	ENSMUST00000002839.8	protein phosphatase 2, regulatory subunit B', delta
1003	Rcan2	ENSMUST00000044895.6	regulator of calcineurin 2
1004	Hpse2	ENSMUST00000099428.3	heparanase 2
1005	Amotl1	ENSMUST00000160770.2	angiomotin-like 1
1006	Rhobtb1	ENSMUST00000020101.6	Rho-related BTB domain containing 1
1007	Prune	ENSMUST00000015855.7	prune homolog (Drosophila)
1008	Zfp365	ENSMUST00000064656.7	zinc finger protein 365
1009	Marcks	ENSMUST00000092584.5	myristoylated alanine rich protein kinase C substrate
1010	Synj1	ENSMUST00000170853.2	synaptojanin 1
1011	Usp31	ENSMUST00000046929.6	ubiquitin specific peptidase 31
1012	Git2	ENSMUST00000112185.3	G protein-coupled receptor kinase-interactor 2
1013	Phactr1	ENSMUST00000148891.2	phosphatase and actin regulator 1
1014	Ubn1	ENSMUST00000052449.5	ubinuclein 1
1015	Ralgps2	ENSMUST00000172057.2	Ral GEF with PH domain and SH3 binding motif 2
1016	Podxl	ENSMUST00000026698.7	podocalyxin-like
1017	Smarca5	ENSMUST00000043359.8	SWI/SNF related, matrix associated, actin dependent regulator of chromatin, subfamily a, member 5
1018	Tenm1	ENSMUST00000115059.2	teneurin transmembrane protein 1
1019	Zbtb26	ENSMUST00000067043.4	zinc finger and BTB domain containing 26
1020	Zfhx4	ENSMUST00000176383.2	zinc finger homeodomain 4
1021	Gfpt1	ENSMUST00000113658.2	glutamine fructose-6-phosphate transaminase 1

1022	Map1a	ENSMUST00000094639.4	microtubule-associated protein 1 A
1023	P2ry4	ENSMUST00000053373.1	pyrimidinergic receptor P2Y, G-protein coupled, 4
1024	Ptpn14	ENSMUST00000027898.5	protein tyrosine phosphatase, non-receptor type 14
1025	Mmp15	ENSMUST00000034243.5	matrix metallopeptidase 15
1026	Rest	ENSMUST00000080359.6	RE1-silencing transcription factor
1027	Arcn1	ENSMUST00000034607.9	archain 1
1028	Btg2	ENSMUST00000020692.6	B cell translocation gene 2, anti-proliferative
1029	Slitrk4	ENSMUST00000069926.8	SLIT and NTRK-like family, member 4
1030	Ccr6	ENSMUST00000164411.3	chemokine (C-C motif) receptor 6
1031	4932411E22Rik	ENSMUST00000050983.1	RIKEN cDNA 4932411E22 gene
1032	Kcnj3	ENSMUST00000112632.1	potassium inwardly-rectifying channel, subfamily J, member 3
1033	Son	ENSMUST00000122302.2	Son DNA binding protein
1034	Ctdp1	ENSMUST00000036229.7	CTD (carboxy-terminal domain, RNA polymerase II, polypeptide A) phosphatase, subunit 1
1035	Nacc1	ENSMUST00000001975.4	nucleus accumbens associated 1, BEN and BTB (POZ) domain containing
1036	Ptprk	ENSMUST00000166468.1	protein tyrosine phosphatase, receptor type, K
1037	Eml5	ENSMUST00000065716.6	echinoderm microtubule associated protein like 5
1038	Clmn	ENSMUST00000109937.2	calmin
1039	Nelfa	ENSMUST00000030993.6	negative elongation factor complex member A, Whsc2
1040	Yap1	ENSMUST00000086580.6	yes-associated protein 1
1041	C2cd2	ENSMUST00000170757.1	C2 calcium-dependent domain containing 2
1042	Hspa13	ENSMUST00000114244.1	heat shock protein 70 family, member 13
1043	Fryl	ENSMUST00000101127.6	furry homolog-like (Drosophila)
1044	Tspan15	ENSMUST00000047883.9	tetraspanin 15
1045	Gm28048	ENSMUST00000160826.1	predicted gene, 28048
1046	Pdcd2	ENSMUST00000054450.6	programmed cell death 2
1047	Srgap3	ENSMUST00000088373.5	SLIT-ROBO Rho GTPase activating protein 3
1048	Sgms2	ENSMUST00000090246.4	sphingomyelin synthase 2
1049	Amotl2	ENSMUST00000035121.8	angiomotin-like 2
1050	Pclo	ENSMUST00000182407.2	piccolo (presynaptic cytomatrix protein)
1051	Cacna1e	ENSMUST00000187541.1	calcium channel, voltage-dependent, R type, alpha 1E subunit
1052	Gramd4	ENSMUST00000138134.2	GRAM domain containing 4
1053	Szrd1	ENSMUST00000102487.3	SUZ RNA binding domain containing 1
1054	Slc1a1	ENSMUST00000025875.4	solute carrier family 1 (neuronal/epithelial high affinity glutamate transporter, system Xag), member 1
1055	Ptch1	ENSMUST00000021921.5	patched homolog 1
1056	Pitpnm2	ENSMUST00000162812.2	phosphatidylinositol transfer protein, membrane-associated 2
1057	Wnt4	ENSMUST00000045747.4	wingless-related MMTV integration site 4
1058	Slc36a1	ENSMUST00000108872.3	solute carrier family 36 (proton/amino acid symporter), member 1
1059	Drd2	ENSMUST00000075764.6	dopamine receptor D2
1060	Sik1	ENSMUST00000024839.4	salt inducible kinase 1
1061	Plekha6	ENSMUST00000105082.3	pleckstrin homology domain containing, family A member 6
1062	Baz2b	ENSMUST00000112550.2	bromodomain adjacent to zinc finger domain, 2B
1063	Elavl2	ENSMUST00000107110.2	ELAV (embryonic lethal, abnormal vision, Drosophila)-like 2 (Hu antigen B)
1064	Elavl4	ENSMUST00000106598.2	ELAV (embryonic lethal, abnormal vision, Drosophila)-like 4 (Hu antigen D)
1065	Zbtb7c	ENSMUST00000058997.8	zinc finger and BTB domain containing 7C
1066	Flrt1	ENSMUST00000113383.2	fibronectin leucine rich transmembrane protein 1
1067	Kdm7a	ENSMUST00000002305.8	lysine (K)-specific demethylase 7A
1068	Slc45a4	ENSMUST00000151288.2	solute carrier family 45, member 4
1069	Fbxl17	ENSMUST00000024761.7	F-box and leucine-rich repeat protein 17
1070	Mllt3	ENSMUST00000078090.6	myeloid/lymphoid or mixed-lineage leukemia (trithorax homolog, Drosophila); translocated to, 3
1071	Dcbld2	ENSMUST00000046663.7	discoidin, CUB and LCCL domain containing 2

1072	Scube1	ENSMUST00000171496.2	signal peptide, CUB domain, EGF-like 1
1073	Dcp1b	ENSMUST00000112777.3	DCP1 decapping enzyme homolog B (S. cerevisiae)
1074	Nedd1	ENSMUST00000020163.6	neural precursor cell expressed, developmentally down-regulated gene 1
1075	E130308A19Rik	ENSMUST00000070150.5	RIKEN cDNA E130308A19 gene
1076	Ncoa2	ENSMUST00000081713.5	nuclear receptor coactivator 2
1077	Lphn1	ENSMUST00000141158.2	latrophilin 1
1078	Slc24a2	ENSMUST00000107157.3	solute carrier family 24 (sodium/potassium/calcium exchanger), member 2
1079	Bbs1	ENSMUST00000053506.6	Bardet-Biedl syndrome 1 (human)
1080	Pcnx	ENSMUST00000021567.5	pecanex homolog (Drosophila)
1081	Gucy1a2	ENSMUST00000115733.1	guanylate cyclase 1, soluble, alpha 2
1082	Ttbk2	ENSMUST00000057135.8	tau tubulin kinase 2
1083	Tfrc	ENSMUST00000023486.9	transferrin receptor
1084	Pak3	ENSMUST00000172330.2	p21 protein (Cdc42/Rac)-activated kinase 3
1085	Rai14	ENSMUST00000090339.4	retinoic acid induced 14
1086	D430041D05Rik	ENSMUST00000089726.4	RIKEN cDNA D430041D05 gene
1087	Pcgf5	ENSMUST00000071267.7	polycomb group ring finger 5
1088	Scube3	ENSMUST00000043503.4	signal peptide, CUB domain, EGF-like 3
1089	Cnih4	ENSMUST00000134115.2	cornichon homolog 4 (Drosophila)
1090	Zdhhc23	ENSMUST00000036321.8	zinc finger, DHHC domain containing 23
1091	Gemin5	ENSMUST00000172035.2	gem (nuclear organelle) associated protein 5
1092	Kif26b	ENSMUST00000160789.1	kinesin family member 26B
1093	Dcp1a	ENSMUST00000022535.7	DCP1 decapping enzyme homolog A (S. cerevisiae)
1094	Jade2	ENSMUST00000109090.2	jade family PHD finger 2
1095	Tmem110	ENSMUST00000006701.5	transmembrane protein 110
1096	Cep350	ENSMUST00000138762.2	centrosomal protein 350
1097	Agfg2	ENSMUST00000031736.10	ArfGAP with FG repeats 2
1098	Cacnb2	ENSMUST00000114723.3	calcium channel, voltage-dependent, beta 2 subunit
1099	Sh3glb1	ENSMUST00000163279.1	SH3-domain GRB2-like B1 (endophilin)
1100	Map1b	ENSMUST00000064762.4	microtubule-associated protein 1B
1101	Arhgap31	ENSMUST00000023487.4	Rho GTPase activating protein 31
1102	Smek1	ENSMUST00000163095.2	SMEK homolog 1, suppressor of mek1 (Dictyostelium)
1103	Clcn6	ENSMUST00000137724.2	chloride channel 6
1104	Kcng3	ENSMUST00000051482.1	potassium voltage-gated channel, subfamily G, member 3
1105	Usp15	ENSMUST00000020334.7	ubiquitin specific peptidase 15
1106	Rims3	ENSMUST00000071093.3	regulating synaptic membrane exocytosis 3
1107	Unc80	ENSMUST00000061620.10	unc-80 homolog (C. elegans)
1108	Ptprt	ENSMUST00000109445.3	protein tyrosine phosphatase, receptor type, T
1109	Gpr153	ENSMUST00000105650.2	G protein-coupled receptor 153
1110	Kcnh1	ENSMUST00000078470.6	potassium voltage-gated channel, subfamily H (eag-related), member 1
1111	Srgn	ENSMUST00000160987.2	serglycin
1112	Smad4	ENSMUST00000025393.8	SMAD family member 4
1113	Shroom2	ENSMUST00000101141.3	shroom family member 2
1114	Ildr2	ENSMUST00000111416.1	immunoglobulin-like domain containing receptor 2
1115	Smcr8	ENSMUST00000056907.6	Smith-Magenis syndrome chromosome region, candidate 8 homolog (human)
1116	Gpalpp1	ENSMUST00000022585.3	GPALPP motifs containing 1
1117	Kcnb1	ENSMUST00000059826.8	potassium voltage gated channel, Shab-related subfamily, member 1
1118	Hdac5	ENSMUST00000008999.6	histone deacetylase 5
1119	Senp6	ENSMUST00000037484.9	SUMO/sentrin specific peptidase 6
1120	Ip6k1	ENSMUST00000035214.5	inositol hexaphosphate kinase 1
1121	Unc13a	ENSMUST00000030170.9	unc-13 homolog A (C. elegans)
1122	Taf1d	ENSMUST00000164079.2	TATA box binding protein (Tbp)-associated factor, RNA polymerase I, D
1123	Rfxank	ENSMUST00000075724.7	regulatory factor X-associated ankyrin-containing protein
1124	Cdk13	ENSMUST00000042365.7	cyclin-dependent kinase 13

1125	Zfp618	ENSMUST00000107415.2	zinc finger protein 618
1126	Rasa2	ENSMUST00000034984.7	RAS p21 protein activator 2
1127	Plekhm1	ENSMUST00000041272.9	pleckstrin homology domain containing, family M (with RUN domain) member 1
1128	Mnt	ENSMUST00000000291.3	max binding protein
1129	Nhlrc2	ENSMUST00000071423.5	NHL repeat containing 2
1130	Xylt1	ENSMUST00000032892.5	xylosyltransferase 1
1131	Sh3bp4	ENSMUST00000066279.5	SH3-domain binding protein 4
1132	Ptar1	ENSMUST00000099560.3	protein prenyltransferase alpha subunit repeat containing 1
1133	Cdh11	ENSMUST00000075190.3	cadherin 11
1134	Pcm1	ENSMUST00000045218.7	pericentriolar material 1
1135	D15Ertd621e	ENSMUST00000037270.3	DNA segment, Chr 15, ERATO Doi 621, expressed
1136	Nav1	ENSMUST00000067414.7	neuron navigator 1
1137	Ptgfrn	ENSMUST00000102694.3	prostaglandin F2 receptor negative regulator
1138	Pitpnc1	ENSMUST00000103064.4	phosphatidylinositol transfer protein, cytoplasmic 1
1139	Kcnn3	ENSMUST00000000811.7	potassium intermediate/small conductance calcium-activated channel, subfamily N, member 3
1140	Ppp2r2a	ENSMUST00000089230.5	protein phosphatase 2, regulatory subunit B, alpha
1141	Bag4	ENSMUST00000038498.8	BCL2-associated athanogene 4
1142	Dnaja4	ENSMUST00000070070.7	DnaJ (Hsp40) homolog, subfamily A, member 4
1143	Gtpbp2	ENSMUST00000024748.8	GTP binding protein 2
1144	Notch1	ENSMUST00000028288.4	notch 1
1145	Rfx5	ENSMUST00000107254.2	regulatory factor X, 5 (influences HLA class II expression)
1146	Tfdp2	ENSMUST00000034982.10	transcription factor Dp 2
1147	Enah	ENSMUST00000078719.7	enabled homolog (Drosophila)
1148	Egr3	ENSMUST00000035908.1	early growth response 3
1149	Arhgap39	ENSMUST00000036176.9	Rho GTPase activating protein 39
1150	Fam135b	ENSMUST00000022953.8	family with sequence similarity 135, member B
1151	Otud4	ENSMUST00000173078.2	OTU domain containing 4
1152	Tmem33	ENSMUST00000037918.6	transmembrane protein 33
1153	1110002E22Rik	ENSMUST00000163080.2	RIKEN cDNA 1110002E22 gene
1154	Slc7a11	ENSMUST00000029297.4	solute carrier family 7 (cationic amino acid transporter, y+ system), member 11
1155	Tnrc6b	ENSMUST00000067689.7	trinucleotide repeat containing 6b
1156	Tmod3	ENSMUST00000072232.7	tropomodulin 3
1157	Plxna4	ENSMUST00000115096.3	plexin A4
1158	Ebf1	ENSMUST00000081265.6	early B cell factor 1
1159	Rap2a	ENSMUST00000062117.7	RAS related protein 2a
1160	Lix1l	ENSMUST00000062058.3	Lix1-like
1161	Gch1	ENSMUST00000089959.6	GTP cyclohydrolase 1
1162	Grin2a	ENSMUST00000115835.2	glutamate receptor, ionotropic, NMDA2A (epsilon 1)
1163	Itm2b	ENSMUST00000022704.7	integral membrane protein 2B
1164	Unc5d	ENSMUST00000168630.2	unc-5 homolog D (C. elegans)
1165	Mcmbp	ENSMUST00000057557.8	MCM (minichromosome maintenance deficient) binding protein
1166	Ext1	ENSMUST00000077273.3	exostoses (multiple) 1
1167	Smarce1	ENSMUST00000103133.3	SWI/SNF related, matrix associated, actin dependent regulator of chromatin, subfamily e, member 1
1168	Gapvd1	ENSMUST00000102800.2	GTPase activating protein and VPS9 domains 1
1169	Klhdc10	ENSMUST00000068259.6	kelch domain containing 10
1170	Robo4	ENSMUST00000115048.2	roundabout homolog 4 (Drosophila)
1171	Apba1	ENSMUST00000025830.7	amyloid beta (A4) precursor protein binding, family A, member 1
1172	Nploc4	ENSMUST00000044271.9	nuclear protein localization 4 homolog (S. cerevisiae)
1173	Sorbs3	ENSMUST00000022682.5	sorbin and SH3 domain containing 3
1174	Efna5	ENSMUST00000076840.6	ephrin A5
1175	Cisd3	ENSMUST00000107584.2	CDGSH iron sulfur domain 3
1176	Camta1	ENSMUST00000097774.3	calmodulin binding transcription activator 1

1177	Arfgef2	ENSMUST00000099078.4	ADP-ribosylation factor guanine nucleotide-exchange factor 2 (brefeldin A-inhibited)
1178	Ggcx	ENSMUST00000065906.7	gamma-glutamyl carboxylase
1179	Tmem212	ENSMUST00000058077.3	transmembrane protein 212
1180	Tox2	ENSMUST00000109428.3	TOX high mobility group box family member 2
1181	Nr1i3	ENSMUST00000155126.2	nuclear receptor subfamily 1, group I, member 3
1182	Rnf139	ENSMUST00000036904.6	ring finger protein 139
1183	Cep170b	ENSMUST00000101018.4	centrosomal protein 170B
1184	Dicer1	ENSMUST00000041987.6	dicer 1, ribonuclease type III
1185	Fastk	ENSMUST00000115043.2	Fas-activated serine/threonine kinase
1186	Smim20	ENSMUST00000121042.2	small integral membrane protein 20
1187	Tbck	ENSMUST00000169172.2	TBC1 domain containing kinase
1188	Extl3	ENSMUST00000022550.7	exostoses (multiple)-like 3
1189	Shank2	ENSMUST00000105902.2	SH3/ankyrin domain gene 2
1190	Fbxo28	ENSMUST00000051431.4	F-box protein 28
1191	Pja2	ENSMUST00000172818.2	praja 2, RING-H2 motif containing
1192	Memo1	ENSMUST00000078459.6	mediator of cell motility 1
1193	Psd3	ENSMUST00000038959.10	pleckstrin and Sec7 domain containing 3
1194	Rps6ka6	ENSMUST00000065976.6	ribosomal protein S6 kinase polypeptide 6
1195	Reep3	ENSMUST00000020023.7	receptor accessory protein 3
1196	Gpc4	ENSMUST00000033450.2	glypican 4
1197	Bcl9l	ENSMUST00000074989.5	B cell CLL/lymphoma 9-like
1198	Obfc1	ENSMUST00000049369.10	oligonucleotide/oligosaccharide-binding fold containing 1
1199	Xrcc2	ENSMUST00000030773.7	X-ray repair complementing defective repair in Chinese hamster cells 2
1200	Fbxl19	ENSMUST00000186116.1	F-box and leucine-rich repeat protein 19
1201	D630045J12Rik	ENSMUST00000117556.2	RIKEN cDNA D630045J12 gene
1202	Cpsf6	ENSMUST00000176686.2	cleavage and polyadenylation specific factor 6
1203	Ahdc1	ENSMUST00000105916.2	AT hook, DNA binding motif, containing 1
1204	Zdhhc3	ENSMUST00000147563.2	zinc finger, DHHC domain containing 3
1205	Khdrbs1	ENSMUST00000066257.5	KH domain containing, RNA binding, signal transduction associated 1
1206	Fnbp1	ENSMUST00000100208.3	formin binding protein 1
1207	Shank1	ENSMUST00000107938.2	SH3/ankyrin domain gene 1
1208	Sptbn2	ENSMUST00000008991.6	spectrin beta, non-erythrocytic 2
1209	Sdccag8	ENSMUST00000027785.9	serologically defined colon cancer antigen 8
1210	Myf6	ENSMUST00000044210.3	myogenic factor 6
1211	Zfp282	ENSMUST00000061890.7	zinc finger protein 282
1212	Zeb2	ENSMUST00000068415.5	zinc finger E-box binding homeobox 2
1213	Ephb2	ENSMUST00000105845.3	Eph receptor B2
1214	Traf4	ENSMUST00000017530.3	TNF receptor associated factor 4
1215	Igf2bp1	ENSMUST00000013559.2	insulin-like growth factor 2 mRNA binding protein 1
1216	Tnrc6a	ENSMUST00000094053.5	trinucleotide repeat containing 6a
1217	Nkiras1	ENSMUST00000132374.2	NFKB inhibitor interacting Ras-like protein 1
1218	Swt1	ENSMUST00000064771.6	SWT1 RNA endoribonuclease homolog (S. cerevisiae)
1219	Slc6a20b	ENSMUST00000026273.8	solute carrier family 6 (neurotransmitter transporter), member 20B
1220	Tmem53	ENSMUST00000106434.2	transmembrane protein 53
1221	Wwp2	ENSMUST00000166615.1	WW domain containing E3 ubiquitin protein ligase 2
1222	Adrb3	ENSMUST00000081438.4	adrenergic receptor, beta 3
1223	Prss12	ENSMUST00000029603.8	protease, serine 12 neurotrypsin (motopsin)
1224	Poldip2	ENSMUST00000001127.5	polymerase (DNA-directed), delta interacting protein 2
1225	Rtf1	ENSMUST00000028767.8	Rtf1, Paf1/RNA polymerase II complex component, homolog (S. cerevisiae)
1226	Slc30a5	ENSMUST00000067246.4	solute carrier family 30 (zinc transporter), member 5
1227	Dlgap2	ENSMUST00000133298.2	discs, large (Drosophila) homolog-associated protein 2
1228	Efnb1	ENSMUST00000052839.6	ephrin B1
1229	Htr1f	ENSMUST00000063076.4	5-hydroxytryptamine (serotonin) receptor 1F
1230	Slc44a1	ENSMUST00000107651.3	solute carrier family 44, member 1

1231	Slitrk3	ENSMUST00000059407.7	SLIT and NTRK-like family, member 3
1232	Wscd1	ENSMUST00000021168.8	WSC domain containing 1
1233	Pddc1	ENSMUST00000106008.1	Parkinson disease 7 domain containing 1
1234	Nop9	ENSMUST00000019441.8	NOP9 nucleolar protein
1235	Syt10	ENSMUST00000029441.3	synaptotagmin X
1236	Upp1	ENSMUST00000101525.3	uridine phosphorylase 1
1237	Dcc	ENSMUST00000114943.4	deleted in colorectal carcinoma
1238	Otud7a	ENSMUST00000058476.8	OTU domain containing 7A
1239	Psmb4	ENSMUST00000005923.6	proteasome (prosome, macropain) subunit, beta type 4
1240	Calml4	ENSMUST00000034777.7	calmodulin-like 4
1241	Dync1i2	ENSMUST00000112140.2	dynein cytoplasmic 1 intermediate chain 2
1242	Sptlc1	ENSMUST00000021920.6	serine palmitoyltransferase, long chain base subunit 1
