# Supplementary material for: miR-9 utilizes precursor pathways in adaptation to alcohol in mouse striatal neurons
Source: Adv Drug Alcohol Res. Author manuscript; Available in PMC 2023 Dec 19. (PMC10730111; doi:10.3389/adar.2023.11323)
Supplement: Mead et al SM Table 2 [file NIHMS1906815-supplement-Mead_et_al_SM_Table_2.pdf]

Supplementary Materials (SM), Table 2
List of murine miR-9-3p predicted targets by TargetScan 7.1

#	Target gene	Representative transcript	Gene name
1	Onecut2	ENSMUST00000175965.3	one cut domain, family member 2
2	Ttll7	ENSMUST00000170055.2	tubulin tyrosine ligase-like family, member 7
3	Mtx3	ENSMUST00000076169.3	metaxin 3
4	Mpeg1	ENSMUST00000081035.7	macrophage expressed gene 1
5	Hs6st1	ENSMUST00000088174.3	heparan sulfate 6-O-sulfotransferase 1
6	Dmxl1	ENSMUST00000041772.5	Dmx-like 1
7	Pgr	ENSMUST00000189181.1	progesterone receptor
8	Stag2	ENSMUST00000115073.3	stromal antigen 2
9	Gen1	ENSMUST00000166117.1	Gen homolog 1, endonuclease (Drosophila)
10	D830030K20Rik	ENSMUST00000178670.1	RIKEN cDNA D830030K20 gene
11	Lcor	ENSMUST00000067795.6	ligand dependent nuclear receptor corepressor
12	Gns	ENSMUST00000040344.6	glucosamine (N-acetyl)-6-sulfatase
13	Rnaset2b	ENSMUST00000179728.1	ribonuclease T2B
14	Tmem236	ENSMUST00000077517.7	transmembrane protein 236
15	Npepps	ENSMUST00000001480.8	aminopeptidase puromycin sensitive
16	Agfg1	ENSMUST00000189220.1	ArfGAP with FG repeats 1
17	Iqcb1	ENSMUST00000114819.2	IQ calmodulin-binding motif containing 1
18	AI118078	ENSMUST00000085754.4	expressed sequence AI118078
19	Stim2	ENSMUST00000117661.3	stromal interaction molecule 2
20	Psma8	ENSMUST00000040860.1	proteasome (prosome, macropain) subunit, alpha type, 8
21	E130309F12Rik	ENSMUST00000076670.2	RIKEN cDNA E130309F12 gene
22	Tbc1d15	ENSMUST00000020339.8	TBC1 domain family, member 15
23	Nipal1	ENSMUST00000087212.3	NIPA-like domain containing 1
24	Myo10	ENSMUST00000110457.2	myosin X
25	Heyl	ENSMUST00000040821.4	hairy/enhancer-of-split related with YRPW motif-like
26	Zfp952	ENSMUST00000087666.5	zinc finger protein 952
27	Tet3	ENSMUST00000089622.5	tet methylcytosine dioxygenase 3
28	Gm5819	ENSMUST00000171469.1	predicted gene 5819
29	Zfp113	ENSMUST00000049393.9	zinc finger protein 113
30	Vldlr	ENSMUST00000167487.2	very low density lipoprotein receptor
31	Rhobtb3	ENSMUST00000022078.6	Rho-related BTB domain containing 3
32	Pard3b	ENSMUST00000075374.4	par-3 family cell polarity regulator beta
33	Tmem194b	ENSMUST00000165859.2	transmembrane protein 194B
34	Cntnap5a	ENSMUST00000043725.8	contactin associated protein-like 5A
35	Cpne9	ENSMUST00000041203.5	copine family member IX
36	Cggbp1	ENSMUST00000067744.7	CGG triplet repeat binding protein 1
37	Plekha5	ENSMUST00000087622.3	pleckstrin homology domain containing, family A member 5
38	BC068281	ENSMUST00000085793.2	cDNA sequence BC068281
39	Carf	ENSMUST00000187978.1	calcium response factor
40	Crk	ENSMUST00000017920.8	v-crk sarcoma virus CT10 oncogene homolog (avian)
41	Zfp141	ENSMUST00000174407.1	zinc finger protein 141
42	Dmd	ENSMUST00000114000.2	dystrophin, muscular dystrophy
43	Fzd7	ENSMUST00000114246.3	frizzled homolog 7 (Drosophila)
44	Esm1	ENSMUST00000038144.8	endothelial cell-specific molecule 1
45	Pth	ENSMUST00000079793.5	parathyroid hormone
46	Ern1	ENSMUST00000001059.3	endoplasmic reticulum (ER) to nucleus signalling 1

47	Rtp1	ENSMUST00000038730.6	receptor transporter protein 1
48	Gypc	ENSMUST00000174459.1	glycophorin C
49	Purb	ENSMUST00000179343.2	purine rich element binding protein B
50	Mnd1	ENSMUST00000047368.6	meiotic nuclear divisions 1 homolog (S. cerevisiae)
51	Dnajc27	ENSMUST00000020986.8	DnaJ (Hsp40) homolog, subfamily C, member 27
52	Pygo1	ENSMUST00000038489.5	pygopus 1
53	Papd5	ENSMUST00000119033.2	PAP associated domain containing 5
54	Noxa1	ENSMUST00000114373.2	NADPH oxidase activator 1
55	Pdcl	ENSMUST00000009174.9	phosducin-like
56	Cd59a	ENSMUST00000040423.6	CD59a antigen
57	Zfp174	ENSMUST00000041778.7	zinc finger protein 174
58	Chsy3	ENSMUST00000080721.4	chondroitin sulfate synthase 3
59	Ccdc117	ENSMUST00000020776.4	coiled-coil domain containing 117
60	Map7d2	ENSMUST00000112471.3	MAP7 domain containing 2
61	H2-M3	ENSMUST00000038580.6	histocompatibility 2, M region locus 3
62	Fuom	ENSMUST00000026539.8	fucose mutarotase
63	Iah1	ENSMUST00000076813.6	isoamyl acetate-hydrolyzing esterase 1 homolog (S. cerevisiae)
64	Pdcd1	ENSMUST00000027507.7	programmed cell death 1
65	Nxt2	ENSMUST00000112916.3	nuclear transport factor 2-like export factor 2
66	Plag1	ENSMUST00000003369.4	pleiomorphic adenoma gene 1
67	Rgs10	ENSMUST00000033133.6	regulator of G-protein signalling 10
68	Sav1	ENSMUST00000021467.7	salvador homolog 1 (Drosophila)
69	Hoxa10	ENSMUST00000125581.1	homeobox A10
70	Ect2	ENSMUST00000108300.2	ect2 oncogene
71	Ndnl2	ENSMUST00000094331.2	necdin-like 2
72	Fam188a	ENSMUST00000028105.7	family with sequence similarity 188, member A
73	Fubp1	ENSMUST00000166984.2	far upstream element (FUSE) binding protein 1
74	4930550L24Rik	ENSMUST00000118305.2	RIKEN cDNA 4930550L24 gene
75	Gpr174	ENSMUST00000178838.1	G protein-coupled receptor 174
76	Igfbp7	ENSMUST00000163898.2	insulin-like growth factor binding protein 7
77	Tmem64	ENSMUST00000062684.8	transmembrane protein 64
78	Cpa3	ENSMUST00000001921.1	carboxypeptidase A3, mast cell
79	Zbtb42	ENSMUST00000174780.1	zinc finger and BTB domain containing 42
80	Zfp266	ENSMUST00000174462.2	zinc finger protein 266
81	Naa20	ENSMUST00000110000.2	N(alpha)-acetyltransferase 20, NatB catalytic subunit
82	Kpna7	ENSMUST00000110673.2	karyopherin alpha 7 (importin alpha 8)
83	Cntnap5c	ENSMUST00000076038.5	contactin associated protein-like 5C
84	Sypl	ENSMUST00000076698.6	synaptophysin-like protein
85	Gm3033	ENSMUST00000112792.3	predicted gene 3033
86	Gm11007	ENSMUST00000178120.2	predicted gene 11007
87	Gm2007	ENSMUST00000178133.2	predicted gene 2007
88	Pdcd4	ENSMUST00000074371.6	programmed cell death 4
89	Cxcl3	ENSMUST00000031326.8	chemokine (C-X-C motif) ligand 3
90	Etl4	ENSMUST00000114606.2	enhancer trap locus 4
91	Pyroxd1	ENSMUST00000041852.7	pyridine nucleotide-disulphide oxidoreductase domain 1
92	Mamld1	ENSMUST00000082088.4	mastermind-like domain containing 1
93	Olfr70	ENSMUST00000055545.3	olfactory receptor 70
94	Egfr	ENSMUST00000020329.7	epidermal growth factor receptor
95	Gcnt2	ENSMUST00000110191.3	glucosaminyl (N-acetyl) transferase 2, I-branching enzyme

96	Rgn	ENSMUST00000023832.6	regucalcin
97	Tyw5	ENSMUST00000162686.2	tRNA-yW synthesizing protein 5
98	Akr1c13	ENSMUST00000021634.2	aldo-keto reductase family 1, member C13
99	Dos	ENSMUST00000170219.3	downstream of Stk11
100	Mgl2	ENSMUST00000041550.6	macrophage galactose N-acetyl-galactosamine specific lectin 2
101	Ap3m1	ENSMUST00000154460.2	adaptor-related protein complex 3, mu 1 subunit
102	Usp15	ENSMUST00000020334.7	ubiquitin specific peptidase 15
103	Ccnd2	ENSMUST00000000188.8	cyclin D2
104	H2afz	ENSMUST00000173790.2	H2A histone family, member Z
105	Il17d	ENSMUST00000089494.4	interleukin 17D
106	Ddx5	ENSMUST00000021062.6	DEAD (Asp-Glu-Ala-Asp) box polypeptide 5
107	Palm2Akap2	ENSMUST00000150412.1	Palm2-Akap2 readthrough transcript
108	Gm10228	ENSMUST00000089105.2	predicted gene 10228
109	Gm10083	ENSMUST00000074466.5	predicted gene 10083
110	Dmrtc1a	ENSMUST00000120808.2	DMRT-like family C1a
111	Slc5a3	ENSMUST00000113975.2	solute carrier family 5 (inositol transporters), member 3
112	Sema4a	ENSMUST00000029700.6	sema domain, immunoglobulin domain (Ig), transmembrane domain (TM) and short cytoplasmic domain, (semaphorin) 4A
113	Klhl34	ENSMUST00000087157.4	kelch-like 34
114	Zfp616	ENSMUST00000178159.1	zinc finger protein 616
115	D10Jhu81e	ENSMUST00000001242.7	DNA segment, Chr 10, Johns Hopkins University 81 expressed
116	Nfxl1	ENSMUST00000087216.6	nuclear transcription factor, X-box binding-like 1
117	Klhl15	ENSMUST00000113915.1	kelch-like 15
118	Gm10109	ENSMUST00000072123.4	predicted gene 10109
119	Stbd1	ENSMUST00000050952.3	starch binding domain 1
120	Mtmr12	ENSMUST00000038172.9	myotubularin related protein 12
121	Glp1r	ENSMUST00000114574.1	glucagon-like peptide 1 receptor
122	Wasf3	ENSMUST00000016143.7	WAS protein family, member 3
123	Gnpnat1	ENSMUST00000046191.7	glucosamine-phosphate N-acetyltransferase 1
124	Magea2	ENSMUST00000069477.2	melanoma antigen, family A, 2
125	4833413E03Rik	ENSMUST00000160270.1	RIKEN cDNA 4833413E03 gene
126	Mpped2	ENSMUST00000111063.2	metallophosphoesterase domain containing 2
127	Gm3532	ENSMUST00000181880.1	predicted gene 3532
128	Dazl	ENSMUST00000010736.7	deleted in azoospermia-like
129	Fzd3	ENSMUST00000131309.1	frizzled homolog 3 (Drosophila)
130	Rpl17	ENSMUST00000079716.5	ribosomal protein L17
131	Gm266	ENSMUST00000010673.5	predicted gene 266
132	Dr1	ENSMUST00000031190.4	down-regulator of transcription 1
133	Rnf141	ENSMUST00000177236.2	ring finger protein 141
134	Churc1	ENSMUST00000110399.2	churchill domain containing 1
135	Gm8271	ENSMUST00000171053.1	predicted gene 8271
136	A1cf	ENSMUST00000075838.5	APOBEC1 complementation factor
137	Slc5a7	ENSMUST00000095712.3	solute carrier family 5 (choline transporter), member 7
138	Gm3755	ENSMUST00000169555.1	predicted gene 3755
139	Gm2244	ENSMUST00000164372.1	predicted gene 2244
140	Gm3278	ENSMUST00000164459.1	predicted gene 3278
141	Bpifb3	ENSMUST00000088950.2	BPI fold containing family B, member 3
142	Gm10408	ENSMUST00000096168.5	predicted gene 10408

143	Gm8050	ENSMUST00000171987.1	predicted gene 8050
144	Ncoa3	ENSMUST00000088095.5	nuclear receptor coactivator 3
145	Peli1	ENSMUST00000093290.6	pellino 1
146	Foxh1	ENSMUST00000037824.4	forkhead box H1
147	Rai2	ENSMUST00000061514.7	retinoic acid induced 2
148	Pde4d	ENSMUST00000120664.2	phosphodiesterase 4D, cAMP specific
149	Jazf1	ENSMUST00000074541.5	JAZF zinc finger 1
150	Edc3	ENSMUST00000043990.8	enhancer of mRNA decapping 3 homolog (S. cerevisiae)
151	Dpy30	ENSMUST00000164832.2	dpy-30 homolog (C. elegans)
152	Pex13	ENSMUST00000020523.3	peroxisomal biogenesis factor 13
153	Rimkla	ENSMUST00000049994.7	ribosomal modification protein rimK-like family member A
154	Blmh	ENSMUST00000021197.4	bleomycin hydrolase
155	Adipor1	ENSMUST00000027727.9	adiponectin receptor 1
156	Pidd1	ENSMUST00000026580.6	p53 induced death domain protein 1
157	Eif4a2	ENSMUST00000077605.6	eukaryotic translation initiation factor 4A2
158	Prss56	ENSMUST00000044533.8	protease, serine 56
159	Tcea1	ENSMUST00000165720.2	transcription elongation factor A (SII) 1
160	Magix	ENSMUST00000115695.3	MAGI family member, X-linked
161	Ctr9	ENSMUST00000005749.5	Ctr9, Paf1/RNA polymerase II complex component, homolog (S. cerevisiae)
162	Pdcl2	ENSMUST00000122213.2	phosducin-like 2
163	Atp6ap1	ENSMUST00000114171.4	ATPase, H+ transporting, lysosomal accessory protein 1
164	AA792892	ENSMUST00000097477.3	expressed sequence AA792892
165	Zfhx3	ENSMUST00000043896.9	zinc finger homeobox 3
166	Pbk	ENSMUST00000022612.4	PDZ binding kinase
167	Efcab14	ENSMUST00000074425.7	EF-hand calcium binding domain 14
168	Kcnq5	ENSMUST00000029667.7	potassium voltage-gated channel, subfamily Q, member 5
169	Foxq1	ENSMUST00000042118.9	forkhead box Q1
170	Tcte2	ENSMUST00000130033.2	t-complex-associated testis expressed 2
171	Slc39a8	ENSMUST00000180196.2	solute carrier family 39 (metal ion transporter), member 8
172	Mad2l1	ENSMUST00000101343.1	MAD2 mitotic arrest deficient-like 1
173	Tuba1b	ENSMUST00000077577.7	tubulin, alpha 1B
174	Apol7c	ENSMUST00000062562.5	apolipoprotein L 7c
175	Klrb1b	ENSMUST00000032472.5	killer cell lectin-like receptor subfamily B member 1B
176	Brwd1	ENSMUST00000113829.2	bromodomain and WD repeat domain containing 1
177	Plce1	ENSMUST00000169713.3	phospholipase C, epsilon 1
178	4933411G11Rik	ENSMUST00000031574.4	RIKEN cDNA 4933411G11Rik gene
179	Caap1	ENSMUST00000030313.8	caspase activity and apoptosis inhibitor 1
180	Cdkn1c	ENSMUST00000167912.2	cyclin-dependent kinase inhibitor 1C (P57)
181	Chrnb3	ENSMUST00000079463.5	cholinergic receptor, nicotinic, beta polypeptide 3
182	Fgf14	ENSMUST00000095529.4	fibroblast growth factor 14
183	Immt	ENSMUST00000166975.2	inner membrane protein, mitochondrial
184	Oas1b	ENSMUST00000086377.3	2'-5' oligoadenylate synthetase 1B
185	Ncr1	ENSMUST00000006792.4	natural cytotoxicity triggering receptor 1
186	Ttll4	ENSMUST00000042125.9	tubulin tyrosine ligase-like family, member 4
187	Dhx15	ENSMUST00000031061.7	DEAH (Asp-Glu-Ala-His) box polypeptide 15

188	Gpalpp1	ENSMUST00000022585.3	GPALPP motifs containing 1
189	Triap1	ENSMUST00000031508.4	TP53 regulated inhibitor of apoptosis 1
190	Syngr2	ENSMUST00000177131.2	synaptogyrin 2
191	Xk	ENSMUST00000015486.6	Kell blood group precursor (McLeod phenotype) homolog
192	1600014C10Rik	ENSMUST00000165308.2	RIKEN cDNA 1600014C10 gene
193	P4ha2	ENSMUST00000174616.2	procollagen-proline, 2-oxoglutarate 4-dioxygenase (proline 4-hydroxylase), alpha II polypeptide
194	Sebox	ENSMUST00000001130.7	SEBOX homeobox
195	Pax9	ENSMUST00000001538.9	paired box 9
196	Atp10b	ENSMUST00000077659.5	ATPase, class V, type 10B
197	Stat4	ENSMUST00000168302.2	signal transducer and activator of transcription 4
198	Zpbp2	ENSMUST00000081033.7	zona pellucida binding protein 2
199	4930549C01Rik	ENSMUST00000046647.2	RIKEN cDNA 4930549C01 gene
200	Fbxl4	ENSMUST00000039234.4	F-box and leucine-rich repeat protein 4
201	Ppm1a	ENSMUST00000021514.8	protein phosphatase 1A, magnesium dependent, alpha isoform
202	Gm5878	ENSMUST00000113802.3	predicted gene 5878
203	Fam134b	ENSMUST00000022881.8	family with sequence similarity 134, member B
204	Nexn	ENSMUST00000046045.8	nexilin
205	Sfrp4	ENSMUST00000002883.5	secreted frizzled-related protein 4
206	Gimap1	ENSMUST00000054368.4	GTPase, IMAP family member 1
207	Zbtb33	ENSMUST00000049740.2	zinc finger and BTB domain containing 33
208	Usp51	ENSMUST00000095755.3	ubiquitin specific protease 51
209	Pecr	ENSMUST00000027381.7	peroxisomal trans-2-enoyl-CoA reductase
210	Grhl1	ENSMUST00000020985.8	grainyhead-like 1 (Drosophila)
211	Mtf2	ENSMUST00000081567.5	metal response element binding transcription factor 2
212	Tfap2b	ENSMUST00000027059.5	transcription factor AP-2 beta
213	Mxi1	ENSMUST00000003870.8	Max interacting protein 1
214	Gtpbp10	ENSMUST00000115441.3	GTP-binding protein 10 (putative)
215	AI593442	ENSMUST00000098768.2	expressed sequence AI593442
216	Serf2	ENSMUST00000139253.2	small EDRK-rich factor 2
217	Slc41a1	ENSMUST00000086559.6	solute carrier family 41, member 1
218	Rwdd2b	ENSMUST00000039101.6	RWD domain containing 2B
219	Spopl	ENSMUST00000132484.1	speckle-type POZ protein-like
220	Tmem263	ENSMUST00000095383.4	transmembrane protein 263
221	Nebl	ENSMUST00000028080.6	nebulette
222	Tsc22d4	ENSMUST00000100539.4	TSC22 domain family, member 4
223	Dnajc19	ENSMUST00000117223.1	DnaJ (Hsp40) homolog, subfamily C, member 19
224	Herpud1	ENSMUST00000161576.2	homocysteine-inducible, endoplasmic reticulum stress-inducible, ubiquitin-like domain member 1
225	Gmcl1	ENSMUST00000001185.8	germ cell-less homolog 1 (Drosophila)
226	Atp6v0a4	ENSMUST00000040259.5	ATPase, H+ transporting, lysosomal V0 subunit A4
227	Lpar5	ENSMUST00000088292.5	lysophosphatidic acid receptor 5
228	Nck1	ENSMUST00000116522.2	non-catalytic region of tyrosine kinase adaptor protein 1
229	Defb38	ENSMUST00000066416.3	defensin beta 38
230	Paox	ENSMUST00000026537.6	polyamine oxidase (exo-N4-amino)
231	Aif1l	ENSMUST00000001920.7	allograft inflammatory factor 1-like
232	Txndc12	ENSMUST00000030296.8	thioredoxin domain containing 12 (endoplasmic reticulum)

233	Cops2	ENSMUST00000110463.2	COP9 (constitutive photomorphogenic) homolog, subunit 2 (Arabidopsis thaliana)
234	Ss18l1	ENSMUST00000041126.8	synovial sarcoma translocation gene on chromosome 18-like 1
235	Mc3r	ENSMUST00000038532.1	melanocortin 3 receptor
236	Plscr4	ENSMUST00000034941.8	phospholipid scramblase 4
237	Hsph1	ENSMUST00000074846.8	heat shock 105kDa/110kDa protein 1
238	Hist1h2bm	ENSMUST00000110476.3	histone cluster 1, H2bm
239	Rab37	ENSMUST00000067754.5	RAB37, member RAS oncogene family
240	Lmtk2	ENSMUST00000041804.7	lemur tyrosine kinase 2
241	Taf1a	ENSMUST00000097043.5	TATA box binding protein (Tbp)-associated factor, RNA polymerase I, A
242	Olfr1426	ENSMUST00000112952.1	olfactory receptor 1426
243	Acot7	ENSMUST00000167926.2	acyl-CoA thioesterase 7
244	Gm7120	ENSMUST00000177361.1	predicted gene 7120
245	Lix1	ENSMUST00000115576.2	limb expression 1 homolog (chicken)
246	Ryr3	ENSMUST00000080673.6	ryanodine receptor 3
247	Ctsl	ENSMUST00000021933.7	cathepsin L
248	Gm16039	ENSMUST00000162034.2	predicted gene 16039
249	Sat1	ENSMUST00000026318.9	spermidine/spermine N1-acetyl transferase 1
250	Otud6b	ENSMUST00000117268.2	OTU domain containing 6B
251	Igfbp4	ENSMUST00000017637.7	insulin-like growth factor binding protein 4
252	Gnpat	ENSMUST00000034466.4	glyceronephosphate O-acyltransferase
253	Gabrb1	ENSMUST00000031122.7	gamma-aminobutyric acid (GABA) A receptor, subunit beta 1
254	Actl6a	ENSMUST00000029214.8	actin-like 6A
255	Onecut1	ENSMUST00000056006.10	one cut domain, family member 1
256	Scd1	ENSMUST00000041331.2	stearoyl-Coenzyme A desaturase 1
257	Stc1	ENSMUST00000014957.8	stanniocalcin 1
258	BC026585	ENSMUST00000119891.1	cDNA sequence BC026585
259	Tex16	ENSMUST00000038546.6	testis expressed gene 16
260	Fundc1	ENSMUST00000026016.7	FUN14 domain containing 1
261	Ick	ENSMUST00000044551.7	intestinal cell kinase
262	Slc7a3	ENSMUST00000101362.2	solute carrier family 7 (cationic amino acid transporter, y+ system), member 3
263	Rfx5	ENSMUST00000107254.2	regulatory factor X, 5 (influences HLA class II expression)
264	Tbc1d13	ENSMUST00000044556.6	TBC1 domain family, member 13
265	Zscan22	ENSMUST00000117189.1	zinc finger and SCAN domain containing 22
266	Pcdhb3	ENSMUST00000051754.1	protocadherin beta 3
267	Id4	ENSMUST00000021810.1	inhibitor of DNA binding 4
268	Mug2	ENSMUST00000081777.6	murinoglobulin 2
269	Galm	ENSMUST00000039205.5	galactose mutarotase
270	Mpdz	ENSMUST00000107262.2	multiple PDZ domain protein
271	Mxra7	ENSMUST00000047715.6	matrix-remodelling associated 7
272	Irx4	ENSMUST00000176684.2	Iroquois related homeobox 4 (Drosophila)
273	Emilin2	ENSMUST00000024849.9	elastin microfibril interfacer 2
274	Pdha1	ENSMUST00000033662.8	pyruvate dehydrogenase E1 alpha 1
275	Fads3	ENSMUST00000115995.2	fatty acid desaturase 3
276	Actr2	ENSMUST00000000137.7	ARP2 actin-related protein 2
277	Zfp53	ENSMUST00000076664.6	zinc finger protein 53
278	9430069I07Rik	ENSMUST00000179647.1	RIKEN cDNA 9430069I07 gene

279	Gm10061	ENSMUST00000073111.2	predicted gene 10061
280	Dazap1	ENSMUST00000105362.2	DAZ associated protein 1
281	D930020B18Rik	ENSMUST00000120642.2	RIKEN cDNA D930020B18 gene
282	Ttc8	ENSMUST00000079146.7	tetratricopeptide repeat domain 8
283	Gipc2	ENSMUST00000046614.9	GIPC PDZ domain containing family, member 2
284	Gm10134	ENSMUST00000074761.4	predicted gene 10134
285	Mblac2	ENSMUST00000057598.5	metallo-beta-lactamase domain containing 2
286	Pds5b	ENSMUST00000016569.5	PDS5, regulator of cohesion maintenance, homolog B (S. cerevisiae)
287	Asb17	ENSMUST00000044089.3	ankyrin repeat and SOCS box-containing 17
288	Prrg1	ENSMUST00000114025.2	proline rich Gla (G-carboxyglutamic acid) 1
289	Mier3	ENSMUST00000109272.3	mesoderm induction early response 1, family member 3
290	Col10a1	ENSMUST00000105511.1	collagen, type X, alpha 1
291	Pan3	ENSMUST00000176600.2	PAN3 polyA specific ribonuclease subunit homolog (S. cerevisiae)
292	Mmp10	ENSMUST00000034488.2	matrix metallopeptidase 10
293	Cnot6l	ENSMUST00000155901.2	CCR4-NOT transcription complex, subunit 6-like
294	Hist2h2be	ENSMUST00000090781.7	histone cluster 2, H2be
295	Rab11fip4	ENSMUST00000017783.7	RAB11 family interacting protein 4 (class II)
296	Wbp5	ENSMUST00000048687.5	WW domain binding protein 5
297	Bhlhe22	ENSMUST00000026120.7	basic helix-loop-helix family, member e22
298	Il2ra	ENSMUST00000028111.4	interleukin 2 receptor, alpha chain
299	Pfdn4	ENSMUST00000063682.6	prefoldin 4
300	Armc9	ENSMUST00000131412.2	armadillo repeat containing 9
301	Baz1b	ENSMUST00000002825.5	bromodomain adjacent to zinc finger domain, 1B
302	Pcmtd1	ENSMUST00000061280.11	protein-L-isoaspartate (D-aspartate) O-methyltransferase domain containing 1
303	Adcy3	ENSMUST00000124505.2	adenylate cyclase 3
304	Tmem237	ENSMUST00000094917.4	transmembrane protein 237
305	Rgs9bp	ENSMUST00000069912.5	regulator of G-protein signalling 9 binding protein
306	AI606181	ENSMUST00000099454.3	expressed sequence AI606181
307	Fry	ENSMUST00000087204.5	furry homolog (Drosophila)
308	Zfp874a	ENSMUST00000057241.9	zinc finger protein 874a
309	Crlf3	ENSMUST00000103233.4	cytokine receptor-like factor 3
310	Smim9	ENSMUST00000101433.3	small integral membrane protein 9
311	Rbbp5	ENSMUST00000027700.9	retinoblastoma binding protein 5
312	Olfr613	ENSMUST00000106888.1	olfactory receptor 613
313	Edil3	ENSMUST00000081769.7	EGF-like repeats and discoidin I-like domains 3
314	Hif1an	ENSMUST00000040455.4	hypoxia-inducible factor 1, alpha subunit inhibitor
315	D5Ertd577e	ENSMUST00000094593.3	DNA segment, Chr 5, ERATO Doi 577, expressed
316	Snx6	ENSMUST00000005798.8	sorting nexin 6
317	Mlf1	ENSMUST00000061322.8	myeloid leukemia factor 1
318	Gabrb2	ENSMUST00000007797.4	gamma-aminobutyric acid (GABA) A receptor, subunit beta 2
319	Ptpn21	ENSMUST00000170188.2	protein tyrosine phosphatase, non-receptor type 21
320	Alg10b	ENSMUST00000100309.1	asparagine-linked glycosylation 10B (alpha-1,2-glucosyltransferase)
321	Pura	ENSMUST00000051301.3	purine rich element binding protein A
322	Ina	ENSMUST00000037636.3	internexin neuronal intermediate filament protein, alpha
323	Gpr111	ENSMUST00000113614.2	G protein-coupled receptor 111

324	Gm5128	ENSMUST00000180198.1	predicted gene 5128
325	Jrkl	ENSMUST00000110582.2	Jrk-like
326	Pot1b	ENSMUST00000086876.5	protection of telomeres 1B
327	Gulp1	ENSMUST00000162600.1	GULP, engulfment adaptor PTB domain containing 1
328	Angptl1	ENSMUST00000027885.7	angiopoietin-like 1
329	9130401M01Rik	ENSMUST00000177504.3	RIKEN cDNA 9130401M01 gene
330	Lrrc4	ENSMUST00000062304.6	leucine rich repeat containing 4
331	Msantd4	ENSMUST00000047173.9	Myb/SANT-like DNA-binding domain containing 4 with coiled-coils
332	Mafk	ENSMUST00000018287.4	v-maf musculoaponeurotic fibrosarcoma oncogene family, protein K (avian)
333	Rfc3	ENSMUST00000038131.9	replication factor C (activator 1) 3
334	Il4	ENSMUST00000150568.2	interleukin 4
335	Slc7a2	ENSMUST00000057784.9	solute carrier family 7 (cationic amino acid transporter, y+ system), member 2
336	Six6	ENSMUST00000021519.6	sine oculis-related homeobox 6
337	Cdc73	ENSMUST00000018337.8	cell division cycle 73, Paf1/RNA polymerase II complex component
338	Mcmdc2	ENSMUST00000171802.2	minichromosome maintenance domain containing 2
339	AB041806	ENSMUST00000062902.6	hypothetical protein, MNCb-2457
340	Smyd4	ENSMUST00000044530.2	SET and MYND domain containing 4
341	Smim3	ENSMUST00000042710.6	small integral membrane protein 3
342	Gabra4	ENSMUST00000031121.5	gamma-aminobutyric acid (GABA) A receptor, subunit alpha 4
343	Rpl5	ENSMUST00000082223.7	ribosomal protein L5
344	Eya3	ENSMUST00000081726.7	eyes absent 3 homolog (Drosophila)
345	Kcnd2	ENSMUST00000081542.5	potassium voltage-gated channel, Shal-related family, member 2
346	Snrk	ENSMUST00000120173.2	SNF related kinase
347	Slc2a12	ENSMUST00000042261.4	solute carrier family 2 (facilitated glucose transporter), member 12
348	Zfp664	ENSMUST00000111417.3	zinc finger protein 664
349	Lrrc49	ENSMUST00000053171.8	leucine rich repeat containing 49
350	Capn7	ENSMUST00000022451.8	calpain 7
351	Pfkm	ENSMUST00000163507.2	phosphofructokinase, muscle
352	Clcn4-2	ENSMUST00000000619.6	chloride channel 4-2
353	2610021A01Rik	ENSMUST00000163475.1	RIKEN cDNA 2610021A01 gene
354	Col4a3bp	ENSMUST00000077672.5	collagen, type IV, alpha 3 (Goodpasture antigen) binding protein
355	Tpd52	ENSMUST00000120143.2	tumor protein D52
356	Cmah	ENSMUST00000167746.2	cytidine monophospho-N-acetylneuraminic acid hydroxylase
357	Mrps36	ENSMUST00000109333.2	mitochondrial ribosomal protein S36
358	Hnrnpa2b1	ENSMUST00000114459.2	heterogeneous nuclear ribonucleoprotein A2/B1
359	Pwwp2a	ENSMUST00000094294.4	PWWP domain containing 2A
360	Elovl6	ENSMUST00000071402.2	ELOVL family member 6, elongation of long chain fatty acids (yeast)
361	Tcf24	ENSMUST00000185184.1	transcription factor 24
362	Pmaip1	ENSMUST00000025399.7	phorbol-12-myristate-13-acetate-induced protein 1
363	Ugt2b34	ENSMUST00000113333.1	UDP glucuronosyltransferase 2 family, polypeptide B34

364	Slc15a2	ENSMUST00000023616.3	solute carrier family 15 (H+/peptide transporter), member 2
365	Foxi3	ENSMUST00000069634.5	forkhead box I3
366	Nbn	ENSMUST00000029879.9	nibrin
367	Crxos	ENSMUST00000150050.2	cone-rod homeobox, opposite strand
368	Arhgap6	ENSMUST00000112131.3	Rho GTPase activating protein 6
369	Rab9b	ENSMUST00000058814.6	RAB9B, member RAS oncogene family
370	Ube2d3	ENSMUST00000166033.1	ubiquitin-conjugating enzyme E2D 3
371	Atp5g1	ENSMUST00000090541.6	ATP synthase, H+ transporting, mitochondrial F0 complex, subunit C1 (subunit 9)
372	Kcnab1	ENSMUST00000161404.2	potassium voltage-gated channel, shaker-related subfamily, beta member 1
373	Kcns3	ENSMUST00000164495.2	potassium voltage-gated channel, delayed-rectifier, subfamily S, member 3
374	Ssr1	ENSMUST00000021864.6	signal sequence receptor, alpha
375	Got1l1	ENSMUST00000038174.6	glutamic-oxaloacetic transaminase 1-like 1
376	Hebp2	ENSMUST00000020000.5	heme binding protein 2
377	Blcap	ENSMUST00000109528.3	bladder cancer associated protein homolog (human)
378	Cd59b	ENSMUST00000090429.2	CD59b antigen
379	Gnai2	ENSMUST00000055704.6	guanine nucleotide binding protein (G protein), alpha inhibiting 2
380	Eral1	ENSMUST00000021183.3	Era (G-protein)-like 1 (E. coli)
381	Cxxc4	ENSMUST00000181904.1	CXXC finger 4
382	Atf6	ENSMUST00000027974.6	activating transcription factor 6
383	Mkx	ENSMUST00000079788.5	mohawk homeobox
384	Hist1h4c	ENSMUST00000102967.1	histone cluster 1, H4c
385	Srp54a	ENSMUST00000021407.10	signal recognition particle 54A
386	Nsmce2	ENSMUST00000168722.1	non-SMC element 2 homolog (MMS21, S. cerevisiae)
387	Dtl	ENSMUST00000027933.5	denticleless homolog (Drosophila)
388	AU016765	ENSMUST00000169472.1	expressed sequence AU016765
389	Paqr9	ENSMUST00000079597.6	progestin and adipoQ receptor family member IX
390	Rbpj	ENSMUST00000087360.3	recombination signal binding protein for immunoglobulin kappa J region
391	Fam73a	ENSMUST00000073089.7	family with sequence similarity 73, member A
392	Nt5dc1	ENSMUST00000047885.8	5'-nucleotidase domain containing 1
393	Vkorc1l1	ENSMUST00000073945.4	vitamin K epoxide reductase complex, subunit 1-like 1
394	Ykt6	ENSMUST00000002818.8	YKT6 homolog (S. Cerevisiae)
395	Gm9970	ENSMUST00000068997.3	predicted gene 9970
396	Nat3	ENSMUST00000070514.3	N-acetyltransferase 3
397	Trim43c	ENSMUST00000163255.3	tripartite motif-containing 43C
398	Hif1a	ENSMUST00000110461.2	hypoxia inducible factor 1, alpha subunit
399	Rab14	ENSMUST00000113025.1	RAB14, member RAS oncogene family
400	Bhmt2	ENSMUST00000015941.7	betaine-homocysteine methyltransferase 2
401	Mrm1	ENSMUST00000018549.7	mitochondrial rRNA methyltransferase 1 homolog (S. cerevisiae)
402	Lcorl	ENSMUST00000045586.7	ligand dependent nuclear receptor corepressor-like
403	A130051J06Rik	ENSMUST00000180649.1	RIKEN cDNA A130051J0 gene
404	Gvin1	ENSMUST00000006667.5	GTPase, very large interferon inducible 1
405	Gm4070	ENSMUST00000106766.3	predicted gene 4070
406	Srgap1	ENSMUST00000020322.6	SLIT-ROBO Rho GTPase activating protein 1

407	Igf2bp3	ENSMUST00000031838.7	insulin-like growth factor 2 mRNA binding protein 3
408	Hist1h2bh	ENSMUST00000078156.4	histone cluster 1, H2bh
409	Suz12	ENSMUST00000017692.9	suppressor of zeste 12 homolog (Drosophila)
410	Trim43a	ENSMUST00000164661.3	tripartite motif-containing 43A
411	Bnip3l	ENSMUST00000111115.2	BCL2/adenovirus E1B interacting protein 3-like
412	Zak	ENSMUST00000135469.2	sterile alpha motif and leucine zipper containing kinase AZK
413	Fkbp7	ENSMUST00000002809.8	FK506 binding protein 7
414	Gm3055	ENSMUST00000105316.2	predicted gene 3055
415	Becn1	ENSMUST00000130916.2	beclin 1, autophagy related
416	Hpgds	ENSMUST00000031982.4	hematopoietic prostaglandin D synthase
417	Mfsd4	ENSMUST00000046658.4	major facilitator superfamily domain containing 4
418	Mip	ENSMUST00000026455.7	major intrinsic protein of eye lens fiber
419	Trmt10a	ENSMUST00000040321.7	tRNA methyltransferase 10A
420	Zfp781	ENSMUST00000076281.5	zinc finger protein 781
421	Gm5444	ENSMUST00000065956.3	predicted gene 5444
422	Taf4b	ENSMUST00000169862.1	TAF4B RNA polymerase II, TATA box binding protein (TBP)-associated factor
423	Ubr3	ENSMUST00000055758.10	ubiquitin protein ligase E3 component n-recognin 3
424	Eif5a2	ENSMUST00000060500.7	eukaryotic translation initiation factor 5A2
425	Sptssb	ENSMUST00000171529.2	serine palmitoyltransferase, small subunit B
426	Alg14	ENSMUST00000039442.7	asparagine-linked glycosylation 14
427	Mettl15	ENSMUST00000081631.4	methyltransferase like 15
428	St8sia4	ENSMUST00000043336.5	ST8 alpha-N-acetyl-neuraminide alpha-2,8-sialyltransferase 4
429	Zfp352	ENSMUST00000107129.1	zinc finger protein 352
430	Clasp2	ENSMUST00000166734.3	CLIP associating protein 2
431	Dvl2	ENSMUST00000190940.1	dishevelled 2, dsh homolog (Drosophila)
432	Mpp5	ENSMUST00000082024.5	membrane protein, palmitoylated 5 (MAGUK p55 subfamily member 5)
433	Zfp292	ENSMUST00000098163.3	zinc finger protein 292
434	Srp54b	ENSMUST00000110708.2	signal recognition particle 54B
435	Atf7ip2	ENSMUST00000044005.8	activating transcription factor 7 interacting protein 2
436	Lmo3	ENSMUST00000162772.2	LIM domain only 3
437	Prps1	ENSMUST00000033809.3	phosphoribosyl pyrophosphate synthetase 1
438	BC030500	ENSMUST00000062978.6	cDNA sequence BC030500
439	Ipcef1	ENSMUST00000105617.2	interaction protein for cytohesin exchange factors 1
440	Btg2	ENSMUST00000020692.6	B cell translocation gene 2, anti-proliferative
441	Bok	ENSMUST00000027499.7	BCL2-related ovarian killer protein
442	Sfrp1	ENSMUST00000033952.7	secreted frizzled-related protein 1
443	Myo16	ENSMUST00000042103.8	myosin XVI
444	Lifr	ENSMUST00000171588.1	leukemia inhibitory factor receptor
445	Slc10a7	ENSMUST00000034111.8	solute carrier family 10 (sodium/bile acid cotransporter family), member 7
446	Kctd18	ENSMUST00000164963.2	potassium channel tetramerisation domain containing 18
447	Zic1	ENSMUST00000034927.7	zinc finger protein of the cerebellum 1
448	Neurog1	ENSMUST00000058475.4	neurogenin 1
449	Srek1ip1	ENSMUST00000022230.9	splicing regulatory glutamine/lysine-rich protein 1interacting protein 1
450	Wsb1	ENSMUST00000017821.6	WD repeat and SOCS box-containing 1
451	Etnk1	ENSMUST00000032413.4	ethanolamine kinase 1

452	Vta1	ENSMUST00000154132.2	Vps20-associated 1 homolog (S. cerevisiae)
453	Npy6r	ENSMUST00000042747.3	neuropeptide Y receptor Y6
454	Cep97	ENSMUST00000023270.8	centrosomal protein 97
455	Gm9804	ENSMUST00000056234.3	predicted gene 9804
456	Gxylt1	ENSMUST00000057896.4	glucoside xylosyltransferase 1
457	Slc5a6	ENSMUST00000114665.2	solute carrier family 5 (sodium-dependent vitamin transporter), member 6
458	Hspa4	ENSMUST00000020630.7	heat shock protein 4
459	Pycr2	ENSMUST00000027802.7	pyrroline-5-carboxylate reductase family, member 2
460	Mcoln1	ENSMUST00000004683.7	mucolipin 1
461	B230219D22Rik	ENSMUST00000057844.8	RIKEN cDNA B230219D22 gene
462	Krt7	ENSMUST00000068904.8	keratin 7
463	Lyrm1	ENSMUST00000106516.1	LYR motif containing 1
464	Sesn3	ENSMUST00000034507.7	sestrin 3
465	Gabra1	ENSMUST00000020707.6	gamma-aminobutyric acid (GABA) A receptor, subunit alpha 1
466	Rfwd3	ENSMUST00000038739.4	ring finger and WD repeat domain 3
467	Itgb1	ENSMUST00000090006.6	integrin beta 1 (fibronectin receptor beta)
468	Il17a	ENSMUST00000027061.4	interleukin 17A
469	Atp11a	ENSMUST00000033818.4	ATPase, class VI, type 11A
470	Olfr164	ENSMUST00000056727.4	olfactory receptor 164
471	Pcdhb17	ENSMUST00000053856.4	protocadherin beta 17
472	Zfp512	ENSMUST00000076264.4	zinc finger protein 512
473	Gid4	ENSMUST00000070681.6	GID complex subunit 4, VID24 homolog (S. cerevisiae)
474	Zfp281	ENSMUST00000047734.9	zinc finger protein 281
475	Lrrtm1	ENSMUST00000159616.1	leucine rich repeat transmembrane neuronal 1
476	Lamtor5	ENSMUST00000145735.1	late endosomal/lysosomal adaptor, MAPK and MTOR activator 5
477	Ctsr	ENSMUST00000021889.4	cathepsin R
478	Sox1	ENSMUST00000180353.1	SRY (sex determining region Y)-box 1
479	Ankef1	ENSMUST00000121717.1	ankyrin repeat and EF-hand domain containing 1
480	Htt	ENSMUST00000080036.2	huntingtin
481	Plk1	ENSMUST00000033154.6	polo-like kinase 1
482	Rhbdd1	ENSMUST00000027322.8	rhomboid domain containing 1
483	Cwf19l2	ENSMUST00000027027.5	CWF19-like 2, cell cycle control (S. pombe)
484	Heatr3	ENSMUST00000034079.8	HEAT repeat containing 3
485	Kctd9	ENSMUST00000078053.7	potassium channel tetramerisation domain containing 9
486	Rasgrp3	ENSMUST00000164192.2	RAS, guanyl releasing protein 3
487	Pop4	ENSMUST00000032585.6	processing of precursor 4, ribonuclease P/MRP family, (S. cerevisiae)
488	Pcdha10	ENSMUST00000115659.4	protocadherin alpha 10
489	Il22	ENSMUST00000096691.4	interleukin 22
490	Fam122b	ENSMUST00000071023.6	family with sequence similarity 122, member B
491	Strip2	ENSMUST00000046028.7	striatin interacting protein 2
492	1700001L19Rik	ENSMUST00000022007.6	RIKEN cDNA 1700001L19 gene
493	Zfp275	ENSMUST00000114499.2	zinc finger protein 275
494	Dhdh	ENSMUST00000011526.4	dihydrodiol dehydrogenase (dimeric)
495	Iltifb	ENSMUST00000163808.1	interleukin 10-related T cell-derived inducible factor beta
496	F13a1	ENSMUST00000164727.2	coagulation factor XIII, A1 subunit

497	Ccdc121	ENSMUST00000058825.4	coiled-coil domain containing 121
498	Acbd3	ENSMUST00000027780.4	acyl-Coenzyme A binding domain containing 3
499	Srd5a2	ENSMUST00000043458.7	steroid 5 alpha-reductase 2
500	Hcar2	ENSMUST00000057145.5	hydroxycarboxylic acid receptor 2
501	Dnajc16	ENSMUST00000038014.5	DnaJ (Hsp40) homolog, subfamily C, member 16
502	Chrnb4	ENSMUST00000034854.6	cholinergic receptor, nicotinic, beta polypeptide 4
503	Asz1	ENSMUST00000010940.5	ankyrin repeat, SAM and basic leucine zipper domain containing 1
504	Ammecr1	ENSMUST00000041317.2	Alport syndrome, mental retardation, midface hypoplasia and elliptocytosis chromosomal region gene 1
505	Acsm4	ENSMUST00000047045.8	acyl-CoA synthetase medium-chain family member 4
506	Mctp2	ENSMUST00000079323.6	multiple C2 domains, transmembrane 2
507	C130032M10Rik	ENSMUST00000180393.1	RIKEN cDNA C130032M10 gene
508	Plekhh1	ENSMUST00000039928.5	pleckstrin homology domain containing, family H (with MyTH4 domain) member 1
509	Ddx6	ENSMUST00000170489.1	DEAD (Asp-Glu-Ala-Asp) box polypeptide 6
510	Zfp36l1	ENSMUST00000021552.1	zinc finger protein 36, C3H type-like 1
511	Map2k6	ENSMUST00000020949.6	mitogen-activated protein kinase kinase 6
512	Hexdc	ENSMUST00000038831.9	hexosaminidase (glycosyl hydrolase family 20, catalytic domain) containing
513	F2rl1	ENSMUST00000022185.8	coagulation factor II (thrombin) receptor-like 1
514	Slc25a35	ENSMUST00000018884.5	solute carrier family 25, member 35
515	Pard6g	ENSMUST00000070219.7	par-6 family cell polarity regulator gamma
516	Mtmr6	ENSMUST00000022563.7	myotubularin related protein 6
517	Nkx1-2	ENSMUST00000054562.3	NK1 transcription factor related, locus 2 (Drosophila)
518	Znrf2	ENSMUST00000079869.7	zinc and ring finger 2
519	Fbxl5	ENSMUST00000047857.10	F-box and leucine-rich repeat protein 5
520	Morc4	ENSMUST00000033811.8	microrchidia 4
521	Fbxl3	ENSMUST00000022720.9	F-box and leucine-rich repeat protein 3
522	Trim52	ENSMUST00000022708.5	tripartite motif-containing 52
523	Lrrc61	ENSMUST00000114545.2	leucine rich repeat containing 61
524	Hoxd13	ENSMUST00000001872.4	homeobox D13
525	Srsf12	ENSMUST00000067864.2	serine/arginine-rich splicing factor 12
526	AI597479	ENSMUST00000010434.7	expressed sequence AI597479
527	9230019H11Rik	ENSMUST00000095874.2	RIKEN cDNA 9230019H11 gene
528	Orc1	ENSMUST00000102744.3	origin recognition complex, subunit 1
529	Pde12	ENSMUST00000052932.9	phosphodiesterase 12
530	BC027072	ENSMUST00000057405.7	cDNA sequence BC027072
531	Csf2rb2	ENSMUST00000096356.3	colony stimulating factor 2 receptor, beta 2, low-affinity (granulocyte-macrophage)
532	Tbccd1	ENSMUST00000004576.6	TBCC domain containing 1
533	Tspan2	ENSMUST00000029451.6	tetraspanin 2
534	Uckl1	ENSMUST00000057816.9	uridine-cytidine kinase 1-like 1
535	Zbed6	ENSMUST00000027736.7	zinc finger, BED domain containing 6
536	Zhx1	ENSMUST00000175805.3	zinc fingers and homeoboxes 1
537	Rnase6	ENSMUST00000095923.3	ribonuclease, RNase A family, 6
538	Mrpl13	ENSMUST00000172387.2	mitochondrial ribosomal protein L13
539	Hoxd12	ENSMUST00000001878.5	homeobox D12
540	Cd14	ENSMUST00000061829.6	CD14 antigen
541	Cd38	ENSMUST00000030964.4	CD38 antigen
542	Enpep	ENSMUST00000029658.8	glutamyl aminopeptidase

543	Mccc1	ENSMUST00000029259.4	methylcrotonoyl-Coenzyme A carboxylase 1 (alpha)
544	Lyn	ENSMUST00000041377.7	Yamaguchi sarcoma viral (v-yes-1) oncogene homolog
545	Gm904	ENSMUST00000099519.1	predicted gene 904
546	Hyal4	ENSMUST00000031691.2	hyaluronoglucosaminidase 4
547	Kprp	ENSMUST00000072363.4	keratinocyte expressed, proline-rich
548	4930432K21Rik	ENSMUST00000118856.2	RIKEN cDNA 4930432K21 gene
549	6030445D17Rik	ENSMUST00000084593.2	RIKEN cDNA 6030445D17 gene
550	Lrp6	ENSMUST00000032322.9	low density lipoprotein receptor-related protein 6
551	Rabgef1	ENSMUST00000119797.2	RAB guanine nucleotide exchange factor (GEF) 1
552	Hist1h2bk	ENSMUST00000110455.3	histone cluster 1, H2bk
553	Dnase1l2	ENSMUST00000088506.6	deoxyribonuclease 1-like 2
554	Cideb	ENSMUST00000001497.7	cell death-inducing DNA fragmentation factor, alpha subunit-like effector B
555	Rnpc3	ENSMUST00000092154.4	RNA-binding region (RNP1, RRM) containing 3
556	Ywhah	ENSMUST00000019109.7	tyrosine 3-monooxygenase/tryptophan 5-monooxygenase activation protein, eta polypeptide
557	Bod1	ENSMUST00000058060.8	biorientation of chromosomes in cell division 1
558	Enoph1	ENSMUST00000169390.2	enolase-phosphatase 1
559	Kcnt2	ENSMUST00000120709.2	potassium channel, subfamily T, member 2
560	Msh4	ENSMUST00000005630.5	mutS homolog 4 (E. coli)
561	Mpp3	ENSMUST00000100400.3	membrane protein, palmitoylated 3 (MAGUK p55 subfamily member 3)
562	Asb15	ENSMUST00000117688.1	ankyrin repeat and SOCS box-containing 15
563	Olfm3	ENSMUST00000081752.7	olfactomedin 3
564	Mrgprb3	ENSMUST00000094383.2	MAS-related GPR, member B3
565	Ncapg	ENSMUST00000117396.1	non-SMC condensin I complex, subunit G
566	Tmcc3	ENSMUST00000065060.6	transmembrane and coiled coil domains 3
567	BC021891	ENSMUST00000034316.4	cDNA sequence BC021891
568	Nim1k	ENSMUST00000178973.1	NIM1 serine/threonine protein kinase
569	Gpr165	ENSMUST00000033554.4	G protein-coupled receptor 165
570	Nsa2	ENSMUST00000073456.7	NSA2 ribosome biogenesis homolog (S. cerevisiae)
571	Ripk1	ENSMUST00000167374.2	receptor (TNFRSF)-interacting serine-threonine kinase 1
572	Bcdin3d	ENSMUST00000040313.4	BCDIN3 domain containing
573	4930402H24Rik	ENSMUST00000044766.9	RIKEN cDNA 4930402H24 gene
574	Gabpb2	ENSMUST00000107209.2	GA repeat binding protein, beta 2
575	Mc1r	ENSMUST00000098324.2	melanocortin 1 receptor
576	Ankmy2	ENSMUST00000041640.3	ankyrin repeat and MYND domain containing 2
577	Zfp398	ENSMUST00000114598.1	zinc finger protein 398
578	Ing5	ENSMUST00000027505.7	inhibitor of growth family, member 5
579	Rictor	ENSMUST00000061656.6	RPTOR independent companion of MTOR, complex 2
580	Cpd	ENSMUST00000021201.5	carboxypeptidase D
581	4632428N05Rik	ENSMUST00000020301.8	RIKEN cDNA 4632428N05 gene
582	Samd12	ENSMUST00000078673.7	sterile alpha motif domain containing 12
583	S100a16	ENSMUST00000098910.2	S100 calcium binding protein A16
584	Myl12a	ENSMUST00000148960.2	myosin, light chain 12A, regulatory, non-sarcomeric
585	Trpm3	ENSMUST00000037901.7	transient receptor potential cation channel, subfamily M, member 3
586	Gm1045	ENSMUST00000178614.1	predicted gene 1045
587	Dfna5	ENSMUST00000031845.7	deafness, autosomal dominant 5 (human)

588	Mlana	ENSMUST00000025719.3	melan-A
589	Sos2	ENSMUST00000035773.8	son of sevenless homolog 2 (Drosophila)
590	Gls	ENSMUST00000114510.2	glutaminase
591	Hsd3b7	ENSMUST00000106271.1	hydroxy-delta-5-steroid dehydrogenase, 3 beta- and steroid delta-isomerase 7
592	Ufl1	ENSMUST00000102994.4	UFM1 specific ligase 1
593	Zpbp	ENSMUST00000020413.3	zona pellucida binding protein
594	Fstl1	ENSMUST00000114763.2	follistatin-like 1
595	Capn2	ENSMUST00000068505.8	calpain 2
596	Tcfl5	ENSMUST00000037877.10	transcription factor-like 5 (basic helix-loop-helix)
597	Cldn23	ENSMUST00000060128.5	claudin 23
598	Gm5531	ENSMUST00000055314.3	predicted gene 5531
599	Mapk8	ENSMUST00000111945.3	mitogen-activated protein kinase 8
600	Acss1	ENSMUST00000028944.3	acyl-CoA synthetase short-chain family member 1
601	Hsf3	ENSMUST00000119035.3	heat shock transcription factor 3
602	Tpm4	ENSMUST00000003575.9	tropomyosin 4
603	Ormdl1	ENSMUST00000027266.3	ORM1-like 1 (S. cerevisiae)
604	Zfp619	ENSMUST00000108015.2	zinc finger protein 619
605	Kcnj2	ENSMUST00000042970.2	potassium inwardly-rectifying channel, subfamily J, member 2
606	Gm17567	ENSMUST00000164429.1	predicted gene, 17567
607	Gm11568	ENSMUST00000107434.1	predicted gene 11568
608	Klhl20	ENSMUST00000111611.2	kelch-like 20
609	Parp1	ENSMUST00000027777.6	poly (ADP-ribose) polymerase family, member 1
610	Cdc5l	ENSMUST00000024727.8	cell division cycle 5-like (S. pombe)
611	Memo1	ENSMUST00000078459.6	mediator of cell motility 1
612	Trim7	ENSMUST00000109213.3	tripartite motif-containing 7
613	E2f1	ENSMUST00000103145.5	E2F transcription factor 1
614	Smarcd2	ENSMUST00000106843.2	SWI/SNF related, matrix associated, actin dependent regulator of chromatin, subfamily d, member 2
615	Csnk1g3	ENSMUST00000069597.6	casein kinase 1, gamma 3
616	Dlg1	ENSMUST00000100001.4	discs, large homolog 1 (Drosophila)
617	Jade3	ENSMUST00000115384.3	jade family PHD finger 3
618	Apoc3	ENSMUST00000121916.2	apolipoprotein C-III
619	Bambi	ENSMUST00000025075.1	BMP and activin membrane-bound inhibitor
620	Tlr3	ENSMUST00000034056.5	toll-like receptor 3
621	Scp2d1	ENSMUST00000028918.3	SCP2 sterol-binding domain containing 1
622	Iapp	ENSMUST00000041993.2	islet amyloid polypeptide
623	Snhg11	ENSMUST00000109488.2	small nucleolar RNA host gene 11
624	Ccdc66	ENSMUST00000050480.6	coiled-coil domain containing 66
625	Pou3f1	ENSMUST00000053491.8	POU domain, class 3, transcription factor 1
626	Idi1	ENSMUST00000169314.3	isopentenyl-diphosphate delta isomerase
627	Prps1l3	ENSMUST00000139049.1	phosphoribosyl pyrophosphate synthetase 1-like 3
628	C78339	ENSMUST00000099547.3	expressed sequence C78339
629	Slc35a5	ENSMUST00000023344.4	solute carrier family 35, member A5
630	Csde1	ENSMUST00000029446.8	cold shock domain containing E1, RNA binding
631	Gjb6	ENSMUST00000039380.8	gap junction protein, beta 6
632	Sptlc3	ENSMUST00000047370.2	serine palmitoyltransferase, long chain base subunit 3
633	Smyd2	ENSMUST00000027897.7	SET and MYND domain containing 2
634	Phox2b	ENSMUST00000012664.9	paired-like homeobox 2b
635	Slc9b2	ENSMUST00000051849.8	solute carrier family 9, subfamily B (NHA2, cation proton antiporter 2), member 2

636	Orai1	ENSMUST00000051016.4	ORAI calcium release-activated calcium modulator 1
637	Kcng3	ENSMUST00000051482.1	potassium voltage-gated channel, subfamily G, member 3
638	Dkk3	ENSMUST00000033036.5	dickkopf homolog 3 (Xenopus laevis)
639	9130409I23Rik	ENSMUST00000036819.6	RIKEN cDNA 9130409I23 gene
640	Med20	ENSMUST00000024778.2	mediator complex subunit 20
641	4933416C03Rik	ENSMUST00000099261.2	RIKEN cDNA 4933416C03 gene
642	Gnai1	ENSMUST00000074694.5	guanine nucleotide binding protein (G protein), alpha inhibiting 1
643	Olfr1166	ENSMUST00000099833.3	olfactory receptor 1166
644	Gm7008	ENSMUST00000038121.4	predicted gene 7008
645	Nrg1	ENSMUST00000073884.4	neuregulin 1
646	Hccs	ENSMUST00000033717.3	holocytochrome c synthetase
647	Mef2a	ENSMUST00000156690.2	myocyte enhancer factor 2A
648	Fam208a	ENSMUST00000022450.4	family with sequence similarity 208, member A
649	Prkar2b	ENSMUST00000036497.10	protein kinase, cAMP dependent regulatory, type II beta
650	Trap1	ENSMUST00000006137.8	TNF receptor-associated protein 1
651	Tbx20	ENSMUST00000052946.5	T-box 20
652	Mat1a	ENSMUST00000047286.6	methionine adenosyltransferase I, alpha
653	Mocs3	ENSMUST00000099071.4	molybdenum cofactor synthesis 3
654	Emb	ENSMUST00000022242.7	embigin
655	Gm11541	ENSMUST00000069852.1	predicted gene 11541
656	E2f7	ENSMUST00000073781.6	E2F transcription factor 7
657	Pxylp1	ENSMUST00000112951.3	2-phosphoxylose phosphatase 1
658	Trim43b	ENSMUST00000167113.2	tripartite motif-containing 43B
659	Shpk	ENSMUST00000006105.6	sedoheptulokinase
660	Fam196a	ENSMUST00000171394.1	family with sequence similarity 196, member A
661	Klhdc10	ENSMUST00000068259.6	kelch domain containing 10
662	1810009A15Rik	ENSMUST00000096251.4	RIKEN cDNA 1810009A15 gene
663	Terf1	ENSMUST00000188371.1	telomeric repeat binding factor 1
664	Sh3bgrl	ENSMUST00000033598.8	SH3-binding domain glutamic acid-rich protein like
665	Fgl2	ENSMUST00000035799.5	fibrinogen-like protein 2
666	Oser1	ENSMUST00000046908.4	oxidative stress responsive serine rich 1
667	Kras	ENSMUST00000111710.2	v-Ki-ras2 Kirsten rat sarcoma viral oncogene homolog
668	Ube2q2	ENSMUST00000059555.9	ubiquitin-conjugating enzyme E2Q (putative) 2
669	Fam118a	ENSMUST00000023069.7	family with sequence similarity 118, member A
670	Sox4	ENSMUST00000067230.5	SRY (sex determining region Y)-box 4
671	Fam204a	ENSMUST00000065286.1	family with sequence similarity 204, member A
672	Hnrnpc	ENSMUST00000111610.5	heterogeneous nuclear ribonucleoprotein C
673	Pbdc1	ENSMUST00000033577.5	polysaccharide biosynthesis domain containing 1
674	Fthl17	ENSMUST00000096509.6	ferritin, heavy polypeptide-like 17
675	Tbx4	ENSMUST00000000096.6	T-box 4
676	Nsun2	ENSMUST00000022087.6	NOL1/NOP2/Sun domain family member 2
677	Pcdh19	ENSMUST00000167944.2	protocadherin 19
678	Akr1c12	ENSMUST00000021632.3	aldo-keto reductase family 1, member C12
679	Wnt5a	ENSMUST00000063465.5	wingless-related MMTV integration site 5A
680	D130052B06Rik	ENSMUST00000101371.2	RIKEN cDNA D130052B06 gene
681	Fbxo33	ENSMUST00000043204.7	F-box protein 33
682	Mitf	ENSMUST00000043637.8	microphthalmia-associated transcription factor
683	Ifi203	ENSMUST00000123708.2	interferon activated gene 203
684	Gm5635	ENSMUST00000097029.3	predicted gene 5635

685	Gm10339	ENSMUST00000181509.1	predicted gene 10339
686	Naa60	ENSMUST00000186375.1	N(alpha)-acetyltransferase 60, NatF catalytic subunit
687	Elovl5	ENSMUST00000034904.8	ELOVL family member 5, elongation of long chain fatty acids (yeast)
688	Tfcp2	ENSMUST00000009877.6	transcription factor CP2
689	Jtb	ENSMUST00000119304.1	jumping translocation breakpoint
690	Gatm	ENSMUST00000028624.8	glycine amidinotransferase (L-arginine:glycine amidinotransferase)
691	Ffar4	ENSMUST00000067098.6	free fatty acid receptor 4
692	Uevld	ENSMUST00000094398.5	UEV and lactate/malate dehyrogenase domains
693	Ank	ENSMUST00000022875.6	progressive ankylosis
694	Tbc1d1	ENSMUST00000101195.3	TBC1 domain family, member 1
695	Usp46	ENSMUST00000119154.2	ubiquitin specific peptidase 46
696	Rnasel	ENSMUST00000182538.2	ribonuclease L (2', 5'-oligoisoadenylate synthetase-dependent)
697	Srgn	ENSMUST00000160987.2	serglycin
698	Gm16286	ENSMUST00000127234.2	predicted gene 16286
699	Bckdhb	ENSMUST00000190166.1	branched chain ketoacid dehydrogenase E1, beta polypeptide
700	Zfp507	ENSMUST00000061586.5	zinc finger protein 507
701	Antxr2	ENSMUST00000031281.9	anthrax toxin receptor 2
702	Oaz1	ENSMUST00000060987.9	ornithine decarboxylase antizyme 1
703	Cdh2	ENSMUST00000025166.8	cadherin 2
704	Sohlh2	ENSMUST00000029369.4	spermatogenesis and oogenesis specific basic helix-loop-helix 2
705	Ermn	ENSMUST00000090940.5	ermin, ERM-like protein
706	Fst	ENSMUST00000022287.5	follistatin
707	Tmem255a	ENSMUST00000089054.5	transmembrane protein 255A
708	Pabpc4l	ENSMUST00000166505.1	poly(A) binding protein, cytoplasmic 4-like
709	Tirap	ENSMUST00000175765.2	toll-interleukin 1 receptor (TIR) domain-containing adaptor protein
710	Wdr37	ENSMUST00000054251.7	WD repeat domain 37
711	Hormad2	ENSMUST00000109948.2	HORMA domain containing 2
712	Zfp69	ENSMUST00000106280.2	zinc finger protein 69
713	Plekha1	ENSMUST00000120441.2	pleckstrin homology domain containing, family A (phosphoinositide binding specific) member 1
714	Tcf25	ENSMUST00000108840.2	transcription factor 25 (basic helix-loop-helix)
715	Krt76	ENSMUST00000100179.1	keratin 76
716	Unkl	ENSMUST00000039734.6	unkempt-like (Drosophila)
717	Eps15	ENSMUST00000102729.4	epidermal growth factor receptor pathway substrate 15
718	Svs3b	ENSMUST00000063132.5	seminal vesicle secretory protein 3B
719	March9	ENSMUST00000040307.5	membrane-associated ring finger (C3HC4) 9
720	Dusp3	ENSMUST00000151678.2	dual specificity phosphatase 3 (vaccinia virus phosphatase VH1-related)
721	Akap17b	ENSMUST00000051906.7	A kinase (PRKA) anchor protein 17B
722	Gm5634	ENSMUST00000105006.1	predicted gene 5634
723	Rslcan18	ENSMUST00000091526.3	regulator of sex-limitation candidate 18
724	Ift80	ENSMUST00000107812.2	intraflagellar transport 80
725	Actr3	ENSMUST00000027579.11	ARP3 actin-related protein 3
726	Gm14458	ENSMUST00000105004.1	predicted gene 14458
727	Cdc14a	ENSMUST00000090464.5	CDC14 cell division cycle 14A
728	Mrpl36	ENSMUST00000022098.8	mitochondrial ribosomal protein L36

729	Cpe	ENSMUST00000048967.7	carboxypeptidase E
730	Zfp365	ENSMUST00000064656.7	zinc finger protein 365
731	Zfp93	ENSMUST00000032696.6	zinc finger protein 93
732	Rpp25	ENSMUST00000080514.7	ribonuclease P/MRP 25 subunit
733	Iqcd	ENSMUST00000069259.3	IQ motif containing D
734	Ptplad2	ENSMUST00000030221.2	protein tyrosine phosphatase-like A domain containing 2
735	Zfp597	ENSMUST00000090522.4	zinc finger protein 597
736	Zscan26	ENSMUST00000032820.8	zinc finger and SCAN domain containing 26
737	Bmpr2	ENSMUST00000087435.6	bone morphogenetic protein receptor, type II (serine/threonine kinase)
738	Neil2	ENSMUST00000038229.4	nei like 2 (E. coli)
739	Ehbp1	ENSMUST00000045167.5	EH domain binding protein 1
740	Olfr722	ENSMUST00000053290.6	olfactory receptor 722
741	Gnao1	ENSMUST00000125716.2	guanine nucleotide binding protein, alpha O
742	Zfyve16	ENSMUST00000022217.8	zinc finger, FYVE domain containing 16
743	Lrrc19	ENSMUST00000053419.3	leucine rich repeat containing 19
744	Gm14511	ENSMUST00000075792.4	predicted gene 14511
745	Catip	ENSMUST00000097697.2	ciliogenesis associated TTC17 interacting protein
746	Tmprss11g	ENSMUST00000134179.2	transmembrane protease, serine 11g
747	Gm10251	ENSMUST00000181786.1	predicted gene 10251
748	Rhot1	ENSMUST00000055056.10	ras homolog gene family, member T1
749	Gnpda2	ENSMUST00000031117.7	glucosamine-6-phosphate deaminase 2
750	Gas2	ENSMUST00000107591.3	growth arrest specific 2
751	Ndfip2	ENSMUST00000181969.2	Nedd4 family interacting protein 2
752	Gm2916	ENSMUST00000181779.1	predicted gene 2916
753	Ifi205	ENSMUST00000059226.6	interferon activated gene 205
754	Fam166b	ENSMUST00000171134.3	family with sequence similarity 166, member B
755	Birc5	ENSMUST00000093906.4	baculoviral IAP repeat-containing 5
756	Dtd1	ENSMUST00000028917.6	D-tyrosyl-tRNA deacylase 1
757	Crbn	ENSMUST00000049675.5	cereblon
758	Qsox2	ENSMUST00000091263.6	quiescin Q6 sulfhydryl oxidase 2
759	Cul3	ENSMUST00000163119.2	cullin 3
760	Zfp329	ENSMUST00000108546.1	zinc finger protein 329
761	Slmap	ENSMUST00000090359.5	sarcolemma associated protein
762	Snx1	ENSMUST00000034946.9	sorting nexin 1
763	B3galnt1	ENSMUST00000061826.1	UDP-GalNAc:betaGlcNAc beta 1,3-galactosaminyltransferase, polypeptide 1
764	Mnda	ENSMUST00000009340.8	myeloid cell nuclear differentiation antigen
765	Grifin	ENSMUST00000042993.6	galectin-related inter-fiber protein
766	Dpy19l2	ENSMUST00000133010.2	dpy-19-like 2 (C. elegans)
767	Il6ra	ENSMUST00000029559.6	interleukin 6 receptor, alpha
768	Padi3	ENSMUST00000026377.3	peptidyl arginine deiminase, type III
769	Dtx1	ENSMUST00000031607.6	deltex 1 homolog (Drosophila)
770	Derl1	ENSMUST00000022993.5	Der1-like domain family, member 1
771	Fhl5	ENSMUST00000029922.8	four and a half LIM domains 5
772	Vps37b	ENSMUST00000040967.7	vacuolar protein sorting 37B (yeast)
773	Strn3	ENSMUST00000013130.9	striatin, calmodulin binding protein 3
774	3110040N11Rik	ENSMUST00000026092.8	RIKEN cDNA 3110040N11 gene
775	Dio1	ENSMUST00000082426.4	deiodinase, iodothyronine, type I
776	RP23-391B20.4	ENSMUST00000162374.2	
777	Evx2	ENSMUST00000001867.7	even skipped homeotic gene 2 homolog

778	Pgbd1	ENSMUST00000122872.2	piggyBac transposable element derived 1
779	Laptm4a	ENSMUST00000020909.3	lysosomal-associated protein transmembrane 4A
780	Snapc1	ENSMUST00000021532.4	small nuclear RNA activating complex, polypeptide 1
781	1810046K07Rik	ENSMUST00000039959.5	RIKEN cDNA 1810046K07 gene
782	Chst8	ENSMUST00000078686.6	carbohydrate (N-acetylgalactosamine 4-0) sulfotransferase 8
783	Ocln	ENSMUST00000069756.5	occludin
784	Gm11992	ENSMUST00000043285.4	predicted gene 11992
785	Tnks2	ENSMUST00000025729.6	tankyrase, TRF1-interacting ankyrin-related ADP-ribose polymerase 2
786	Ppp1r8	ENSMUST00000030702.8	protein phosphatase 1, regulatory (inhibitor) subunit 8
787	Ccdc68	ENSMUST00000043929.5	coiled-coil domain containing 68
788	Agps	ENSMUST00000047232.8	alkylglycerone phosphate synthase
789	Arrdc3	ENSMUST00000099356.3	arrestin domain containing 3
790	Ahcyl1	ENSMUST00000029490.9	S-adenosylhomocysteine hydrolase-like 1
791	Axl	ENSMUST00000002677.5	AXL receptor tyrosine kinase
792	Fam173b	ENSMUST00000042702.6	family with sequence similarity 173, member B
793	Itsn2	ENSMUST00000062580.7	intersectin 2
794	Thap8	ENSMUST00000108187.2	THAP domain containing 8
795	Ranbp6	ENSMUST00000099525.3	RAN binding protein 6
796	Ercc8	ENSMUST00000054835.9	excision repaiross-complementing rodent repair deficiency, complementation group 8
797	Abcc5	ENSMUST00000079158.7	ATP-binding cassette, sub-family C (CFTR/MRP), member 5
798	Tiparp	ENSMUST00000047906.4	TCDD-inducible poly(ADP-ribose) polymerase
799	Hsd3b3	ENSMUST00000107019.2	hydroxy-delta-5-steroid dehydrogenase, 3 beta- and steroid delta-isomerase 3
800	Hiat1	ENSMUST00000029570.6	hippocampus abundant gene transcript 1
801	Fcamr	ENSMUST00000027670.3	Fc receptor, IgA, IgM, high affinity
802	Tceal5	ENSMUST00000066819.5	transcription elongation factor A (SII)-like 5
803	B3gnt4	ENSMUST00000031384.5	UDP-GlcNAc:betaGal beta-1,3-N-acetylglucosaminyltransferase 4
804	Dgkb	ENSMUST00000040500.7	diacylglycerol kinase, beta
805	Csnk1e	ENSMUST00000117786.2	casein kinase 1, epsilon
806	Dcbld2	ENSMUST00000046663.7	discoidin, CUB and LCCL domain containing 2
807	BC094916	ENSMUST00000139092.2	cDNA sequence BC094916
808	Frmd6	ENSMUST00000057859.7	FERM domain containing 6
809	Gdap1	ENSMUST00000026879.7	ganglioside-induced differentiation-associated-protein 1
810	Slc25a12	ENSMUST00000151937.2	solute carrier family 25 (mitochondrial carrier, Aralar), member 12
811	Tfb1m	ENSMUST00000041003.6	transcription factor B1, mitochondrial
812	Cdk6	ENSMUST00000165117.2	cyclin-dependent kinase 6
813	Mme	ENSMUST00000029400.1	membrane metallo endopeptidase
814	Plekhg3	ENSMUST00000075249.4	pleckstrin homology domain containing, family G (with RhoGef domain) member 3
815	Tmem218	ENSMUST00000034632.8	transmembrane protein 218
816	Gm21685	ENSMUST00000185072.2	predicted gene, 21685
817	Bdnf	ENSMUST00000053317.6	brain derived neurotrophic factor
818	Sprtn	ENSMUST00000034467.5	SprT-like N-terminal domain
819	Pcbp3	ENSMUST00000001148.5	poly(rC) binding protein 3

820	Srrm1	ENSMUST00000105861.2	serine/arginine repetitive matrix 1
821	Tasp1	ENSMUST00000110079.3	taspase, threonine aspartase 1
822	Gm9923	ENSMUST00000061324.4	predicted pseudogene 9923
823	Larp4b	ENSMUST00000091829.3	La ribonucleoprotein domain family, member 4B
824	Serpinb9	ENSMUST00000063191.8	serine (or cysteine) peptidase inhibitor, clade B, member 9
825	Cdk15	ENSMUST00000160662.2	cyclin-dependent kinase 15
826	Lamp2	ENSMUST00000016678.8	lysosomal-associated membrane protein 2
827	Dcun1d4	ENSMUST00000063882.6	DCN1, defective in cullin neddylation 1, domain containing 4 (S. cerevisiae)
828	Mus81	ENSMUST00000124334.2	MUS81 endonuclease homolog (yeast)
829	Pla2g12a	ENSMUST00000029629.9	phospholipase A2, group XIIA
830	Rgs17	ENSMUST00000131996.2	regulator of G-protein signaling 17
831	Bag2	ENSMUST00000044691.8	BCL2-associated athanogene 2
832	Krt20	ENSMUST00000017743.2	keratin 20
833	Ndfip1	ENSMUST00000025293.3	Nedd4 family interacting protein 1
834	Syt6	ENSMUST00000090697.5	synaptotagmin VI
835	Syne2	ENSMUST00000143031.2	spectrin repeat containing, nuclear envelope 2
836	Kcnmb1	ENSMUST00000020362.2	potassium large conductance calcium-activated channel, subfamily M, beta member 1
837	Cdkn2aip	ENSMUST00000038738.5	CDKN2A interacting protein
838	Nek7	ENSMUST00000186017.1	NIMA (never in mitosis gene a)-related expressed kinase 7
839	Ago3	ENSMUST00000069097.7	argonaute RISC catalytic subunit 3
840	Clic4	ENSMUST00000037099.8	chloride intracellular channel 4 (mitochondrial)
841	Smpdl3b	ENSMUST00000030709.8	sphingomyelin phosphodiesterase, acid-like 3B
842	Pdp1	ENSMUST00000108297.2	pyruvate dehyrogenase phosphatase catalytic subunit 1
843	Tmem167	ENSMUST00000161568.2	transmembrane protein 167
844	Pdzd8	ENSMUST00000099274.2	PDZ domain containing 8
845	Lrsam1	ENSMUST00000028132.8	leucine rich repeat and sterile alpha motif containing 1
846	Mybl1	ENSMUST00000088658.5	myeloblastosis oncogene-like 1
847	Srsf11	ENSMUST00000121326.2	serine/arginine-rich splicing factor 11
848	Opcml	ENSMUST00000115243.3	opioid binding protein/cell adhesion molecule-like
849	Zscan4f	ENSMUST00000091440.2	zinc finger and SCAN domain containing 4F
850	Rph3a	ENSMUST00000079204.5	rabphilin 3A
851	Map1lc3b	ENSMUST00000034270.11	microtubule-associated protein 1 light chain 3 beta
852	Iqcf3	ENSMUST00000062917.9	IQ motif containing F3
853	Eps8	ENSMUST00000058210.7	epidermal growth factor receptor pathway substrate 8
854	Cdkn2a	ENSMUST00000107131.1	cyclin-dependent kinase inhibitor 2A
855	Gm10010	ENSMUST00000071101.6	predicted gene 10010
856	Spryd7	ENSMUST00000022497.9	SPRY domain containing 7
857	Star	ENSMUST00000033979.4	steroidogenic acute regulatory protein
858	Smarcc1	ENSMUST00000035057.8	SWI/SNF related, matrix associated, actin dependent regulator of chromatin, subfamily c, member 1
859	Psma3	ENSMUST00000160027.2	proteasome (prosome, macropain) subunit, alpha type 3
860	Itgav	ENSMUST00000028499.5	integrin alpha V
861	Entpd4	ENSMUST00000184973.2	ectonucleoside triphosphate diphosphohydrolase 4
862	Lypd6	ENSMUST00000112712.4	LY6/PLAUR domain containing 6
863	Cog8	ENSMUST00000095517.6	component of oligomeric golgi complex 8

864	Trim16	ENSMUST00000055006.6	tripartite motif-containing 16
865	Bmi1	ENSMUST00000028071.7	Bmi1 polycomb ring finger oncogene
866	Gm11149	ENSMUST00000068730.3	predicted gene 11149
867	Vwa5a	ENSMUST00000001544.6	von Willebrand factor A domain containing 5A
868	Papln	ENSMUST00000121733.2	papilin, proteoglycan-like sulfated glycoprotein
869	Slco1b2	ENSMUST00000042812.6	solute carrier organic anion transporter family, member 1b2
870	Arhgef28	ENSMUST00000109426.1	Rho guanine nucleotide exchange factor (GEF) 28
871	Srprb	ENSMUST00000035157.9	signal recognition particle receptor, B subunit
872	Fam83a	ENSMUST00000160942.2	family with sequence similarity 83, member A
873	Nfe2l3	ENSMUST00000005103.6	nuclear factor, erythroid derived 2, like 3
874	Ptpn2	ENSMUST00000122412.1	protein tyrosine phosphatase, non-receptor type 2
875	Wdr48	ENSMUST00000036561.6	WD repeat domain 48
876	Ephx1	ENSMUST00000036928.6	epoxide hydrolase 1, microsomal
877	Ppp1r3b	ENSMUST00000070481.6	protein phosphatase 1, regulatory (inhibitor) subunit 3B
878	Ppp3r1	ENSMUST00000102880.4	protein phosphatase 3, regulatory subunit B, alpha isoform (calcineurin B, type I)
879	Ren1	ENSMUST00000094556.2	renin 1 structural
880	Gm10742	ENSMUST00000099198.2	predicted gene 10742
881	Hs2st1	ENSMUST00000043325.7	heparan sulfate 2-O-sulfotransferase 1
882	Numb	ENSMUST00000117217.2	numb gene homolog (Drosophila)
883	Slc38a2	ENSMUST00000023099.6	solute carrier family 38, member 2
884	Bloc1s3	ENSMUST00000077408.6	biogenesis of lysosomal organelles complex-1, subunit 3
885	Tstd2	ENSMUST00000107772.2	thiosulfate sulfurtransferase (rhodanese)-like domain containing 2
886	Slc25a51	ENSMUST00000107796.2	solute carrier family 25, member 51
887	Prkce	ENSMUST00000097275.3	protein kinase C, epsilon
888	Inpp4b	ENSMUST00000172031.2	inositol polyphosphate-4-phosphatase, type II
889	Shc3	ENSMUST00000021898.5	src homology 2 domain-containing transforming protein C3
890	Capza1	ENSMUST00000094028.5	capping protein (actin filament) muscle Z-line, alpha 1
891	Sult1b1	ENSMUST00000031199.5	sulfotransferase family 1B, member 1
892	Dlgap5	ENSMUST00000043296.4	discs, large (Drosophila) homolog-associated protein 5
893	Cbln1	ENSMUST00000034076.9	cerebellin 1 precursor protein
894	Gm7247	ENSMUST00000162998.1	predicted gene 7247
895	Cdca5	ENSMUST00000025704.2	cell division cycle associated 5
896	Cpeb4	ENSMUST00000109412.3	cytoplasmic polyadenylation element binding protein 4
897	Cyp2c50	ENSMUST00000068094.7	cytochrome P450, family 2, subfamily c, polypeptide 50
898	Cyp2c37	ENSMUST00000049178.2	cytochrome P450, family 2. subfamily c, polypeptide 37
899	Krt33a	ENSMUST00000018399.2	keratin 33A
900	Olfr389	ENSMUST00000122224.2	olfactory receptor 389
901	Gabra6	ENSMUST00000020703.7	gamma-aminobutyric acid (GABA) A receptor, subunit alpha 6
902	Dclre1b	ENSMUST00000063502.7	DNA cross-link repair 1B, PSO2 homolog (S. cerevisiae)

903	Nox4	ENSMUST00000032781.8	NADPH oxidase 4
904	Zswim6	ENSMUST00000105097.2	zinc finger SWIM-type containing 6
905	Zmat4	ENSMUST00000042352.5	zinc finger, matrin type 4
906	Pabpc1	ENSMUST00000001809.9	poly(A) binding protein, cytoplasmic 1
907	Mex3d	ENSMUST00000105350.1	mex3 homolog D (C. elegans)
908	Lrrn4cl	ENSMUST00000096257.2	LRRN4 C-terminal like
909	Actl7a	ENSMUST00000095079.5	actin-like 7a
910	Nt5c2	ENSMUST00000086961.8	5'-nucleotidase, cytosolic II
911	Prrx1	ENSMUST00000075805.7	paired related homeobox 1
912	March1	ENSMUST00000110255.2	membrane-associated ring finger (C3HC4) 1
913	Kif9	ENSMUST00000061155.7	kinesin family member 9
914	Tle1	ENSMUST00000107337.2	transducin-like enhancer of split 1, homolog of Drosophila E(spl)
915	Qk	ENSMUST00000097414.4	quaking
916	Slc6a6	ENSMUST00000032185.7	solute carrier family 6 (neurotransmitter transporter, taurine), member 6
917	Acox2	ENSMUST00000022271.8	acyl-Coenzyme A oxidase 2, branched chain
918	Usp53	ENSMUST00000090379.2	ubiquitin specific peptidase 53
919	Camk1g	ENSMUST00000016323.5	calcium/calmodulin-dependent protein kinase I gamma
920	Tex13	ENSMUST00000044179.7	testis expressed gene 13
921	Rap2a	ENSMUST00000062117.7	RAS related protein 2a
922	Gmeb1	ENSMUST00000168553.2	glucocorticoid modulatory element binding protein 1
923	Metap1	ENSMUST00000029804.8	methionyl aminopeptidase 1
924	B630019K06Rik	ENSMUST00000064196.4	RIKEN cDNA B630019K06 gene
925	Hltf	ENSMUST00000002502.6	helicase-like transcription factor
926	Fsd1l	ENSMUST00000132151.2	fibronectin type III and SPRY domain containing 1-like
927	Uchl1	ENSMUST00000031131.9	ubiquitin carboxy-terminal hydrolase L1
928	Fam149b	ENSMUST00000090503.5	family with sequence similarity 149, member B
929	Mipol1	ENSMUST00000123498.2	mirror-image polydactyly gene 1 homolog (human)
930	Armcx2	ENSMUST00000119010.2	armadillo repeat containing, X-linked 2
931	Elk3	ENSMUST00000008542.6	ELK3, member of ETS oncogene family
932	Acly	ENSMUST00000107389.2	ATP citrate lyase
933	Calcrl	ENSMUST00000074262.3	calcitonin receptor-like
934	Ftsj1	ENSMUST00000033513.4	FtsJ homolog 1 (E. coli)
935	Rbm25	ENSMUST00000048155.10	RNA binding motif protein 25
936	Herpud2	ENSMUST00000008573.7	HERPUD family member 2
937	Ccdc177	ENSMUST00000073251.6	coiled-coil domain containing 177
938	Pabpc2	ENSMUST00000063219.2	poly(A) binding protein, cytoplasmic 2
939	Uox	ENSMUST00000121133.1	urate oxidase
940	Ppp1cb	ENSMUST00000015100.9	protein phosphatase 1, catalytic subunit, beta isoform
941	Pigm	ENSMUST00000052455.2	phosphatidylinositol glycan anchor biosynthesis, class M
942	Usp12	ENSMUST00000085614.5	ubiquitin specific peptidase 12
943	D230025D16Rik	ENSMUST00000034361.4	RIKEN cDNA D230025D16 gene
944	Itm2b	ENSMUST00000022704.7	integral membrane protein 2B
945	Otud6a	ENSMUST00000060241.2	OTU domain containing 6A
946	Gm3259	ENSMUST00000101015.4	predicted gene 3259
947	Kctd4	ENSMUST00000050120.2	potassium channel tetramerisation domain containing 4
948	Matr3	ENSMUST00000166793.2	matrin 3

949	Wdr41	ENSMUST00000056512.8	WD repeat domain 41
950	Tmem33	ENSMUST00000037918.6	transmembrane protein 33
951	Pigw	ENSMUST00000067058.2	phosphatidylinositol glycan anchor biosynthesis, class W
952	Nr2c2ap	ENSMUST00000095273.5	nuclear receptor 2C2-associated protein
953	Cmtr2	ENSMUST00000056972.5	cap methyltransferase 2
954	Tmc7	ENSMUST00000044195.4	transmembrane channel-like gene family 7
955	3110082I17Rik	ENSMUST00000066052.7	RIKEN cDNA 3110082I17 gene
956	Mcur1	ENSMUST00000021800.4	mitochondrial calcium uniporter regulator 1
957	Dclre1c	ENSMUST00000102988.4	DNA cross-link repair 1C, PSO2 homolog (S. cerevisiae)
958	Kynu	ENSMUST00000028223.3	kynureninase (L-kynurenine hydrolase)
959	Ogdhl	ENSMUST00000022480.7	oxoglutarate dehydrogenase-like
960	4921501E09Rik	ENSMUST00000024121.4	RIKEN cDNA 4921501E09 gene
961	Myadm	ENSMUST00000164553.2	myeloid-associated differentiation marker
962	Mb21d2	ENSMUST00000100023.1	Mab-21 domain containing 2
963	Dnaja2	ENSMUST00000034138.5	DnaJ (Hsp40) homolog, subfamily A, member 2
964	Pbx3	ENSMUST00000113132.3	pre B cell leukemia homeobox 3
965	Pou3f2	ENSMUST00000178174.2	POU domain, class 3, transcription factor 2
966	Ptgs2	ENSMUST00000035065.8	prostaglandin-endoperoxide synthase 2
967	Dnaja3	ENSMUST00000060067.6	DnaJ (Hsp40) homolog, subfamily A, member 3
968	1110032F04Rik	ENSMUST00000054551.2	RIKEN cDNA 1110032F04 gene
969	Ppp1r3f	ENSMUST00000150787.2	protein phosphatase 1, regulatory (inhibitor) subunit 3F
970	Gm21743	ENSMUST00000187504.1	predicted gene, 21743
971	Cpeb3	ENSMUST00000079754.5	cytoplasmic polyadenylation element binding protein 3
972	Ufm1	ENSMUST00000146598.2	ubiquitin-fold modifier 1
973	Klrb1c	ENSMUST00000167691.3	killer cell lectin-like receptor subfamily B member 1C
974	Alkbh5	ENSMUST00000044250.3	alkB, alkylation repair homolog 5 (E. coli)
975	Atxn3	ENSMUST00000021606.6	ataxin 3
976	Svil	ENSMUST00000143254.2	supervillin
977	Ampd3	ENSMUST00000005829.7	adenosine monophosphate deaminase 3
978	E2f2	ENSMUST00000061721.5	E2F transcription factor 2
979	Cgn	ENSMUST00000107273.2	cingulin
980	Cldn8	ENSMUST00000049697.4	claudin 8
981	1110051M20Rik	ENSMUST00000102594.5	RIKEN cDNA 1110051M20 gene
982	Tdp2	ENSMUST00000038039.2	tyrosyl-DNA phosphodiesterase 2
983	Anln	ENSMUST00000040912.7	anillin, actin binding protein
984	Adam9	ENSMUST00000084032.5	a disintegrin and metallopeptidase domain 9 (meltrin gamma)
985	Cbfb	ENSMUST00000052209.3	core binding factor beta
986	Agtr1a	ENSMUST00000066412.7	angiotensin II receptor, type 1a
987	Vstm2a	ENSMUST00000109645.3	V-set and transmembrane domain containing 2A
988	Cdk13	ENSMUST00000042365.7	cyclin-dependent kinase 13
989	Ctxn3	ENSMUST00000091892.2	cortexin 3
990	Chrm5	ENSMUST00000099589.3	cholinergic receptor, muscarinic 5
991	Rassf3	ENSMUST00000026902.7	Ras association (RalGDS/AF-6) domain family member 3
992	Fam199x	ENSMUST00000047852.7	family with sequence similarity 199, X-linked
993	Gm16432	ENSMUST00000094273.4	predicted gene 16432

994	Creb1	ENSMUST00000087366.5	cAMP responsive element binding protein 1
995	Fam81a	ENSMUST00000034749.9	family with sequence similarity 81, member A
996	Slc25a17	ENSMUST00000023040.7	solute carrier family 25 (mitochondrial carrier, peroxisomal membrane protein), member 17
997	Kpnb1	ENSMUST00000001479.4	karyopherin (importin) beta 1
998	9530068E07Rik	ENSMUST00000109057.2	RIKEN cDNA 9530068E07 gene
999	D1Ertd622e	ENSMUST00000053033.8	DNA segment, Chr 1, ERATO Doi 622, expressed
1000	Med22	ENSMUST00000102899.4	mediator complex subunit 22
1001	Crebzf	ENSMUST00000107205.1	CREB/ATF bZIP transcription factor
1002	Grsf1	ENSMUST00000078945.6	G-rich RNA sequence binding factor 1
1003	Ppp4r2	ENSMUST00000063854.4	protein phosphatase 4, regulatory subunit 2
1004	Pou2af1	ENSMUST00000034554.7	POU domain, class 2, associating factor 1
1005	Oas1g	ENSMUST00000086368.6	2'-5' oligoadenylate synthetase 1G
1006	A730017C20Rik	ENSMUST00000165666.3	RIKEN cDNA A730017C20 gene
1007	Fign	ENSMUST00000131615.3	fidgetin
1008	Brwd3	ENSMUST00000150434.2	bromodomain and WD repeat domain containing 3
1009	Stk4	ENSMUST00000018353.8	serine/threonine kinase 4
1010	Emr1	ENSMUST00000086763.6	EGF-like module containing, mucin-like, hormone receptor-like sequence 1
1011	Pum2	ENSMUST00000178015.2	pumilio RNA-binding family member 2
1012	Pcmt1	ENSMUST00000159917.2	protein-L-isoaspartate (D-aspartate) O-methyltransferase 1
1013	Prr11	ENSMUST00000051395.8	proline rich 11
1014	Spidr	ENSMUST00000040248.7	scaffolding protein involved i DNA repair
1015	Mixl1	ENSMUST00000027778.7	Mix1 homeobox-like 1 (Xenopus laevis)
1016	Prkcd	ENSMUST00000112210.4	protein kinase C, delta
1017	Itm2a	ENSMUST00000033591.5	integral membrane protein 2A
1018	Abcg2	ENSMUST00000114294.2	ATP-binding cassette, sub-family G (WHITE), member 2
1019	Ceacam19	ENSMUST00000052605.7	carcinoembryonic antigen-related cell adhesion molecule 19
1020	Abcb1a	ENSMUST00000047753.4	ATP-binding cassette, sub-family B (MDR/TAP), member 1A
1021	Sft2d3	ENSMUST00000054984.6	SFT2 domain containing 3
1022	Scd3	ENSMUST00000026220.5	stearoyl-coenzyme A desaturase 3
1023	Crls1	ENSMUST00000028835.7	cardiolipin synthase 1
1024	Creb5	ENSMUST00000047450.7	cAMP responsive element binding protein 5
1025	Tmem196	ENSMUST00000058644.9	transmembrane protein 196
1026	Gbp9	ENSMUST00000031238.7	guanylate-binding protein 9
1027	Tceal3	ENSMUST00000060904.5	transcription elongation factor A (SII)-like 3
1028	Cwc25	ENSMUST00000018685.3	CWC25 spliceosome-associated protein homolog (S. cerevisiae)
1029	Mysm1	ENSMUST00000075872.3	myb-like, SWIRM and MPN domains 1
1030	Sspn	ENSMUST00000111702.2	sarcospan
1031	Tle4	ENSMUST00000052011.8	transducin-like enhancer of split 4, homolog of Drosophila E(spl)
1032	Ets2	ENSMUST00000023612.9	E26 avian leukemia oncogene 2, 3' domain
1033	B430305J03Rik	ENSMUST00000066298.2	RIKEN cDNA B430305J03 gene
1034	Ints2	ENSMUST00000018212.7	integrator complex subunit 2
1035	3110043O21Rik	ENSMUST00000084724.4	RIKEN cDNA 3110043O21 gene
1036	Inhbb	ENSMUST00000038765.5	inhibin beta-B
1037	Fam216b	ENSMUST00000066437.4	family with sequence similarity 216, member B

1038	Htra4	ENSMUST00000084031.4	HtrA serine peptidase 4
1039	Elmod2	ENSMUST00000177594.2	ELMO/CED-12 domain containing 2
1040	Gimap3	ENSMUST00000038811.8	GTPase, IMAP family member 3
1041	Synj2bp	ENSMUST00000163402.2	synaptojanin 2 binding protein
1042	Farp2	ENSMUST00000122402.2	FERM, RhoGEF and pleckstrin domain protein 2
1043	Skida1	ENSMUST00000091420.5	SKI/DACH domain containing 1
1044	Txlnb	ENSMUST00000037964.6	taxilin beta
1045	Tceal6	ENSMUST00000033783.1	transcription elongation factor A (SII)-like 6
1046	2210408I21Rik	ENSMUST00000168779.1	RIKEN cDNA 2210408I21 gene
1047	Kcnd3	ENSMUST00000098761.4	potassium voltage-gated channel, Shal-related family, member 3
1048	Hemt1	ENSMUST00000188180.1	hematopoietic cell transcript 1
1049	Gm15023	ENSMUST00000113183.1	predicted gene 15023
1050	AV320801	ENSMUST00000068340.2	expressed sequence AV320801
1051	Brs3	ENSMUST00000033464.3	bombesin-like receptor 3
1052	Aldh9a1	ENSMUST00000028004.9	aldehyde dehydrogenase 9, subfamily A1
1053	Spcs3	ENSMUST00000067476.8	signal peptidase complex subunit 3 homolog (S. cerevisiae)
1054	Tmem106b	ENSMUST00000031556.8	transmembrane protein 106B
1055	A630007B06Rik	ENSMUST00000118592.2	RIKEN cDNA A630007B06 gene
1056	Acvr1b	ENSMUST00000000544.10	activin A receptor, type 1B
1057	Rbbp6	ENSMUST00000052135.8	retinoblastoma binding protein 6
1058	Ldlrad4	ENSMUST00000063775.3	low density lipoprotein receptor class A domain containing 4
1059	Uggt2	ENSMUST00000156203.2	UDP-glucose glycoprotein glucosyltransferase 2
1060	Stx11	ENSMUST00000163425.2	syntaxin 11
1061	Aldh1a3	ENSMUST00000015278.8	aldehyde dehydrogenase family 1, subfamily A3
1062	Gm14288	ENSMUST00000123863.1	predicted gene 14288
1063	Taf13	ENSMUST00000143054.1	TAF13 RNA polymerase II, TATA box binding protein (TBP)-associated factor
1064	Muc13	ENSMUST00000023520.6	mucin 13, epithelial transmembrane
1065	Pank1	ENSMUST00000036584.6	pantothenate kinase 1
1066	Fam228a	ENSMUST00000111154.2	family with sequence similarity 228, member A
1067	Lats1	ENSMUST00000165952.2	large tumor suppressor
1068	Cacul1	ENSMUST00000081790.8	CDK2 associated, cullin domain 1
1069	Uxs1	ENSMUST00000126008.2	UDP-glucuronate decarboxylase 1
1070	Klhl28	ENSMUST00000021331.7	kelch-like 28
1071	Lnpep	ENSMUST00000041047.2	leucyl/cystinyl aminopeptidase
1072	mt-Nd2	ENSMUST00000082396.1	mitochondrially encoded NADH dehydrogenase 2
1073	Kank4	ENSMUST00000102790.3	KN motif and ankyrin repeat domains 4
1074	Pcca	ENSMUST00000038374.7	propionyl-Coenzyme A carboxylase, alpha polypeptide
1075	AI317395	ENSMUST00000163705.2	expressed sequence AI317395
1076	Tmem55a	ENSMUST00000029875.3	transmembrane protein 55A
1077	Gclc	ENSMUST00000034905.8	glutamate-cysteine ligase, catalytic subunit
1078	Rbfox1	ENSMUST00000056416.7	RNA binding protein, fox-1 homolog (C. elegans) 1
1079	Rsf1	ENSMUST00000042399.8	remodeling and spacing factor 1
1080	Dsp	ENSMUST00000127906.2	desmoplakin
1081	Mgat2	ENSMUST00000060579.9	mannoside acetylglucosaminyltransferase 2
1082	Gm4980	ENSMUST00000178124.2	predicted gene 4980
1083	Pla2g16	ENSMUST00000025925.5	phospholipase A2, group XVI
1084	Dbt	ENSMUST00000000349.6	dihydrolipoamide branched chain transacylase E2

1085	Fam126a	ENSMUST00000030849.7	family with sequence similarity 126, member A
1086	Apol8	ENSMUST00000089450.3	apolipoprotein L 8
1087	Zfp937	ENSMUST00000073782.3	zinc finger protein 937
1088	Lpp	ENSMUST00000038053.7	LIM domain containing preferred translocation partner in lipoma
1089	A330021E22Rik	ENSMUST00000054865.7	RIKEN cDNA A330021E22 gene
1090	Bcl2l13	ENSMUST00000009256.2	BCL2-like 13 (apoptosis facilitator)
1091	Anxa7	ENSMUST00000065504.10	annexin A7
1092	Ube2d2a	ENSMUST00000170693.2	ubiquitin-conjugating enzyme E2D 2A
1093	Galnt13	ENSMUST00000112636.2	UDP-N-acetyl-alpha-D-galactosamine:polypeptide N-acetylgalactosaminyltransferase 13
1094	Slc36a4	ENSMUST00000061568.7	solute carrier family 36 (proton/amino acid symporter), member 4
1095	Car12	ENSMUST00000071889.7	carbonic anyhydrase 12
1096	Synrg	ENSMUST00000092834.6	synergin, gamma
1097	Naa11	ENSMUST00000060265.4	N(alpha)-acetyltransferase 11, NatA catalytic subunit
1098	Tmem170b	ENSMUST00000129449.1	transmembrane protein 170B
1099	Nfx1	ENSMUST00000098143.5	nuclear transcription factor, X-box binding 1
1100	Rasa1	ENSMUST00000109552.2	RAS p21 protein activator 1
1101	Napg	ENSMUST00000025474.7	N-ethylmaleimide sensitive fusion protein attachment protein gamma
1102	Lysmd3	ENSMUST00000049055.6	LysM, putative peptidoglycan-binding, domain containing 3
1103	Pdk3	ENSMUST00000045748.6	pyruvate dehydrogenase kinase, isoenzyme 3
1104	Slc7a12	ENSMUST00000037321.7	solute carrier family 7 (cationic amino acid transporter, y+ system), member 12
1105	Bai3	ENSMUST00000126626.2	brain-specific angiogenesis inhibitor 3
1106	Cd200r3	ENSMUST00000114622.4	CD200 receptor 3
1107	Tmem71	ENSMUST00000048372.5	transmembrane protein 71
1108	Tmem233	ENSMUST00000111997.1	transmembrane protein 233
1109	Scin	ENSMUST00000078481.8	scinderin
1110	Ell2	ENSMUST00000001583.6	elongation factor RNA polymerase II 2
1111	Runx1t1	ENSMUST00000006761.4	runt-related transcription factor 1; translocated to, 1 (cyclin D-related)
1112	Dpp6	ENSMUST00000120555.2	dipeptidylpeptidase 6
1113	Vmn2r42	ENSMUST00000142934.1	vomeronasal 2, receptor 42
1114	Sfrp2	ENSMUST00000029625.7	secreted frizzled-related protein 2
1115	Esrrg	ENSMUST00000027906.7	estrogen-related receptor gamma
1116	Zrsr2	ENSMUST00000090840.4	zinc finger (CCCH type), RNA binding motif and serine/arginine rich 2
1117	Cerkl	ENSMUST00000143974.1	ceramide kinase-like
1118	Dennd2c	ENSMUST00000173206.2	DENN/MADD domain containing 2C
1119	Sh3bgrl2	ENSMUST00000113215.4	SH3 domain binding glutamic acid-rich protein like 2
1120	St8sia6	ENSMUST00000003509.8	ST8 alpha-N-acetyl-neuraminide alpha-2,8-sialyltransferase 6
1121	Tnpo1	ENSMUST00000109399.3	transportin 1
1122	Armc10	ENSMUST00000095495.2	armadillo repeat containing 10
1123	Mpv17l	ENSMUST00000128757.2	Mpv17 transgene, kidney disease mutant-like
1124	Fcho2	ENSMUST00000099277.5	FCH domain only 2
1125	App	ENSMUST00000005406.10	amyloid beta (A4) precursor protein
1126	BC025446	ENSMUST00000185307.1	cDNA sequence BC025446
1127	Far1	ENSMUST00000033018.9	fatty acyl CoA reductase 1

1128	Arf2	ENSMUST00000057921.4	ADP-ribosylation factor 2
1129	Tslp	ENSMUST00000025237.3	thymic stromal lymphopoietin
1130	Popdc2	ENSMUST00000023494.7	popeye domain containing 2
1131	Cbll1	ENSMUST00000064240.7	Casitas B-lineage lymphoma-like 1
1132	Eif4e3	ENSMUST00000032151.2	eukaryotic translation initiation factor 4E member 3
1133	Frem1	ENSMUST00000170248.3	Fras1 related extracellular matrix protein 1
1134	Appl1	ENSMUST00000036570.4	adaptor protein, phosphotyrosine interaction, PH domain and leucine zipper containing 1
1135	Psg16	ENSMUST00000071399.7	pregnancy specific glycoprotein 16
1136	Tmem245	ENSMUST00000068792.7	transmembrane protein 245
1137	Nxpe2	ENSMUST00000034527.8	neurexophilin and PC-esterase domain family, member 2
1138	Zrsr1	ENSMUST00000049506.6	zinc finger (CCCH type), RNA binding motif and serine/arginine rich 1
1139	Sgpp2	ENSMUST00000036172.9	sphingosine-1-phosphate phosphotase 2
1140	Golt1b	ENSMUST00000032372.6	golgi transport 1 homolog B (S. cerevisiae)
1141	Epm2aip1	ENSMUST00000060711.6	EPM2A (laforin) interacting protein 1
1142	Tardbp	ENSMUST00000084125.4	TAR DNA binding protein
1143	Zscan4c	ENSMUST00000131379.2	zinc finger and SCAN domain containing 4C
1144	Grm6	ENSMUST00000171427.2	glutamate receptor, metabotropic 6
1145	Kcnh7	ENSMUST00000075052.4	potassium voltage-gated channel, subfamily H (eag-related), member 7
1146	Gm8909	ENSMUST00000173353.2	predicted gene 8909
1147	Slc4a1ap	ENSMUST00000117262.2	solute carrier family 4 (anion exchanger), member 1, adaptor protein
1148	F5	ENSMUST00000086040.5	coagulation factor V
1149	Kitl	ENSMUST00000105283.2	kit ligand
1150	Zfp14	ENSMUST00000077787.7	zinc finger protein 14
1151	Inpp4a	ENSMUST00000027287.5	inositol polyphosphate-4-phosphatase, type I
1152	Amigo2	ENSMUST00000053106.5	adhesion molecule with Ig like domain 2
1153	Arl8b	ENSMUST00000032196.6	ADP-ribosylation factor-like 8B
1154	Map4k5	ENSMUST00000110567.2	mitogen-activated protein kinase kinase kinase kinase 5
1155	Gm16485	ENSMUST00000104915.2	predicted gene 16485
1156	Impa2	ENSMUST00000025403.6	inositol (myo)-1(or 4)-monophosphatase 2
1157	Slc35f6	ENSMUST00000062962.6	solute carrier family 35, member F6
1158	Slc16a7	ENSMUST00000063318.3	solute carrier family 16 (monocarboxylic acid transporters), member 7
1159	Arv1	ENSMUST00000034463.3	ARV1 homolog (yeast)
1160	Kctd8	ENSMUST00000054095.4	potassium channel tetramerisation domain containing 8
1161	Ktn1	ENSMUST00000022391.8	kinectin 1
1162	Tmem206	ENSMUST00000027940.5	transmembrane protein 206
1163	Mbtps2	ENSMUST00000058098.9	membrane-bound transcription factor peptidase, site 2
1164	Hipk3	ENSMUST00000028600.8	homeodomain interacting protein kinase 3
1165	Xrcc2	ENSMUST00000030773.7	X-ray repair complementing defective repair in Chinese hamster cells 2
1166	Tom1l1	ENSMUST00000020849.3	target of myb1-like 1 (chicken)
1167	Wnk1	ENSMUST00000060043.7	WNK lysine deficient protein kinase 1
1168	Zfp605	ENSMUST00000112528.2	zinc finger protein 605
1169	Pla1a	ENSMUST00000002926.6	phospholipase A1 member A
1170	Htr3a	ENSMUST00000003826.7	5-hydroxytryptamine (serotonin) receptor 3A

1171	Mrpl53	ENSMUST00000113938.3	mitochondrial ribosomal protein L53
1172	Slit2	ENSMUST00000170109.3	slit homolog 2 (Drosophila)
1173	Rp1l1	ENSMUST00000058229.4	retinitis pigmentosa 1 homolog (human)-like 1
1174	Gm12353	ENSMUST00000108250.2	predicted gene 12353
1175	Tmtc3	ENSMUST00000058154.9	transmembrane and tetratricopeptide repeat containing 3
1176	Mier1	ENSMUST00000106858.2	mesoderm induction early response 1 homolog (Xenopus laevis
1177	Hivep2	ENSMUST00000191138.1	human immunodeficiency virus type I enhancer binding protein 2
1178	Uri1	ENSMUST00000085513.4	URI1, prefoldin-like chaperone
1179	Arhgef6	ENSMUST00000033468.5	Rac/Cdc42 guanine nucleotide exchange factor (GEF) 6
1180	Serp1	ENSMUST00000029385.7	stress-associated endoplasmic reticulum protein 1
1181	Acvr2a	ENSMUST00000063886.3	activin receptor IIA
1182	Tead3	ENSMUST00000154873.2	TEA domain family member 3
1183	4933402D24Rik	ENSMUST00000027102.1	RIKEN cDNA 4933402D24 gene
1184	Olfr536	ENSMUST00000064392.6	olfactory receptor 536
1185	Zbtbd6	ENSMUST00000100359.1	kelch repeat and BTB (POZ) domain containing 6
1186	Tbcd	ENSMUST00000103013.4	tubulin-specific chaperone d
1187	Timm8a1	ENSMUST00000054213.4	translocase of inner mitochondrial membrane 8A1
1188	Pafah1b2	ENSMUST00000172450.1	platelet-activating factor acetylhydrolase, isoform 1b, subunit 2
1189	Zfp945	ENSMUST00000160457.3	zinc finger protein 945
1190	Gm20425	ENSMUST00000166836.1	predicted gene 20425
1191	Lgals8	ENSMUST00000124888.2	lectin, galactose binding, soluble 8
1192	Trim2	ENSMUST00000107692.2	tripartite motif-containing 2
1193	Slc38a11	ENSMUST00000112420.2	solute carrier family 38, member 11
1194	Ppp1r2	ENSMUST00000060188.8	protein phosphatase 1, regulatory (inhibitor) subunit 2
1195	Dock4	ENSMUST00000037488.6	dedicator of cytokinesis 4
1196	Sall4	ENSMUST00000075044.4	sal-like 4 (Drosophila)
1197	Pik3cg	ENSMUST00000053215.8	phosphoinositide-3-kinase, catalytic, gamma polypeptide
1198	Phactr2	ENSMUST00000105545.5	phosphatase and actin regulator 2
1199	Insm1	ENSMUST00000089257.5	insulinoma-associated 1
1200	C1ql3	ENSMUST00000061545.5	C1q-like 3
1201	Rapgefl1	ENSMUST00000107479.2	Rap guanine nucleotide exchange factor (GEF)-like 1
1202	Ticam2	ENSMUST00000070084.4	toll-like receptor adaptor molecule 2
1203	Senp8	ENSMUST00000163586.2	SUMO/sentrin specific peptidase 8
1204	Sh3gl2	ENSMUST00000030212.9	SH3-domain GRB2-like 2
1205	Bmpr1b	ENSMUST00000029948.9	bone morphogenetic protein receptor, type 1B
1206	Tsn	ENSMUST00000027623.8	translin
1207	Btla	ENSMUST00000063654.4	B and T lymphocyte associated
1208	Lsamp	ENSMUST00000099761.4	limbic system-associated membrane protein
1209	Pdpk1	ENSMUST00000102927.4	3-phosphoinositide dependent protein kinase 1
1210	Zfp235	ENSMUST00000056549.7	zinc finger protein 235
1211	Bag3	ENSMUST00000033136.7	BCL2-associated athanogene 3
1212	Btbd19	ENSMUST00000183310.1	BTB (POZ) domain containing 19
1213	Oxgr1	ENSMUST00000058213.5	oxoglutarate (alpha-ketoglutarate) receptor 1
1214	Cenpm	ENSMUST00000089157.4	centromere protein M
1215	1810011H11Rik	ENSMUST00000039191.6	RIKEN cDNA 1810011H11 gene

1216	Zdhhc21	ENSMUST00000030110.9	zinc finger, DHHC domain containing 21
1217	Galr1	ENSMUST00000065224.6	galanin receptor 1
1218	Sacm1l	ENSMUST00000026270.7	SAC1 (suppressor of actin mutations 1, homolog)-like (S. cerevisiae)
1219	Naf1	ENSMUST00000118009.1	nuclear assembly factor 1 homolog (S. cerevisiae)
1220	Trnt1	ENSMUST00000057578.10	tRNA nucleotidyl transferase, CCA-adding, 1
1221	Rac1	ENSMUST00000080537.8	RAS-related C3 botulinum substrate 1
1222	A830073O21Rik	ENSMUST00000169090.1	RIKEN cDNA A830073O21 gene
1223	Aadat	ENSMUST00000079472.2	aminoadipate aminotransferase
1224	Fbxo11	ENSMUST00000005504.9	F-box protein 11
1225	Plek	ENSMUST00000102881.4	pleckstrin
1226	Bcr	ENSMUST00000164107.1	breakpoint cluster region
1227	Sgcd	ENSMUST00000077221.5	sarcoglycan, delta (dystrophin-associated glycoprotein)
1228	AI314180	ENSMUST00000102889.4	expressed sequence AI314180
1229	Dclk1	ENSMUST00000167204.2	doublecortin-like kinase 1
1230	Brca2	ENSMUST00000044620.7	breast cancer 2
1231	Sorcs1	ENSMUST00000164039.3	VPS10 domain receptor protein SORCS 1
1232	Cast	ENSMUST00000065629.4	calpastatin
1233	Smurf2	ENSMUST00000103067.4	SMAD specific E3 ubiquitin protein ligase 2
1234	Nus1	ENSMUST00000023830.9	nuclear undecaprenyl pyrophosphate synthase 1 homolog (S. cerevisiae)
1235	Zic3	ENSMUST00000088627.5	zinc finger protein of the cerebellum 3
1236	Bag1	ENSMUST00000108089.2	BCL2-associated athanogene 1
1237	Gng4	ENSMUST00000021734.7	guanine nucleotide binding protein (G protein), gamma 4
1238	P2ry1	ENSMUST00000029331.1	purinergic receptor P2Y, G-protein coupled 1
1239	Rundc3b	ENSMUST00000047485.9	RUN domain containing 3B
1240	Slc9a7	ENSMUST00000072451.5	solute carrier family 9 (sodium/hydrogen exchanger), member 7
1241	Per3	ENSMUST00000103204.5	period circadian clock 3
1242	Klf5	ENSMUST00000005279.6	Kruppel-like factor 5
1243	Zcchc2	ENSMUST00000119166.2	zinc finger, CCHC domain containing 2
1244	2810417H13Rik	ENSMUST00000045802.6	RIKEN cDNA 2810417H13 gene
1245	Zscan4d	ENSMUST00000067210.5	zinc finger and SCAN domain containing 4D
1246	Twsg1	ENSMUST00000024906.4	twisted gastrulation homolog 1 (Drosophila)
1247	Rnf128	ENSMUST00000113026.1	ring finger protein 128
1248	Ncam2	ENSMUST00000037785.8	neural cell adhesion molecule 2
1249	Sema4d	ENSMUST00000110040.3	sema domain, immunoglobulin domain (Ig), transmembrane domain (TM) and short cytoplasmic domain, (semaphorin) 4D
1250	Tiam1	ENSMUST00000114122.2	T cell lymphoma invasion and metastasis 1
1251	Rsbn1l	ENSMUST00000036489.5	round spermatid basic protein 1-like
1252	Gm3646	ENSMUST00000171319.2	predicted gene 3646
1253	Efnb2	ENSMUST00000001319.8	ephrin B2
1254	Zfp114	ENSMUST00000086010.5	zinc finger protein 114
1255	Acmsd	ENSMUST00000038006.7	amino carboxymuconate semialdehyde decarboxylase
1256	Ubxn10	ENSMUST00000105811.3	UBX domain protein 10
1257	Ccrn4l	ENSMUST00000023849.9	CCR4 carbon catabolite repression 4-like (S. cerevisiae)
1258	Pyhin1	ENSMUST00000056071.7	pyrin and HIN domain family, member 1
1259	Fam198b	ENSMUST00000029567.8	family with sequence similarity 198, member B

1260	Gm26596	ENSMUST00000180464.1	predicted gene, 26596
1261	Hmbox1	ENSMUST00000022544.8	homeobox containing 1
1262	Pkib	ENSMUST00000075992.6	protein kinase inhibitor beta, cAMP dependent, testis specific
1263	Corin	ENSMUST00000167460.3	corin
1264	Pik3ca	ENSMUST00000029201.8	phosphatidylinositol 3-kinase, catalytic, alpha polypeptide
1265	Pid1	ENSMUST00000168574.3	phosphotyrosine interaction domain containing 1
1266	Rftn1	ENSMUST00000044503.8	raftlin lipid raft linker 1
1267	Myh10	ENSMUST00000102611.4	myosin, heavy polypeptide 10, non-muscle
1268	Atp7a	ENSMUST00000055941.6	ATPase, Cu++ transporting, alpha polypeptide
1269	Aldh7a1	ENSMUST00000174518.2	aldehyde dehydrogenase family 7, member A1
1270	Gprc5b	ENSMUST00000008878.8	G protein-coupled receptor, family C, group 5, member B
1271	Smc1b	ENSMUST00000023068.6	structural maintenance of chromosomes 1B
1272	Luc7l3	ENSMUST00000107820.1	LUC7-like 3 (S. cerevisiae)
1273	Gm13288	ENSMUST00000094977.1	predicted gene 13288
1274	Pcdhb5	ENSMUST00000078271.2	protocadherin beta 5
1275	Otud4	ENSMUST00000173078.2	OTU domain containing 4
1276	Cnih1	ENSMUST00000015903.5	cornichon homolog 1 (Drosophila)
1277	Lsm1	ENSMUST00000038421.6	LSM1 homolog, U6 small nuclear RNA associated (S. cerevisiae)
1278	Tcf7l2	ENSMUST00000111656.2	transcription factor 7 like 2, T cell specific, HMG box
1279	Yod1	ENSMUST00000049813.5	YOD1 OTU deubiquitinating enzyme 1 homologue (S. cerevisiae)
1280	Gm17365	ENSMUST00000178236.1	predicted gene, 17365
1281	Slc32a1	ENSMUST00000045738.4	solute carrier family 32 (GABA vesicular transporter), member 1
1282	Plcg1	ENSMUST00000109462.2	phospholipase C, gamma 1
1283	Fzd4	ENSMUST00000058755.3	frizzled homolog 4 (Drosophila)
1284	Aff1	ENSMUST00000054979.4	AF4/FMR2 family, member 1
1285	Stox2	ENSMUST00000110367.3	storkhead box 2
1286	Cetn2	ENSMUST00000114551.4	centrin 2
1287	Usp25	ENSMUST00000023580.6	ubiquitin specific peptidase 25
1288	Mtdh	ENSMUST00000022865.10	metadherin
1289	Rif1	ENSMUST00000069794.5	Rap1 interacting factor 1 homolog (yeast)
1290	Fgf1	ENSMUST00000117566.2	fibroblast growth factor 1
1291	Pilra	ENSMUST00000058897.5	paired immunoglobin-like type 2 receptor alpha
1292	Arfrp1	ENSMUST00000170190.2	ADP-ribosylation factor related protein 1
1293	Fam171a1	ENSMUST00000115099.3	family with sequence similarity 171, member A1
1294	Nap1l5	ENSMUST00000059539.3	nucleosome assembly protein 1-like 5
1295	Hyal6	ENSMUST00000031690.5	hyaluronoglucosaminidase 6
1296	Rhou	ENSMUST00000045487.3	ras homolog gene family, member U
1297	Snx31	ENSMUST00000013755.6	sorting nexin 31
1298	Slc25a36	ENSMUST00000085206.5	solute carrier family 25, member 36
1299	Adam10	ENSMUST00000067880.7	a disintegrin and metallopeptidase domain 10
1300	Psmb11	ENSMUST00000097177.4	proteasome (prosome, macropain) subunit, beta type, 11
1301	Acap2	ENSMUST00000058033.7	ArfGAP with coiled-coil, ankyrin repeat and PH domains 2
1302	Gm13242	ENSMUST00000105738.3	predicted gene 13242

1303	Pcdh8	ENSMUST00000039568.5	protocadherin 8
1304	Cd24a	ENSMUST00000058714.8	CD24a antigen
1305	Sim1	ENSMUST00000020071.3	single-minded homolog 1 (Drosophila)
1306	Jakmip2	ENSMUST00000082254.6	janus kinase and microtubule interacting protein 2
1307	Dazap2	ENSMUST00000000356.8	DAZ associated protein 2
1308	Trio	ENSMUST00000090247.5	triple functional domain (PTPRF interacting)
1309	Cep135	ENSMUST00000121979.2	centrosomal protein 135
1310	Rap1a	ENSMUST00000090678.6	RAS-related protein-1a
1311	Il17rb	ENSMUST00000122205.2	interleukin 17 receptor B
1312	Pde1c	ENSMUST00000166890.2	phosphodiesterase 1C
1313	Dkk2	ENSMUST00000029665.5	dickkopf homolog 2 (Xenopus laevis)
1314	Gdap2	ENSMUST00000029459.4	ganglioside-induced differentiation-associated-protein 2
1315	Rnf217	ENSMUST00000081989.7	ring finger protein 217
1316	Akr1b3	ENSMUST00000102980.5	aldo-keto reductase family 1, member B3 (aldose reductase)
1317	Fgf9	ENSMUST00000165526.2	fibroblast growth factor 9
1318	Gpr149	ENSMUST00000058535.5	G protein-coupled receptor 149
1319	Pdpn	ENSMUST00000030317.8	podoplanin
1320	Slc4a10	ENSMUST00000102735.4	solute carrier family 4, sodium bicarbonate cotransporter-like, member 10
1321	Rnf125	ENSMUST00000050004.1	ring finger protein 125
1322	Vps4b	ENSMUST00000094646.5	vacuolar protein sorting 4b (yeast)
1323	Gtf2h1	ENSMUST00000006774.5	general transcription factor II H, polypeptide 1
1324	H2-Ob	ENSMUST00000095342.4	histocompatibility 2, O region beta locus
1325	Vasp	ENSMUST00000032561.8	vasodilator-stimulated phosphoprotein
1326	Ptprb	ENSMUST00000092167.5	protein tyrosine phosphatase, receptor type, B
1327	Cradd	ENSMUST00000053594.5	CASP2 and RIPK1 domain containing adaptor with death domain
1328	Hcls1	ENSMUST00000023531.9	hematopoietic cell specific Lyn substrate 1
1329	Slitrk6	ENSMUST00000078386.2	SLIT and NTRK-like family, member 6
1330	Gm1564	ENSMUST00000100378.3	predicted gene 1564
1331	Pask	ENSMUST00000027493.3	PAS domain containing serine/threonine kinase
1332	Tbx2	ENSMUST00000000095.6	T-box 2
1333	Srp72	ENSMUST00000101087.4	signal recognition particle 72
1334	Marcks	ENSMUST00000092584.5	myristoylated alanine rich protein kinase C substrate
1335	Amdhd1	ENSMUST00000016034.2	amidohydrolase domain containing 1
1336	Abca2	ENSMUST00000102919.3	ATP-binding cassette, sub-family A (ABC1), member 2
1337	Dcaf10	ENSMUST00000155551.2	DDB1 and CUL4 associated factor 10
1338	Qser1	ENSMUST00000117237.1	glutamine and serine rich 1
1339	Palm3	ENSMUST00000055077.6	paralemmin 3
1340	H2bfm	ENSMUST00000059808.4	H2B histone family, member M
1341	Uqcrc2	ENSMUST00000033176.5	ubiquinol cytochrome c reductase core protein 2
1342	Napepld	ENSMUST00000115217.2	N-acyl phosphatidylethanolamine phospholipase D
1343	Serf1	ENSMUST00000132053.2	small EDRK-rich factor 1
1344	Ube2v2	ENSMUST00000115777.4	ubiquitin-conjugating enzyme E2 variant 2
1345	Prlr	ENSMUST00000128921.2	prolactin receptor
1346	Nkain3	ENSMUST00000119374.2	Na+/K+ transporting ATPase interacting 3
1347	Adcy1	ENSMUST00000020706.4	adenylate cyclase 1
1348	Plk3	ENSMUST00000076859.6	polo-like kinase 3
1349	Xlr	ENSMUST00000067782.2	X-linked lymphocyte-regulated

1350	Cep170b	ENSMUST00000101018.4	centrosomal protein 170B
1351	Cnn2	ENSMUST00000004784.5	calponin 2
1352	Atxn2	ENSMUST00000051950.8	ataxin 2
1353	Cln6	ENSMUST00000034776.7	ceroid-lipofuscinosis, neuronal 6
1354	Rab23	ENSMUST00000088287.4	RAB23, member RAS oncogene family
1355	Prdm1	ENSMUST00000039174.5	PR domain containing 1, with ZNF domain
1356	Vezf1	ENSMUST00000018521.5	vascular endothelial zinc finger 1
1357	Icmt	ENSMUST00000048892.8	isoprenylcysteine carboxyl methyltransferase
1358	7530416G11Rik	ENSMUST00000178942.1	RIKEN cDNA 7530416G11 gene
1359	Klhl41	ENSMUST00000100050.3	kelch-like 41
1360	Mbnl2	ENSMUST00000088419.7	muscleblind-like 2
1361	Me1	ENSMUST00000034989.9	malic enzyme 1, NADP(+)-dependent, cytosolic
1362	Rnf14	ENSMUST00000170811.2	ring finger protein 14
1363	Phf6	ENSMUST00000078944.7	PHD finger protein 6
1364	Kdm7a	ENSMUST00000002305.8	lysine (K)-specific demethylase 7A
1365	Ccser2	ENSMUST00000090024.5	coiled-coil serine rich 2
1366	Suco	ENSMUST00000048377.5	SUN domain containing ossification factor
1367	Acbd7	ENSMUST00000115089.1	acyl-Coenzyme A binding domain containing 7
1368	Arhgap5	ENSMUST00000110725.1	Rho GTPase activating protein 5
1369	1700013F07Rik	ENSMUST00000029485.5	RIKEN cDNA 1700013F07 gene
1370	Tmem19	ENSMUST00000092170.5	transmembrane protein 19
1371	POU2F1	ENSMUST00000111427.3	POU domain, class 2, transcription factor 1 isoform B
1372	Zfand5	ENSMUST00000025659.5	zinc finger, AN1-type domain 5
1373	Tssk4	ENSMUST00000164809.2	testis-specific serine kinase 4
1374	Pitx2	ENSMUST00000029657.10	paired-like homeodomain transcription factor 2
1375	Mbtd1	ENSMUST00000063718.5	mbt domain containing 1
1376	Oxr1	ENSMUST00000022918.8	oxidation resistance 1
1377	Gstcd	ENSMUST00000029651.5	glutathione S-transferase, C-terminal domain containing
1378	Tmem169	ENSMUST00000027380.6	transmembrane protein 169
1379	Slc9a4	ENSMUST00000027233.7	solute carrier family 9 (sodium/hydrogen exchanger), member 4
1380	Plekhs1	ENSMUST00000178590.2	pleckstrin homology domain containing, family S member 1
1381	Glrb	ENSMUST00000029654.9	glycine receptor, beta subunit
1382	Gm14226	ENSMUST00000130870.1	predicted gene 14226
1383	Thbs1	ENSMUST00000039559.8	thrombospondin 1
1384	Hemgn	ENSMUST00000107764.3	hemogen
1385	Hdhd2	ENSMUST00000150990.2	haloacid dehalogenase-like hydrolase domain containing 2
1386	F11r	ENSMUST00000043839.4	F11 receptor
1387	Sdc2	ENSMUST00000022871.5	syndecan 2
1388	Shisa3	ENSMUST00000087241.5	shisa homolog 3 (Xenopus laevis)
1389	Bdp1	ENSMUST00000038104.6	B double prime 1, subunit of RNA polymerase III transcription initiation factor IIIB
1390	Slc39a6	ENSMUST00000070726.4	solute carrier family 39 (metal ion transporter), member 6
1391	Acot4	ENSMUST00000021652.3	acyl-CoA thioesterase 4
1392	Prr15l	ENSMUST00000107636.3	proline rich 15-like
1393	Tnfrsf13b	ENSMUST00000010286.2	tumor necrosis factor receptor superfamily, member 13b

1394	Arl14epl	ENSMUST00000097580.1	ADP-ribosylation factor-like 14 effector protein-like
1395	Ccdc38	ENSMUST00000092215.5	coiled-coil domain containing 38
1396	Olfml1	ENSMUST00000120990.1	olfactomedin-like 1
1397	Plod2	ENSMUST00000070522.8	procollagen lysine, 2-oxoglutarate 5-dioxygenase 2
1398	Selt	ENSMUST00000107924.1	selenoprotein T
1399	Syne1	ENSMUST00000095899.3	spectrin repeat containing, nuclear envelope 1
1400	Gm4794	ENSMUST00000165904.1	predicted gene 4794
1401	Gm15737	ENSMUST00000113434.1	predicted gene 15737
1402	Tcam1	ENSMUST00000044462.3	testicular cell adhesion molecule 1
1403	N28178	ENSMUST00000107975.2	expressed sequence N28178
1404	Amigo1	ENSMUST00000106656.1	adhesion molecule with Ig like domain 1
1405	Sema3e	ENSMUST00000073957.6	sema domain, immunoglobulin domain (Ig), short basic domain, secreted, (semaphorin) 3E
1406	Mov10	ENSMUST00000002297.6	Moloney leukemia virus 10
1407	Tmem220	ENSMUST00000061786.5	transmembrane protein 220
1408	Npc1	ENSMUST00000025279.5	Niemann-Pick type C1
1409	Trim32	ENSMUST00000050850.8	tripartite motif-containing 32
1410	4921522P10Rik	ENSMUST00000048606.1	RIKEN cDNA 4921522P10 gene
1411	Slc26a4	ENSMUST00000001253.7	solute carrier family 26, member 4
1412	Ddhd2	ENSMUST00000033975.6	DDHD domain containing 2
1413	Dnajb9	ENSMUST00000015049.3	DnaJ (Hsp40) homolog, subfamily B, member 9
1414	Ccdc15	ENSMUST00000037275.5	coiled-coil domain containing 15
1415	Itga4	ENSMUST00000099972.4	integrin alpha 4
1416	Themis	ENSMUST00000056097.5	thymocyte selection associated
1417	Efcab1	ENSMUST00000090277.1	EF hand calcium binding domain 1
1418	Cyp24a1	ENSMUST00000038824.5	cytochrome P450, family 24, subfamily a, polypeptide 1
1419	Gopc	ENSMUST00000105475.3	golgi associated PDZ and coiled-coil motif containing
1420	Ube2l3	ENSMUST00000115699.2	ubiquitin-conjugating enzyme E2L 3
1421	A230046K03Rik	ENSMUST00000038388.5	RIKEN cDNA A230046K03 gene
1422	Dusp12	ENSMUST00000027970.8	dual specificity phosphatase 12
1423	Lrrc1	ENSMUST00000183873.2	leucine rich repeat containing 1
1424	Ptchd4	ENSMUST00000048691.4	patched domain containing 4
1425	Ksr1	ENSMUST00000018478.5	kinase suppressor of ras 1
1426	Scgb1b27	ENSMUST00000085618.4	secretoglobin, family 1B, member 27
1427	Itsn1	ENSMUST00000056482.8	intersectin 1 (SH3 domain protein 1A)
1428	Kcnj1	ENSMUST00000047334.8	potassium inwardly-rectifying channel, subfamily J, member 1
1429	Impdh1	ENSMUST00000160878.2	inosine 5'-phosphate dehydrogenase 1
1430	Zranb2	ENSMUST00000106057.2	zinc finger, RAN-binding domain containing 2
1431	Mapre1	ENSMUST00000028981.8	microtubule-associated protein, RP/EB family, member 1
1432	Fam117b	ENSMUST00000036540.6	family with sequence similarity 117, member B
1433	Golph3l	ENSMUST00000060323.6	golgi phosphoprotein 3-like
1434	Kcnj3	ENSMUST00000112632.1	potassium inwardly-rectifying channel, subfamily J, member 3
1435	Tmem47	ENSMUST00000026760.2	transmembrane protein 47
1436	Nabp1	ENSMUST00000027279.6	nucleic acid binding protein 1
1437	Oprk1	ENSMUST00000160777.2	opioid receptor, kappa 1
1438	Fxr1	ENSMUST00000001620.8	fragile X mental retardation gene 1, autosomal homolog

1439	Deptor	ENSMUST00000096433.4	DEP domain containing MTOR-interacting protein
1440	Acp1	ENSMUST00000062740.8	acid phosphatase 1, soluble
1441	Slc12a5	ENSMUST00000099092.4	solute carrier family 12, member 5
1442	Mon1b	ENSMUST00000179926.2	MON1 homolog b (yeast)
1443	1810011O10Rik	ENSMUST00000052622.5	RIKEN cDNA 1810011O10 gene
1444	Klhl31	ENSMUST00000057781.7	kelch-like 31
1445	Itga6	ENSMUST00000028522.4	integrin alpha 6
1446	Rel	ENSMUST00000102864.3	reticuloendotheliosis oncogene
1447	Cttnbp2nl	ENSMUST00000077548.6	CTTNBP2 N-terminal like
1448	Tceal1	ENSMUST00000055104.5	transcription elongation factor A (SII)-like 1
1449	Fbxo32	ENSMUST00000022986.6	F-box protein 32
1450	Rnf170	ENSMUST00000110575.2	ring finger protein 170
1451	Plxdc2	ENSMUST00000028081.7	plexin domain containing 2
1452	Ubxn4	ENSMUST00000027592.4	UBX domain protein 4
1453	Krt222	ENSMUST00000103132.4	keratin 222
1454	Mfap1a	ENSMUST00000089926.5	microfibrillar-associated protein 1A
1455	Vash2	ENSMUST00000047409.7	vasohibin 2
1456	Zdbf2	ENSMUST00000114132.2	zinc finger, DBF-type containing 2
1457	Bach1	ENSMUST00000026703.5	BTB and CNC homology 1
1458	Cdh11	ENSMUST00000075190.3	cadherin 11
1459	Htr5b	ENSMUST00000055884.7	5-hydroxytryptamine (serotonin) receptor 5B
1460	Ssx2ip	ENSMUST00000106153.3	synovial sarcoma, X breakpoint 2 interacting protein
1461	Gm7276	ENSMUST00000097520.2	predicted gene 7276
1462	Igf1	ENSMUST00000105300.3	insulin-like growth factor 1
1463	Zfp459	ENSMUST00000056470.8	zinc finger protein 459
1464	Zfp503	ENSMUST00000043409.7	zinc finger protein 503
1465	Ccnd1	ENSMUST00000093962.4	cyclin D1
1466	Hps5	ENSMUST00000107654.2	Hermansky-Pudlak syndrome 5 homolog (human)
1467	Nrk	ENSMUST00000113052.2	Nik related kinase
1468	9330159N05Rik	ENSMUST00000180541.1	RIKEN cDNA 9330159N05 gene
1469	Taf1d	ENSMUST00000164079.2	TATA box binding protein (Tbp)-associated factor, RNA polymerase I, D
1470	Rprd1b	ENSMUST00000109518.2	regulation of nuclear pre-mRNA domain containing 1B
1471	Pkn2	ENSMUST00000043812.9	protein kinase N2
1472	Rreb1	ENSMUST00000128570.2	ras responsive element binding protein 1
1473	Eaf1	ENSMUST00000022446.5	ELL associated factor 1
1474	Micu3	ENSMUST00000068999.8	mitochondrial calcium uptake family, member 3
1475	Egf	ENSMUST00000029653.2	epidermal growth factor
1476	Bod1l	ENSMUST00000050556.7	biorientation of chromosomes in cell division 1-like
1477	Sla	ENSMUST00000164163.2	src-like adaptor
1478	Fcrls	ENSMUST00000090986.5	Fc receptor-like S, scavenger receptor
1479	Zbtb11	ENSMUST00000050248.8	zinc finger and BTB domain containing 11
1480	Tmbim1	ENSMUST00000016309.10	transmembrane BAX inhibitor motif containing 1
1481	Gm14322	ENSMUST00000119838.3	predicted gene 14322
1482	Pcdh11x	ENSMUST00000113358.4	protocadherin 11 X-linked
1483	Caprin1	ENSMUST00000028607.7	cell cycle associated protein 1
1484	Gm14418	ENSMUST00000128657.2	predicted gene 14418
1485	Robo2	ENSMUST00000117200.2	roundabout homolog 2 (Drosophila)
1486	Hnrnpk	ENSMUST00000116403.3	heterogeneous nuclear ribonucleoprotein K
1487	Abi2	ENSMUST00000052332.9	abl-interactor 2
1488	Rps6ka2	ENSMUST00000024575.6	ribosomal protein S6 kinase, polypeptide 2

1489	Sparcl1	ENSMUST00000031249.3	SPARC-like 1
1490	Neto1	ENSMUST00000058829.2	neuropilin (NRP) and tolloid (TLL)-like 1
1491	Fbln7	ENSMUST00000110324.2	fibulin 7
1492	3222401L13Rik	ENSMUST00000097608.2	RIKEN cDNA 3222401L13 gene
1493	A530053G22Rik	ENSMUST00000060147.4	RIKEN cDNA A530053G22 gene
1494	Ankrd13c	ENSMUST00000040787.8	ankyrin repeat domain 13c
1495	Barhl1	ENSMUST00000050776.3	BarH-like 1 (Drosophila)
1496	Kctd12	ENSMUST00000184744.1	potassium channel tetramerisation domain containing 12
1497	Rps6ka5	ENSMUST00000043599.6	ribosomal protein S6 kinase, polypeptide 5
1498	Scaper	ENSMUST00000037408.8	S phase cyclin A-associated protein in the ER
1499	Rbm24	ENSMUST00000037923.3	RNA binding motif protein 24
1500	Tmem87a	ENSMUST00000090046.6	transmembrane protein 87A
1501	Gabrb3	ENSMUST00000085240.5	gamma-aminobutyric acid (GABA) A receptor, subunit beta 3
1502	BC023829	ENSMUST00000101506.4	cDNA sequence BC023829
1503	Lif	ENSMUST00000066283.6	leukemia inhibitory factor
1504	Gm7903	ENSMUST00000113172.2	predicted gene 7903
1505	Nmur2	ENSMUST00000037682.2	neuromedin U receptor 2
1506	1700086D15Rik	ENSMUST00000020855.3	RIKEN cDNA 1700086D15 gene
1507	Wnk3	ENSMUST00000096285.4	WNK lysine deficient protein kinase 3
1508	Ube2g1	ENSMUST00000021148.7	ubiquitin-conjugating enzyme E2G 1
1509	Mcc	ENSMUST00000164666.2	mutated in colorectal cancers
1510	D930048N14Rik	ENSMUST00000117859.2	RIKEN cDNA D930048N14 gene
1511	Evi5	ENSMUST00000112642.2	ecotropic viral integration site 5
1512	Hps3	ENSMUST00000012580.7	Hermansky-Pudlak syndrome 3 homolog (human)
1513	Trove2	ENSMUST00000159879.1	TROVE domain family, member 2
1514	Clcn5	ENSMUST00000004428.8	chloride channel 5
1515	Anapc1	ENSMUST00000014499.4	anaphase promoting complex subunit 1
1516	Cyyr1	ENSMUST00000114174.2	cysteine and tyrosine-rich protein 1
1517	Lypd1	ENSMUST00000159417.1	Ly6/Plaur domain containing 1
1518	Tacc1	ENSMUST00000084512.5	transforming, acidic coiled-coil containing protein 1
1519	Copg2	ENSMUST00000048774.7	coatomer protein complex, subunit gamma 2
1520	Foxa1	ENSMUST00000044380.6	forkhead box A1
1521	Slc9a5	ENSMUST00000073149.5	solute carrier family 9 (sodium/hydrogen exchanger), member 5
1522	Kbtbd13	ENSMUST00000068307.3	kelch repeat and BTB (POZ) domain containing 13
1523	Ptprr	ENSMUST00000063470.5	protein tyrosine phosphatase, receptor type, R
1524	Nin	ENSMUST00000085314.4	ninein
1525	Hapln1	ENSMUST00000022108.7	hyaluronan and proteoglycan link protein 1
1526	Man1a2	ENSMUST00000008907.8	mannosidase, alpha, class 1A, member 2
1527	Slc16a14	ENSMUST00000027422.6	solute carrier family 16 (monocarboxylic acid transporters), member 14
1528	Myrf	ENSMUST00000088013.6	myelin regulatory factor
1529	Zfp953	ENSMUST00000081582.6	zinc finger protein 953
1530	Prokr2	ENSMUST00000049997.8	prokineticin receptor 2
1531	Synj2	ENSMUST00000115789.2	synaptojanin 2
1532	Ntrk3	ENSMUST00000039431.8	neurotrophic tyrosine kinase, receptor, type 3
1533	Tusc1	ENSMUST00000066774.5	tumor suppressor candidate 1
1534	Gpr161	ENSMUST00000178700.2	G protein-coupled receptor 161
1535	Fbxl14	ENSMUST00000032094.6	F-box and leucine-rich repeat protein 14
1536	Slc25a41	ENSMUST00000058661.8	solute carrier family 25, member 41

1537	Zbtb26	ENSMUST00000067043.4	zinc finger and BTB domain containing 26
1538	BC051628	ENSMUST00000050026.6	cDNA sequence BC051628
1539	Pax7	ENSMUST00000030508.8	paired box 7
1540	Crkl	ENSMUST00000006293.3	v-crk sarcoma virus CT10 oncogene homolog (avian)-like
1541	Dnaja4	ENSMUST00000070070.7	DnaJ (Hsp40) homolog, subfamily A, member 4
1542	Ddi2	ENSMUST00000102484.4	DNA-damage inducible protein 2
1543	Tmem65	ENSMUST00000072113.5	transmembrane protein 65
1544	Blzf1	ENSMUST00000027866.5	basic leucine zipper nuclear factor 1
1545	Fancc	ENSMUST00000073029.6	Fanconi anemia, complementation group C
1546	Vmn1r180	ENSMUST00000173816.3	vomeronasal 1 receptor 180
1547	Cybrd1	ENSMUST00000028403.2	cytochrome b reductase 1
1548	Osbpl2	ENSMUST00000040668.8	oxysterol binding protein-like 2
1549	Crispld1	ENSMUST00000095075.4	cysteine-rich secretory protein LCCL domain containing 1
1550	Nfkbie	ENSMUST00000024742.7	nuclear factor of kappa light polypeptide gene enhancer in B cells inhibitor, epsilon
1551	Ing2	ENSMUST00000080353.2	inhibitor of growth family, member 2
1552	Npas2	ENSMUST00000056815.7	neuronal PAS domain protein 2
1553	Txnrd1	ENSMUST00000020484.6	thioredoxin reductase 1
1554	Tgfbr1	ENSMUST00000007757.9	transforming growth factor, beta receptor I
1555	Trim36	ENSMUST00000167364.2	tripartite motif-containing 36
1556	Cd28	ENSMUST00000027165.2	CD28 antigen
1557	Krr1	ENSMUST00000163048.2	KRR1, small subunit (SSU) processome component, homolog (yeast)
1558	Plcxd3	ENSMUST00000061925.4	phosphatidylinositol-specific phospholipase C, X domain containing 3
1559	Trdmt1	ENSMUST00000124488.1	tRNA aspartic acid methyltransferase 1
1560	Pvrl3	ENSMUST00000023334.9	poliovirus receptor-related 3
1561	Casp16	ENSMUST00000088673.4	caspase 16, apoptosis-related cysteine peptidase
1562	Cxadr	ENSMUST00000023572.8	coxsackie virus and adenovirus receptor
1563	St8sia3	ENSMUST00000025477.8	ST8 alpha-N-acetyl-neuraminide alpha-2,8-sialyltransferase 3
1564	2410127L17Rik	ENSMUST00000025632.9	RIKEN cDNA 2410127L17 gene
1565	BC106179	ENSMUST00000055369.4	cDNA sequence BC106179
1566	Adad1	ENSMUST00000144629.2	adenosine deaminase domain containing 1 (testis specific)
1567	Sp2	ENSMUST00000107626.2	Sp2 transcription factor
1568	Zfp518a	ENSMUST00000050092.6	zinc finger protein 518A
1569	Sh2b3	ENSMUST00000086310.2	SH2B adaptor protein 3
1570	Prf1	ENSMUST00000035419.5	perforin 1 (pore forming protein)
1571	Ar	ENSMUST00000052837.7	androgen receptor
1572	Sec61g	ENSMUST00000109643.2	SEC61, gamma subunit
1573	Dhdds	ENSMUST00000144668.2	dehydrodolichyl diphosphate synthase
1574	Ttc26	ENSMUST00000162554.2	tetratricopeptide repeat domain 26
1575	Rbms3	ENSMUST00000111773.4	RNA binding motif, single stranded interacting protein
1576	E330009J07Rik	ENSMUST00000101492.4	RIKEN cDNA E330009J07 gene
1577	Aph1a	ENSMUST00000015894.6	anterior pharynx defective 1a homolog (C. elegans)
1578	Eif5	ENSMUST00000166123.2	eukaryotic translation initiation factor 5
1579	Lrrc18	ENSMUST00000038956.5	leucine rich repeat containing 18
1580	Tbx18	ENSMUST00000034991.7	T-box18

1581	Gm6768	ENSMUST00000022467.9	predicted gene 6768
1582	Rnf114	ENSMUST00000078050.6	ring finger protein 114
1583	Rest	ENSMUST00000080359.6	RE1-silencing transcription factor
1584	Zfp229	ENSMUST00000182603.1	zinc finger protein 229
1585	Slc26a2	ENSMUST00000146409.2	solute carrier family 26 (sulfate transporter), member 2
1586	Pcdh17	ENSMUST00000071370.5	protocadherin 17
1587	Scarb2	ENSMUST00000031377.7	scavenger receptor class B, member 2
1588	E230025N22Rik	ENSMUST00000115682.1	Riken cDNA E230025N22 gene
1589	Zbtb20	ENSMUST00000114694.3	zinc finger and BTB domain containing 20
1590	Zbtb7a	ENSMUST00000048128.9	zinc finger and BTB domain containing 7a
1591	Hrnr	ENSMUST00000090856.4	hornerin
1592	4933430I17Rik	ENSMUST00000062145.1	RIKEN cDNA 4933430I17 gene
1593	Brca1	ENSMUST00000017290.5	breast cancer 1
1594	Fundc2	ENSMUST00000033541.4	FUN14 domain containing 2
1595	Usp9x	ENSMUST00000089302.5	ubiquitin specific peptidase 9, X chromosome
1596	Gm9887	ENSMUST00000053451.1	predicted gene 9887
1597	Arhgap18	ENSMUST00000039557.7	Rho GTPase activating protein 18
1598	Pms2	ENSMUST00000148011.2	postmeiotic segregation increased 2 (S. cerevisiae)
1599	Galnt11	ENSMUST00000045737.8	UDP-N-acetyl-alpha-D-galactosamine:polypeptide N-acetylgalactosaminyltransferase 11
1600	Hus1	ENSMUST00000020683.4	Hus1 homolog (S. pombe)
1601	Cacng2	ENSMUST00000019290.2	calcium channel, voltage-dependent, gamma subunit 2
1602	Ccdc132	ENSMUST00000164052.3	coiled-coil domain containing 132
1603	Cdh7	ENSMUST00000146282.2	cadherin 7, type 2
1604	Kdm4b	ENSMUST00000025036.5	lysine (K)-specific demethylase 4B
1605	Wdr89	ENSMUST00000062370.8	WD repeat domain 89
1606	Cacna1e	ENSMUST00000187541.1	calcium channel, voltage-dependent, R type, alpha 1E subunit
1607	Mcrs1	ENSMUST00000041190.10	microspherule protein 1
1608	Ppp3cb	ENSMUST00000159027.2	protein phosphatase 3, catalytic subunit, beta isoform
1609	Enpp6	ENSMUST00000039840.8	ectonucleotide pyrophosphatase/phosphodiesterase 6
1610	Ankra2	ENSMUST00000123924.2	ankyrin repeat, family A (RFXANK-like), 2
1611	Tmem140	ENSMUST00000074949.3	transmembrane protein 140
1612	Ywhae	ENSMUST00000067664.4	tyrosine 3-monooxygenase/tryptophan 5-monooxygenase activation protein, epsilon polypeptide
1613	Xpo1	ENSMUST00000102869.2	exportin 1, CRM1 homolog (yeast)
1614	Cldn1	ENSMUST00000023154.2	claudin 1
1615	Psma4	ENSMUST00000034848.8	proteasome (prosome, macropain) subunit, alpha type 4
1616	Socs6	ENSMUST00000070116.6	suppressor of cytokine signaling 6
1617	Gm9949	ENSMUST00000067743.1	predicted gene 9949
1618	Slc1a3	ENSMUST00000157065.1	solute carrier family 1 (glial high affinity glutamate transporter), member 3
1619	Cyp4a14	ENSMUST00000030487.2	cytochrome P450, family 4, subfamily a, polypeptide 14
1620	Olfr519	ENSMUST00000084752.1	olfactory receptor 519
1621	Mfsd6	ENSMUST00000156876.2	major facilitator superfamily domain containing 6
1622	Kcnip4	ENSMUST00000087395.5	Kv channel interacting protein 4
1623	Fcrl1	ENSMUST00000163661.2	Fc receptor-like 1

1624	Rsbn1	ENSMUST00000106806.1	rosbin, round spermatid basic protein 1
1625	Wtap	ENSMUST00000159986.2	Wilms tumour 1-associating protein
1626	Cd33	ENSMUST00000039861.5	CD33 antigen
1627	Cntnap2	ENSMUST00000114641.2	contactin associated protein-like 2
1628	Camta1	ENSMUST00000097774.3	calmodulin binding transcription activator 1
1629	Apaf1	ENSMUST00000159110.2	apoptotic peptidase activating factor 1
1630	Pcdhb18	ENSMUST00000055949.2	protocadherin beta 18
1631	Cep41	ENSMUST00000031810.9	centrosomal protein 41
1632	Plekhh2	ENSMUST00000047206.5	pleckstrin homology domain containing, family H (with MyTH4 domain) member 2
1633	Cd2ap	ENSMUST00000024709.7	CD2-associated protein
1634	Ano5	ENSMUST00000043944.5	anoctamin 5
1635	Vti1a	ENSMUST00000095950.2	vesicle transport through interaction with t-SNAREs 1A
1636	Atf7	ENSMUST00000184616.2	activating transcription factor 7
1637	Rab2b	ENSMUST00000022765.8	RAB2B, member RAS oncogene family
1638	Wdr20	ENSMUST00000095410.2	WD repeat domain 20
1639	5430427O19Rik	ENSMUST00000113976.1	RIKEN cDNA 5430427O19 gene
1640	Mrap2	ENSMUST00000113149.2	melanocortin 2 receptor accessory protein 2
1641	Nipal2	ENSMUST00000040791.7	NIPA-like domain containing 2
1642	AF529169	ENSMUST00000044491.7	cDNA sequence AF529169
1643	E030002O03Rik	ENSMUST00000051137.9	RIKEN cDNA E030002O03 gene
1644	Bnc1	ENSMUST00000026096.7	basonuclin 1
1645	Dcaf6	ENSMUST00000027856.7	DDB1 and CUL4 associated factor 6
1646	Taf9	ENSMUST00000190594.1	TAF9 RNA polymerase II, TATA box binding protein (TBP)-associated factor
1647	Etaa1	ENSMUST00000076661.6	Ewing tumor-associated antigen 1
1648	Sfmbt1	ENSMUST00000054230.5	Scm-like with four mbt domains 1
1649	Thoc3	ENSMUST00000026990.5	THO complex 3
1650	Vps13c	ENSMUST00000077879.5	vacuolar protein sorting 13C (yeast)
1651	Zfp386	ENSMUST00000183125.1	zinc finger protein 386 (Kruppel-like)
1652	Spice1	ENSMUST00000050897.6	spindle and centriole associated protein 1
1653	Dyrk2	ENSMUST00000004281.8	dual-specificity tyrosine-(Y)-phosphorylation regulated kinase 2
1654	C030039L03Rik	ENSMUST00000120004.2	RIKEN cDNA C030039L03 gene
1655	Gltpd2	ENSMUST00000057685.2	glycolipid transfer protein domain containing 2
1656	Jup	ENSMUST00000001592.9	junction plakoglobin
1657	Cul5	ENSMUST00000120122.2	cullin 5
1658	Panx3	ENSMUST00000011262.2	pannexin 3
1659	Rex2	ENSMUST00000075775.5	reduced expression 2
1660	Rab3d	ENSMUST00000115351.4	RAB3D, member RAS oncogene family
1661	Hipk2	ENSMUST00000161779.2	homeodomain interacting protein kinase 2
1662	Taok1	ENSMUST00000017435.5	TAO kinase 1
1663	Pbx1	ENSMUST00000176790.2	pre B cell leukemia homeobox 1
1664	Slc16a2	ENSMUST00000042664.6	solute carrier family 16 (monocarboxylic acid transporters), member 2
1665	Wac	ENSMUST00000167020.2	WW domain containing adaptor with coiled-coil
1666	Gpr50	ENSMUST00000070449.5	G-protein-coupled receptor 50
1667	Hist1h4b	ENSMUST00000102965.3	histone cluster 1, H4b
1668	Mpz	ENSMUST00000070758.4	myelin protein zero
1669	Map9	ENSMUST00000091014.4	microtubule-associated protein 9
1670	Rgs21	ENSMUST00000184189.2	regulator of G-protein signalling 21

1671	Rnf150	ENSMUST00000078525.5	ring finger protein 150
1672	Dlg3	ENSMUST00000113736.3	discs, large homolog 3 (Drosophila)
1673	4933426M11Rik	ENSMUST00000068519.5	RIKEN cDNA 4933426M11 gene
1674	Sgpl1	ENSMUST00000092498.6	sphingosine phosphate lyase 1
1675	Tanc2	ENSMUST00000100330.4	tetratricopeptide repeat, ankyrin repeat and coiled-coil containing 2
1676	Epc2	ENSMUST00000092123.5	enhancer of polycomb homolog 2 (Drosophila)
1677	Ube2f	ENSMUST00000171165.2	ubiquitin-conjugating enzyme E2F (putative)
1678	4930524B15Rik	ENSMUST00000102829.3	RIKEN cDNA 4930524B15 gene
1679	Slk	ENSMUST00000026043.6	STE20-like kinase
1680	Scai	ENSMUST00000038874.6	suppressor of cancer cell invasion
1681	Cnbp	ENSMUST00000032138.9	cellular nucleic acid binding protein
1682	Hgf	ENSMUST00000030683.3	hepatocyte growth factor
1683	Prr23a	ENSMUST00000170349.2	proline rich 23A
1684	Fgf11	ENSMUST00000102585.1	fibroblast growth factor 11
1685	Ncoa4	ENSMUST00000163336.2	nuclear receptor coactivator 4
1686	Sept11	ENSMUST00000074733.7	septin 11
1687	Smad7	ENSMUST00000026999.4	SMAD family member 7
1688	Spata13	ENSMUST00000022566.8	spermatogenesis associated 13
1689	F8	ENSMUST00000033539.7	coagulation factor VIII
1690	Asb10	ENSMUST00000048302.7	ankyrin repeat and SOCS box-containing 10
1691	Fgf12	ENSMUST00000100024.1	fibroblast growth factor 12
1692	Tnik	ENSMUST00000159236.3	TRAF2 and NCK interacting kinase
1693	Itgam	ENSMUST00000106242.4	integrin alpha M
1694	Rnf180	ENSMUST00000069686.6	ring finger protein 180
1695	Rrs1	ENSMUST00000072079.8	RRS1 ribosome biogenesis regulator homolog (S. cerevisiae)
1696	Mfap3l	ENSMUST00000160719.2	microfibrillar-associated protein 3-like
1697	Zfp600	ENSMUST00000092750.6	zinc finger protein 600
1698	Mpzl3	ENSMUST00000114664.2	myelin protein zero-like 3
1699	Tspyl1	ENSMUST00000061372.6	testis-specific protein, Y-encoded-like 1
1700	Creb3l1	ENSMUST00000028663.4	cAMP responsive element binding protein 3-like 1
1701	Ric3	ENSMUST00000055993.7	resistance to inhibitors of cholinesterase 3 homolog (C. elegans)
1702	Cnot7	ENSMUST00000034012.4	CCR4-NOT transcription complex, subunit 7
1703	Col24a1	ENSMUST00000029848.4	collagen, type XXIV, alpha 1
1704	Ccdc25	ENSMUST00000022614.5	coiled-coil domain containing 25
1705	Stx6	ENSMUST00000027743.7	syntaxin 6
1706	Kbtbd2	ENSMUST00000114323.2	kelch repeat and BTB (POZ) domain containing 2
1707	Rcor1	ENSMUST00000084968.8	REST corepressor 1
1708	Fam184b	ENSMUST00000016023.7	family with sequence similarity 184, member B
1709	Lrit1	ENSMUST00000120052.1	leucine-rich repeat, immunoglobulin-like and transmembrane domains 1
1710	Gm8975	ENSMUST00000156818.1	predicted gene 8975
1711	Map3k2	ENSMUST00000096575.3	mitogen-activated protein kinase kinase kinase 2
1712	Arl5b	ENSMUST00000069870.4	ADP-ribosylation factor-like 5B
1713	Snupn	ENSMUST00000068856.4	snurportin 1
1714	Cnot6	ENSMUST00000145353.2	CCR4-NOT transcription complex, subunit 6
1715	Fem1c	ENSMUST00000036226.5	fem-1 homolog c (C.elegans)
1716	Chrna4	ENSMUST00000067120.8	cholinergic receptor, nicotinic, alpha polypeptide 4
1717	Epb4.2	ENSMUST00000102490.4	erythrocyte protein band 4.2
1718	Kat2b	ENSMUST00000000724.9	K(lysine) acetyltransferase 2B

1719	Relt	ENSMUST00000008462.4	RELT tumor necrosis factor receptor
1720	4931440P22Rik	ENSMUST00000099076.3	RIKEN cDNA 4931440P22 gene
1721	Fam46c	ENSMUST00000061455.8	family with sequence similarity 46, member C
1722	Hs3st3b1	ENSMUST00000094103.3	heparan sulfate (glucosamine) 3-O-sulfotransferase 3B1
1723	Guca1b	ENSMUST00000024774.8	guanylate cyclase activator 1B
1724	Trabd2b	ENSMUST00000094894.3	TraB domain containing 2B
1725	Gpr21	ENSMUST00000065441.6	G protein-coupled receptor 21
1726	Sec63	ENSMUST00000019937.4	SEC63-like (S. cerevisiae)
1727	Fam210a	ENSMUST00000042852.6	family with sequence similarity 210, member A
1728	Ubp1	ENSMUST00000084885.6	upstream binding protein 1
1729	Pcnx	ENSMUST00000021567.5	pecanex homolog (Drosophila)
1730	Zmiz1	ENSMUST00000162645.2	zinc finger, MIZ-type containing 1
1731	Cdc27	ENSMUST00000093923.3	cell division cycle 27
1732	Dpy19l4	ENSMUST00000084892.6	dpy-19-like 4 (C. elegans)
1733	Ikbkg	ENSMUST00000114133.3	inhibitor of kappaB kinase gamma
1734	Mbd5	ENSMUST00000112745.2	methyl-CpG binding domain protein 5
1735	Akap2	ENSMUST00000102903.2	A kinase (PRKA) anchor protein 2
1736	Foxp2	ENSMUST00000115477.2	forkhead box P2
1737	Eif2s3x	ENSMUST00000050328.9	eukaryotic translation initiation factor 2, subunit 3, structural gene X-linked
1738	S100b	ENSMUST00000036387.7	S100 protein, beta polypeptide, neural
1739	Ankrd13a	ENSMUST00000102578.5	ankyrin repeat domain 13a
1740	Ntng1	ENSMUST00000156177.3	netrin G1
1741	Kcna6	ENSMUST00000185333.1	potassium voltage-gated channel, shaker-related, subfamily, member 6
1742	Tfe3	ENSMUST00000077680.4	transcription factor E3
1743	A830018L16Rik	ENSMUST00000135014.2	RIKEN cDNA A830018L16 gene
1744	Nhs	ENSMUST00000087085.4	Nance-Horan syndrome (human)
1745	March2	ENSMUST00000173015.2	membrane-associated ring finger (C3HC4) 2
1746	Gabpa	ENSMUST00000009120.7	GA repeat binding protein, alpha
1747	Ism1	ENSMUST00000099307.3	isthmin 1 homolog (zebrafish)
1748	Clock	ENSMUST00000075159.1	circadian locomotor output cycles kaput
1749	Rpl15	ENSMUST00000080281.8	ribosomal protein L15
1750	Wee2	ENSMUST00000038907.8	WEE1 homolog 2 (S. pombe)
1751	Esr1	ENSMUST00000105590.2	estrogen receptor 1 (alpha)
1752	Gdf7	ENSMUST00000037313.4	growth differentiation factor 7
1753	Steap2	ENSMUST00000164219.2	six transmembrane epithelial antigen of prostate 2
1754	Glp2r	ENSMUST00000051765.8	glucagon-like peptide 2 receptor
1755	Wipf3	ENSMUST00000163746.2	WAS/WASL interacting protein family, member 3
1756	Asxl2	ENSMUST00000111215.4	additional sex combs like 2 (Drosophila)
1757	Flrt3	ENSMUST00000110057.2	fibronectin leucine rich transmembrane protein 3
1758	Ubn2	ENSMUST00000160583.2	ubinuclein 2
1759	Socs4	ENSMUST00000065562.4	suppressor of cytokine signaling 4
1760	Pof1b	ENSMUST00000039887.3	premature ovarian failure 1B
1761	Wdtc1	ENSMUST00000105906.1	WD and tetratricopeptide repeats 1
1762	Ap5m1	ENSMUST00000037473.4	adaptor-related protein complex 5, mu 1 subunit
1763	Gucy1a2	ENSMUST00000115733.1	guanylate cyclase 1, soluble, alpha 2
1764	Dnajc28	ENSMUST00000169982.1	DnaJ (Hsp40) homolog, subfamily C, member 28
1765	Srd5a3	ENSMUST00000031143.7	steroid 5 alpha-reductase 3
1766	Chpf	ENSMUST00000079205.8	chondroitin polymerizing factor
1767	Pkhd1	ENSMUST00000088448.6	polycystic kidney and hepatic disease 1

1768	Pde4b	ENSMUST00000106908.3	phosphodiesterase 4B, cAMP specific
1769	Mpp7	ENSMUST00000115869.2	membrane protein, palmitoylated 7 (MAGUK p55 subfamily member 7)
1770	Celf1	ENSMUST00000068726.7	CUGBP, Elav-like family member 1
1771	Hcn1	ENSMUST00000006991.7	hyperpolarization-activated, cyclic nucleotide-gated K+ 1
1772	Gpr1	ENSMUST00000050536.8	G protein-coupled receptor 1
1773	Fam114a1	ENSMUST00000031080.9	family with sequence similarity 114, member A1
1774	H1f0	ENSMUST00000180086.1	H1 histone family, member 0
1775	Il1f5	ENSMUST00000028360.2	interleukin 1 family, member 5 (delta)
1776	Keap1	ENSMUST00000164812.2	kelch-like ECH-associated protein 1
1777	2310035C23Rik	ENSMUST00000086721.4	RIKEN cDNA 2310035C23 gene
1778	E2f3	ENSMUST00000102948.4	E2F transcription factor 3
1779	Mark1	ENSMUST00000027929.4	MAP/microtubule affinity-regulating kinase 1
1780	Golim4	ENSMUST00000117242.2	golgi integral membrane protein 4
1781	Cd300e	ENSMUST00000062787.7	CD300e antigen
1782	St8sia1	ENSMUST00000032421.3	ST8 alpha-N-acetyl-neuraminide alpha-2,8-sialyltransferase 1
1783	Ebf2	ENSMUST00000176161.2	early B cell factor 2
1784	Dhrs9	ENSMUST00000063690.3	dehydrogenase/reductase (SDR family) member 9
1785	Scd4	ENSMUST00000058856.8	stearoyl-coenzyme A desaturase 4
1786	Zfp3	ENSMUST00000060444.5	zinc finger protein 3
1787	B3galt2	ENSMUST00000038252.3	UDP-Gal:betaGlcNAc beta 1,3-galactosyltransferase, polypeptide 2
1788	Pou2f1	ENSMUST00000111429.5	POU domain, class 2, transcription factor 1
1789	Lacc1	ENSMUST00000062789.9	laccase (multicopper oxidoreductase) domain containing 1
1790	Pipox	ENSMUST00000017597.4	pipecolic acid oxidase
1791	Kcnj16	ENSMUST00000106636.2	potassium inwardly-rectifying channel, subfamily J, member 16
1792	Negr1	ENSMUST00000074015.5	neuronal growth regulator 1
1793	Arhgef38	ENSMUST00000147041.4	Rho guanine nucleotide exchange factor (GEF) 38
1794	Macrod2	ENSMUST00000110064.2	MACRO domain containing 2
1795	Acsl4	ENSMUST00000112907.2	acyl-CoA synthetase long-chain family member 4
1796	Birc3	ENSMUST00000013949.9	baculoviral IAP repeat-containing 3
1797	Tmpo	ENSMUST00000072239.8	thymopoietin
1798	Hsp90aa1	ENSMUST00000094361.5	heat shock protein 90, alpha (cytosolic), class A member 1
1799	Enah	ENSMUST00000078719.7	enabled homolog (Drosophila)
1800	Tmem186	ENSMUST00000052505.8	transmembrane protein 186
1801	Ppp2r3c	ENSMUST00000021410.8	protein phosphatase 2, regulatory subunit B", gamma
1802	Adam7	ENSMUST00000022640.7	a disintegrin and metallopeptidase domain 7
1803	Coasy	ENSMUST00000001806.4	Coenzyme A synthase
1804	Pik3r3	ENSMUST00000030464.8	phosphatidylinositol 3 kinase, regulatory subunit, polypeptide 3 (p55)
1805	Fam168b	ENSMUST00000167518.2	family with sequence similarity 168, member B
1806	Rassf9	ENSMUST00000055355.4	Ras association (RalGDS/AF-6) domain family (N-terminal) member 9
1807	Zfp326	ENSMUST00000031227.5	zinc finger protein 326
1808	D130040H23Rik	ENSMUST00000078257.6	RIKEN cDNA D130040H23 gene
1809	Chst11	ENSMUST00000040110.7	carbohydrate sulfotransferase 11
1810	Mmp16	ENSMUST00000029881.4	matrix metallopeptidase 16

1811	Slc30a10	ENSMUST00000061093.6	solute carrier family 30, member 10
1812	Gm3629	ENSMUST00000171706.1	predicted gene 3629
1813	Kcna4	ENSMUST00000037012.2	potassium voltage-gated channel, shaker-related subfamily, member 4
1814	Rab39b	ENSMUST00000033545.5	RAB39B, member RAS oncogene family
1815	Tenm3	ENSMUST00000110346.2	teneurin transmembrane protein 3
1816	Prdm14	ENSMUST00000047577.6	PR domain containing 14
1817	Hspa4l	ENSMUST00000077083.7	heat shock protein 4 like
1818	Phex	ENSMUST00000079945.5	phosphate regulating gene with homologies to endopeptidases on the X chromosome (hypophosphatemia, vitamin D resistant rickets)
1819	Armcx6	ENSMUST00000113194.2	armadillo repeat containing, X-linked 6
1820	Pxdn	ENSMUST00000122328.2	peroxidasin homolog (Drosophila)
1821	Rnf24	ENSMUST00000059372.5	ring finger protein 24
1822	1700052N19Rik	ENSMUST00000118544.2	RIKEN cDNA 1700052N19 gene
1823	Vopp1	ENSMUST00000114297.2	vesicular, overexpressed in cancer, prosurvival protein 1
1824	Eri1	ENSMUST00000033927.6	exoribonuclease 1
1825	Nudcd1	ENSMUST00000038719.6	NudC domain containing 1
1826	Bicc1	ENSMUST00000143791.2	bicaudal C homolog 1 (Drosophila)
1827	Wrn	ENSMUST00000033991.7	Werner syndrome homolog (human)
1828	Itga8	ENSMUST00000028106.5	integrin alpha 8
1829	Tox3	ENSMUST00000109621.4	TOX high mobility group box family member 3
1830	Gsx1	ENSMUST00000065382.5	GS homeobox 1
1831	Gpr158	ENSMUST00000055946.7	G protein-coupled receptor 158
1832	Styx	ENSMUST00000111835.2	serine/threonine/tyrosine interaction protein
1833	Slc2a13	ENSMUST00000109283.1	solute carrier family 2 (facilitated glucose transporter), member 13
1834	Hyal1	ENSMUST00000010195.8	hyaluronoglucosaminidase 1
1835	Akirin1	ENSMUST00000102636.3	akirin 1
1836	Slc16a6	ENSMUST00000070152.6	solute carrier family 16 (monocarboxylic acid transporters), member 6
1837	Stk24	ENSMUST00000079817.7	serine/threonine kinase 24
1838	Bend4	ENSMUST00000169190.1	BEN domain containing 4
1839	Nxnl1	ENSMUST00000163659.1	nucleoredoxin-like 1
1840	Baz2b	ENSMUST00000112550.2	bromodomain adjacent to zinc finger domain, 2B
1841	Necab1	ENSMUST00000041606.8	N-terminal EF-hand calcium binding protein 1
1842	Dgke	ENSMUST00000107894.2	diacylglycerol kinase, epsilon
1843	Ccin	ENSMUST00000095107.2	calicin
1844	Pcdhb10	ENSMUST00000051126.2	protocadherin beta 10
1845	D16Ertd472e	ENSMUST00000114220.2	DNA segment, Chr 16, ERATO Doi 472, expressed
1846	Carhsp1	ENSMUST00000008537.8	calcium regulated heat stable protein 1
1847	St6gal2	ENSMUST00000025000.3	beta galactoside alpha 2,6 sialyltransferase 2
1848	Nhsl2	ENSMUST00000101339.5	NHS-like 2
1849	Yy2	ENSMUST00000065806.4	Yy2 transcription factor
1850	Pdk1	ENSMUST00000006669.5	pyruvate dehydrogenase kinase, isoenzyme 1
1851	Ccser1	ENSMUST00000126214.2	coiled-coil serine rich 1
1852	Tusc2	ENSMUST00000010198.3	tumor suppressor candidate 2
1853	Bcl2	ENSMUST00000112751.1	B cell leukemia/lymphoma 2
1854	Srsf6	ENSMUST00000130411.1	serine/arginine-rich splicing factor 6
1855	Slc1a2	ENSMUST00000080210.4	solute carrier family 1 (glial high affinity glutamate transporter), member 2

1856	Cyhr1	ENSMUST00000081291.7	cysteine and histidine rich 1
1857	Riok3	ENSMUST00000025270.6	RIO kinase 3
1858	Ywhab	ENSMUST00000018470.4	tyrosine 3-monooxygenase/tryptophan 5-monooxygenase activation protein, beta polypeptide
1859	Zcchc18	ENSMUST00000033804.4	zinc finger, CCHC domain containing 18
1860	Ppp1r12a	ENSMUST00000070663.5	protein phosphatase 1, regulatory (inhibitor) subunit 12A
1861	Hic2	ENSMUST00000090190.6	hypermethylated in cancer 2
1862	Tmem74	ENSMUST00000067469.4	transmembrane protein 74
1863	Nhlrc3	ENSMUST00000056749.8	NHL repeat containing 3
1864	Ap3m2	ENSMUST00000163739.1	adaptor-related protein complex 3, mu 2 subunit
1865	Scn7a	ENSMUST00000042792.6	sodium channel, voltage-gated, type VII, alpha
1866	Mkl2	ENSMUST00000149359.1	MKL/myocardin-like 2
1867	Gmps	ENSMUST00000029405.7	guanine monophosphate synthetase
1868	Zfp40	ENSMUST00000037057.8	zinc finger protein 40
1869	Fam122a	ENSMUST00000099556.1	family with sequence similarity 122, member A
1870	Ttbk1	ENSMUST00000047034.8	tau tubulin kinase 1
1871	Galnt10	ENSMUST00000066987.8	UDP-N-acetyl-alpha-D-galactosamine:polypeptide N-acetylgalactosaminyltransferase 10
1872	Fermt1	ENSMUST00000038280.4	fermitin family homolog 1 (Drosophila)
1873	Zfp518b	ENSMUST00000057258.5	zinc finger protein 518B
1874	Lrig2	ENSMUST00000046316.6	leucine-rich repeats and immunoglobulin-like domains 2
1875	Phc3	ENSMUST00000064718.6	polyhomeotic-like 3 (Drosophila)
1876	Cntn1	ENSMUST00000169825.2	contactin 1
1877	Usp14	ENSMUST00000092096.7	ubiquitin specific peptidase 14
1878	Cnksr2	ENSMUST00000026750.9	connector enhancer of kinase suppressor of Ras 2
1879	Apol10b	ENSMUST00000089465.4	apolipoprotein L 10B
1880	Zfp770	ENSMUST00000050668.3	zinc finger protein 770
1881	Traf6	ENSMUST00000004949.7	TNF receptor-associated factor 6
1882	Syt13	ENSMUST00000028648.2	synaptotagmin XIII
1883	Cdca3	ENSMUST00000024270.8	cell division cycle associated 3
1884	Tnip2	ENSMUST00000030991.8	TNFAIP3 interacting protein 2
1885	Gm6710	ENSMUST00000109012.3	predicted gene 6710
1886	Hist2h4	ENSMUST00000171473.2	histone cluster 2, H4
1887	Fbxo42	ENSMUST00000030757.9	F-box protein 42
1888	Pcif1	ENSMUST00000041643.3	PDX1 C-terminal inhibiting factor 1
1889	Slc12a2	ENSMUST00000115366.2	solute carrier family 12, member 2
1890	Plekhf2	ENSMUST00000054776.3	pleckstrin homology domain containing, family F (with FYVE domain) member 2
1891	Nos3	ENSMUST00000030834.5	nitric oxide synthase 3, endothelial cell
1892	Rnf19a	ENSMUST00000022890.8	ring finger protein 19A
1893	Oprm1	ENSMUST00000152674.1	opioid receptor, mu 1
1894	Gpatch2l	ENSMUST00000071106.4	G patch domain containing 2 like
1895	Bmp3	ENSMUST00000031278.4	bone morphogenetic protein 3
1896	Abi1	ENSMUST00000140164.2	abl-interactor 1
1897	Fnbp1l	ENSMUST00000162409.2	formin binding protein 1-like
1898	Magi1	ENSMUST00000089317.6	membrane associated guanylate kinase, WW and PDZ domain containing 1
1899	Slc44a1	ENSMUST00000107651.3	solute carrier family 44, member 1
1900	Prkar1a	ENSMUST00000106677.2	protein kinase, cAMP dependent regulatory, type I, alpha

1901	Bmp2k	ENSMUST00000035635.8	BMP2 inducible kinase
1902	Kcnj11	ENSMUST00000180081.1	potassium inwardly rectifying channel, subfamily J, member 11
1903	Myt1l	ENSMUST00000049784.10	myelin transcription factor 1-like
1904	Pcgf3	ENSMUST00000046975.6	polycomb group ring finger 3
1905	Tcf23	ENSMUST00000006818.2	transcription factor 23
1906	Specc1l	ENSMUST00000105421.3	sperm antigen with calponin homology and coiled-coil domains 1-like
1907	Fam46d	ENSMUST00000101292.3	family with sequence similarity 46, member D
1908	Sdpr	ENSMUST00000051572.7	serum deprivation response
1909	Cdc14b	ENSMUST00000109770.1	CDC14 cell division cycle 14B
1910	Glis3	ENSMUST00000162022.2	GLIS family zinc finger 3
1911	Colec10	ENSMUST00000036737.3	collectin sub-family member 10
1912	Isy1	ENSMUST00000089497.4	ISY1 splicing factor homolog (S. cerevisiae)
1913	Zc3h15	ENSMUST00000081591.6	zinc finger CCCH-type containing 15
1914	C77370	ENSMUST00000118314.2	expressed sequence C77370
1915	B3glct	ENSMUST00000100404.3	beta-3-glucosyltransferase
1916	Lonrf1	ENSMUST00000065297.5	LON peptidase N-terminal domain and ring finger 1
1917	Il4ra	ENSMUST00000033004.6	interleukin 4 receptor, alpha
1918	Endod1	ENSMUST00000167549.1	endonuclease domain containing 1
1919	Xndc1	ENSMUST00000106950.2	Xrcc1 N-terminal domain containing 1
1920	Trpc5	ENSMUST00000040184.3	transient receptor potential cation channel, subfamily C, member 5
1921	Gpr137c	ENSMUST00000146150.1	G protein-coupled receptor 137C
1922	Prss35	ENSMUST00000036426.7	protease, serine 35
1923	Wdr1	ENSMUST00000005234.9	WD repeat domain 1
1924	Dido1	ENSMUST00000103055.2	death inducer-obliterator 1
1925	Ankhd1	ENSMUST00000155329.3	ankyrin repeat and KH domain containing 1
1926	Pitpnc1	ENSMUST00000103064.4	phosphatidylinositol transfer protein, cytoplasmic 1
1927	Pfkfb3	ENSMUST00000028114.7	6-phosphofructo-2-kinase/fructose-2,6-biphosphatase 3
1928	Atp6v1b2	ENSMUST00000006435.7	ATPase, H+ transporting, lysosomal V1 subunit B2
1929	B230217C12Rik	ENSMUST00000054783.4	RIKEN cDNA B230217C12 gene
1930	Ccny	ENSMUST00000053917.4	cyclin Y
1931	Sp4	ENSMUST00000026367.9	trans-acting transcription factor 4
1932	Tmcc1	ENSMUST00000088896.4	transmembrane and coiled coil domains 1
1933	Zfp882	ENSMUST00000110002.2	zinc finger protein 882
1934	Grap2	ENSMUST00000043149.7	GRB2-related adaptor protein 2
1935	Ctnna3	ENSMUST00000105440.2	catenin (cadherin associated protein), alpha 3
1936	Ostc	ENSMUST00000043937.7	oligosaccharyltransferase complex subunit
1937	Tvp23b	ENSMUST00000014321.4	trans-golgi network vesicle protein 23B
1938	Col9a3	ENSMUST00000132527.3	collagen, type IX, alpha 3
1939	3110039M20Rik	ENSMUST00000110746.1	RIKEN cDNA 3110039M20 gene
1940	Kcnc2	ENSMUST00000092175.2	potassium voltage gated channel, Shaw-related subfamily, member 2
1941	Il1rapl1	ENSMUST00000113966.2	interleukin 1 receptor accessory protein-like 1
1942	Dlx4	ENSMUST00000021241.7	distal-less homeobox 4
1943	Pard6b	ENSMUST00000052125.6	par-6 family cell polarity regulator beta
1944	Samd8	ENSMUST00000119430.1	sterile alpha motif domain containing 8
1945	Rab27b	ENSMUST00000121693.2	RAB27B, member RAS oncogene family
1946	Cox15	ENSMUST00000045562.5	cytochrome c oxidase assembly protein 15
1947	Prdm11	ENSMUST00000111274.2	PR domain containing 11

1948	Lrrc8c	ENSMUST00000067924.7	leucine rich repeat containing 8 family, member C
1949	Wwtr1	ENSMUST00000029380.8	WW domain containing transcription regulator 1
1950	Mal2	ENSMUST00000025356.2	mal, T cell differentiation protein 2
1951	Olig2	ENSMUST00000035608.8	oligodendrocyte transcription factor 2
1952	Arhgap21	ENSMUST00000114594.2	Rho GTPase activating protein 21
1953	Mlec	ENSMUST00000112121.2	malectin
1954	Olfr606	ENSMUST00000098200.1	olfactory receptor 606
1955	Gid8	ENSMUST00000029090.3	GID complex subunit 8 homolog (S. cerevisiae)
1956	Lrrc38	ENSMUST00000052458.2	leucine rich repeat containing 38
1957	Ddx3x	ENSMUST00000000804.6	DEAD/H (Asp-Glu-Ala-Asp/His) box polypeptide 3, X-linked
1958	Cog6	ENSMUST00000036665.4	component of oligomeric golgi complex 6
1959	Gorasp1	ENSMUST00000035099.7	golgi reassembly stacking protein 1
1960	Sucla2	ENSMUST00000160507.2	succinate-Coenzyme A ligase, ADP-forming, beta subunit
1961	Dcp2	ENSMUST00000025350.8	DCP2 decapping enzyme homolog (S. cerevisiae)
1962	Zbtb44	ENSMUST00000115222.3	zinc finger and BTB domain containing 44
1963	Ctnnbip1	ENSMUST00000030839.7	catenin beta interacting protein 1
1964	Ssbp2	ENSMUST00000004094.9	single-stranded DNA binding protein 2
1965	Acn9	ENSMUST00000040826.5	ACN9 homolog (S. cerevisiae)
1966	Olfr464	ENSMUST00000074874.3	olfactory receptor 464
1967	Cib2	ENSMUST00000041901.6	calcium and integrin binding family member 2
1968	Unc5c	ENSMUST00000106236.3	unc-5 homolog C (C. elegans)
1969	Ttpa	ENSMUST00000117632.2	tocopherol (alpha) transfer protein
1970	Aldh1l2	ENSMUST00000020497.8	aldehyde dehydrogenase 1 family, member L2
1971	Abca6	ENSMUST00000044003.8	ATP-binding cassette, sub-family A (ABC1), member 6
1972	Ugt2a3	ENSMUST00000031195.2	UDP glucuronosyltransferase 2 family, polypeptide A3
1973	Vav3	ENSMUST00000046864.8	vav 3 oncogene
1974	Cdh6	ENSMUST00000036439.4	cadherin 6
1975	Bicd2	ENSMUST00000048544.7	bicaudal D homolog 2 (Drosophila)
1976	Phactr1	ENSMUST00000148891.2	phosphatase and actin regulator 1
1977	Eny2	ENSMUST00000060652.3	enhancer of yellow 2 homolog (Drosophila)
1978	Ston2	ENSMUST00000052969.8	stonin 2
1979	Zbtb43	ENSMUST00000028125.6	zinc finger and BTB domain containing 43
1980	Prr5l	ENSMUST00000163762.2	proline rich 5 like
1981	Rdh5	ENSMUST00000026406.8	retinol dehydrogenase 5
1982	Serac1	ENSMUST00000097432.4	serine active site containing 1
1983	Ptp4a1	ENSMUST00000027232.8	protein tyrosine phosphatase 4a1
1984	Psd3	ENSMUST00000038959.10	pleckstrin and Sec7 domain containing 3
1985	Zkscan1	ENSMUST00000019660.5	zinc finger with KRAB and SCAN domains 1
1986	Dennd5b	ENSMUST00000111557.2	DENN/MADD domain containing 5B
1987	Rtp2	ENSMUST00000061030.8	receptor transporter protein 2
1988	AU041133	ENSMUST00000105314.2	expressed sequence AU041133
1989	Mboat1	ENSMUST00000047311.9	membrane bound O-acyltransferase domain containing 1
1990	Cxcr5	ENSMUST00000179828.2	chemokine (C-X-C motif) receptor 5
1991	Unc13c	ENSMUST00000184666.2	unc-13 homolog C (C. elegans)
1992	Fam126b	ENSMUST00000161600.2	family with sequence similarity 126, member B
1993	Nvl	ENSMUST00000027797.7	nuclear VCP-like

1994	Slc17a3	ENSMUST00000166467.2	solute carrier family 17 (sodium phosphate), member 3
1995	Arpp19	ENSMUST00000169492.2	cAMP-regulated phosphoprotein 19
1996	Kdsr	ENSMUST00000010049.5	3-ketodihydrosphingosine reductase
1997	Rad18	ENSMUST00000077088.5	RAD18 homolog (S. cerevisiae)
1998	Mbnl3	ENSMUST00000114875.2	muscleblind-like 3 (Drosophila)
1999	Ebag9	ENSMUST00000022964.7	estrogen receptor-binding fragment-associated gene 9
2000	Rnf2	ENSMUST00000187048.1	ring finger protein 2
2001	Coa5	ENSMUST00000027286.6	cytochrome C oxidase assembly factor 5
2002	Fut11	ENSMUST00000048016.1	fucosyltransferase 11
2003	Tnrc6b	ENSMUST00000067689.7	trinucleotide repeat containing 6b
2004	Tm7sf3	ENSMUST00000037709.10	transmembrane 7 superfamily member 3
2005	Kbtbd11	ENSMUST00000069399.6	kelch repeat and BTB (POZ) domain containing 11
2006	Casq2	ENSMUST00000029454.6	calsequestrin 2
2007	Chmp4c	ENSMUST00000029049.5	charged multivesicular body protein 4C
2008	Tacr3	ENSMUST00000029822.4	tachykinin receptor 3
2009	Tlr9	ENSMUST00000062241.10	toll-like receptor 9
2010	Zfp707	ENSMUST00000109966.2	zinc finger protein 707
2011	Tmprss3	ENSMUST00000024833.6	transmembrane protease, serine 3
2012	Slc7a11	ENSMUST00000029297.4	solute carrier family 7 (cationic amino acid transporter, y+ system), member 11
2013	Plekhb2	ENSMUST00000027297.5	pleckstrin homology domain containing, family B (evectins) member 2
2014	Dsn1	ENSMUST00000103129.3	DSN1, MIND kinetochore complex component, homolog (S. cerevisiae)
2015	Dzank1	ENSMUST00000081982.6	double zinc ribbon and ankyrin repeat domains 1
2016	Cdk14	ENSMUST00000115451.2	cyclin-dependent kinase 14
2017	Skap2	ENSMUST00000078214.5	src family associated phosphoprotein 2
2018	Rassf8	ENSMUST00000111704.2	Ras association (RalGDS/AF-6) domain family (N-terminal) member 8
2019	Gabrg3	ENSMUST00000068911.7	gamma-aminobutyric acid (GABA) A receptor, subunit gamma 3
2020	Crebl2	ENSMUST00000046303.6	cAMP responsive element binding protein-like 2
2021	Frk	ENSMUST00000019913.8	fyn-related kinase
2022	Gm6406	ENSMUST00000071302.2	predicted gene 6406
2023	Cdc42bpa	ENSMUST00000111117.2	CDC42 binding protein kinase alpha
2024	Kif27	ENSMUST00000043605.5	kinesin family member 27
2025	Ccdc96	ENSMUST00000060100.2	coiled-coil domain containing 96
2026	Tnfrsf19	ENSMUST00000111234.3	tumor necrosis factor receptor superfamily, member 19
2027	Agbl5	ENSMUST00000069705.5	ATP/GTP binding protein-like 5
2028	Prtg	ENSMUST00000055535.8	protogenin homolog (Gallus gallus)
2029	Sdk2	ENSMUST00000041627.8	sidekick homolog 2 (chicken)
2030	Erc1	ENSMUST00000183703.2	ELKS/RAB6-interacting/CAST family member 1
2031	Slc25a48	ENSMUST00000021971.5	solute carrier family 25, member 48
2032	Tulp4	ENSMUST00000149756.2	tubby like protein 4
2033	Fam134a	ENSMUST00000097694.5	family with sequence similarity 134, member A
2034	Abl1	ENSMUST00000028190.7	c-abl oncogene 1, non-receptor tyrosine kinase
2035	Frrs1l	ENSMUST00000053681.5	ferric-chelate reductase 1 like
2036	Jph1	ENSMUST00000038382.4	junctophilin 1
2037	Prrxl1	ENSMUST00000189022.1	paired related homeobox protein-like 1
2038	Hs6st3	ENSMUST00000065904.3	heparan sulfate 6-O-sulfotransferase 3

2039	Zfhx4	ENSMUST00000176383.2	zinc finger homeodomain 4
2040	Gucy2e	ENSMUST00000021259.3	guanylate cyclase 2e
2041	Olfr78	ENSMUST00000060187.8	olfactory receptor 78
2042	E130308A19Rik	ENSMUST00000070150.5	RIKEN cDNA E130308A19 gene
2043	Dpysl3	ENSMUST00000121805.2	dihydropyrimidinase-like 3
2044	Zbtb16	ENSMUST00000093852.3	zinc finger and BTB domain containing 16
2045	Megf9	ENSMUST00000107359.3	multiple EGF-like-domains 9
2046	1110002E22Rik	ENSMUST00000163080.2	RIKEN cDNA 1110002E22 gene
2047	Zfp799	ENSMUST00000179695.1	zinc finger protein 799
2048	Hsd17b7	ENSMUST00000027989.7	hydroxysteroid (17-beta) dehydrogenase 7
2049	Trim14	ENSMUST00000046897.7	tripartite motif-containing 14
2050	Reep5	ENSMUST00000006027.5	receptor accessory protein 5
2051	Zfp641	ENSMUST00000023722.6	zinc finger protein 641
2052	Mical2	ENSMUST00000050149.6	microtubule associated monooxygenase, calponin and LIM domain containing 2
2053	Lpin2	ENSMUST00000129635.2	lipin 2
2054	Ids	ENSMUST00000101509.3	iduronate 2-sulfatase
2055	Elovl2	ENSMUST00000021793.8	elongation of very long chain fatty acids (FEN1/Elo2, SUR4/Elo3, yeast)-like 2
2056	Slc45a2	ENSMUST00000117100.2	solute carrier family 45, member 2
2057	Ttpal	ENSMUST00000171696.2	tocopherol (alpha) transfer protein-like
2058	Atm	ENSMUST00000118282.2	ataxia telangiectasia mutated homolog (human)
2059	Vil1	ENSMUST00000027366.7	villin 1
2060	Thbs2	ENSMUST00000170872.1	thrombospondin 2
2061	Slc26a3	ENSMUST00000001254.5	solute carrier family 26, member 3
2062	Rybp	ENSMUST00000101118.2	RING1 and YY1 binding protein
2063	Drd1a	ENSMUST00000021932.5	dopamine receptor D1A
2064	Morn4	ENSMUST00000051772.8	MORN repeat containing 4
2065	Camk2a	ENSMUST00000102888.4	calcium/calmodulin-dependent protein kinase II alpha
2066	Esco1	ENSMUST00000097670.4	establishment of cohesion 1 homolog 1 (S. cerevisiae)
2067	She	ENSMUST00000050401.5	src homology 2 domain-containing transforming protein E
2068	Sstr4	ENSMUST00000109962.3	somatostatin receptor 4
2069	Mpp6	ENSMUST00000166318.2	membrane protein, palmitoylated 6 (MAGUK p55 subfamily member 6)
2070	Cbfa2t3	ENSMUST00000127984.2	core-binding factor, runt domain, alpha subunit 2, translocated to, 3 (human)
2071	Adra1d	ENSMUST00000103184.3	adrenergic receptor, alpha 1d
2072	Sftpa1	ENSMUST00000170719.1	surfactant associated protein A1
2073	Csnk1a1	ENSMUST00000165123.2	casein kinase 1, alpha 1
2074	Rpgrip1l	ENSMUST00000047783.8	Rpgrip1-like
2075	Slitrk2	ENSMUST00000166241.1	SLIT and NTRK-like family, member 2
2076	Mdga2	ENSMUST00000113942.2	MAM domain containing glycosylphosphatidylinositol anchor 2
2077	Prkg1	ENSMUST00000065067.7	protein kinase, cGMP-dependent, type I
2078	Ccdc148	ENSMUST00000077687.5	coiled-coil domain containing 148
2079	Nkrf	ENSMUST00000057093.6	NF-kappaB repressing factor
2080	Srrm4	ENSMUST00000076124.5	serine/arginine repetitive matrix 4
2081	Phtf2	ENSMUST00000118174.2	putative homeodomain transcription factor 2
2082	Gnal	ENSMUST00000025402.8	guanine nucleotide binding protein, alpha stimulating, olfactory type

2083	Btnl9	ENSMUST00000046522.7	butyrophilin-like 9
2084	Pten	ENSMUST00000013807.7	phosphatase and tensin homolog
2085	Trim30b	ENSMUST00000171830.1	tripartite motif-containing 30B
2086	Cacnb4	ENSMUST00000178799.2	calcium channel, voltage-dependent, beta 4 subunit
2087	Nufip2	ENSMUST00000100802.5	nuclear fragile X mental retardation protein interacting protein 2
2088	Suv39h2	ENSMUST00000027956.3	suppressor of variegation 3-9 homolog 2 (Drosophila)
2089	Tgfa	ENSMUST00000032066.9	transforming growth factor alpha
2090	Pcdh1	ENSMUST00000160721.2	protocadherin 1
2091	Cd46	ENSMUST00000160817.2	CD46 antigen, complement regulatory protein
2092	D430041D05Rik	ENSMUST00000089726.4	RIKEN cDNA D430041D05 gene
2093	Cdkn1b	ENSMUST00000067327.5	cyclin-dependent kinase inhibitor 1B
2094	Wnt2	ENSMUST00000010941.3	wingless-related MMTV integration site 2
2095	Mpl	ENSMUST00000102671.4	myeloproliferative leukemia virus oncogene
2096	Fbxo44	ENSMUST00000167160.2	F-box protein 44
2097	AW011738	ENSMUST00000105140.2	expressed sequence AW011738
2098	Ano3	ENSMUST00000099623.4	anoctamin 3
2099	Ece1	ENSMUST00000102518.4	endothelin converting enzyme 1
2100	Bnc2	ENSMUST00000176612.2	basonuclin 2
2101	Cept1	ENSMUST00000068301.5	choline/ethanolaminephosphotransferase 1
2102	Gm10030	ENSMUST00000080872.4	predicted gene 10030
2103	6030458C11Rik	ENSMUST00000057256.3	RIKEN cDNA 6030458C11 gene
2104	Znhit6	ENSMUST00000098534.4	zinc finger, HIT type 6
2105	Nab1	ENSMUST00000186764.1	Ngfi-A binding protein 1
2106	Reps2	ENSMUST00000112334.2	RALBP1 associated Eps domain containing protein 2
2107	Pou2f2	ENSMUST00000108418.5	POU domain, class 2, transcription factor 2
2108	Adamts6	ENSMUST00000065766.6	a disintegrin-like and metallopeptidase (reprolysin type) with thrombospondin type 1 motif, 6
2109	Zc2hc1c	ENSMUST00000059341.4	zinc finger, C2HC-type containing 1C
2110	Ifng	ENSMUST00000068592.3	interferon gamma
2111	Pde6a	ENSMUST00000025468.8	phosphodiesterase 6A, cGMP-specific, rod, alpha
2112	Pggt1b	ENSMUST00000025354.3	protein geranylgeranyltransferase type I, beta subunit
2113	Pigo	ENSMUST00000098109.3	phosphatidylinositol glycan anchor biosynthesis, class O
2114	Tcf12	ENSMUST00000183404.2	transcription factor 12
2115	Shisa6	ENSMUST00000066679.6	shisa homolog 6 (Xenopus laevis)
2116	Syt7	ENSMUST00000169121.2	synaptotagmin VII
2117	Arid2	ENSMUST00000096250.4	AT rich interactive domain 2 (ARID, RFX-like)
2118	Phf3	ENSMUST00000186733.1	PHD finger protein 3
2119	Lclat1	ENSMUST00000067545.6	lysocardiolipin acyltransferase 1
2120	Stam2	ENSMUST00000102759.2	signal transducing adaptor molecule (SH3 domain and ITAM motif) 2
2121	Arsb	ENSMUST00000091403.4	arylsulfatase B
2122	Xpo7	ENSMUST00000167242.2	exportin 7
2123	Rcan3	ENSMUST00000030606.8	regulator of calcineurin 3
2124	Dlg2	ENSMUST00000107196.3	discs, large homolog 2 (Drosophila)
2125	Cyld	ENSMUST00000043526.9	cylindromatosis (turban tumor syndrome)
2126	Zfp944	ENSMUST00000115535.2	zinc finger protein 944
2127	Gpc6	ENSMUST00000078849.5	glypican 6
2128	Cftr	ENSMUST00000045706.6	cystic fibrosis transmembrane conductance regulator
2129	Rab3il1	ENSMUST00000121418.2	RAB3A interacting protein (rabin3)-like 1
2130	Gm53	ENSMUST00000129907.2	predicted gene 53

2131	Dzip1	ENSMUST00000047208.6	DAZ interacting protein 1
2132	Strn	ENSMUST00000145910.3	striatin, calmodulin binding protein
2133	Fmo2	ENSMUST00000045902.7	flavin containing monooxygenase 2
2134	B230359F08Rik	ENSMUST00000103671.2	RIKEN cDNA B230359F08 gene
2135	5730455P16Rik	ENSMUST00000179332.2	RIKEN cDNA 5730455P16 gene
2136	Fam63b	ENSMUST00000049031.5	family with sequence similarity 63, member B
2137	Camk4	ENSMUST00000042868.4	calcium/calmodulin-dependent protein kinase IV
2138	Omp	ENSMUST00000098281.2	olfactory marker protein
2139	Kctd3	ENSMUST00000085678.2	potassium channel tetramerisation domain containing 3
2140	Slc10a5	ENSMUST00000078748.3	solute carrier family 10 (sodium/bile acid cotransporter family), member 5
2141	Ubxn7	ENSMUST00000115151.3	UBX domain protein 7
2142	Eya4	ENSMUST00000092665.5	eyes absent 4 homolog (Drosophila)
2143	Pik3c2a	ENSMUST00000170430.1	phosphatidylinositol 3-kinase, C2 domain containing, alpha polypeptide
2144	Zscan30	ENSMUST00000115829.1	zinc finger and SCAN domain containing 30
2145	Wdr31	ENSMUST00000120095.2	WD repeat domain 31
2146	Primpol	ENSMUST00000040468.9	primase and polymerase (DNA-directed)
2147	Lipt2	ENSMUST00000032967.3	lipoyl(octanoyl) transferase 2 (putative)
2148	Ankrd61	ENSMUST00000079624.6	ankyrin repeat domain 61
2149	Brcc3	ENSMUST00000033544.8	BRCA1/BRCA2-containing complex, subunit 3
2150	Aff2	ENSMUST00000033532.6	AF4/FMR2 family, member 2
2151	Col19a1	ENSMUST00000115244.3	collagen, type XIX, alpha 1
2152	Rnf157	ENSMUST00000100202.4	ring finger protein 157
2153	Pcdhb1	ENSMUST00000052366.3	protocadherin beta 1
2154	Sox9	ENSMUST00000000579.2	SRY (sex determining region Y)-box 9
2155	Naip6	ENSMUST00000118574.2	NLR family, apoptosis inhibitory protein 6
2156	Parp14	ENSMUST00000042665.8	poly (ADP-ribose) polymerase family, member 14
2157	Rpusd2	ENSMUST00000028796.1	RNA pseudouridylate synthase domain containing 2
2158	Thoc2	ENSMUST00000047037.9	THO complex 2
2159	Cntnap3	ENSMUST00000091554.4	contactin associated protein-like 3
2160	Dcp1a	ENSMUST00000022535.7	DCP1 decapping enzyme homolog A (S. cerevisiae)
2161	Ccdc6	ENSMUST00000147545.2	coiled-coil domain containing 6
2162	Ppp2r2a	ENSMUST00000089230.5	protein phosphatase 2, regulatory subunit B, alpha
2163	Phip	ENSMUST00000034787.6	pleckstrin homology domain interacting protein
2164	Cisd1	ENSMUST00000045887.8	CDGSH iron sulfur domain 1
2165	Epb4.1l5	ENSMUST00000191046.1	erythrocyte protein band 4.1-like 5
2166	Soat1	ENSMUST00000189661.1	sterol O-acyltransferase 1
2167	Pde8b	ENSMUST00000162412.2	phosphodiesterase 8B
2168	Mat2a	ENSMUST00000059472.8	methionine adenosyltransferase II, alpha
2169	Vcpip1	ENSMUST00000057438.6	valosin containing protein (p97)/p47 complex interacting protein 1
2170	Csrnp2	ENSMUST00000061457.5	cysteine-serine-rich nuclear protein 2
2171	5830462I19Rik	ENSMUST00000168137.1	RIKEN cDNA 5830462I19 gene
2172	Adam28	ENSMUST00000111072.2	a disintegrin and metallopeptidase domain 28
2173	Acnat2	ENSMUST00000081541.3	acyl-coenzyme A amino acid N-acyltransferase 2
2174	Tmem2	ENSMUST00000096194.3	transmembrane protein 2
2175	Car5b	ENSMUST00000033739.4	carbonic anhydrase 5b, mitochondrial
2176	Lin28b	ENSMUST00000079390.6	lin-28 homolog B (C. elegans)
2177	Siah2	ENSMUST00000070368.7	seven in absentia 2
2178	Diap3	ENSMUST00000022599.8	diaphanous homolog 3 (Drosophila)

2179	Enpp4	ENSMUST00000024757.8	ectonucleotide pyrophosphatase/phosphodiesterase 4
2180	Mark3	ENSMUST00000084953.6	MAP/microtubule affinity-regulating kinase 3
2181	Ypel2	ENSMUST00000018571.4	yippee-like 2 (Drosophila)
2182	Xcr1	ENSMUST00000182350.1	chemokine (C motif) receptor 1
2183	Kif26b	ENSMUST00000160789.1	kinesin family member 26B
2184	Tktl2	ENSMUST00000183187.1	transketolase-like 2
2185	Ccdc85c	ENSMUST00000136175.1	coiled-coil domain containing 85C
2186	Olfr558	ENSMUST00000094124.3	olfactory receptor 558
2187	Dppa2	ENSMUST00000097175.4	developmental pluripotency associated 2
2188	Hn1l	ENSMUST00000024981.7	hematological and neurological expressed 1-like
2189	Gm4245	ENSMUST00000109027.3	predicted gene 4245
2190	Ikzf4	ENSMUST00000133342.2	IKAROS family zinc finger 4
2191	Pcgf5	ENSMUST00000071267.7	polycomb group ring finger 5
2192	Cd36	ENSMUST00000082367.7	CD36 antigen
2193	Strbp	ENSMUST00000028279.4	spermatid perinuclear RNA binding protein
2194	Amer2	ENSMUST00000022561.6	APC membrane recruitment 2
2195	Dtd2	ENSMUST00000021339.7	D-tyrosyl-tRNA deacylase 2
2196	Dcdc2a	ENSMUST00000069614.6	doublecortin domain containing 2a
2197	Hspa13	ENSMUST00000114244.1	heat shock protein 70 family, member 13
2198	Ovol1	ENSMUST00000025861.1	OVO homolog-like 1 (Drosophila)
2199	Bcl6	ENSMUST00000023151.5	B cell leukemia/lymphoma 6
2200	Peli3	ENSMUST00000120475.2	pellino 3
2201	Slc25a32	ENSMUST00000022908.8	solute carrier family 25, member 32
2202	Veph1	ENSMUST00000029419.7	ventricular zone expressed PH domain-containing 1
2203	Iqcf5	ENSMUST00000085113.4	IQ motif containing F5
2204	Gm6133	ENSMUST00000164064.1	predicted gene 6133
2205	Slc30a7	ENSMUST00000067485.3	solute carrier family 30 (zinc transporter), member 7
2206	Naa25	ENSMUST00000042163.9	N(alpha)-acetyltransferase 25, NatB auxiliary subunit
2207	Elp3	ENSMUST00000022609.5	elongator acetyltransferase complex subunit 3
2208	Gm14434	ENSMUST00000122097.3	predicted gene 14434
2209	Axin2	ENSMUST00000052915.8	axin2
2210	Gm4724	ENSMUST00000109045.3	predicted gene 4724
2211	Ppip5k2	ENSMUST00000042509.7	diphosphoinositol pentakisphosphate kinase 2
2212	Bmf	ENSMUST00000090219.7	BCL2 modifying factor
2213	Pcdh9	ENSMUST00000068992.2	protocadherin 9
2214	Sept9	ENSMUST00000093907.5	septin 9
2215	Cnn3	ENSMUST00000029773.8	calponin 3, acidic
2216	Iws1	ENSMUST00000025243.3	IWS1 homolog (S. cerevisiae)
2217	Irs1	ENSMUST00000069799.2	insulin receptor substrate 1
2218	Pigk	ENSMUST00000159899.2	phosphatidylinositol glycan anchor biosynthesis, class K
2219	Mcm3	ENSMUST00000053266.10	minichromosome maintenance deficient 3 (S. cerevisiae)
2220	Sox5	ENSMUST00000111748.2	SRY (sex determining region Y)-box 5
2221	Emc8	ENSMUST00000181950.1	ER membrane protein complex subunit 8
2222	Fbxl20	ENSMUST00000103143.4	F-box and leucine-rich repeat protein 20
2223	Magi2	ENSMUST00000101558.4	membrane associated guanylate kinase, WW and PDZ domain containing 2
2224	Ociad1	ENSMUST00000071081.7	OCIA domain containing 1
2225	Slfn5	ENSMUST00000067443.3	schlafen 5
2226	Pik3r1	ENSMUST00000055518.7	phosphatidylinositol 3-kinase, regulatory subunit, polypeptide 1 (p85 alpha)

2227	Spry4	ENSMUST00000025295.6	sprouty homolog 4 (Drosophila)
2228	Fgd4	ENSMUST00000161861.2	FYVE, RhoGEF and PH domain containing 4
2229	Ilf3	ENSMUST00000115414.1	interleukin enhancer binding factor 3
2230	Scamp1	ENSMUST00000022197.9	secretory carrier membrane protein 1
2231	Adat1	ENSMUST00000034427.6	adenosine deaminase, tRNA-specific 1
2232	D15Ertd621e	ENSMUST00000037270.3	DNA segment, Chr 15, ERATO Doi 621, expressed
2233	Fam184a	ENSMUST00000020003.8	family with sequence similarity 184, member A
2234	Prpf4b	ENSMUST00000077853.3	PRP4 pre-mRNA processing factor 4 homolog B (yeast)
2235	Mllt3	ENSMUST00000078090.6	myeloid/lymphoid or mixed-lineage leukemia (trithorax homolog, Drosophila); translocated to, 3
2236	Zc3h7b	ENSMUST00000109554.1	zinc finger CCCH type containing 7B
2237	Gbp11	ENSMUST00000171587.1	guanylate binding protein 11
2238	Epg5	ENSMUST00000044622.5	ectopic P-granules autophagy protein 5 homolog (C. elegans)
2239	Col22a1	ENSMUST00000159993.2	collagen, type XXII, alpha 1
2240	Epn2	ENSMUST00000108711.2	epsin 2
2241	Zfp316	ENSMUST00000161448.2	zinc finger protein 316
2242	Nyap2	ENSMUST00000123285.1	neuronal tyrosine-phophorylated phosphoinositide 3-kinase adaptor 2
2243	Nrxn1	ENSMUST00000160844.4	neurexin I
2244	Gm6086	ENSMUST00000097632.4	predicted gene 6086
2245	Csmd1	ENSMUST00000082104.6	CUB and Sushi multiple domains 1
2246	Gm14305	ENSMUST00000108981.3	predicted gene 14305
2247	Pak7	ENSMUST00000035264.3	p21 protein (Cdc42/Rac)-activated kinase 7
2248	Neu4	ENSMUST00000050890.7	sialidase 4
2249	4921511H03Rik	ENSMUST00000160634.1	RIKEN cDNA 4921511H03 gene
2250	Pak3	ENSMUST00000172330.2	p21 protein (Cdc42/Rac)-activated kinase 3
2251	Megf10	ENSMUST00000075770.7	multiple EGF-like-domains 10
2252	Six4	ENSMUST00000043208.7	sine oculis-related homeobox 4
2253	Map3k1	ENSMUST00000109267.3	mitogen-activated protein kinase kinase kinase 1
2254	Nlgn3	ENSMUST00000118111.2	neuroligin 3
2255	Satb2	ENSMUST00000042857.8	special AT-rich sequence binding protein 2
2256	Npnt	ENSMUST00000042744.10	nephronectin
2257	Ddx51	ENSMUST00000031478.5	DEAD (Asp-Glu-Ala-Asp) box polypeptide 51
2258	Ednrb	ENSMUST00000022718.4	endothelin receptor type B
2259	Amd1	ENSMUST00000099945.4	S-adenosylmethionine decarboxylase 1
2260	Gucy2f	ENSMUST00000042530.3	guanylate cyclase 2f
2261	Hgs	ENSMUST00000106205.2	HGF-regulated tyrosine kinase substrate
2262	Nrp1	ENSMUST00000026917.8	neuropilin 1
2263	Ralgps1	ENSMUST00000042615.7	Ral GEF with PH domain and SH3 binding motif 1
2264	Btbd9	ENSMUST00000168787.2	BTB (POZ) domain containing 9
2265	Foxo3	ENSMUST00000105502.2	forkhead box O3
2266	Wasf2	ENSMUST00000084241.6	WAS protein family, member 2
2267	Mtss1	ENSMUST00000080371.6	metastasis suppressor 1
2268	Zfyve20	ENSMUST00000014694.8	zinc finger, FYVE domain containing 20
2269	Amer1	ENSMUST00000084535.5	APC membrane recruitment 1
2270	Tmem178b	ENSMUST00000061740.7	transmembrane protein 178B
2271	Gm10615	ENSMUST00000098355.3	predicted gene 10615
2272	Pcdh12	ENSMUST00000025311.5	protocadherin 12
2273	Tnpo2	ENSMUST00000093360.5	transportin 2 (importin 3, karyopherin beta 2b)
2274	Ap3s2	ENSMUST00000075657.6	adaptor-related protein complex 3, sigma 2 subunit

2275	Fsd1	ENSMUST00000011733.8	fibronectin type 3 and SPRY domain-containing protein
2276	Msantd3	ENSMUST00000107704.1	Myb/SANT-like DNA-binding domain containing 3
2277	Sema3c	ENSMUST00000030568.8	sema domain, immunoglobulin domain (Ig), short basic domain, secreted, (semaphorin) 3C
2278	Gcc2	ENSMUST00000057659.8	GRIP and coiled-coil domain containing 2
2279	Kcnv1	ENSMUST00000022967.5	potassium channel, subfamily V, member 1
2280	Snai2	ENSMUST00000023356.6	snail homolog 2 (Drosophila)
2281	Pdpr	ENSMUST00000039333.4	pyruvate dehydrogenase phosphatase regulatory subunit
2282	Wdfy2	ENSMUST00000014691.8	WD repeat and FYVE domain containing 2
2283	Kirrel	ENSMUST00000159976.2	kin of IRRE like (Drosophila)
2284	Apba1	ENSMUST00000025830.7	amyloid beta (A4) precursor protein binding, family A, member 1
2285	Rab33b	ENSMUST00000054387.6	RAB33B, member RAS oncogene family
2286	Olfr912	ENSMUST00000071681.2	olfactory receptor 912
2287	Ehd3	ENSMUST00000024860.7	EH-domain containing 3
2288	Oas1a	ENSMUST00000080322.7	2'-5' oligoadenylate synthetase 1A
2289	Gm10176	ENSMUST00000159707.1	predicted gene 10176
2290	Hoxa4	ENSMUST00000101395.2	homeobox A4
2291	Mocs1	ENSMUST00000024797.10	molybdenum cofactor synthesis 1
2292	Slc39a14	ENSMUST00000068044.8	solute carrier family 39 (zinc transporter), member 14
2293	Tgfbr2	ENSMUST00000061101.5	transforming growth factor, beta receptor II
2294	Gstt2	ENSMUST00000038257.6	glutathione S-transferase, theta 2
2295	Itga9	ENSMUST00000044165.8	integrin alpha 9
2296	Fam168a	ENSMUST00000049053.7	family with sequence similarity 168, member A
2297	Gpr116	ENSMUST00000113599.1	G protein-coupled receptor 116
2298	Mak	ENSMUST00000165087.2	male germ cell-associated kinase
2299	Plxnc1	ENSMUST00000099337.3	plexin C1
2300	Mid2	ENSMUST00000112990.2	midline 2
2301	Bcap29	ENSMUST00000177962.2	B cell receptor associated protein 29
2302	Mavs	ENSMUST00000041362.6	mitochondrial antiviral signaling protein
2303	Pard3	ENSMUST00000162907.2	par-3 family cell polarity regulator
2304	Mettl16	ENSMUST00000141755.2	methyltransferase like 16
2305	Zdhhc23	ENSMUST00000036321.8	zinc finger, DHHC domain containing 23
2306	Xpo4	ENSMUST00000174545.2	exportin 4
2307	Ildr2	ENSMUST00000111416.1	immunoglobulin-like domain containing receptor 2
2308	Spsb1	ENSMUST00000038562.3	splA/ryanodine receptor domain and SOCS box containing 1
2309	Ago2	ENSMUST00000044113.10	argonaute RISC catalytic subunit 2
2310	Prkaa2	ENSMUST00000030243.7	protein kinase, AMP-activated, alpha 2 catalytic subunit
2311	Zfp26	ENSMUST00000159569.2	zinc finger protein 26
2312	Cxcl14	ENSMUST00000021970.9	chemokine (C-X-C motif) ligand 14
2313	Chml	ENSMUST00000104984.1	choroideremia-like
2314	Gm14419	ENSMUST00000126358.2	predicted gene 14419
2315	Mrgprb1	ENSMUST00000094384.3	MAS-related GPR, member B1
2316	Rab3gap1	ENSMUST00000037649.5	RAB3 GTPase activating protein subunit 1
2317	Lipg	ENSMUST00000066532.4	lipase, endothelial
2318	Fam84a	ENSMUST00000020926.6	family with sequence similarity 84, member A
2319	Otud7b	ENSMUST00000090785.3	OTU domain containing 7B
2320	Zfp395	ENSMUST00000066994.6	zinc finger protein 395

2321	Pak2	ENSMUST00000023467.8	p21 protein (Cdc42/Rac)-activated kinase 2
2322	Apbb2	ENSMUST00000159786.2	amyloid beta (A4) precursor protein-binding, family B, member 2
2323	Syt14	ENSMUST00000016344.7	synaptotagmin XIV
2324	Trhde	ENSMUST00000061632.7	TRH-degrading enzyme
2325	Ppat	ENSMUST00000140076.1	phosphoribosyl pyrophosphate amidotransferase
2326	Zdhhc20	ENSMUST00000089473.3	zinc finger, DHHC domain containing 20
2327	Auts2	ENSMUST00000161804.2	autism susceptibility candidate 2
2328	Msl2	ENSMUST00000085177.4	male-specific lethal 2 homolog (Drosophila)
2329	Efna5	ENSMUST00000076840.6	ephrin A5
2330	Usp7	ENSMUST00000160405.2	ubiquitin specific peptidase 7
2331	Angptl2	ENSMUST00000004208.5	angiopoietin-like 2
2332	Cct6b	ENSMUST00000021040.4	chaperonin containing Tcp1, subunit 6b (zeta)
2333	Htr6	ENSMUST00000105802.2	5-hydroxytryptamine (serotonin) receptor 6
2334	Rassf10	ENSMUST00000182858.1	Ras association (RalGDS/AF-6) domain family (N-terminal) member 10
2335	Eda	ENSMUST00000113776.2	ectodysplasin-A
2336	Csrnp3	ENSMUST00000122912.2	cysteine-serine-rich nuclear protein 3
2337	Nlrp4a	ENSMUST00000068767.4	NLR family, pyrin domain containing 4A
2338	B4galnt2	ENSMUST00000038343.6	beta-1,4-N-acetyl-galactosaminyl transferase 2
2339	Rapgef6	ENSMUST00000102743.4	Rap guanine nucleotide exchange factor (GEF) 6
2340	Fam20b	ENSMUST00000122424.2	family with sequence similarity 20, member B
2341	Gpr35	ENSMUST00000169198.2	G protein-coupled receptor 35
2342	Srsf7	ENSMUST00000063417.9	serine/arginine-rich splicing factor 7
2343	Efhc1	ENSMUST00000038447.5	EF-hand domain (C-terminal) containing 1
2344	Usp31	ENSMUST00000046929.6	ubiquitin specific peptidase 31
2345	Nkapl	ENSMUST00000068235.5	NFKB activating protein-like
2346	4930550C14Rik	ENSMUST00000005262.1	RIKEN cDNA 4930550C14 gene
2347	Rnf169	ENSMUST00000080817.4	ring finger protein 169
2348	Spag9	ENSMUST00000041956.8	sperm associated antigen 9
2349	Atl3	ENSMUST00000170373.2	atlastin GTPase 3
2350	Acat1	ENSMUST00000034547.5	acetyl-Coenzyme A acetyltransferase 1
2351	Spns2	ENSMUST00000045303.4	spinster homolog 2
2352	Zfp935	ENSMUST00000076195.5	zinc finger protein 935
2353	Gabarapl1	ENSMUST00000032264.6	gamma-aminobutyric acid (GABA) A receptor-associated protein-like 1
2354	Rsc1a1	ENSMUST00000105782.1	regulatory solute carrier protein, family 1, member 1
2355	Mdm4	ENSMUST00000188090.1	transformed mouse 3T3 cell double minute 4
2356	Gm12695	ENSMUST00000107071.1	predicted gene 12695
2357	Sox6	ENSMUST00000166877.2	SRY (sex determining region Y)-box 6
2358	Tdgf1	ENSMUST00000035075.8	teratocarcinoma-derived growth factor 1
2359	Spred1	ENSMUST00000028829.7	sprouty protein with EVH-1 domain 1, related sequence
2360	Masp1	ENSMUST00000089883.5	mannan-binding lectin serine peptidase 1
2361	Usp49	ENSMUST00000024779.9	ubiquitin specific peptidase 49
2362	Nalcn	ENSMUST00000000201.5	sodium leak channel, non-selective
2363	Dmc1	ENSMUST00000023065.6	DMC1 dosage suppressor of mck1 homolog, meiosis-specific homologous recombination
2364	Cplx2	ENSMUST00000026985.8	complexin 2
2365	Cdkl2	ENSMUST00000086978.6	cyclin-dependent kinase-like 2 (CDC2-related kinase)
2366	Slc38a9	ENSMUST00000052514.4	solute carrier family 38, member 9
2367	Tmem25	ENSMUST00000114705.2	transmembrane protein 25

2368	Pcsk1	ENSMUST00000022075.4	proprotein convertase subtilisin/kexin type 1
2369	Stx17	ENSMUST00000107720.3	syntaxin 17
2370	Osbpl3	ENSMUST00000114468.3	oxysterol binding protein-like 3
2371	Cyp2d12	ENSMUST00000068861.6	cytochrome P450, family 2, subfamily d, polypeptide 12
2372	Zdhhc24	ENSMUST00000006632.7	zinc finger, DHHC domain containing 24
2373	Slc15a4	ENSMUST00000031367.9	solute carrier family 15, member 4
2374	Btbd7	ENSMUST00000045652.6	BTB (POZ) domain containing 7
2375	Krtap6-5	ENSMUST00000074637.2	keratin associated protein 6-5
2376	4930453N24Rik	ENSMUST00000076991.6	RIKEN cDNA 4930453N24 gene
2377	Scube1	ENSMUST00000171496.2	signal peptide, CUB domain, EGF-like 1
2378	Ttc39b	ENSMUST00000102823.4	tetratricopeptide repeat domain 39B
2379	Lman2	ENSMUST00000021940.7	lectin, mannose-binding 2
2380	E430018J23Rik	ENSMUST00000074249.6	RIKEN cDNA E430018J23 gene
2381	Prkca	ENSMUST00000059595.5	protein kinase C, alpha
2382	Slco5a1	ENSMUST00000188454.1	solute carrier organic anion transporter family, member 5A1
2383	Amd2	ENSMUST00000080898.2	S-adenosylmethionine decarboxylase 2
2384	Gng12	ENSMUST00000043148.7	guanine nucleotide binding protein (G protein), gamma 12
2385	4930578C19Rik	ENSMUST00000044188.4	RIKEN cDNA 4930578C19 gene
2386	Gan	ENSMUST00000064488.5	giant axonal neuropathy
2387	Rhpn2	ENSMUST00000032705.7	rhophilin, Rho GTPase binding protein 2
2388	Tada2b	ENSMUST00000031097.7	transcriptional adaptor 2B
2389	Ubxn2a	ENSMUST00000020962.6	UBX domain protein 2A
2390	Aff3	ENSMUST00000095027.3	AF4/FMR2 family, member 3
2391	Chst13	ENSMUST00000070890.4	carbohydrate (chondroitin 4) sulfotransferase 13
2392	Smoc1	ENSMUST00000110347.3	SPARC related modular calcium binding 1
2393	Trim33	ENSMUST00000029444.7	tripartite motif-containing 33
2394	Adal	ENSMUST00000028702.4	adenosine deaminase-like
2395	Intu	ENSMUST00000091186.3	inturned planar cell polarity effector homolog (Drosophila)
2396	Fam105a	ENSMUST00000100739.3	family with sequence similarity 105, member A
2397	Tmem251	ENSMUST00000057416.6	transmembrane protein 251
2398	Ino80d	ENSMUST00000097718.3	INO80 complex subunit D
2399	Vdac1	ENSMUST00000102758.2	voltage-dependent anion channel 1
2400	Samd5	ENSMUST00000100070.2	sterile alpha motif domain containing 5
2401	Mkln1	ENSMUST00000026699.9	muskelin 1, intracellular mediator containing kelch motifs
2402	Ttc14	ENSMUST00000099153.4	tetratricopeptide repeat domain 14
2403	Fam83b	ENSMUST00000183437.2	family with sequence similarity 83, member B
2404	Tox4	ENSMUST00000022766.6	TOX high mobility group box family member 4
2405	Timm10b	ENSMUST00000106783.2	translocase of inner mitochondrial membrane 10B
2406	Syt4	ENSMUST00000025110.3	synaptotagmin IV
2407	Fbn2	ENSMUST00000025497.6	fibrillin 2
2408	Tead1	ENSMUST00000059768.11	TEA domain family member 1
2409	Hs6st2	ENSMUST00000088172.6	heparan sulfate 6-O-sulfotransferase 2
2410	Usp3	ENSMUST00000127569.2	ubiquitin specific peptidase 3
2411	Yap1	ENSMUST00000086580.6	yes-associated protein 1
2412	Cap2	ENSMUST00000021802.9	CAP, adenylate cyclase-associated protein, 2 (yeast)
2413	Calm1	ENSMUST00000110082.4	calmodulin 1
2414	Sec16a	ENSMUST00000114082.3	SEC16 homolog A (S. cerevisiae)

2415	Ptbp2	ENSMUST00000029780.7	polypyrimidine tract binding protein 2
2416	Tpd52l2	ENSMUST00000149163.2	tumor protein D52-like 2
2417	Gramd1b	ENSMUST00000121357.2	GRAM domain containing 1B
2418	Abcb10	ENSMUST00000075578.6	ATP-binding cassette, sub-family B (MDR/TAP), member 10
2419	Lurap1	ENSMUST00000030469.4	leucine rich adaptor protein 1
2420	Clmn	ENSMUST00000109937.2	calmin
2421	Fxyd3	ENSMUST00000167369.2	FXYD domain-containing ion transport regulator 3
2422	Igf1r	ENSMUST00000005671.8	insulin-like growth factor I receptor
2423	Rab6a	ENSMUST00000107048.2	RAB6A, member RAS oncogene family
2424	Prex2	ENSMUST00000027056.6	phosphatidylinositol-3,4,5-trisphosphate-dependent Rac exchange factor 2
2425	Sec24a	ENSMUST00000109097.3	Sec24 related gene family, member A (S. cerevisiae)
2426	Ythdf1	ENSMUST00000037299.9	YTH domain family 1
2427	Vps52	ENSMUST00000025178.10	vacuolar protein sorting 52 (yeast)
2428	Sbspon	ENSMUST00000040695.4	somatomedin B and thrombospondin, type 1 domain containing
2429	Mansc1	ENSMUST00000047443.3	MANSC domain containing 1
2430	Slc26a7	ENSMUST00000042221.8	solute carrier family 26, member 7
2431	Mprip	ENSMUST00000116371.2	myosin phosphatase Rho interacting protein
2432	Mylk4	ENSMUST00000057428.5	myosin light chain kinase family, member 4
2433	Slitrk4	ENSMUST00000069926.8	SLIT and NTRK-like family, member 4
2434	Ric8b	ENSMUST00000038523.8	resistance to inhibitors of cholinesterase 8 homolog B (C. elegans)
2435	Rnd3	ENSMUST00000154545.1	Rho family GTPase 3
2436	Lrrc9	ENSMUST00000162159.2	leucine rich repeat containing 9
2437	Ccdc50	ENSMUST00000100026.4	coiled-coil domain containing 50
2438	Wdfy1	ENSMUST00000113512.2	WD repeat and FYVE domain containing 1
2439	Pigh	ENSMUST00000072154.7	phosphatidylinositol glycan anchor biosynthesis, class H
2440	Faf1	ENSMUST00000102724.4	Fas-associated factor 1
2441	Aox3	ENSMUST00000040999.8	aldehyde oxidase 3
2442	Snx27	ENSMUST00000029783.10	sorting nexin family member 27
2443	Trim71	ENSMUST00000111816.2	tripartite motif-containing 71
2444	Crtc3	ENSMUST00000122255.2	CREB regulated transcription coactivator 3
2445	Murc	ENSMUST00000030033.4	muscle-related coiled-coil protein
2446	Peli2	ENSMUST00000073150.4	pellino 2
2447	Rps12	ENSMUST00000073926.6	ribosomal protein S12
2448	Zfp661	ENSMUST00000110366.2	zinc finger protein 661
2449	Man2a1	ENSMUST00000086723.4	mannosidase 2, alpha 1
2450	A930033H14Rik	ENSMUST00000170048.1	RIKEN cDNA A930033H14 gene
2451	Ttc9	ENSMUST00000036116.5	tetratricopeptide repeat domain 9
2452	Med1	ENSMUST00000107545.3	mediator complex subunit 1
2453	Dio2	ENSMUST00000082432.3	deiodinase, iodothyronine, type II
2454	Ankrd40	ENSMUST00000051221.7	ankyrin repeat domain 40
2455	M5C1000I18Rik	ENSMUST00000068526.6	RIKEN cDNA M5C1000I18 gene
2456	Wdr52	ENSMUST00000099742.3	WD repeat domain 52
2457	Rps19	ENSMUST00000108428.2	ribosomal protein S19
2458	Homer2	ENSMUST00000026922.8	homer homolog 2 (Drosophila)
2459	Racgap1	ENSMUST00000171702.2	Rac GTPase-activating protein 1
2460	Hook3	ENSMUST00000037182.8	hook homolog 3 (Drosophila)
2461	Tfec	ENSMUST00000031533.7	transcription factor EC

2462	Tnfsf10	ENSMUST00000046383.6	tumor necrosis factor (ligand) superfamily, member 10
2463	Nhlrc2	ENSMUST00000071423.5	NHL repeat containing 2
2464	Gcsam	ENSMUST00000023339.4	germinal center associated, signaling and motility
2465	Sphkap	ENSMUST00000160953.2	SPHK1 interactor, AKAP domain containing
2466	Cdc26	ENSMUST00000084525.6	cell division cycle 26
2467	Sestd1	ENSMUST00000102660.2	SEC14 and spectrin domains 1
2468	Polg	ENSMUST00000073889.8	polymerase (DNA directed), gamma
2469	Naa15	ENSMUST00000029303.7	N(alpha)-acetyltransferase 15, NatA auxiliary subunit
2470	C330018D20Rik	ENSMUST00000025488.9	RIKEN cDNA C330018D20 gene
2471	Ska3	ENSMUST00000022536.2	spindle and kinetochore associated complex subunit 3
2472	Eef2k	ENSMUST00000047875.9	eukaryotic elongation factor-2 kinase
2473	Sdcbp	ENSMUST00000029912.5	syndecan binding protein
2474	Zfp879	ENSMUST00000109134.2	zinc finger protein 879
2475	Tmc1	ENSMUST00000039500.3	transmembrane channel-like gene family 1
2476	Tmem151b	ENSMUST00000180252.1	transmembrane protein 151B
2477	Scrt2	ENSMUST00000064061.3	scratch homolog 2, zinc finger protein (Drosophila)
2478	Sec14l3	ENSMUST00000068322.6	SEC14-like 3 (S. cerevisiae)
2479	Hsf2	ENSMUST00000079833.4	heat shock factor 2
2480	Mettl11b	ENSMUST00000159679.2	methyltransferase like 11B
2481	Rora	ENSMUST00000034766.8	RAR-related orphan receptor alpha
2482	Lpar2	ENSMUST00000034325.4	lysophosphatidic acid receptor 2
2483	Fbxo3	ENSMUST00000111136.2	F-box protein 3
2484	Leap2	ENSMUST00000036045.5	liver-expressed antimicrobial peptide 2
2485	Tmem69	ENSMUST00000106478.3	transmembrane protein 69
2486	Rin3	ENSMUST00000056950.8	Ras and Rab interactor 3
2487	Scyl3	ENSMUST00000027876.5	SCY1-like 3 (S. cerevisiae)
2488	Hsbp1l1	ENSMUST00000166219.1	heat shock factor binding protein 1-like 1
2489	Rfx3	ENSMUST00000165566.2	regulatory factor X, 3 (influences HLA class II expression)
2490	2810007J24Rik	ENSMUST00000168252.3	RIKEN cDNA 2810007J24 gene
2491	Fbxo28	ENSMUST00000051431.4	F-box protein 28
2492	Aasdhppt	ENSMUST00000051589.7	aminoadipate-semialdehyde dehydrogenase-phosphopantetheinyl transferase
2493	Gm14403	ENSMUST00000108940.2	predicted gene 14403
2494	Pcdhb19	ENSMUST00000059571.5	protocadherin beta 19
2495	Pik3cd	ENSMUST00000038859.8	phosphatidylinositol 3-kinase catalytic delta polypeptide
2496	Med13	ENSMUST00000043624.8	mediator complex subunit 13
2497	Pgm2l1	ENSMUST00000084935.4	phosphoglucomutase 2-like 1
2498	Rps26-ps1	ENSMUST00000077208.4	ribosomal protein S26, pseudogene 1
2499	Ss18	ENSMUST00000040964.7	synovial sarcoma translocation, Chromosome 18
2500	Klhl7	ENSMUST00000163409.2	kelch-like 7
2501	Cdyl2	ENSMUST00000109102.2	chromodomain protein, Y chromosome-like 2
2502	Eif3j1	ENSMUST00000028668.7	eukaryotic translation initiation factor 3, subunit J1
2503	Bckdk	ENSMUST00000071056.8	branched chain ketoacid dehydrogenase kinase
2504	Nell1	ENSMUST00000081872.6	NEL-like 1
2505	Chst7	ENSMUST00000044138.7	carbohydrate (N-acetylglucosamino) sulfotransferase 7
2506	Susd5	ENSMUST00000135338.1	sushi domain containing 5
2507	Acpp	ENSMUST00000062723.7	acid phosphatase, prostate
2508	Ccdc120	ENSMUST00000033490.7	coiled-coil domain containing 120
2509	Ube2z	ENSMUST00000100528.4	ubiquitin-conjugating enzyme E2Z (putative)

2510	Vwa5b1	ENSMUST00000030533.6	von Willebrand factor A domain containing 5B1
2511	Dmrt3	ENSMUST00000048935.4	doublesex and mab-3 related transcription factor 3
2512	Dennd1a	ENSMUST00000102787.4	DENN/MADD domain containing 1A
2513	Fktn	ENSMUST00000128667.2	fukutin
2514	Hdx	ENSMUST00000113422.3	highly divergent homeobox
2515	Smarca2	ENSMUST00000025862.8	SWI/SNF related, matrix associated, actin dependent regulator of chromatin, subfamily a, member 2
2516	Gabrg1	ENSMUST00000031119.1	gamma-aminobutyric acid (GABA) A receptor, subunit gamma 1
2517	Ctif	ENSMUST00000165559.1	CBP80/20-dependent translation initiation factor
2518	Itgb3	ENSMUST00000021028.4	integrin beta 3
2519	Skint4	ENSMUST00000106564.2	selection and upkeep of intraepithelial T cells 4
2520	Lyg2	ENSMUST00000078307.5	lysozyme G-like 2
2521	Ttc34	ENSMUST00000050220.2	tetratricopeptide repeat domain 34
2522	Mpp2	ENSMUST00000017458.5	membrane protein, palmitoylated 2 (MAGUK p55 subfamily member 2)
2523	Aim1	ENSMUST00000020017.8	absent in melanoma 1
2524	Syt9	ENSMUST00000073459.6	synaptotagmin IX
2525	Cmtm6	ENSMUST00000035007.8	CKLF-like MARVEL transmembrane domain containing 6
2526	Lzts1	ENSMUST00000185176.2	leucine zipper, putative tumor suppressor 1
2527	Maneal	ENSMUST00000064444.7	mannosidase, endo-alpha-like
2528	Tssc1	ENSMUST00000035657.7	tumor suppressing subtransferable candidate 1
2529	Tmem43	ENSMUST00000032183.4	transmembrane protein 43
2530	Mfn2	ENSMUST00000105716.3	mitofusin 2
2531	B3gnt1	ENSMUST00000053705.6	UDP-GlcNAc:betaGal beta-1,3-N-acetylglucosaminyltransferase 1
2532	Greb1l	ENSMUST00000048977.9	growth regulation by estrogen in breast cancer-like
2533	Kcnj10	ENSMUST00000056136.3	potassium inwardly-rectifying channel, subfamily J, member 10
2534	Zer1	ENSMUST00000044751.9	zyg-11 related, cell cycle regulator
2535	Oxsr1	ENSMUST00000040853.5	oxidative-stress responsive 1
2536	Capn6	ENSMUST00000087316.5	calpain 6
2537	Tfcp2l1	ENSMUST00000027629.9	transcription factor CP2-like 1
2538	Hecw1	ENSMUST00000110516.2	HECT, C2 and WW domain containing E3 ubiquitin protein ligase 1
2539	Swap70	ENSMUST00000033325.7	SWA-70 protein
2540	Spock1	ENSMUST00000185502.1	sparc/osteonectin, cwcv and kazal-like domains proteoglycan 1
2541	Adamtsl3	ENSMUST00000173287.2	ADAMTS-like 3
2542	Manba	ENSMUST00000029814.9	mannosidase, beta A, lysosomal
2543	Ctdsp2	ENSMUST00000105256.3	CTD (carboxy-terminal domain, RNA polymerase II, polypeptide A) small phosphatase 2
2544	Nrg3	ENSMUST00000166968.3	neuregulin 3
2545	Nr6a1	ENSMUST00000168098.2	nuclear receptor subfamily 6, group A, member 1
2546	Ttl	ENSMUST00000035812.8	tubulin tyrosine ligase
2547	Pkp1	ENSMUST00000027667.7	plakophilin 1
2548	Irs4	ENSMUST00000067841.7	insulin receptor substrate 4
2549	Cdh20	ENSMUST00000062528.8	cadherin 20
2550	Slc5a1	ENSMUST00000011178.2	solute carrier family 5 (sodium/glucose cotransporter), member 1
2551	Arhgap44	ENSMUST00000047463.9	Rho GTPase activating protein 44

2552	Tub	ENSMUST00000033341.6	tubby candidate gene
2553	Vsig10l	ENSMUST00000107977.1	ZV-set and immunoglobulin domain containing 10 like
2554	Zfyve28	ENSMUST00000094868.4	zinc finger, FYVE domain containing 28
2555	Ppp3r2	ENSMUST00000029991.2	protein phosphatase 3, regulatory subunit B, alpha isoform (calcineurin B, type II)
2556	Aktip	ENSMUST00000120349.2	thymoma viral proto-oncogene 1 interacting protein
2557	Dcx	ENSMUST00000033642.4	doublecortin
2558	Pip4k2b	ENSMUST00000018691.8	phosphatidylinositol-5-phosphate 4-kinase, type II, beta
2559	Pfkfb4	ENSMUST00000051873.9	6-phosphofructo-2-kinase/fructose-2,6-biphosphatase 4
2560	Nrip1	ENSMUST00000121927.2	nuclear receptor interacting protein 1
2561	Nynrin	ENSMUST00000168479.1	NYN domain and retroviral integrase containing
2562	Kcnq2	ENSMUST00000149964.3	potassium voltage-gated channel, subfamily Q, member 2
2563	Cd84	ENSMUST00000135386.1	CD84 antigen
2564	Rap2c	ENSMUST00000053593.7	RAP2C, member of RAS oncogene family
2565	C2cd2l	ENSMUST00000065080.8	C2 calcium-dependent domain containing 2-like
2566	Gpr155	ENSMUST00000076463.6	G protein-coupled receptor 155
2567	Timeless	ENSMUST00000055539.5	timeless circadian clock 1
2568	Prkab2	ENSMUST00000045743.7	protein kinase, AMP-activated, beta 2 non-catalytic subunit
2569	Gbp7	ENSMUST00000171263.2	guanylate binding protein 7
2570	Plagl1	ENSMUST00000121646.2	pleiomorphic adenoma gene-like 1
2571	Limd1	ENSMUST00000026269.2	LIM domains containing 1
2572	Icosl	ENSMUST00000105393.1	icos ligand
2573	Ccdc36	ENSMUST00000076592.2	coiled-coil domain containing 36
2574	Gpr101	ENSMUST00000057645.5	G protein-coupled receptor 101
2575	Baz1a	ENSMUST00000038926.7	bromodomain adjacent to zinc finger domain 1A
2576	1200014J11Rik	ENSMUST00000021135.3	RIKEN cDNA 1200014J11 gene
2577	Map3k3	ENSMUST00000002044.9	mitogen-activated protein kinase kinase kinase 3
2578	Mex3b	ENSMUST00000082237.5	mex3 homolog B (C. elegans)
2579	Kctd20	ENSMUST00000118762.2	potassium channel tetramerisation domain containing 20
2580	Map3k13	ENSMUST00000042065.6	mitogen-activated protein kinase kinase kinase 13
2581	Hs3st3a1	ENSMUST00000058652.5	heparan sulfate (glucosamine) 3-O-sulfotransferase 3A1
2582	Kcng1	ENSMUST00000109191.1	potassium voltage-gated channel, subfamily G, member 1
2583	Bahd1	ENSMUST00000036578.6	bromo adjacent homology domain containing 1
2584	Ino80	ENSMUST00000049920.8	INO80 homolog (S. cerevisiae)
2585	Rell1	ENSMUST00000154169.1	RELT-like 1
2586	Dlg5	ENSMUST00000073687.7	discs, large homolog 5 (Drosophila)
2587	Setbp1	ENSMUST00000025430.9	SET binding protein 1
2588	AU018091	ENSMUST00000171749.1	expressed sequence AU018091
2589	Limk1	ENSMUST00000015137.4	LIM-domain containing, protein kinase
2590	Zbtb8b	ENSMUST00000106046.2	zinc finger and BTB domain containing 8b
2591	L3mbtl1	ENSMUST00000035751.6	l(3)mbt-like (Drosophila)
2592	Tmprss11f	ENSMUST00000116553.3	transmembrane protease, serine 11f
2593	Ube2g2	ENSMUST00000174510.2	ubiquitin-conjugating enzyme E2G 2

2594	Chrna2	ENSMUST00000022620.9	cholinergic receptor, nicotinic, alpha polypeptide 2 (neuronal)
2595	Foxn1	ENSMUST00000108294.1	forkhead box N1
2596	Trim41	ENSMUST00000047145.8	tripartite motif-containing 41
2597	Tmprss11b	ENSMUST00000038448.6	transmembrane protease, serine 11B
2598	Bhlhe23	ENSMUST00000108878.1	basic helix-loop-helix family, member e23
2599	Gm9747	ENSMUST00000023262.5	predicted gene 9747
2600	Fut4	ENSMUST00000061498.5	fucosyltransferase 4
2601	Cacna1i	ENSMUST00000160424.2	calcium channel, voltage-dependent, alpha 1I subunit
2602	Dzip3	ENSMUST00000121869.2	DAZ interacting protein 3, zinc finger
2603	Chmp6	ENSMUST00000026434.7	charged multivesicular body protein 6
2604	Dock3	ENSMUST00000044532.5	dedicator of cyto-kinesis 3
2605	Fbxl16	ENSMUST00000045692.7	F-box and leucine-rich repeat protein 16
2606	Usp54	ENSMUST00000022356.5	ubiquitin specific peptidase 54
2607	Zfp445	ENSMUST00000056467.7	zinc finger protein 445
2608	Ckap5	ENSMUST00000111338.4	cytoskeleton associated protein 5
2609	Naip1	ENSMUST00000022142.5	NLR family, apoptosis inhibitory protein 1
2610	Kif1b	ENSMUST00000030806.5	kinesin family member 1B
2611	Vwc2l	ENSMUST00000053922.6	von Willebrand factor C domain-containing protein 2-like
2612	Zfp568	ENSMUST00000146074.2	zinc finger protein 568
2613	Fam135b	ENSMUST00000022953.8	family with sequence similarity 135, member B
2614	Rap1gap	ENSMUST00000097837.5	Rap1 GTPase-activating protein
2615	Adcy7	ENSMUST00000169037.2	adenylate cyclase 7
2616	Ak4	ENSMUST00000102780.2	adenylate kinase 4
2617	Acad12	ENSMUST00000041252.7	acyl-Coenzyme A dehydrogenase family, member 12
2618	Mum1l1	ENSMUST00000113045.3	melanoma associated antigen (mutated) 1-like 1
2619	Gm15800	ENSMUST00000119892.3	predicted gene 15800
2620	Zfhx2	ENSMUST00000036328.8	zinc finger homeobox 2
2621	Brd4	ENSMUST00000114475.3	bromodomain containing 4
2622	Fignl2	ENSMUST00000178140.1	fidgetin-like 2
2623	Hey1	ENSMUST00000042412.3	hairy/enhancer-of-split related with YRPW motif 1
2624	Impact	ENSMUST00000025290.5	impact, RWD domain protein
2625	Aph1b	ENSMUST00000034934.9	anterior pharynx defective 1b homolog (C. elegans)
2626	Acer3	ENSMUST00000033020.8	alkaline ceramidase 3
2627	Shroom4	ENSMUST00000089520.2	shroom family member 4
2628	Pde3b	ENSMUST00000032909.8	phosphodiesterase 3B, cGMP-inhibited
2629	Kpna3	ENSMUST00000022496.7	karyopherin (importin) alpha 3
2630	Lgr6	ENSMUST00000044828.8	leucine-rich repeat-containing G protein-coupled receptor 6
2631	Utrn	ENSMUST00000076817.4	utrophin
2632	Btbd11	ENSMUST00000105307.2	BTB (POZ) domain containing 11
2633	Atp7b	ENSMUST00000006742.5	ATPase, Cu++ transporting, beta polypeptide
2634	Cflar	ENSMUST00000114313.2	CASP8 and FADD-like apoptosis regulator
2635	Camk1d	ENSMUST00000044009.8	calcium/calmodulin-dependent protein kinase ID
2636	Adcy9	ENSMUST00000117801.2	adenylate cyclase 9
2637	Ankfy1	ENSMUST00000155998.1	ankyrin repeat and FYVE domain containing 1
2638	Nt5c1a	ENSMUST00000068262.5	5'-nucleotidase, cytosolic IA
2639	Rpp14	ENSMUST00000180369.1	ribonuclease P 14 subunit
2640	Whsc1l1	ENSMUST00000142395.2	Wolf-Hirschhorn syndrome candidate 1-like 1 (human)
2641	Lepre1	ENSMUST00000030393.7	leprecan 1

2642	Znfx1	ENSMUST00000048988.8	zinc finger, NFX1-type containing 1
2643	Gm16515	ENSMUST00000019075.3	predicted gene, Gm16515
2644	Rock2	ENSMUST00000020904.6	Rho-associated coiled-coil containing protein kinase 2
2645	Samhd1	ENSMUST00000057725.4	SAM domain and HD domain, 1
2646	Ankle2	ENSMUST00000031474.8	ankyrin repeat and LEM domain containing 2
2647	Zfp516	ENSMUST00000171238.2	zinc finger protein 516
2648	Armc1	ENSMUST00000029125.8	armadillo repeat containing 1
2649	Naa40	ENSMUST00000025675.9	N(alpha)-acetyltransferase 40, NatD catalytic subunit, homolog (S. cerevisiae)
2650	Rapgef5	ENSMUST00000109691.2	Rap guanine nucleotide exchange factor (GEF) 5
2651	Abl2	ENSMUST00000166172.3	v-abl Abelson murine leukemia viral oncogene 2 (arg, Abelson-related gene)
2652	Fam46a	ENSMUST00000034802.9	family with sequence similarity 46, member A
2653	Nfam1	ENSMUST00000109503.4	Nfat activating molecule with ITAM motif 1
2654	Asap3	ENSMUST00000047526.7	ArfGAP with SH3 domain, ankyrin repeat and PH domain 3
2655	Atp2b4	ENSMUST00000048953.8	ATPase, Ca++ transporting, plasma membrane 4
2656	Crispld2	ENSMUST00000132583.2	cysteine-rich secretory protein LCCL domain containing 2
2657	Frmd3	ENSMUST00000084474.5	FERM domain containing 3
2658	Rtn3	ENSMUST00000065304.6	reticulon 3
2659	Pdxdc1	ENSMUST00000115804.3	pyridoxal-dependent decarboxylase domain containing 1
2660	Rab22a	ENSMUST00000029024.4	RAB22A, member RAS oncogene family
2661	Arfip2	ENSMUST00000131446.2	ADP-ribosylation factor interacting protein 2
2662	Gm14139	ENSMUST00000109926.2	predicted gene 14139
2663	Adamts12	ENSMUST00000061318.7	a disintegrin-like and metallopeptidase (reprolysin type) with thrombospondin type 1 motif, 12
2664	Arhgap29	ENSMUST00000037958.8	Rho GTPase activating protein 29
2665	Zfp457	ENSMUST00000049705.7	zinc finger protein 457
2666	Ikzf5	ENSMUST00000046306.9	IKAROS family zinc finger 5
2667	Prune2	ENSMUST00000087689.4	prune homolog 2 (Drosophila)
2668	Aebp2	ENSMUST00000032359.9	AE binding protein 2
2669	Cers6	ENSMUST00000028426.3	ceramide synthase 6
2670	Crebrf	ENSMUST00000062519.8	CREB3 regulatory factor
2671	Exoc8	ENSMUST00000098312.2	exocyst complex component 8
2672	Nr3c1	ENSMUST00000115567.2	nuclear receptor subfamily 3, group C, member 1
2673	Mapk4	ENSMUST00000159162.1	mitogen-activated protein kinase 4
2674	Angel2	ENSMUST00000027947.7	angel homolog 2 (Drosophila)
2675	Usp13	ENSMUST00000072312.6	ubiquitin specific peptidase 13 (isopeptidase T-3)
2676	Klf10	ENSMUST00000074043.5	Kruppel-like factor 10
2677	Siah1a	ENSMUST00000045296.4	seven in absentia 1A
2678	Camkv	ENSMUST00000035700.8	CaM kinase-like vesicle-associated
2679	Clic6	ENSMUST00000023670.3	chloride intracellular channel 6
2680	Htr1f	ENSMUST00000063076.4	5-hydroxytryptamine (serotonin) receptor 1F
2681	Zfp59	ENSMUST00000108331.2	zinc finger protein 59
2682	Sh3rf3	ENSMUST00000153031.1	SH3 domain containing ring finger 3
2683	Srsf1	ENSMUST00000079866.5	serine/arginine-rich splicing factor 1
2684	Tspan3	ENSMUST00000034876.8	tetraspanin 3
2685	Trim11	ENSMUST00000108809.2	tripartite motif-containing 11
2686	Maoa	ENSMUST00000026013.5	monoamine oxidase A
2687	Fosl2	ENSMUST00000031017.9	fos-like antigen 2

2688	Kif3b	ENSMUST00000028977.6	kinesin family member 3B
2689	Marveld3	ENSMUST00000001722.8	MARVEL (membrane-associating) domain containing 3
2690	Tenm4	ENSMUST00000107162.2	teneurin transmembrane protein 4
2691	Rnf149	ENSMUST00000062525.5	ring finger protein 149
2692	Akap11	ENSMUST00000123853.2	A kinase (PRKA) anchor protein 11
2693	Acbd5	ENSMUST00000028121.8	acyl-Coenzyme A binding domain containing 5
2694	Nat8l	ENSMUST00000056355.8	N-acetyltransferase 8-like
2695	Pcm1	ENSMUST00000045218.7	pericentriolar material 1
2696	Fzd5	ENSMUST00000063982.6	frizzled homolog 5 (Drosophila)
2697	Adam22	ENSMUST00000088761.5	a disintegrin and metallopeptidase domain 22
2698	Fbxo21	ENSMUST00000035579.6	F-box protein 21
2699	Mul1	ENSMUST00000044058.5	mitochondrial ubiquitin ligase activator of NFKB 1
2700	Mars2	ENSMUST00000061334.9	methionine-tRNA synthetase 2 (mitochondrial)
2701	Gas2l3	ENSMUST00000099374.3	growth arrest-specific 2 like 3
2702	Cbfa2t2	ENSMUST00000109725.2	core-binding factor, runt domain, alpha subunit 2, translocated to, 2 (human)
2703	Pdk4	ENSMUST00000019721.4	pyruvate dehydrogenase kinase, isoenzyme 4
2704	Lancl3	ENSMUST00000069763.2	LanC lantibiotic synthetase component C-like 3 (bacterial)
2705	Arhgef37	ENSMUST00000171629.1	Rho guanine nucleotide exchange factor (GEF) 37
2706	P2rx7	ENSMUST00000100737.4	purinergic receptor P2X, ligand-gated ion channel, 7
2707	Ptchd1	ENSMUST00000038665.5	patched domain containing 1
2708	Serinc5	ENSMUST00000049488.7	serine incorporator 5
2709	Rab39	ENSMUST00000068449.3	RAB39, member RAS oncogene family
2710	B4galt6	ENSMUST00000070080.5	UDP-Gal:betaGlcNAc beta 1,4-galactosyltransferase, polypeptide 6
2711	Slc25a43	ENSMUST00000047655.6	solute carrier family 25, member 43
2712	U2surp	ENSMUST00000079659.6	U2 snRNP-associated SURP domain containing
2713	Hivep3	ENSMUST00000106307.3	human immunodeficiency virus type I enhancer binding protein 3
2714	Hsf4	ENSMUST00000172525.2	heat shock transcription factor 4
2715	Gm8923	ENSMUST00000143490.1	predicted gene 8923
2716	Fli1	ENSMUST00000016231.8	Friend leukemia integration 1
2717	Timp2	ENSMUST00000017610.4	tissue inhibitor of metalloproteinase 2
2718	Ttyh1	ENSMUST00000129423.2	tweety homolog 1 (Drosophila)
2719	Morc2a	ENSMUST00000096441.3	microrchidia 2A
2720	Pqlc2	ENSMUST00000053862.6	PQ loop repeat containing 2
2721	Braf	ENSMUST00000002487.9	Braf transforming gene
2722	Dnm1l	ENSMUST00000023477.8	dynamin 1-like
2723	Zfp706	ENSMUST00000078976.7	zinc finger protein 706
2724	Pex7	ENSMUST00000020182.9	peroxisomal biogenesis factor 7
2725	Fyco1	ENSMUST00000167595.3	FYVE and coiled-coil domain containing 1
2726	Grin2a	ENSMUST00000115835.2	glutamate receptor, ionotropic, NMDA2A (epsilon 1)
2727	Desi2	ENSMUST00000027783.8	desumoylating isopeptidase 2
2728	Dlgap1	ENSMUST00000133983.2	discs, large (Drosophila) homolog-associated protein 1
2729	Zbtb25	ENSMUST00000176102.2	zinc finger and BTB domain containing 25
2730	Glg1	ENSMUST00000169020.2	golgi apparatus protein 1
2731	Pramef8	ENSMUST00000059790.5	PRAME family member 8
2732	Faxc	ENSMUST00000029908.7	failed axon connections homolog (Drosophila)

2733	Slc25a21	ENSMUST00000044634.6	solute carrier family 25 (mitochondrial oxodicarboxylate carrier), member 21
2734	Fam53b	ENSMUST00000097998.3	family with sequence similarity 53, member B
2735	Ints12	ENSMUST00000029650.8	integrator complex subunit 12
2736	Mettl10	ENSMUST00000033257.9	methyltransferase like 10
2737	Tmeff2	ENSMUST00000081851.3	transmembrane protein with EGF-like and two follistatin-like domains 2
2738	Clcn1	ENSMUST00000031894.7	chloride channel 1
2739	Arl6ip6	ENSMUST00000028336.6	ADP-ribosylation factor-like 6 interacting protein 6
2740	Nipa2	ENSMUST00000119041.2	non imprinted in Prader-Willi/Angelman syndrome 2 homolog (human)
2741	1810013L24Rik	ENSMUST00000023150.5	RIKEN cDNA 1810013L24 gene
2742	Layn	ENSMUST00000098782.3	layilin
2743	Cdc42se1	ENSMUST00000053872.6	CDC42 small effector 1
2744	Btbd16	ENSMUST00000048453.5	BTB (POZ) domain containing 16
2745	Tspan9	ENSMUST00000112173.2	tetraspanin 9
2746	Rabep1	ENSMUST00000100928.5	rabaptin, RAB GTPase binding effector protein 1
2747	Kctd16	ENSMUST00000091927.4	potassium channel tetramerisation domain containing 16
2748	Nkiras1	ENSMUST00000132374.2	NFKB inhibitor interacting Ras-like protein 1
2749	Hist1h3f	ENSMUST00000075558.3	histone cluster 1, H3f
2750	Ado	ENSMUST00000075686.4	2-aminoethanethiol (cysteamine) dioxygenase
2751	Itgb6	ENSMUST00000059888.9	integrin beta 6
2752	Wbp1l	ENSMUST00000138302.3	WW domain binding protein 1 like
2753	Ppp1r16b	ENSMUST00000052927.5	protein phosphatase 1, regulatory (inhibitor) subunit 16B
2754	Prrg4	ENSMUST00000028593.5	proline rich Gla (G-carboxyglutamic acid) 4 (transmembrane)
2755	Inpp1	ENSMUST00000027271.3	inositol polyphosphate-1-phosphatase
2756	Rbm4	ENSMUST00000178615.2	RNA binding motif protein 4
2757	Pms1	ENSMUST00000027267.8	postmeiotic segregation increased 1 (S. cerevisiae)
2758	Prr7	ENSMUST00000046533.7	proline rich 7 (synaptic)
2759	Fgd6	ENSMUST00000020208.4	FYVE, RhoGEF and PH domain containing 6
2760	Paip2b	ENSMUST00000058383.8	poly(A) binding protein interacting protein 2B
2761	Mynn	ENSMUST00000047502.7	myoneurin
2762	Ets1	ENSMUST00000034534.7	E26 avian leukemia oncogene 1, 5' domain
2763	Zcchc9	ENSMUST00000022121.7	zinc finger, CCHC domain containing 9
2764	Tomm70a	ENSMUST00000166897.1	translocase of outer mitochondrial membrane 70 homolog A (yeast)
2765	mt-Nd5	ENSMUST00000082418.1	mitochondrially encoded NADH dehydrogenase 5
2766	Rdh1	ENSMUST00000073639.5	retinol dehydrogenase 1 (all trans)
2767	BC048403	ENSMUST00000136432.1	cDNA sequence BC048403
2768	Zfp618	ENSMUST00000107415.2	zinc finger protein 618
2769	Bard1	ENSMUST00000027393.7	BRCA1 associated RING domain 1
2770	Lrrc58	ENSMUST00000078717.6	leucine rich repeat containing 58
2771	Prpf39	ENSMUST00000120580.2	PRP39 pre-mRNA processing factor 39 homolog (yeast)
2772	Adcyap1r1	ENSMUST00000070756.6	adenylate cyclase activating polypeptide 1 receptor 1
2773	Trim44	ENSMUST00000102573.2	tripartite motif-containing 44
2774	Serbp1	ENSMUST00000042990.4	serpine1 mRNA binding protein 1
2775	Tmem56	ENSMUST00000128909.2	transmembrane protein 56
2776	Mr1	ENSMUST00000027744.9	major histocompatibility complex, class I-related

2777	Rpl18a	ENSMUST00000054220.8	ribosomal protein L18A
2778	Slc6a2	ENSMUST00000072939.6	solute carrier family 6 (neurotransmitter transporter, noradrenalin), member 2
2779	Emc7	ENSMUST00000069747.5	ER membrane protein complex subunit 7
2780	Fzd1	ENSMUST00000054294.5	frizzled homolog 1 (Drosophila)
2781	Kcnb2	ENSMUST00000170146.2	potassium voltage gated channel, Shab-related subfamily, member 2
2782	Mtnr1a	ENSMUST00000067984.7	melatonin receptor 1A
2783	Rab18	ENSMUST00000097680.5	RAB18, member RAS oncogene family
2784	Zfp830	ENSMUST00000056677.7	zinc finger protein 830
2785	Eda2r	ENSMUST00000037353.4	ectodysplasin A2 receptor
2786	Selp	ENSMUST00000162746.1	selectin, platelet
2787	Ndnf	ENSMUST00000054351.4	neuron-derived neurotrophic factor
2788	Gria2	ENSMUST00000107745.2	glutamate receptor, ionotropic, AMPA2 (alpha 2)
2789	Synpr	ENSMUST00000070323.6	synaptoporin
2790	4833403I15Rik	ENSMUST00000025358.2	RIKEN cDNA 4833403I15 gene
2791	Terf2	ENSMUST00000116425.2	telomeric repeat binding factor 2
2792	Bcl2l11	ENSMUST00000110341.3	BCL2-like 11 (apoptosis facilitator)
2793	Paxbp1	ENSMUST00000118522.2	PAX3 and PAX7 binding protein 1
2794	Mc5r	ENSMUST00000172148.1	melanocortin 5 receptor
2795	Sorl1	ENSMUST00000060989.8	sortilin-related receptor, LDLR class A repeats-containing
2796	Pex5l	ENSMUST00000108225.4	peroxisomal biogenesis factor 5-like
2797	Gipc3	ENSMUST00000045102.5	GIPC PDZ domain containing family, member 3
2798	Mtrf1l	ENSMUST00000019908.8	mitochondrial translational release factor 1-like
2799	Exoc3	ENSMUST00000035934.5	exocyst complex component 3
2800	Wdr82	ENSMUST00000020490.6	WD repeat domain containing 82
2801	Uhmk1	ENSMUST00000123399.1	U2AF homology motif (UHM) kinase 1
2802	Mthfr	ENSMUST00000069604.9	5,10-methylenetetrahydrofolate reductase
2803	Mcidas	ENSMUST00000092089.4	multiciliate differentiation and DNA synthesis associated cell cycle protein
2804	Ndc1	ENSMUST00000139560.2	NDC1 transmembrane nucleoporin
2805	Ube2h	ENSMUST00000102993.4	ubiquitin-conjugating enzyme E2H
2806	Nova1	ENSMUST00000021438.6	neuro-oncological ventral antigen 1
2807	Serpina3i	ENSMUST00000109958.2	serine (or cysteine) peptidase inhibitor, clade A, member 3I
2808	Klhl42	ENSMUST00000036003.7	kelch-like 42
2809	Mycbp	ENSMUST00000030400.8	c-myc binding protein
2810	Papolb	ENSMUST00000099400.2	poly (A) polymerase beta (testis specific)
2811	Stk35	ENSMUST00000166282.2	serine/threonine kinase 35
2812	Ncam1	ENSMUST00000114476.2	neural cell adhesion molecule 1
2813	Smu1	ENSMUST00000030117.4	smu-1 suppressor of mec-8 and unc-52 homolog (C. elegans)
2814	Zfp345	ENSMUST00000109914.1	zinc finger protein 345
2815	Itpkb	ENSMUST00000070181.6	inositol 1,4,5-trisphosphate 3-kinase B
2816	Zfp74	ENSMUST00000108205.2	zinc finger protein 74
2817	Nudt3	ENSMUST00000025050.6	nudix (nucleotide diphosphate linked moiety X)-type motif 3
2818	Nap1l1	ENSMUST00000171797.1	nucleosome assembly protein 1-like 1
2819	Rprd1a	ENSMUST00000046206.3	regulation of nuclear pre-mRNA domain containing 1A
2820	Rccd1	ENSMUST00000121882.2	RCC1 domain containing 1

2821	Lrrc7	ENSMUST00000106044.1	leucine rich repeat containing 7
2822	Irgq	ENSMUST00000049020.7	immunity-related GTPase family, Q
2823	Bbox1	ENSMUST00000046233.3	butyrobetaine (gamma), 2-oxoglutarate dioxygenase 1 (gamma-butyrobetaine hydroxylase)
2824	Slit3	ENSMUST00000069837.3	slit homolog 3 (Drosophila)
2825	B3galt1	ENSMUST00000112346.2	UDP-Gal:betaGlcNAc beta 1,3-galactosyltransferase, polypeptide 1
2826	Rab30	ENSMUST00000032879.8	RAB30, member RAS oncogene family
2827	Nlrc3	ENSMUST00000180200.2	NLR family, CARD domain containing 3
2828	Zfp870	ENSMUST00000178401.1	zinc finger protein 870
2829	Agbl2	ENSMUST00000170320.2	ATP/GTP binding protein-like 2
2830	Tprkb	ENSMUST00000113753.2	Tp53rk binding protein
2831	Ccdc157	ENSMUST00000093381.5	coiled-coil domain containing 157
2832	Dhx40	ENSMUST00000018569.8	DEAH (Asp-Glu-Ala-His) box polypeptide 40
2833	Cbx5	ENSMUST00000118152.2	chromobox 5
2834	Ptgdr2	ENSMUST00000037261.3	prostaglandin D2 receptor 2
2835	Snrpd1	ENSMUST00000002551.3	small nuclear ribonucleoprotein D1
2836	Zfp933	ENSMUST00000105718.2	zinc finger protein 933
2837	Thnsl1	ENSMUST00000054591.4	threonine synthase-like 1 (bacterial)
2838	Alg9	ENSMUST00000034561.5	asparagine-linked glycosylation 9 (alpha 1,2 mannosyltransferase)
2839	6720489N17Rik	ENSMUST00000091563.4	RIKEN cDNA 6720489N17 gene
2840	Atp13a3	ENSMUST00000061350.7	ATPase type 13A3
2841	Eefsec	ENSMUST00000165242.1	eukaryotic elongation factor, selenocysteine-tRNA-specific
2842	St6galnac2	ENSMUST00000079545.5	ST6 (alpha-N-acetyl-neuraminyl-2,3-beta-galactosyl-1,3)-N-acetylgalactosaminide alpha-2,6-sialyltransferase 2
2843	Nrg4	ENSMUST00000164721.2	neuregulin 4
2844	Meis2	ENSMUST00000110907.2	Meis homeobox 2
2845	Fam217b	ENSMUST00000094251.5	family with sequence similarity 217, member B
2846	Hnrnpa3	ENSMUST00000111962.2	heterogeneous nuclear ribonucleoprotein A3
2847	Bcl11b	ENSMUST00000066060.5	B cell leukemia/lymphoma 11B
2848	Vat1l	ENSMUST00000049509.6	vesicle amine transport protein 1 homolog-like (T. californica)
2849	Aatk	ENSMUST00000064307.4	apoptosis-associated tyrosine kinase
2850	Slc8a3	ENSMUST00000085238.7	solute carrier family 8 (sodium/calcium exchanger), member 3
2851	Smarcal1	ENSMUST00000047615.9	SWI/SNF related matrix associated, actin dependent regulator of chromatin, subfamily a-like 1
2852	Gpatch8	ENSMUST00000143842.1	G patch domain containing 8
2853	Dynlt1c	ENSMUST00000092966.4	dynein light chain Tctex-type 1C
2854	Smyd3	ENSMUST00000128302.2	SET and MYND domain containing 3
2855	Slc9a2	ENSMUST00000027231.7	solute carrier family 9 (sodium/hydrogen exchanger), member 2
2856	Smarce1	ENSMUST00000103133.3	SWI/SNF related, matrix associated, actin dependent regulator of chromatin, subfamily e, member 1
2857	B3galnt2	ENSMUST00000099747.3	UDP-GalNAc:betaGlcNAc beta 1,3-galactosaminyltransferase, polypeptide 2
2858	Trim66	ENSMUST00000106739.2	tripartite motif-containing 66
2859	Gm14124	ENSMUST00000109922.1	predicted gene 14124
2860	Frs2	ENSMUST00000020381.3	fibroblast growth factor receptor substrate 2

2861	Tomm20	ENSMUST00000179857.1	translocase of outer mitochondrial membrane 20 homolog (yeast)
2862	Elf4	ENSMUST00000114958.2	E74-like factor 4 (ets domain transcription factor)
2863	Cyp2d22	ENSMUST00000023083.7	cytochrome P450, family 2, subfamily d, polypeptide 22
2864	Gprin3	ENSMUST00000051065.4	GPRIN family member 3
2865	Emc1	ENSMUST00000042096.8	ER membrane protein complex subunit 1
2866	Csnk2a1	ENSMUST00000099224.4	casein kinase 2, alpha 1 polypeptide
2867	Pogz	ENSMUST00000107270.3	pogo transposable element with ZNF domain
2868	Lcmt2	ENSMUST00000110674.3	leucine carboxyl methyltransferase 2
2869	Trub1	ENSMUST00000026073.7	TruB pseudouridine (psi) synthase homolog 1 (E. coli)
2870	Tbcel	ENSMUST00000066148.6	tubulin folding cofactor E-like
2871	9130008F23Rik	ENSMUST00000068258.2	RIKEN cDNA 9130008F23 gene
2872	Tram2	ENSMUST00000037998.5	translocating chain-associating membrane protein 2
2873	Kctd21	ENSMUST00000054107.5	potassium channel tetramerisation domain containing 21
2874	Gm13151	ENSMUST00000148762.3	predicted gene 13151
2875	Lmbr1	ENSMUST00000055195.5	limb region 1
2876	Zfp704	ENSMUST00000041124.7	zinc finger protein 704
2877	Tbpl1	ENSMUST00000127698.2	TATA box binding protein-like 1
2878	BC049715	ENSMUST00000052702.6	cDNA sequence BC049715
2879	Med25	ENSMUST00000003049.6	mediator complex subunit 25
2880	Acot11	ENSMUST00000102762.4	acyl-CoA thioesterase 11
2881	Tmf1	ENSMUST00000095664.3	TATA element modulatory factor 1
2882	Pgap1	ENSMUST00000097739.4	post-GPI attachment to proteins 1
2883	Sgcb	ENSMUST00000081170.7	sarcoglycan, beta (dystrophin-associated glycoprotein)
2884	Prkag3	ENSMUST00000188073.1	protein kinase, AMP-activated, gamma 3 non-catatlytic subunit
2885	Egr3	ENSMUST00000035908.1	early growth response 3
2886	Ctbs	ENSMUST00000061937.7	chitobiase, di-N-acetyl-
2887	Kpna4	ENSMUST00000029353.3	karyopherin (importin) alpha 4
2888	Zfp111	ENSMUST00000086006.6	zinc finger protein 111
2889	Gm9938	ENSMUST00000067077.1	predicted gene 9938
2890	Dcaf8	ENSMUST00000074144.5	DDB1 and CUL4 associated factor 8
2891	Rasa2	ENSMUST00000034984.7	RAS p21 protein activator 2
2892	Fbxw2	ENSMUST00000028220.4	F-box and WD-40 domain protein 2
2893	Rps6kb1	ENSMUST00000154617.2	ribosomal protein S6 kinase, polypeptide 1
2894	Bbip1	ENSMUST00000135402.2	BBSome interacting protein 1
2895	Zdhhc15	ENSMUST00000042070.5	zinc finger, DHHC domain containing 15
2896	Abhd6	ENSMUST00000166497.2	abhydrolase domain containing 6
2897	Colgalt2	ENSMUST00000044311.8	collagen beta(1-O)galactosyltransferase 2
2898	Egln1	ENSMUST00000034469.5	egl-9 family hypoxia-inducible factor 1
2899	Pik3r5	ENSMUST00000021283.7	phosphoinositide-3-kinase, regulatory subunit 5, p101
2900	Pip4k2a	ENSMUST00000006912.6	phosphatidylinositol-5-phosphate 4-kinase, type II, alpha
2901	Arl5a	ENSMUST00000036541.7	ADP-ribosylation factor-like 5A
2902	Lztfl1	ENSMUST00000026274.8	leucine zipper transcription factor-like 1
2903	Lrrcc1	ENSMUST00000169079.2	leucine rich repeat and coiled-coil domain containing 1

2904	Nucks1	ENSMUST00000062264.7	nuclear casein kinase and cyclin-dependent kinase substrate 1
2905	Foxc1	ENSMUST00000062292.3	forkhead box C1
2906	Prdm5	ENSMUST00000172638.1	PR domain containing 5
2907	Mypn	ENSMUST00000095580.2	myopalladin
2908	Flrt2	ENSMUST00000057324.3	fibronectin leucine rich transmembrane protein 2
2909	Sirpa	ENSMUST00000103203.2	signal-regulatory protein alpha
2910	Limk2	ENSMUST00000110029.3	LIM motif-containing protein kinase 2
2911	Trpm2	ENSMUST00000105401.3	transient receptor potential cation channel, subfamily M, member 2
2912	Trim24	ENSMUST00000031859.8	tripartite motif-containing 24
2913	Itch	ENSMUST00000029126.9	itchy, E3 ubiquitin protein ligase
2914	Cacna1d	ENSMUST00000112249.2	calcium channel, voltage-dependent, L type, alpha 1D subunit
2915	Tet1	ENSMUST00000050826.8	tet methylcytosine dioxygenase 1
2916	Aph1c	ENSMUST00000169282.2	anterior pharynx defective 1c homolog (C. elegans)
2917	Dnajc18	ENSMUST00000025208.5	DnaJ (Hsp40) homolog, subfamily C, member 18
2918	Kdm2b	ENSMUST00000031435.8	lysine (K)-specific demethylase 2B
2919	Zfp958	ENSMUST00000073201.5	zinc finger protein 958
2920	Plp1	ENSMUST00000033800.7	proteolipid protein (myelin) 1
2921	Arnt2	ENSMUST00000085077.3	aryl hydrocarbon receptor nuclear translocator 2
2922	Sdc4	ENSMUST00000017153.3	syndecan 4
2923	Nusap1	ENSMUST00000028771.7	nucleolar and spindle associated protein 1
2924	Tubal3	ENSMUST00000021639.6	tubulin, alpha-like 3
2925	Gne	ENSMUST00000030201.8	glucosamine (UDP-N-acetyl)-2-epimerase/N-acetylmannosamine kinase
2926	Slc5a9	ENSMUST00000102721.2	solute carrier family 5 (sodium/glucose cotransporter), member 9
2927	Cln5	ENSMUST00000022721.6	ceroid-lipofuscinosis, neuronal 5
2928	Eif1ax	ENSMUST00000087143.6	eukaryotic translation initiation factor 1A, X-linked
2929	Tex12	ENSMUST00000034568.5	testis expressed gene 12
2930	Btg1	ENSMUST00000038377.7	B cell translocation gene 1, anti-proliferative
2931	Bysl	ENSMUST00000024783.8	bystin-like
2932	Map3k9	ENSMUST00000035987.7	mitogen-activated protein kinase kinase kinase 9
2933	Adam12	ENSMUST00000067680.5	a disintegrin and metallopeptidase domain 12 (meltrin alpha)
2934	Slc24a4	ENSMUST00000079020.5	solute carrier family 24 (sodium/potassium/calcium exchanger), member 4
2935	5830473C10Rik	ENSMUST00000094615.4	RIKEN cDNA 5830473C10 gene
2936	Gpsm2	ENSMUST00000029482.10	G-protein signalling modulator 2 (AGS3-like, C. elegans)
2937	Bdh1	ENSMUST00000115227.4	3-hydroxybutyrate dehydrogenase, type 1
2938	Tsr2	ENSMUST00000112683.3	TSR2 20S rRNA accumulation
2939	Cry2	ENSMUST00000090559.6	cryptochrome 2 (photolyase-like)
2940	AW554918	ENSMUST00000165400.2	expressed sequence AW554918
2941	Jmjd6	ENSMUST00000047616.4	jumonji domain containing 6
2942	Psmf1	ENSMUST00000042452.5	proteasome (prosome, macropain) inhibitor subunit 1
2943	Arhgap32	ENSMUST00000174641.2	Rho GTPase activating protein 32
2944	Megf6	ENSMUST00000030897.9	multiple EGF-like-domains 6
2945	Nacc2	ENSMUST00000114159.3	nucleus accumbens associated 2, BEN and BTB (POZ) domain containing
2946	Fut8	ENSMUST00000062804.7	fucosyltransferase 8

2947	Pi15	ENSMUST00000088476.3	peptidase inhibitor 15
2948	Mmd2	ENSMUST00000037048.7	monocyte to macrophage differentiation-associated 2
2949	Zbtb37	ENSMUST00000171748.2	zinc finger and BTB domain containing 37
2950	Eapp	ENSMUST00000161592.2	E2F-associated phosphoprotein
2951	Vps37a	ENSMUST00000098817.2	vacuolar protein sorting 37A (yeast)
2952	Plxna2	ENSMUST00000027952.6	plexin A2
2953	Zfp408	ENSMUST00000099714.4	zinc finger protein 408
2954	AA415398	ENSMUST00000079611.7	expressed sequence AA415398
2955	Zbtb1	ENSMUST00000042779.3	zinc finger and BTB domain containing 1
2956	Zfp738	ENSMUST00000137496.3	zinc finger protein 738
2957	Prss23	ENSMUST00000041761.5	protease, serine 23
2958	Utp15	ENSMUST00000040972.2	UTP15, U3 small nucleolar ribonucleoprotein, homolog (yeast)
2959	Klra2	ENSMUST00000032306.9	killer cell lectin-like receptor, subfamily A, member 2
2960	Hilpda	ENSMUST00000054445.8	hypoxia inducible lipid droplet associated
2961	Etv3	ENSMUST00000119109.2	ets variant 3
2962	Ido2	ENSMUST00000121992.1	indoleamine 2,3-dioxygenase 2
2963	Dpysl4	ENSMUST00000026551.9	dihydropyrimidinase-like 4
2964	Ccdc116	ENSMUST00000023452.8	coiled-coil domain containing 116
2965	Gmfb	ENSMUST00000111817.2	glia maturation factor, beta
2966	Dcp1b	ENSMUST00000112777.3	DCP1 decapping enzyme homolog B (S. cerevisiae)
2967	Pde5a	ENSMUST00000066728.5	phosphodiesterase 5A, cGMP-specific
2968	Ccdc130	ENSMUST00000005120.6	coiled-coil domain containing 130
2969	Lrat	ENSMUST00000029632.6	lecithin-retinol acyltransferase (phosphatidylcholine-retinol-O-acyltransferase)
2970	Dffa	ENSMUST00000030816.3	DNA fragmentation factor, alpha subunit
2971	Bcmo1	ENSMUST00000176860.1	beta-carotene 15,15'-monooxygenase
2972	Slc22a15	ENSMUST00000190824.1	solute carrier family 22 (organic anion/cation transporter), member 15
2973	Etf1	ENSMUST00000025218.7	eukaryotic translation termination factor 1
2974	S1pr1	ENSMUST00000055676.2	sphingosine-1-phosphate receptor 1
2975	Fndc8	ENSMUST00000018988.5	fibronectin type III domain containing 8
2976	Ccdc89	ENSMUST00000061391.7	coiled-coil domain containing 89
2977	Lrrc57	ENSMUST00000102496.2	leucine rich repeat containing 57
2978	Bbx	ENSMUST00000138166.2	bobby sox homolog (Drosophila)
2979	Eno4	ENSMUST00000054280.6	enolase 4
2980	Flcn	ENSMUST00000102697.4	folliculin
2981	Nxt1	ENSMUST00000109961.2	NTF2-related export protein 1
2982	Trip11	ENSMUST00000021605.8	thyroid hormone receptor interactor 11
2983	Rasgrf2	ENSMUST00000099326.4	RAS protein-specific guanine nucleotide-releasing factor 2
2984	Atf7ip	ENSMUST00000032335.7	activating transcription factor 7 interacting protein
2985	Mbnl1	ENSMUST00000099087.2	muscleblind-like 1 (Drosophila)
2986	Btn1a1	ENSMUST00000041674.8	butyrophilin, subfamily 1, member A1
2987	Zfp191	ENSMUST00000066497.6	zinc finger protein 191
2988	Ing3	ENSMUST00000031680.4	inhibitor of growth family, member 3
2989	Sco1	ENSMUST00000092996.4	SCO cytochrome oxidase deficient homolog 1 (yeast)
2990	Mau2	ENSMUST00000050561.7	MAU2 chromatid cohesion factor homolog (C. elegans)
2991	Fam49a	ENSMUST00000069066.7	family with sequence similarity 49, member A
2992	Gda	ENSMUST00000087600.4	guanine deaminase
2993	2410131K14Rik	ENSMUST00000049138.7	RIKEN cDNA 2410131K14 gene

2994	Prdm16	ENSMUST00000070313.8	PR domain containing 16
2995	Dspp	ENSMUST00000112771.1	dentin sialophosphoprotein
2996	Fam84b	ENSMUST00000100635.4	family with sequence similarity 84, member B
2997	Naif1	ENSMUST00000048431.2	nuclear apoptosis inducing factor 1
2998	Mia3	ENSMUST00000069922.6	melanoma inhibitory activity 3
2999	Dsg1a	ENSMUST00000077146.3	desmoglein 1 alpha
3000	Fibcd1	ENSMUST00000028188.7	fibrinogen C domain containing 1
3001	Spa17	ENSMUST00000002013.9	sperm autoantigenic protein 17
3002	Dmtf1	ENSMUST00000071921.7	cyclin D binding myb-like transcription factor 1
3003	Slc24a2	ENSMUST00000107157.3	solute carrier family 24 (sodium/potassium/calcium exchanger), member 2
3004	Arrdc4	ENSMUST00000048068.8	arrestin domain containing 4
3005	Ldlr	ENSMUST00000034713.7	low density lipoprotein receptor
3006	Rgag4	ENSMUST00000113631.1	retrotransposon gag domain containing 4
3007	Alx4	ENSMUST00000111254.1	aristaless-like homeobox 4
3008	Fancf	ENSMUST00000169357.1	Fanconi anemia, complementation group F
3009	Cldn18	ENSMUST00000136429.2	claudin 18
3010	Scn2b	ENSMUST00000170998.2	sodium channel, voltage-gated, type II, beta
3011	Rap1gap2	ENSMUST00000102521.2	RAP1 GTPase activating protein 2
3012	Dlx1	ENSMUST00000037119.3	distal-less homeobox 1
3013	Oas2	ENSMUST00000081491.7	2'-5' oligoadenylate synthetase 2
3014	Dmbx1	ENSMUST00000064806.7	diencephalon/mesencephalon homeobox 1
3015	Atf1	ENSMUST00000023769.5	activating transcription factor 1
3016	Mast4	ENSMUST00000167058.2	microtubule associated serine/threonine kinase family member 4
3017	Stxbp5	ENSMUST00000038213.8	syntaxin binding protein 5 (tomosyn)
3018	Ldlrad2	ENSMUST00000178923.1	low density lipoprotein receptor A domain containing 2
3019	Grin3a	ENSMUST00000093859.5	glutamate receptor ionotropic, NMDA3A
3020	Fndc7	ENSMUST00000053065.5	fibronectin type III domain containing 7
3021	Rnf166	ENSMUST00000014614.3	ring finger protein 166
3022	Slc28a3	ENSMUST00000022036.8	solute carrier family 28 (sodium-coupled nucleoside transporter), member 3
3023	Unc80	ENSMUST00000061620.10	unc-80 homolog (C. elegans)
3024	Grip1	ENSMUST00000041962.9	glutamate receptor interacting protein 1
3025	Pkdrej	ENSMUST00000064370.4	polycystic kidney disease (polycystin) and REJ (sperm receptor for egg jelly homolog, sea urchin)
3026	Micu1	ENSMUST00000165563.2	mitochondrial calcium uptake 1
3027	Psg29	ENSMUST00000075934.5	pregnancy-specific glycoprotein 29
3028	Tenm1	ENSMUST00000115059.2	teneurin transmembrane protein 1
3029	Tspan7	ENSMUST00000115526.1	tetraspanin 7
3030	Dpp10	ENSMUST00000112606.2	dipeptidylpeptidase 10
3031	Sept3	ENSMUST00000023095.7	septin 3
3032	Msrb3	ENSMUST00000092143.6	methionine sulfoxide reductase B3
3033	Celf3	ENSMUST00000029784.5	CUGBP, Elav-like family member 3
3034	Kcnv2	ENSMUST00000056708.3	potassium channel, subfamily V, member 2
3035	Cr2	ENSMUST00000082321.3	complement receptor 2
3036	Gm8113	ENSMUST00000160298.1	predicted gene 8113
3037	Epb4.1l4b	ENSMUST00000030142.3	erythrocyte protein band 4.1-like 4b
3038	Uspl1	ENSMUST00000117878.2	ubiquitin specific peptidase like 1
3039	Col8a2	ENSMUST00000070132.6	collagen, type VIII, alpha 2

3040	Slc6a3	ENSMUST00000022100.6	solute carrier family 6 (neurotransmitter transporter, dopamine), member 3
3041	Nt5dc3	ENSMUST00000099396.2	5'-nucleotidase domain containing 3
3042	Dhh	ENSMUST00000023737.4	desert hedgehog
3043	Gm5595	ENSMUST00000107992.1	predicted gene 5595
3044	Ube2w	ENSMUST00000117146.2	ubiquitin-conjugating enzyme E2W (putative)
3045	Asxl3	ENSMUST00000097655.3	additional sex combs like 3 (Drosophila)
3046	Cox10	ENSMUST00000049091.8	cytochrome c oxidase assembly protein 10
3047	Ccdc158	ENSMUST00000060930.4	coiled-coil domain containing 158
3048	Ccdc88c	ENSMUST00000085096.4	coiled-coil domain containing 88C
3049	Col27a1	ENSMUST00000036300.7	collagen, type XXVII, alpha 1
3050	Palld	ENSMUST00000121785.2	palladin, cytoskeletal associated protein
3051	Zfp652	ENSMUST00000107717.2	zinc finger protein 652
3052	Fndc3b	ENSMUST00000046157.4	fibronectin type III domain containing 3B
3053	Zfx	ENSMUST00000088102.6	zinc finger protein X-linked
3054	Cyb5d1	ENSMUST00000108660.2	cytochrome b5 domain containing 1
3055	Chd2	ENSMUST00000169922.3	chromodomain helicase DNA binding protein 2
3056	Ckap4	ENSMUST00000053871.3	cytoskeleton-associated protein 4
3057	Trmt2a	ENSMUST00000140206.2	TRM2 tRNA methyltransferase 2A
3058	Vsig10	ENSMUST00000111967.2	V-set and immunoglobulin domain containing 10
3059	H2-T24	ENSMUST00000174063.1	histocompatibility 2, T region locus 24
3060	Syne3	ENSMUST00000067005.4	spectrin repeat containing, nuclear envelope family member 3
3061	Prrg3	ENSMUST00000048790.6	proline rich Gla (G-carboxyglutamic acid) 3 (transmembrane)
3062	Chd9	ENSMUST00000109614.3	chromodomain helicase DNA binding protein 9
3063	Uprt	ENSMUST00000087867.5	uracil phosphoribosyltransferase (FUR1) homolog (S. cerevisiae)
3064	Casr	ENSMUST00000114847.3	calcium-sensing receptor
3065	Dram1	ENSMUST00000020249.1	DNA-damage regulated autophagy modulator 1
3066	Ipo9	ENSMUST00000041023.8	importin 9
3067	Rnf165	ENSMUST00000026494.8	ring finger protein 165
3068	Ercc3	ENSMUST00000025241.6	excision repair cross-complementing rodent repair deficiency, complementation group 3
3069	Tlr6	ENSMUST00000062315.4	toll-like receptor 6
3070	Rc3h1	ENSMUST00000161609.2	RING CCCH (C3H) domains 1
3071	Herc3	ENSMUST00000041401.8	hect domain and RLD 3
3072	Tapt1	ENSMUST00000055128.7	transmembrane anterior posterior transformation 1
3073	Cmtm4	ENSMUST00000179802.1	CKLF-like MARVEL transmembrane domain containing 4
3074	Mtmr7	ENSMUST00000048898.11	myotubularin related protein 7
3075	Spata6	ENSMUST00000084354.3	spermatogenesis associated 6
3076	Gpd1l	ENSMUST00000146623.2	glycerol-3-phosphate dehydrogenase 1-like
3077	Trps1	ENSMUST00000165201.3	trichorhinophalangeal syndrome I (human)
3078	Il22ra1	ENSMUST00000102546.3	interleukin 22 receptor, alpha 1
3079	Lgi2	ENSMUST00000039750.5	leucine-rich repeat LGI family, member 2
3080	Rai1	ENSMUST00000102688.1	retinoic acid induced 1
3081	Phf20l1	ENSMUST00000048188.8	PHD finger protein 20-like 1
3082	Irf9	ENSMUST00000134863.2	interferon regulatory factor 9
3083	1810032O08Rik	ENSMUST00000144049.2	RIKEN cDNA 1810032O08 gene
3084	Prcp	ENSMUST00000076052.6	prolylcarboxypeptidase (angiotensinase C)
3085	2810459M11Rik	ENSMUST00000165824.2	RIKEN cDNA 2810459M11 gene

3086	Hipk1	ENSMUST00000118317.2	homeodomain interacting protein kinase 1
3087	Nbeal1	ENSMUST00000160834.2	neurobeachin like 1
3088	Tsc1	ENSMUST00000028155.6	tuberous sclerosis 1
3089	Ppp2r5d	ENSMUST00000002839.8	protein phosphatase 2, regulatory subunit B', delta
3090	D630002J18Rik	ENSMUST00000181854.1	RIKEN cDNA D630002J18 gene
3091	Dlgap4	ENSMUST00000169464.3	discs, large homolog-associated protein 4 (Drosophila)
3092	Abcc2	ENSMUST00000026208.5	ATP-binding cassette, sub-family C (CFTR/MRP), member 2
3093	Eif4ebp2	ENSMUST00000020288.8	eukaryotic translation initiation factor 4E binding protein 2
3094	Rab11fip1	ENSMUST00000033878.7	RAB11 family interacting protein 1 (class I)
3095	Klf16	ENSMUST00000038558.8	Kruppel-like factor 16
3096	Foxk1	ENSMUST00000072837.5	forkhead box K1
3097	Synj1	ENSMUST00000170853.2	synaptojanin 1
3098	Trim23	ENSMUST00000069187.6	tripartite motif-containing 23
3099	Sepsecs	ENSMUST00000031069.7	Sep (O-phosphoserine) tRNA:Sec (selenocysteine) tRNA synthase
3100	Erlin1	ENSMUST00000112028.4	ER lipid raft associated 1
3101	Ostn	ENSMUST00000066852.8	osteocrin
3102	Adrbk2	ENSMUST00000065167.4	adrenergic receptor kinase, beta 2
3103	Zfp36l2	ENSMUST00000060366.6	zinc finger protein 36, C3H type-like 2
3104	Sntb2	ENSMUST00000047425.3	syntrophin, basic 2
3105	Ifnar1	ENSMUST00000023689.5	interferon (alpha and beta) receptor 1
3106	Tomt	ENSMUST00000106969.2	transmembrane O-methyltransferase
3107	A530054K11Rik	ENSMUST00000012314.8	RIKEN cDNA A530054K11 gene
3108	Atmin	ENSMUST00000109099.3	ATM interactor
3109	BC007180	ENSMUST00000189969.1	cDNA sequence BC007180
3110	Sema3a	ENSMUST00000030714.7	sema domain, immunoglobulin domain (Ig), short basic domain, secreted, (semaphorin) 3A
3111	Stard10	ENSMUST00000163799.3	START domain containing 10
3112	Ankrd45	ENSMUST00000111608.2	ankyrin repeat domain 45
3113	Unc13a	ENSMUST00000030170.9	unc-13 homolog A (C. elegans)
3114	Prex1	ENSMUST00000036719.6	phosphatidylinositol-3,4,5-trisphosphate-dependent Rac exchange factor 1
3115	Sult6b1	ENSMUST00000169544.2	sulfotransferase family, cytosolic, 6B, member 1
3116	AY358078	ENSMUST00000053821.4	cDNA sequence AY358078
3117	Ubtd2	ENSMUST00000051053.4	ubiquitin domain containing 2
3118	Tor4a	ENSMUST00000081869.6	torsin family 4, member A
3119	Rab11fip5	ENSMUST00000060837.7	RAB11 family interacting protein 5 (class I)
3120	Zfp354b	ENSMUST00000109124.4	zinc finger protein 354B
3121	Sh3glb1	ENSMUST00000163279.1	SH3-domain GRB2-like B1 (endophilin)
3122	Clec16a	ENSMUST00000115823.1	C-type lectin domain family 16, member A
3123	Nktr	ENSMUST00000035112.7	natural killer tumor recognition sequence
3124	Gprasp2	ENSMUST00000173804.2	G protein-coupled receptor associated sorting protein 2
3125	Skil	ENSMUST00000118470.2	SKI-like
3126	Slc37a3	ENSMUST00000090243.4	solute carrier family 37 (glycerol-3-phosphate transporter), member 3
3127	Klf9	ENSMUST00000036884.1	Kruppel-like factor 9
3128	Cpt1c	ENSMUST00000063761.7	carnitine palmitoyltransferase 1c

3129	Relb	ENSMUST00000094762.4	avian reticuloendotheliosis viral (v-rel) oncogene related B
3130	Ptprn2	ENSMUST00000070733.8	protein tyrosine phosphatase, receptor type, N polypeptide 2
3131	Cobll1	ENSMUST00000112429.3	Cobl-like 1
3132	Slc30a1	ENSMUST00000044954.6	solute carrier family 30 (zinc transporter), member 1
3133	Pcsk9	ENSMUST00000049507.5	proprotein convertase subtilisin/kexin type 9
3134	Med12l	ENSMUST00000040325.8	mediator complex subunit 12-like
3135	Rad54l2	ENSMUST00000046502.6	RAD54 like 2 (S. cerevisiae)
3136	Chrm2	ENSMUST00000172278.2	cholinergic receptor, muscarinic 2, cardiac
3137	Crem	ENSMUST00000151311.2	cAMP responsive element modulator
3138	Lbh	ENSMUST00000024857.7	limb-bud and heart
3139	Stxbp5l	ENSMUST00000114787.2	syntaxin binding protein 5-like
3140	Gucy1a3	ENSMUST00000048976.6	guanylate cyclase 1, soluble, alpha 3
3141	Sema5a	ENSMUST00000067458.6	sema domain, seven thrombospondin repeats (type 1 and type 1-like), transmembrane domain (TM) and short cytoplasmic domain, (semaphorin) 5A
3142	Smad2	ENSMUST00000168423.2	SMAD family member 2
3143	Aif1	ENSMUST00000172693.2	allograft inflammatory factor 1
3144	Kctd10	ENSMUST00000102581.5	potassium channel tetramerisation domain containing 10
3145	Rgs7bp	ENSMUST00000063551.5	regulator of G-protein signalling 7 binding protein
3146	Ppp2r5a	ENSMUST00000067976.3	protein phosphatase 2, regulatory subunit B', alpha
3147	Poc5	ENSMUST00000099295.4	POC5 centriolar protein homolog (Chlamydomonas)
3148	Lrtm1	ENSMUST00000055662.2	leucine-rich repeats and transmembrane domains 1
3149	Rpp38	ENSMUST00000062672.6	ribonuclease P/MRP 38 subunit
3150	Mtap7d3	ENSMUST00000114766.2	MAP7 domain containing 3
3151	Rgp1	ENSMUST00000117140.2	RGP1 retrograde golgi transport homolog (S. cerevisiae)
3152	Tspan32	ENSMUST00000075172.6	tetraspanin 32
3153	Sycp2	ENSMUST00000081134.4	synaptonemal complex protein 2
3154	Snx12	ENSMUST00000077876.3	sorting nexin 12
3155	Fam45a	ENSMUST00000119633.2	family with sequence similarity 45, member A
3156	Fat3	ENSMUST00000082170.5	FAT tumor suppressor homolog 3 (Drosophila)
3157	Frem2	ENSMUST00000091137.4	Fras1 related extracellular matrix protein 2
3158	Rbm12	ENSMUST00000059647.6	RNA binding motif protein 12
3159	Casp6	ENSMUST00000029626.8	caspase 6
3160	Mill2	ENSMUST00000072386.5	MHC I like leukocyte 2
3161	Ms4a4b	ENSMUST00000035258.8	membrane-spanning 4-domains, subfamily A, member 4B
3162	Tmem136	ENSMUST00000061833.4	transmembrane protein 136
3163	Fabp5	ENSMUST00000029046.8	fatty acid binding protein 5, epidermal
3164	Zfp42	ENSMUST00000082120.3	zinc finger protein 42
3165	Mphosph9	ENSMUST00000031344.7	M-phase phosphoprotein 9
3166	Smchd1	ENSMUST00000127430.1	SMC hinge domain containing 1
3167	Ppp2r5e	ENSMUST00000021447.7	protein phosphatase 2, regulatory subunit B', epsilon
3168	Golga3	ENSMUST00000112512.2	golgi autoantigen, golgin subfamily a, 3
3169	A430033K04Rik	ENSMUST00000069862.8	RIKEN cDNA A430033K04 gene
3170	Mrpl39	ENSMUST00000116584.1	mitochondrial ribosomal protein L39
3171	Dcaf17	ENSMUST00000154704.2	DDB1 and CUL4 associated factor 17
3172	Nfia	ENSMUST00000092532.7	nuclear factor I/A
3173	Nmd3	ENSMUST00000029358.9	NMD3 homolog (S. cerevisiae)

3174	Ptpn6	ENSMUST00000004377.9	protein tyrosine phosphatase, non-receptor type 6
3175	Myo6	ENSMUST00000035889.9	myosin VI
3176	Neu1	ENSMUST00000007253.5	neuraminidase 1
3177	Glra2	ENSMUST00000058787.8	glycine receptor, alpha 2 subunit
3178	Trub2	ENSMUST00000048044.6	TruB pseudouridine (psi) synthase homolog 2 (E. coli)
3179	Abcb8	ENSMUST00000115077.2	ATP-binding cassette, sub-family B (MDR/TAP), member 8
3180	Trit1	ENSMUST00000102649.3	tRNA isopentenyltransferase 1
3181	F2rl3	ENSMUST00000058099.8	coagulation factor II (thrombin) receptor-like 3
3182	Cldn19	ENSMUST00000094823.3	claudin 19
3183	Tmc2	ENSMUST00000077988.2	transmembrane channel-like gene family 2
3184	Pdcd5	ENSMUST00000118501.2	programmed cell death 5
3185	Cdk7	ENSMUST00000091299.6	cyclin-dependent kinase 7
3186	H2-M5	ENSMUST00000169189.2	histocompatibility 2, M region locus 5
3187	Itpr2	ENSMUST00000053273.9	inositol 1,4,5-triphosphate receptor 2
3188	Zbtb41	ENSMUST00000039867.7	zinc finger and BTB domain containing 41 homolog
3189	Tbc1d25	ENSMUST00000039892.3	TBC1 domain family, member 25
3190	Ggt5	ENSMUST00000072217.3	gamma-glutamyltransferase 5
3191	Slit1	ENSMUST00000169141.2	slit homolog 1 (Drosophila)
3192	Kcnh5	ENSMUST00000042299.2	potassium voltage-gated channel, subfamily H (eag-related), member 5
3193	Rho	ENSMUST00000032471.6	rhodopsin
3194	Col11a1	ENSMUST00000092155.6	collagen, type XI, alpha 1
3195	Bves	ENSMUST00000095715.3	blood vessel epicardial substance
3196	Itga1	ENSMUST00000061673.7	integrin alpha 1
3197	Rhbdl3	ENSMUST00000017836.7	rhomboid, veinlet-like 3 (Drosophila)
3198	Rhof	ENSMUST00000186469.1	ras homolog gene family, member f
3199	Sel1l	ENSMUST00000021347.6	sel-1 suppressor of lin-12-like (C. elegans)
3200	Tlr4	ENSMUST00000048096.6	toll-like receptor 4
3201	Cntf	ENSMUST00000112933.1	ciliary neurotrophic factor
3202	Rcc2	ENSMUST00000071169.3	regulator of chromosome condensation 2
3203	Mrps5	ENSMUST00000028852.7	mitochondrial ribosomal protein S5
3204	Abt1	ENSMUST00000041782.2	activator of basal transcription 1
3205	Ogfrl1	ENSMUST00000027343.5	opioid growth factor receptor-like 1
3206	Lrguk	ENSMUST00000070189.4	leucine-rich repeats and guanylate kinase domain containing
3207	Pcdhb12	ENSMUST00000055495.5	protocadherin beta 12
3208	Acyp2	ENSMUST00000074613.3	acylphosphatase 2, muscle type
3209	Tpte	ENSMUST00000077194.1	transmembrane phosphatase with tensin homology
3210	Cox11	ENSMUST00000020851.9	cytochrome c oxidase assembly protein 11
3211	3632451O06Rik	ENSMUST00000036972.7	RIKEN cDNA 3632451O06 gene
3212	Gdpgp1	ENSMUST00000062915.7	GDP-D-glucose phosphorylase 1
3213	Cfl2	ENSMUST00000078124.7	cofilin 2, muscle
3214	Ptgfr	ENSMUST00000029670.5	prostaglandin F receptor
3215	Rimbp2	ENSMUST00000111346.1	RIMS binding protein 2
3216	Arl4a	ENSMUST00000146905.1	ADP-ribosylation factor-like 4A
3217	Ccnt2	ENSMUST00000112570.1	cyclin T2
3218	Zmym4	ENSMUST00000106108.3	zinc finger, MYM-type 4
3219	Odf3l1	ENSMUST00000055036.6	outer dense fiber of sperm tails 3-like 1
3220	Xylt1	ENSMUST00000032892.5	xylosyltransferase 1
3221	Ago4	ENSMUST00000084289.4	argonaute RISC catalytic subunit 4

3222	Gpr182	ENSMUST00000079692.5	G protein-coupled receptor 182
3223	Rassf4	ENSMUST00000035842.4	Ras association (RalGDS/AF-6) domain family member 4
3224	Trim61	ENSMUST00000124790.2	tripartite motif-containing 61
3225	Dctn1	ENSMUST00000113913.2	dynactin 1
3226	Pla2g2e	ENSMUST00000030531.8	phospholipase A2, group IIE
3227	Aff4	ENSMUST00000060945.6	AF4/FMR2 family, member 4
3228	Arpc1b	ENSMUST00000085679.7	actin related protein 2/3 complex, subunit 1B
3229	Hk2	ENSMUST00000000642.5	hexokinase 2
3230	P2ry4	ENSMUST00000053373.1	pyrimidinergic receptor P2Y, G-protein coupled, 4
3231	Tlk1	ENSMUST00000038584.8	tousled-like kinase 1
3232	Wdr33	ENSMUST00000025264.6	WD repeat domain 33
3233	Efcab9	ENSMUST00000054327.2	EF-hand calcium binding domain 9
3234	Thap11	ENSMUST00000040445.7	THAP domain containing 11
3235	Nepn	ENSMUST00000067085.4	nephrocan
3236	Zfyve9	ENSMUST00000106657.2	zinc finger, FYVE domain containing 9
3237	Map3k7cl	ENSMUST00000026700.7	Map3k7 C-terminal like
3238	Igf2bp1	ENSMUST00000013559.2	insulin-like growth factor 2 mRNA binding protein 1
3239	Nup160	ENSMUST00000057481.6	nucleoporin 160
3240	Dapk3	ENSMUST00000178422.2	death-associated protein kinase 3
3241	Amotl1	ENSMUST00000160770.2	angiomotin-like 1
3242	Mcmbp	ENSMUST00000057557.8	MCM (minichromosome maintenance deficient) binding protein
3243	Decr1	ENSMUST00000029877.8	2,4-dienoyl CoA reductase 1, mitochondrial
3244	Btc	ENSMUST00000121044.2	betacellulin, epidermal growth factor family member
3245	Rpp14	ENSMUST00000023924.3	ribonuclease P 14 subunit
3246	Fbxl17	ENSMUST00000024761.7	F-box and leucine-rich repeat protein 17
3247	Rufy2	ENSMUST00000119567.2	RUN and FYVE domain-containing 2
3248	Fcho1	ENSMUST00000093444.7	FCH domain only 1
3249	Lhx9	ENSMUST00000019374.8	LIM homeobox protein 9
3250	Eif2d	ENSMUST00000068805.8	eukaryotic translation initiation factor 2D
3251	Slc17a4	ENSMUST00000021769.9	solute carrier family 17 (sodium phosphate), member 4
3252	Fam92a	ENSMUST00000108285.3	family with sequence similarity 92, member A
3253	Clec14a	ENSMUST00000062254.2	C-type lectin domain family 14, member a
3254	Sap30l	ENSMUST00000020826.5	SAP30-like
3255	Hhip	ENSMUST00000079038.2	Hedgehog-interacting protein
3256	Proser2	ENSMUST00000054254.6	proline and serine rich 2
3257	Zfp184	ENSMUST00000102978.2	zinc finger protein 184 (Kruppel-like)
3258	Msantd1	ENSMUST00000050535.1	Myb/SANT-like DNA-binding domain containing 1
3259	Osbpl6	ENSMUST00000111929.2	oxysterol binding protein-like 6
3260	Harbi1	ENSMUST00000090608.5	harbinger transposase derived 1
3261	Homer1	ENSMUST00000079086.6	homer homolog 1 (Drosophila)
3262	Dnajc15	ENSMUST00000022590.3	DnaJ (Hsp40) homolog, subfamily C, member 15
3263	Plxna4	ENSMUST00000115096.3	plexin A4
3264	Slco2a1	ENSMUST00000035148.7	solute carrier organic anion transporter family, member 2a1
3265	4933411K16Rik	ENSMUST00000164518.3	RIKEN cDNA 4933411K16 gene
3266	Lss	ENSMUST00000048678.6	lanosterol synthase
3267	Edem1	ENSMUST00000089162.3	ER degradation enhancer, mannosidase alpha-like 1
3268	Steap3	ENSMUST00000112641.2	STEAP family member 3

3269	Utp6	ENSMUST00000043152.5	UTP6, small subunit (SSU) processome component, homolog (yeast)
3270	Hspa1b	ENSMUST00000172753.1	heat shock protein 1B
3271	Dnajb11	ENSMUST00000178320.1	DnaJ (Hsp40) homolog, subfamily B, member 11
3272	Cyp7b1	ENSMUST00000035625.6	cytochrome P450, family 7, subfamily b, polypeptide 1
3273	Mbl1	ENSMUST00000047095.2	mannose-binding lectin (protein A) 1
3274	Raly	ENSMUST00000116389.3	hnRNP-associated with lethal yellow
3275	Dnajc9	ENSMUST00000022345.6	DnaJ (Hsp40) homolog, subfamily C, member 9
3276	Sntg1	ENSMUST00000140295.2	syntrophin, gamma 1
3277	Evi2a	ENSMUST00000103236.3	ecotropic viral integration site 2a
3278	St3gal1	ENSMUST00000092640.5	ST3 beta-galactoside alpha-2,3-sialyltransferase 1
3279	Zdhhc6	ENSMUST00000076891.5	zinc finger, DHHC domain containing 6
3280	Elp4	ENSMUST00000122965.2	elongation protein 4 homolog (S. cerevisiae)
3281	Bnip2	ENSMUST00000034754.6	BCL2/adenovirus E1B interacting protein 2
3282	Cmtr1	ENSMUST00000024816.7	cap methyltransferase 1
3283	Larp4	ENSMUST00000057632.9	La ribonucleoprotein domain family, member 4
3284	Dyrk1b	ENSMUST00000085901.7	dual-specificity tyrosine-(Y)-phosphorylation regulated kinase 1b
3285	Hist1h2ac	ENSMUST00000171127.2	histone cluster 1, H2ac
3286	Mocs2	ENSMUST00000183407.1	molybdenum cofactor synthesis 2
3287	Plin3	ENSMUST00000019726.6	perilipin 3
3288	Pafah2	ENSMUST00000105870.2	platelet-activating factor acetylhydrolase 2
3289	Blnk	ENSMUST00000117695.2	B cell linker
3290	Tbk1	ENSMUST00000020316.2	TANK-binding kinase 1
3291	Pdp2	ENSMUST00000059588.6	pyruvate dehyrogenase phosphatase catalytic subunit 2
3292	Tlr12	ENSMUST00000074829.2	toll-like receptor 12
3293	Urm1	ENSMUST00000091142.3	ubiquitin related modifier 1 homolog (S. cerevisiae)
3294	Csrp2	ENSMUST00000020403.5	cysteine and glycine-rich protein 2
3295	Zfp101	ENSMUST00000167107.3	zinc finger protein 101
3296	Nsun7	ENSMUST00000065530.7	NOL1/NOP2/Sun domain family, member 7
3297	Chrnb2	ENSMUST00000029562.3	cholinergic receptor, nicotinic, beta polypeptide 2 (neuronal)
3298	Zc3h6	ENSMUST00000110320.3	zinc finger CCCH type containing 6
3299	Tmem246	ENSMUST00000042750.2	transmembrane protein 246
3300	Ros1	ENSMUST00000020045.4	Ros1 proto-oncogene
3301	Sass6	ENSMUST00000029571.8	spindle assembly 6 homolog (C. elegans)
3302	Slc6a20b	ENSMUST00000026273.8	solute carrier family 6 (neurotransmitter transporter), member 20B
3303	Srsf10	ENSMUST00000126641.1	serine/arginine-rich splicing factor 10
3304	Lrrc71	ENSMUST00000023846.5	leucine rich repeat containing 71
3305	Ctso	ENSMUST00000029649.2	cathepsin O
3306	Adamts1	ENSMUST00000023610.9	a disintegrin-like and metallopeptidase (reprolysin type) with thrombospondin type 1 motif, 1
3307	Bag4	ENSMUST00000038498.8	BCL2-associated athanogene 4
3308	Rsrc1	ENSMUST00000161726.1	arginine/serine-rich coiled-coil 1
3309	Cyp2d10	ENSMUST00000072776.3	cytochrome P450, family 2, subfamily d, polypeptide 10
3310	Myo5b	ENSMUST00000074157.7	myosin VB
3311	Zdhhc7	ENSMUST00000034280.7	zinc finger, DHHC domain containing 7
3312	Dnase2b	ENSMUST00000029836.4	deoxyribonuclease II beta

3313	Aplf	ENSMUST00000065997.2	aprataxin and PNKP like factor
3314	Fbxo7	ENSMUST00000130320.2	F-box protein 7
3315	Tmem126a	ENSMUST00000032844.5	transmembrane protein 126A
3316	Tmem57	ENSMUST00000030628.9	transmembrane protein 57
3317	Nubpl	ENSMUST00000040090.10	nucleotide binding protein-like
3318	Tomm6	ENSMUST00000113301.1	translocase of outer mitochondrial membrane 6 homolog (yeast)
3319	Srpx2	ENSMUST00000113304.1	sushi-repeat-containing protein, X-linked 2
3320	Add2	ENSMUST00000032069.5	adducin 2 (beta)
3321	Inpp5f	ENSMUST00000043138.7	inositol polyphosphate-5-phosphatase F
3322	Arel1	ENSMUST00000043169.8	apoptosis resistant E3 ubiquitin protein ligase 1
3323	Tfam	ENSMUST00000092430.5	transcription factor A, mitochondrial
3324	Klf13	ENSMUST00000063694.8	Kruppel-like factor 13
3325	F930015N05Rik	ENSMUST00000177999.1	RIKEN cDNA F930015N05 gene
3326	Tpp1	ENSMUST00000033184.4	tripeptidyl peptidase I
3327	Mlkl	ENSMUST00000056157.8	mixed lineage kinase domain-like
3328	Mapk1	ENSMUST00000069107.8	mitogen-activated protein kinase 1
3329	Pcdhb7	ENSMUST00000053037.3	protocadherin beta 7
3330	Col6a2	ENSMUST00000001181.7	collagen, type VI, alpha 2
3331	Retsat	ENSMUST00000070597.7	retinol saturase (all trans retinol 13,14 reductase)
3332	Rab29	ENSMUST00000027693.7	RAB29, member RAS oncogene family
3333	Cacna1c	ENSMUST00000186889.1	calcium channel, voltage-dependent, L type, alpha 1C subunit
3334	Ccr4	ENSMUST00000054414.3	chemokine (C-C motif) receptor 4
3335	Arfgef2	ENSMUST00000099078.4	ADP-ribosylation factor guanine nucleotide-exchange factor 2 (brefeldin A-inhibited)
3336	Bhlha9	ENSMUST00000056184.1	basic helix-loop-helix family, member a9
3337	Pon3	ENSMUST00000031773.3	paraoxonase 3
3338	Smim20	ENSMUST00000121042.2	small integral membrane protein 20
3339	Rnf11	ENSMUST00000030284.4	ring finger protein 11
3340	Gclm	ENSMUST00000029769.8	glutamate-cysteine ligase, modifier subunit
3341	Itgb3bp	ENSMUST00000146258.1	integrin beta 3 binding protein (beta3-endonexin)
3342	Rps20	ENSMUST00000138502.1	ribosomal protein S20
3343	5430435G22Rik	ENSMUST00000064679.3	RIKEN cDNA 5430435G22 gene
3344	Tomm22	ENSMUST00000023062.3	translocase of outer mitochondrial membrane 22 homolog (yeast)
3345	Mphosph6	ENSMUST00000034303.1	M phase phosphoprotein 6
3346	Ddx19b	ENSMUST00000040241.9	DEAD (Asp-Glu-Ala-Asp) box polypeptide 19b
3347	Trp53bp1	ENSMUST00000110647.2	transformation related protein 53 binding protein 1
3348	Mrpl17	ENSMUST00000124482.1	mitochondrial ribosomal protein L17
3349	Asb3	ENSMUST00000020551.7	ankyrin repeat and SOCS box-containing 3
3350	Snx10	ENSMUST00000049152.9	sorting nexin 10
3351	Tlr8	ENSMUST00000112170.1	toll-like receptor 8
3352	Xkr8	ENSMUST00000045550.4	X Kell blood group precursor related family member 8 homolog
3353	Agpat5	ENSMUST00000149565.2	1-acylglycerol-3-phosphate O-acyltransferase 5 (lysophosphatidic acid acyltransferase, epsilon)
3354	Myl1	ENSMUST00000120415.2	myosin, light polypeptide 1
3355	Slc10a4	ENSMUST00000031127.7	solute carrier family 10 (sodium/bile acid cotransporter family), member 4
3356	Zfp874b	ENSMUST00000019572.7	zinc finger protein 874b
3357	Fopnl	ENSMUST00000120707.1	Fgfr1op N-terminal like

3358	Cul4a	ENSMUST00000016680.8	cullin 4A
3359	Tyms	ENSMUST00000026846.6	thymidylate synthase
3360	Gin1	ENSMUST00000027571.7	gypsy retrotransposon integrase 1
3361	Pcolce2	ENSMUST00000015498.8	procollagen C-endopeptidase enhancer 2
3362	Gjc1	ENSMUST00000068933.6	gap junction protein, gamma 1
3363	Tpo	ENSMUST00000021005.9	thyroid peroxidase
3364	Gpr171	ENSMUST00000085040.4	G protein-coupled receptor 171
3365	Psma2	ENSMUST00000170836.2	proteasome (prosome, macropain) subunit, alpha type 2
3366	Taf8	ENSMUST00000067103.2	TAF8 RNA polymerase II, TATA box binding protein (TBP)-associated factorq
3367	Cdca2	ENSMUST00000150006.3	cell division cycle associated 2
3368	Polr3g	ENSMUST00000048993.6	polymerase (RNA) III (DNA directed) polypeptide G
3369	Hoxb9	ENSMUST00000000010.8	homeobox B9
3370	Pcyox1	ENSMUST00000032065.9	prenylcysteine oxidase 1
3371	1700011H14Rik	ENSMUST00000022398.8	RIKEN cDNA 1700011H14 gene
3372	Hsd17b1	ENSMUST00000019445.5	hydroxysteroid (17-beta) dehydrogenase 1
3373	Otud1	ENSMUST00000052168.5	OTU domain containing 1
3374	Plin4	ENSMUST00000190703.1	perilipin 4
3375	Rps4l	ENSMUST00000071745.3	ribosomal protein S4-like
3376	Ngly1	ENSMUST00000022310.6	N-glycanase 1
3377	Sh2d1b2	ENSMUST00000179976.1	SH2 domain protein 1B2
3378	Adrb2	ENSMUST00000053640.3	adrenergic receptor, beta 2
3379	Vrk2	ENSMUST00000109504.2	vaccinia related kinase 2
3380	Mrpl3	ENSMUST00000035177.9	mitochondrial ribosomal protein L3
3381	Cytip	ENSMUST00000028175.6	cytohesin 1 interacting protein
3382	Bet1	ENSMUST00000049166.4	blocked early in transport 1 homolog (S. cerevisiae)
3383	Prkd2	ENSMUST00000168093.3	protein kinase D2
3384	Gstt1	ENSMUST00000001713.4	glutathione S-transferase, theta 1
3385	Zfp873	ENSMUST00000105313.1	zinc finger protein 873
3386	St3gal2	ENSMUST00000034197.4	ST3 beta-galactoside alpha-2,3-sialyltransferase 2
3387	Xdh	ENSMUST00000024866.4	xanthine dehydrogenase
3388	Krcc1	ENSMUST00000168700.2	lysine-rich coiled-coil 1
3389	Ccr6	ENSMUST00000164411.3	chemokine (C-C motif) receptor 6
3390	Slc39a10	ENSMUST00000027131.5	solute carrier family 39 (zinc transporter), member 10
3391	Tubb2b	ENSMUST00000075774.3	tubulin, beta 2B class IIB
3392	Clpb	ENSMUST00000001884.8	ClpB caseinolytic peptidase B
3393	Ap5z1	ENSMUST00000038699.8	adaptor-related protein complex 5, zeta 1 subunit
3394	Exd2	ENSMUST00000038185.8	exonuclease 3'-5' domain containing 2
3395	Flrt1	ENSMUST00000113383.2	fibronectin leucine rich transmembrane protein 1
3396	Daam2	ENSMUST00000057610.6	dishevelled associated activator of morphogenesis 2
3397	Plekhf1	ENSMUST00000098513.4	pleckstrin homology domain containing, family F (with FYVE domain) member 1
3398	Cyp2d37-ps	ENSMUST00000100380.3	cytochrome P450, family 2, subfamily d, polypeptide 37, pseudogene
3399	Uty	ENSMUST00000143286.2	ubiquitously transcribed tetratricopeptide repeat gene, Y chromosome
3400	Zfml	ENSMUST00000032088.8	zinc finger, matrin-like
3401	Fbxo16	ENSMUST00000169656.1	F-box protein 16
3402	Asf1a	ENSMUST00000020004.6	anti-silencing function 1A histone chaperone
3403	Thoc1	ENSMUST00000025137.7	THO complex 1

3404	Serpinc1	ENSMUST00000064725.5	serine (or cysteine) peptidase inhibitor, clade C (antithrombin), member 1
3405	Cdc37l1	ENSMUST00000050148.3	cell division cycle 37-like 1
3406	Ndufa4	ENSMUST00000031637.5	NADH dehydrogenase (ubiquinone) 1 alpha subcomplex, 4
3407	Cacng8	ENSMUST00000182222.2	calcium channel, voltage-dependent, gamma subunit 8
3408	Slc22a22	ENSMUST00000022995.7	solute carrier family 22 (organic cation transporter), member 22
3409	Nupr1l	ENSMUST00000178355.1	nuclear protein transcriptional regulator 1 like
3410	Mboat2	ENSMUST00000110942.4	membrane bound O-acyltransferase domain containing 2
3411	Ophn1	ENSMUST00000033560.3	oligophrenin 1
3412	Ccdc71	ENSMUST00000061209.5	coiled-coil domain containing 71
3413	Abcd2	ENSMUST00000069511.6	ATP-binding cassette, sub-family D (ALD), member 2
3414	Depdc5	ENSMUST00000087897.5	DEP domain containing 5
3415	Helz	ENSMUST00000106746.2	helicase with zinc finger domain
3416	Snrnp35	ENSMUST00000031349.8	small nuclear ribonucleoprotein 35 (U11/U12)
3417	Vapb	ENSMUST00000067530.4	vesicle-associated membrane protein, associated protein B and C
3418	Maml3	ENSMUST00000121440.2	mastermind like 3 (Drosophila)
3419	Slc17a2	ENSMUST00000006786.5	solute carrier family 17 (sodium phosphate), member 2
3420	Pde4c	ENSMUST00000034307.8	phosphodiesterase 4C, cAMP specific
3421	Card6	ENSMUST00000118365.2	caspase recruitment domain family, member 6
3422	Samm50	ENSMUST00000023071.6	sorting and assembly machinery component 50 homolog (S. cerevisiae)
3423	Tsc22d2	ENSMUST00000099090.2	TSC22 domain family, member 2
3424	Epha7	ENSMUST00000029964.6	Eph receptor A7
3425	Plcl1	ENSMUST00000042986.9	phospholipase C-like 1
3426	Cyp2j9	ENSMUST00000055693.8	cytochrome P450, family 2, subfamily j, polypeptide 9
3427	Cdkn2b	ENSMUST00000097981.4	cyclin-dependent kinase inhibitor 2B (p15, inhibits CDK4)
3428	Zfp90	ENSMUST00000034382.7	zinc finger protein 90
3429	Zfp2	ENSMUST00000109129.2	zinc finger protein 2
3430	Prpf38a	ENSMUST00000079213.5	PRP38 pre-mRNA processing factor 38 (yeast) domain containing A
3431	Ttc39a	ENSMUST00000064129.8	tetratricopeptide repeat domain 39A
3432	Ccdc13	ENSMUST00000135986.3	coiled-coil domain containing 13
3433	Edf1	ENSMUST00000015236.3	endothelial differentiation-related factor 1
3434	Pmf1	ENSMUST00000056370.7	polyamine-modulated factor 1
3435	Rps3a1	ENSMUST00000029722.6	ribosomal protein S3A1
3436	Zfp30	ENSMUST00000032803.6	zinc finger protein 30
3437	Nrg2	ENSMUST00000115713.2	neuregulin 2
3438	Sf3b3	ENSMUST00000042012.5	splicing factor 3b, subunit 3
3439	Heatr2	ENSMUST00000026975.6	HEAT repeat containing 2
3440	Ppp1r7	ENSMUST00000027494.5	protein phosphatase 1, regulatory (inhibitor) subunit 7
3441	Itgbl1	ENSMUST00000049681.8	integrin, beta-like 1
3442	Ahdc1	ENSMUST00000105916.2	AT hook, DNA binding motif, containing 1

3443	Ccdc127	ENSMUST00000022063.8	coiled-coil domain containing 127
3444	Pgm3	ENSMUST00000070064.5	phosphoglucomutase 3
3445	Smim19	ENSMUST00000033935.9	small integral membrane protein 19
3446	Tmem261	ENSMUST00000030103.8	transmembrane protein 261
3447	Wdr86	ENSMUST00000068693.6	WD repeat domain 86
3448	Cpxm2	ENSMUST00000033149.4	carboxypeptidase X 2 (M14 family)
3449	Serpinb9b	ENSMUST00000006392.2	serine (or cysteine) peptidase inhibitor, clade B, member 9b
3450	Eif4e	ENSMUST00000029803.7	eukaryotic translation initiation factor 4E
3451	Spns3	ENSMUST00000092940.7	spinster homolog 3
3452	Fam65b	ENSMUST00000110383.2	family with sequence similarity 65, member B
3453	Ndufaf4	ENSMUST00000029925.4	NADH dehydrogenase (ubiquinone) 1 alpha subcomplex, assembly factor 4
3454	Cdk8	ENSMUST00000031640.9	cyclin-dependent kinase 8
3455	Lingo2	ENSMUST00000164772.2	leucine rich repeat and Ig domain containing 2
3456	Ahsa2	ENSMUST00000109539.2	AHA1, activator of heat shock protein ATPase 2
3457	Slc22a23	ENSMUST00000040336.6	solute carrier family 22, member 23
3458	Mnat1	ENSMUST00000021523.6	menage a trois 1
3459	Cd53	ENSMUST00000038845.8	CD53 antigen
3460	Lsg1	ENSMUST00000117363.3	large subunit GTPase 1 homolog (S. cerevisiae)
3461	Cisd2	ENSMUST00000029815.7	CDGSH iron sulfur domain 2
3462	Ski	ENSMUST00000084103.4	ski sarcoma viral oncogene homolog (avian)
3463	Hint3	ENSMUST00000161074.2	histidine triad nucleotide binding protein 3
3464	Txlna	ENSMUST00000046425.10	taxilin alpha
3465	Nipal3	ENSMUST00000102549.4	NIPA-like domain containing 3
3466	Rcsd1	ENSMUST00000040357.9	RCSD domain containing 1
3467	Cdca7l	ENSMUST00000021592.10	cell division cycle associated 7 like
3468	Plbd2	ENSMUST00000031597.6	phospholipase B domain containing 2
3469	Cox20	ENSMUST00000027781.6	COX20 Cox2 chaperone
3470	Zmynd8	ENSMUST00000109262.2	zinc finger, MYND-type containing 8
3471	Zzz3	ENSMUST00000106100.3	zinc finger, ZZ domain containing 3
3472	Pwp2	ENSMUST00000042556.9	PWP2 periodic tryptophan protein homolog (yeast)
3473	Lrp12	ENSMUST00000022916.6	low density lipoprotein-related protein 12
3474	Serpina10	ENSMUST00000044231.6	serine (or cysteine) peptidase inhibitor, clade A (alpha-1 antiproteinase, antitrypsin), member 10
3475	Igsf5	ENSMUST00000113794.2	immunoglobulin superfamily, member 5
3476	Mbd2	ENSMUST00000074058.5	methyl-CpG binding domain protein 2
3477	Fbxo25	ENSMUST00000043520.3	F-box protein 25
3478	Runx3	ENSMUST00000056977.8	runt related transcription factor 3
3479	Plekho2	ENSMUST00000068944.7	pleckstrin homology domain containing, family O member 2
3480	Fastkd5	ENSMUST00000110262.1	FAST kinase domains 5
3481	Me2	ENSMUST00000025439.3	malic enzyme 2, NAD(+)-dependent, mitochondrial
3482	Mfsd3	ENSMUST00000019224.7	major facilitator superfamily domain containing 3
3483	Ormdl2	ENSMUST00000026409.3	ORM1-like 2 (S. cerevisiae)
3484	Zfp128	ENSMUST00000144578.1	zinc finger protein 128
3485	Sfr1	ENSMUST00000099353.4	SWI5 dependent recombination repair 1
3486	1700049G17Rik	ENSMUST00000098639.3	RIKEN cDNA 1700049G17 gene
3487	Rit1	ENSMUST00000029692.9	Ras-like without CAAX 1
3488	CTNND1	ENSMUST00000189772.1	Catenin delta-1
3489	Mfsd7b	ENSMUST00000085635.4	major facilitator superfamily domain containing 7B
3490	Gfm1	ENSMUST00000077271.7	G elongation factor, mitochondrial 1

3491	Tex2	ENSMUST00000042780.8	testis expressed gene 2
3492	Hnrnpll	ENSMUST00000184635.2	heterogeneous nuclear ribonucleoprotein L-like
3493	Zfp599	ENSMUST00000086281.4	zinc finger protein 599
3494	Ctnnd1	ENSMUST00000111696.2	catenin (cadherin associated protein), delta 1
3495	Vmp1	ENSMUST00000018315.4	vacuole membrane protein 1
3496	Nucb2	ENSMUST00000032895.9	nucleobindin 2
3497	Fap	ENSMUST00000102732.4	fibroblast activation protein
3498	Ext1	ENSMUST00000077273.3	exostoses (multiple) 1
3499	Gdf3	ENSMUST00000032211.4	growth differentiation factor 3
3500	Daglb	ENSMUST00000045593.9	diacylglycerol lipase, beta
3501	Mbl2	ENSMUST00000025797.5	mannose-binding lectin (protein C) 2
3502	Dicer1	ENSMUST00000041987.6	dicer 1, ribonuclease type III
3503	Exd1	ENSMUST00000060009.3	exonuclease 3'-5' domain containing 1
3504	Srcin1	ENSMUST00000107596.3	SRC kinase signaling inhibitor 1
3505	AU022252	ENSMUST00000141112.1	expressed sequence AU022252
3506	Psd4	ENSMUST00000102942.2	pleckstrin and Sec7 domain containing 4
3507	Prkra	ENSMUST00000002808.6	protein kinase, interferon inducible double stranded RNA dependent activator
3508	Surf4	ENSMUST00000015011.4	surfeit gene 4
3509	Ecm2	ENSMUST00000051504.7	extracellular matrix protein 2, female organ and adipocyte specific
3510	Them7	ENSMUST00000068813.2	thioesterase superfamily member 7
3511	Cdc42ep2	ENSMUST00000055458.4	CDC42 effector protein (Rho GTPase binding) 2
3512	Zfp623	ENSMUST00000037260.6	zinc finger protein 623
3513	Nup153	ENSMUST00000021803.9	nucleoporin 153
3514	Tnfrsf11a	ENSMUST00000027559.7	tumor necrosis factor receptor superfamily, member 11a
3515	Ldhb	ENSMUST00000032373.6	lactate dehydrogenase B
3516	Sod3	ENSMUST00000101208.4	superoxide dismutase 3, extracellular
3517	Prep	ENSMUST00000099858.2	prolyl endopeptidase
3518	Ppp2ca	ENSMUST00000020608.2	protein phosphatase 2 (formerly 2A), catalytic subunit, alpha isoform
3519	Fbxl22	ENSMUST00000056890.8	F-box and leucine-rich repeat protein 22
3520	Erlec1	ENSMUST00000073192.8	endoplasmic reticulum lectin 1
3521	Birc6	ENSMUST00000180037.2	baculoviral IAP repeat-containing 6
3522	Trdn	ENSMUST00000095762.4	triadin
3523	Psen1	ENSMUST00000101225.1	presenilin 1
3524	Rnaseh2b	ENSMUST00000022499.7	ribonuclease H2, subunit B
3525	Ttn	ENSMUST00000099980.4	titin
3526	Csrp2bp	ENSMUST00000028911.9	cysteine and glycine-rich protein 2 binding protein
3527	Itgb1bp1	ENSMUST00000076260.5	integrin beta 1 binding protein 1
3528	Nomo1	ENSMUST00000033121.6	nodal modulator 1
3529	Abcd3	ENSMUST00000029770.5	ATP-binding cassette, sub-family D (ALD), member 3
3530	9130230L23Rik	ENSMUST00000067737.6	RIKEN cDNA 9130230L23 gene
3531	Scg3	ENSMUST00000034699.6	secretogranin III
3532	Hoxb4	ENSMUST00000049241.8	homeobox B4
3533	Rps3	ENSMUST00000032998.7	ribosomal protein S3
3534	Acadsb	ENSMUST00000015829.8	acyl-Coenzyme A dehydrogenase, short/branched chain
3535	Atxn10	ENSMUST00000163242.1	ataxin 10
3536	Pofut1	ENSMUST00000049863.6	protein O-fucosyltransferase 1

3537	Rrp12	ENSMUST00000038677.3	ribosomal RNA processing 12 homolog (S. cerevisiae)
3538	Msi2	ENSMUST00000107909.2	musashi RNA-binding protein 2
3539	Oit3	ENSMUST00000009798.4	oncoprotein induced transcript 3
3540	Phactr3	ENSMUST00000108917.2	phosphatase and actin regulator 3
3541	Ccdc135	ENSMUST00000058479.6	coiled-coil domain containing 135
3542	Hddc2	ENSMUST00000000304.7	HD domain containing 2
3543	Tmod2	ENSMUST00000064433.4	tropomodulin 2
3544	Fam50a	ENSMUST00000114160.1	family with sequence similarity 50, member A
3545	Ccs	ENSMUST00000037246.5	copper chaperone for superoxide dismutase
3546	Prpf38b	ENSMUST00000029480.8	PRP38 pre-mRNA processing factor 38 (yeast) domain containing B
3547	Il15	ENSMUST00000034148.6	interleukin 15
3548	Tpp2	ENSMUST00000188313.1	tripeptidyl peptidase II
3549	Maml2	ENSMUST00000177755.1	mastermind like 2 (Drosophila)
3550	Efcab2	ENSMUST00000027775.7	EF-hand calcium binding domain 2
3551	Il17ra	ENSMUST00000002976.3	interleukin 17 receptor A
3552	Dnajc1	ENSMUST00000166495.2	DnaJ (Hsp40) homolog, subfamily C, member 1
3553	Nanp	ENSMUST00000066640.4	N-acetylneuraminic acid phosphatase
3554	Ube2e3	ENSMUST00000028398.8	ubiquitin-conjugating enzyme E2E 3
3555	Fzd8	ENSMUST00000041080.5	frizzled homolog 8 (Drosophila)
3556	Pklr	ENSMUST00000107482.4	pyruvate kinase liver and red blood cell
3557	Pelo	ENSMUST00000109226.3	pelota homolog (Drosophila)
3558	Ept1	ENSMUST00000145167.2	ethanolaminephosphotransferase 1 (CDP-ethanolamine-specific)
3559	Hk1	ENSMUST00000099691.5	hexokinase 1
3560	Neurl1b	ENSMUST00000053020.7	neuralized homolog 1b (Drosophila)
3561	5830415F09Rik	ENSMUST00000030015.5	RIKEN cDNA 5830415F09 gene
3562	Tspan15	ENSMUST00000047883.9	tetraspanin 15
3563	Mri1	ENSMUST00000126435.2	methylthioribose-1-phosphate isomerase homolog (S. cerevisiae)
3564	Dusp11	ENSMUST00000032071.9	dual specificity phosphatase 11 (RNA/RNP complex 1-interacting)
3565	Taf3	ENSMUST00000026888.5	TAF3 RNA polymerase II, TATA box binding protein (TBP)-associated factor
3566	Medag	ENSMUST00000093110.6	mesenteric estrogen dependent adipogenesis
3567	Sin3a	ENSMUST00000167715.2	transcriptional regulator, SIN3A (yeast)
3568	Lrrc14b	ENSMUST00000022064.4	leucine rich repeat containing 14B
3569	Nfe2l1	ENSMUST00000169828.2	nuclear factor, erythroid derived 2,-like 1
3570	Dcun1d5	ENSMUST00000034499.8	DCN1, defective in cullin neddylation 1, domain containing 5 (S. cerevisiae)
3571	Snrpf	ENSMUST00000020203.6	small nuclear ribonucleoprotein polypeptide F
3572	Iars	ENSMUST00000165316.2	isoleucine-tRNA synthetase
3573	Fastkd1	ENSMUST00000073152.7	FAST kinase domains 1
3574	Arhgap24	ENSMUST00000073302.6	Rho GTPase activating protein 24
3575	Tax1bp1	ENSMUST00000080723.5	Tax1 (human T cell leukemia virus type I) binding protein 1
3576	Myo5a	ENSMUST00000123128.2	myosin VA
3577	Mrps26	ENSMUST00000145614.1	mitochondrial ribosomal protein S26
3578	Pycard	ENSMUST00000033056.3	PYD and CARD domain containing
3579	Tanc1	ENSMUST00000037526.5	tetratricopeptide repeat, ankyrin repeat and coiled-coil containing 1

3580	Gm3043	ENSMUST00000170639.1	predicted gene 3043
3581	Sqstm1	ENSMUST00000015981.6	sequestosome 1
3582	Btrc	ENSMUST00000065601.6	beta-transducin repeat containing protein
3583	Psmb5	ENSMUST00000022803.4	proteasome (prosome, macropain) subunit, beta type 5
3584	Orc4	ENSMUST00000028098.5	origin recognition complex, subunit 4
3585	Ndufs4	ENSMUST00000022286.6	NADH dehydrogenase (ubiquinone) Fe-S protein 4
3586	Scaf8	ENSMUST00000076734.6	SR-related CTD-associated factor 8
3587	Mdfic	ENSMUST00000189359.1	MyoD family inhibitor domain containing
3588	Arid5a	ENSMUST00000137906.1	AT rich interactive domain 5A (MRF1-like)
3589	Dennd2a	ENSMUST00000036877.4	DENN/MADD domain containing 2A
3590	Pgm5	ENSMUST00000047666.4	phosphoglucomutase 5
3591	Emcn	ENSMUST00000122064.2	endomucin
3592	Fam227a	ENSMUST00000109648.3	family with sequence similarity 227, member A
3593	Ebpl	ENSMUST00000022494.8	emopamil binding protein-like
3594	C87436	ENSMUST00000050497.8	expressed sequence C87436
3595	Plet1	ENSMUST00000114474.2	placenta expressed transcript 1
3596	Nrf1	ENSMUST00000115212.2	nuclear respiratory factor 1
3597	Kcnn3	ENSMUST00000000811.7	potassium intermediate/small conductance calcium-activated channel, subfamily N, member 3
3598	Tmem44	ENSMUST00000140402.2	transmembrane protein 44
3599	Gga2	ENSMUST00000033160.9	golgi associated, gamma adaptin ear containing, ARF binding protein 2
3600	Mob3b	ENSMUST00000102975.4	MOB kinase activator 3B
3601	Stx7	ENSMUST00000020174.5	syntaxin 7
3602	Gbp5	ENSMUST00000029936.4	guanylate binding protein 5
3603	Rbms2	ENSMUST00000099139.2	RNA binding motif, single stranded interacting protein 2
3604	Zfp609	ENSMUST00000159109.1	zinc finger protein 609
3605	Anxa6	ENSMUST00000108883.4	annexin A6
3606	Cenpf	ENSMUST00000171929.2	centromere protein F
3607	Magea1	ENSMUST00000078193.4	melanoma antigen, family A, 1
3608	Fam161a	ENSMUST00000109557.3	family with sequence similarity 161, member A
3609	Cyp2b10	ENSMUST00000072438.7	cytochrome P450, family 2, subfamily b, polypeptide 10
3610	D10Wsu102e	ENSMUST00000020488.7	DNA segment, Chr 10, Wayne State University 102, expressed
3611	Chmp5	ENSMUST00000030128.5	charged multivesicular body protein 5
3612	Pik3r6	ENSMUST00000102613.2	phosphoinositide-3-kinase, regulatory subunit 6
3613	Slc35b4	ENSMUST00000019143.8	solute carrier family 35, member B4
3614	Alpk1	ENSMUST00000029662.6	alpha-kinase 1
3615	C8b	ENSMUST00000031663.4	complement component 8, beta polypeptide
3616	Adcy8	ENSMUST00000023007.5	adenylate cyclase 8
3617	Kidins220	ENSMUST00000066652.5	kinase D-interacting substrate 220
3618	Emx2	ENSMUST00000062216.3	empty spiracles homeobox 2
3619	Naa35	ENSMUST00000022038.8	N(alpha)-acetyltransferase 35, NatC auxiliary subunit
3620	Wdr77	ENSMUST00000010278.6	WD repeat domain 77
3621	Zc3h14	ENSMUST00000110105.3	zinc finger CCCH type containing 14
3622	Ces3b	ENSMUST00000093221.7	carboxylesterase 3B
3623	Clasp1	ENSMUST00000188710.1	CLIP associating protein 1
3624	Saal1	ENSMUST00000143082.1	serum amyloid A-like 1
3625	Nme2	ENSMUST00000021217.5	NME/NM23 nucleoside diphosphate kinase 2

3626	Kctd1	ENSMUST00000168989.2	potassium channel tetramerisation domain containing 1
3627	Atg12	ENSMUST00000035648.4	autophagy related 12
3628	Gcom1	ENSMUST00000166843.2	GRINL1A complex locus
3629	Tars	ENSMUST00000022849.5	threonyl-tRNA synthetase
3630	Plekhg2	ENSMUST00000094644.5	pleckstrin homology domain containing, family G (with RhoGef domain) member 2
3631	Acp2	ENSMUST00000002172.8	acid phosphatase 2, lysosomal
3632	Polr2m	ENSMUST00000034720.6	polymerase (RNA) II (DNA directed) polypeptide M
3633	Ppp1r36	ENSMUST00000063977.7	protein phosphatase 1, regulatory subunit 36
3634	Ubr5	ENSMUST00000110336.2	ubiquitin protein ligase E3 component n-recognin 5
3635	Wbscr17	ENSMUST00000086023.6	Williams-Beuren syndrome chromosome region 17 homolog (human)
3636	Esrp2	ENSMUST00000115979.3	epithelial splicing regulatory protein 2
3637	Cd93	ENSMUST00000099269.3	CD93 antigen
3638	Taz	ENSMUST00000124200.2	tafazzin
3639	Nop9	ENSMUST00000019441.8	NOP9 nucleolar protein
3640	Zfp318	ENSMUST00000113481.2	zinc finger protein 318
3641	Mfng	ENSMUST00000018313.5	MFNG O-fucosylpeptide 3-beta-N-acetylglucosaminyltransferase
3642	Lect2	ENSMUST00000062806.4	leukocyte cell-derived chemotaxin 2
3643	Cdh10	ENSMUST00000166873.3	cadherin 10
3644	Ints7	ENSMUST00000045450.5	integrator complex subunit 7
3645	Nup155	ENSMUST00000163765.1	nucleoporin 155
3646	Dusp7	ENSMUST00000172306.2	dual specificity phosphatase 7
3647	Upk1b	ENSMUST00000057767.4	uroplakin 1B
3648	Nelfe	ENSMUST00000165953.2	negative elongation factor complex member E, Rdbp
3649	Eml6	ENSMUST00000058902.5	echinoderm microtubule associated protein like 6
3650	Smc3	ENSMUST00000025930.9	structural maintenance of chromosomes 3
3651	Pole4	ENSMUST00000095786.5	polymerase (DNA-directed), epsilon 4 (p12 subunit)
3652	Gcc1	ENSMUST00000090511.3	golgi coiled coil 1
3653	Tmc6	ENSMUST00000026659.4	transmembrane channel-like gene family 6
3654	Lgals3bp	ENSMUST00000043722.4	lectin, galactoside-binding, soluble, 3 binding protein
3655	Ska1	ENSMUST00000040188.9	spindle and kinetochore associated complex subunit 1
3656	Nxpe3	ENSMUST00000099705.3	neurexophilin and PC-esterase domain family, member 3
3657	Nkx2-5	ENSMUST00000015723.4	NK2 homeobox 5
3658	Atg2b	ENSMUST00000041055.7	autophagy related 2B
3659	Cldn25	ENSMUST00000023426.6	claudin 25
3660	Katnbl1	ENSMUST00000028552.3	katanin p80 subunit B like 1
3661	Irx2	ENSMUST00000074372.5	Iroquois related homeobox 2 (Drosophila)
3662	Bcl11a	ENSMUST00000109516.2	B cell CLL/lymphoma 11A (zinc finger protein)
3663	Thap3	ENSMUST00000036680.2	THAP domain containing, apoptosis associated protein 3
3664	Ccl9	ENSMUST00000019266.5	chemokine (C-C motif) ligand 9
3665	Zbtb21	ENSMUST00000113734.3	zinc finger and BTB domain containing 21
3666	Mbd3	ENSMUST00000092295.4	methyl-CpG binding domain protein 3
3667	Eea1	ENSMUST00000053484.6	early endosome antigen 1
3668	Hsd17b6	ENSMUST00000026462.6	hydroxysteroid (17-beta) dehydrogenase 6
3669	Traf7	ENSMUST00000176652.2	TNF receptor-associated factor 7
3670	Slc8a1	ENSMUST00000163680.3	solute carrier family 8 (sodium/calcium exchanger), member 1

3671	Mios	ENSMUST00000040017.7	missing oocyte, meiosis regulator, homolog (Drosophila)
3672	Echdc1	ENSMUST00000020034.4	enoyl Coenzyme A hydratase domain containing 1
3673	Pddc1	ENSMUST00000106008.1	Parkinson disease 7 domain containing 1
3674	Gspt2	ENSMUST00000096368.3	G1 to S phase transition 2
3675	Manbal	ENSMUST00000081202.5	mannosidase, beta A, lysosomal-like
3676	Crp	ENSMUST00000038495.3	C-reactive protein, pentraxin-related
3677	Senp6	ENSMUST00000037484.9	SUMO/sentrin specific peptidase 6
3678	Tnfaip3	ENSMUST00000019997.5	tumor necrosis factor, alpha-induced protein 3
3679	Lctl	ENSMUST00000034969.8	lactase-like
3680	Dennd6b	ENSMUST00000078953.7	DENN/MADD domain containing 6B
3681	Hnrnpr	ENSMUST00000148843.4	heterogeneous nuclear ribonucleoprotein R
3682	Srfbp1	ENSMUST00000025406.7	serum response factor binding protein 1
3683	Ikbkap	ENSMUST00000030140.2	inhibitor of kappa light polypeptide enhancer in B cells, kinase complex-associated protein
3684	Rpp30	ENSMUST00000025714.7	ribonuclease P/MRP 30 subunit
3685	Mrps31	ENSMUST00000033934.3	mitochondrial ribosomal protein S31
3686	Rab13	ENSMUST00000107373.2	RAB13, member RAS oncogene family
3687	Gas2l1	ENSMUST00000056649.7	growth arrest-specific 2 like 1
3688	Pygb	ENSMUST00000045441.7	brain glycogen phosphorylase
3689	Gm20390	ENSMUST00000170303.1	predicted gene 20390
3690	Commd8	ENSMUST00000013693.6	COMM domain containing 8
3691	Cdc23	ENSMUST00000133181.1	CDC23 cell division cycle 23
3692	Fam174a	ENSMUST00000059975.7	family with sequence similarity 174, member A
3693	Hdlbp	ENSMUST00000170883.2	high density lipoprotein (HDL) binding protein
3694	Arhgap31	ENSMUST00000023487.4	Rho GTPase activating protein 31
3695	Psip1	ENSMUST00000030207.9	PC4 and SFRS1 interacting protein 1
3696	Herc4	ENSMUST00000020258.8	hect domain and RLD 4
3697	Mcm4	ENSMUST00000023353.3	minichromosome maintenance deficient 4 homolog (S. cerevisiae)
3698	Epb4.1l4a	ENSMUST00000025234.5	erythrocyte protein band 4.1-like 4a
3699	Tbp	ENSMUST00000162505.2	TATA box binding protein
3700	Lst1	ENSMUST00000097336.4	leukocyte specific transcript 1
3701	Fbxw17	ENSMUST00000046974.4	F-box and WD-40 domain protein 17
3702	Naalad2	ENSMUST00000172171.2	N-acetylated alpha-linked acidic dipeptidase 2
3703	Rad51d	ENSMUST00000018985.9	RAD51 homolog D
3704	Chchd5	ENSMUST00000035481.4	coiled-coil-helix-coiled-coil-helix domain containing 5
3705	Hmmr	ENSMUST00000020579.8	hyaluronan mediated motility receptor (RHAMM)
3706	2810021J22Rik	ENSMUST00000073924.4	RIKEN cDNA 2810021J22 gene
3707	Tnfsf12Tnfsf13	ENSMUST00000180587.2	Tnfsf12-Tnfsf13 readthrough transcript
3708	Cnpy2	ENSMUST00000026446.2	canopy 2 homolog (zebrafish)
3709	Eogt	ENSMUST00000113387.2	EGF domain-specific O-linked N-acetylglucosamine (GlcNAc) transferase
3710	Klhdc7a	ENSMUST00000105031.3	kelch domain containing 7A
3711	Extl2	ENSMUST00000029575.6	exostoses (multiple)-like 2
3712	2700060E02Rik	ENSMUST00000022341.5	RIKEN cDNA 2700060E02 gene
3713	Ulk4	ENSMUST00000171923.2	unc-51-like kinase 4
3714	AI464131	ENSMUST00000054920.4	expressed sequence AI464131
3715	Acot12	ENSMUST00000022120.4	acyl-CoA thioesterase 12
3716	Nop58	ENSMUST00000191142.1	NOP58 ribonucleoprotein
3717	Dnaaf2	ENSMUST00000021356.5	dynein, axonemal assembly factor 2

3718	Zfp276	ENSMUST00000001092.9	zinc finger protein (C2H2 type) 276
3719	Rab9	ENSMUST00000112091.3	RAB9, member RAS oncogene family
3720	Mkrn1	ENSMUST00000114823.2	makorin, ring finger protein, 1
3721	Ccdc34	ENSMUST00000028580.6	coiled-coil domain containing 34
3722	Med28	ENSMUST00000118833.1	mediator complex subunit 28
3723	E030030I06Rik	ENSMUST00000069372.6	RIKEN cDNA E030030I06 gene
3724	Fem1b	ENSMUST00000034775.8	feminization 1 homolog b (C. elegans)
3725	Ly6c1	ENSMUST00000065408.10	lymphocyte antigen 6 complex, locus C1
3726	Klhl12	ENSMUST00000027725.5	kelch-like 12
3727	Mrpl32	ENSMUST00000015816.3	mitochondrial ribosomal protein L32
3728	Rap2b	ENSMUST00000049064.2	RAP2B, member of RAS oncogene family
3729	Rnf146	ENSMUST00000037548.5	ring finger protein 146
3730	Tmem173	ENSMUST00000115728.3	transmembrane protein 173
3731	Tnfsf13	ENSMUST00000018896.8	tumor necrosis factor (ligand) superfamily, member 13
3732	Gm5113	ENSMUST00000085668.4	predicted gene 5113
3733	Pfkfb2	ENSMUST00000066863.7	6-phosphofructo-2-kinase/fructose-2,6-biphosphatase 2
3734	Ubb	ENSMUST00000019649.3	ubiquitin B
3735	Dcun1d1	ENSMUST00000178098.2	DCN1, defective in cullin neddylation 1, domain containing 1 (S. cerevisiae)
3736	Prl7a2	ENSMUST00000006660.5	prolactin family 7, subfamily a, member 2
3737	Ttc30b	ENSMUST00000099996.4	tetratricopeptide repeat domain 30B
3738	9130019O22Rik	ENSMUST00000049052.3	RIKEN cDNA 9130019O22 gene
3739	Fga	ENSMUST00000029630.9	fibrinogen alpha chain
3740	Hip1r	ENSMUST00000000939.9	huntingtin interacting protein 1 related
3741	Atp1b2	ENSMUST00000047889.7	ATPase, Na+/K+ transporting, beta 2 polypeptide
3742	Srpk2	ENSMUST00000088392.4	serine/arginine-rich protein specific kinase 2
3743	Lmf1	ENSMUST00000063344.9	lipase maturation factor 1
3744	Cfh	ENSMUST00000066859.7	complement component factor h
3745	Tor1a	ENSMUST00000028200.8	torsin family 1, member A (torsin A)
3746	Zscan21	ENSMUST00000110961.3	zinc finger and SCAN domain containing 21
3747	Slc7a4	ENSMUST00000090165.4	solute carrier family 7 (cationic amino acid transporter, y+ system), member 4
3748	Dok2	ENSMUST00000022698.7	docking protein 2
3749	Sdhc	ENSMUST00000111336.4	succinate dehydrogenase complex, subunit C, integral membrane protein
3750	Ift52	ENSMUST00000018002.7	intraflagellar transport 52
3751	Parp3	ENSMUST00000067218.8	poly (ADP-ribose) polymerase family, member 3
3752	Hgsnat	ENSMUST00000037609.6	heparan-alpha-glucosaminide N-acetyltransferase
3753	Myoz3	ENSMUST00000056533.7	myozenin 3
3754	Cyp2b9	ENSMUST00000082214.4	cytochrome P450, family 2, subfamily b, polypeptide 9
3755	Camk2n1	ENSMUST00000050918.3	calcium/calmodulin-dependent protein kinase II inhibitor 1
3756	Tsku	ENSMUST00000165257.2	tsukushi
3757	Znrd1as	ENSMUST00000040177.6	Znrd1 antisense
3758	Esyt2	ENSMUST00000100986.2	extended synaptotagmin-like protein 2
3759	Nosip	ENSMUST00000003513.4	nitric oxide synthase interacting protein
3760	Arhgap1	ENSMUST00000111329.2	Rho GTPase activating protein 1
3761	Tnip1	ENSMUST00000108889.4	TNFAIP3 interacting protein 1

3762	Eif2s3y	ENSMUST00000091197.3	eukaryotic translation initiation factor 2, subunit 3, structural gene Y-linked
3763	Gabbr1	ENSMUST00000025338.10	gamma-aminobutyric acid (GABA) B receptor, 1
3764	Dpt	ENSMUST00000027861.4	dermatopontin
3765	Slc2a8	ENSMUST00000028129.7	solute carrier family 2, (facilitated glucose transporter), member 8
3766	Otulin	ENSMUST00000059662.7	OTU deubiquitinase with linear linkage specificity
3767	2210016F16Rik	ENSMUST00000022032.5	RIKEN cDNA 2210016F16 gene
3768	Aldh16a1	ENSMUST00000007977.8	aldehyde dehydrogenase 16 family, member A1
3769	Rgs5	ENSMUST00000027997.3	regulator of G-protein signaling 5
3770	AU021092	ENSMUST00000050160.4	expressed sequence AU021092
3771	Kif2a	ENSMUST00000022204.10	kinesin family member 2A
3772	Lmo4	ENSMUST00000120539.2	LIM domain only 4
3773	Snx13	ENSMUST00000048519.10	sorting nexin 13
3774	Adprh	ENSMUST00000002923.8	ADP-ribosylarginine hydrolase
3775	Denr	ENSMUST00000023869.9	density-regulated protein
3776	Cdc37	ENSMUST00000019615.9	cell division cycle 37
3777	Zfp759	ENSMUST00000052716.7	zinc finger protein 759
3778	Tmem212	ENSMUST00000058077.3	transmembrane protein 212
3779	Rag2	ENSMUST00000044031.3	recombination activating gene 2
3780	Wfdc1	ENSMUST00000024107.5	WAP four-disulfide core domain 1
3781	Myo1e	ENSMUST00000034745.7	myosin IE
3782	C3ar1	ENSMUST00000042081.8	complement component 3a receptor 1
3783	Fuca1	ENSMUST00000030434.4	fucosidase, alpha-L- 1, tissue
3784	Yipf1	ENSMUST00000075693.6	Yip1 domain family, member 1
3785	Gm10638	ENSMUST00000098532.2	predicted gene 10638
3786	Pold4	ENSMUST00000025773.3	polymerase (DNA-directed), delta 4
3787	Ikbke	ENSMUST00000062108.4	inhibitor of kappaB kinase epsilon
3788	Csnk2a2	ENSMUST00000056919.7	casein kinase 2, alpha prime polypeptide
3789	Nedd4l	ENSMUST00000163516.2	neural precursor cell expressed, developmentally down-regulated gene 4-like
3790	Phf10	ENSMUST00000024657.6	PHD finger protein 10
3791	Ccdc58	ENSMUST00000164916.2	coiled-coil domain containing 58
3792	Mms19	ENSMUST00000171561.2	MMS19 (MET18 S. cerevisiae)
3793	Cask	ENSMUST00000115438.4	calcium/calmodulin-dependent serine protein kinase (MAGUK family)
3794	Saysd1	ENSMUST00000059666.4	SAYSVFN motif domain containing 1
3795	Ms4a4d	ENSMUST00000025581.6	membrane-spanning 4-domains, subfamily A, member 4D
3796	Ces1c	ENSMUST00000034189.9	carboxylesterase 1C
3797	Pax8	ENSMUST00000153601.2	paired box 8
3798	Lama3	ENSMUST00000092070.7	laminin, alpha 3
3799	Adamts5	ENSMUST00000023611.5	a disintegrin-like and metallopeptidase (reprolysin type) with thrombospondin type 1 motif, 5 (aggrecanase-2)
3800	Bhmt	ENSMUST00000099309.4	betaine-homocysteine methyltransferase
3801	Cyp2e1	ENSMUST00000026552.7	cytochrome P450, family 2, subfamily e, polypeptide 1
3802	Dlk1	ENSMUST00000056110.9	delta-like 1 homolog (Drosophila)
3803	Colec12	ENSMUST00000040069.8	collectin sub-family member 12
3804	Kcng2	ENSMUST00000077962.7	potassium voltage-gated channel, subfamily G, member 2

3805	Myl9	ENSMUST00000088552.6	myosin, light polypeptide 9, regulatory
3806	Kdelc1	ENSMUST00000065767.3	KDEL (Lys-Asp-Glu-Leu) containing 1
3807	Mtmr9	ENSMUST00000058679.5	myotubularin related protein 9
3808	Anxa4	ENSMUST00000113675.2	annexin A4
3809	Hist2h3c2	ENSMUST00000167403.3	histone cluster 2, H3c2
3810	Dgcr14	ENSMUST00000003621.8	DiGeorge syndrome critical region gene 14
3811	Apon	ENSMUST00000060782.3	apolipoprotein N
3812	Ipo7	ENSMUST00000084731.3	importin 7
3813	Rmnd1	ENSMUST00000042251.5	required for meiotic nuclear division 1 homolog (S. cerevisiae)
3814	Zmat2	ENSMUST00000001419.8	zinc finger, matrin type 2
3815	Pyroxd2	ENSMUST00000076505.3	pyridine nucleotide-disulphide oxidoreductase domain 2
3816	Naga	ENSMUST00000023088.7	N-acetyl galactosaminidase, alpha
3817	Ict1	ENSMUST00000153983.2	immature colon carcinoma transcript 1
3818	Slx4ip	ENSMUST00000099311.3	SLX4 interacting protein
3819	Zbtb9	ENSMUST00000120016.1	zinc finger and BTB domain containing 9
3820	Atp1a4	ENSMUST00000111243.1	ATPase, Na+/K+ transporting, alpha 4 polypeptide
3821	Cnih3	ENSMUST00000027795.8	cornichon homolog 3 (Drosophila)
3822	Ier5	ENSMUST00000055322.5	immediate early response 5
3823	Bmpr1a	ENSMUST00000165280.2	bone morphogenetic protein receptor, type 1A
3824	Slc43a1	ENSMUST00000111625.1	solute carrier family 43, member 1
3825	Hpd	ENSMUST00000031398.8	4-hydroxyphenylpyruvic acid dioxygenase
3826	Ralbp1	ENSMUST00000166543.3	ralA binding protein 1
3827	Slc9a9	ENSMUST00000033463.9	solute carrier family 9 (sodium/hydrogen exchanger), member 9
3828	Donson	ENSMUST00000117159.2	downstream neighbor of SON
3829	Csad	ENSMUST00000023805.1	cysteine sulfinic acid decarboxylase
3830	Fat1	ENSMUST00000191428.1	FAT tumor suppressor homolog 1 (Drosophila)
3831	Hrsp12	ENSMUST00000022946.5	heat-responsive protein 12
3832	Tspan14	ENSMUST00000047652.5	tetraspanin 14
3833	Rwdd3	ENSMUST00000170781.1	RWD domain containing 3
3834	Akr1c6	ENSMUST00000021630.9	aldo-keto reductase family 1, member C6
3835	Pm20d1	ENSMUST00000112393.3	peptidase M20 domain containing 1
3836	Zfp119a	ENSMUST00000079642.7	zinc finger protein 119a
3837	Chd7	ENSMUST00000051558.4	chromodomain helicase DNA binding protein 7
3838	Skp1a	ENSMUST00000037324.6	S-phase kinase-associated protein 1A
3839	2310057M21Rik	ENSMUST00000059438.9	RIKEN cDNA 2310057M21 gene
3840	Cyp4f39	ENSMUST00000003413.6	cytochrome P450, family 4, subfamily f, polypeptide 39
3841	Hs3st6	ENSMUST00000044922.6	heparan sulfate (glucosamine) 3-O-sulfotransferase 6
3842	Eif4ebp1	ENSMUST00000033880.5	eukaryotic translation initiation factor 4E binding protein 1
3843	Hp1bp3	ENSMUST00000105827.2	heterochromatin protein 1, binding protein 3
3844	Tbc1d30	ENSMUST00000064107.5	TBC1 domain family, member 30
3845	Nt5m	ENSMUST00000102695.3	5',3'-nucleotidase, mitochondrial
3846	Atp6v1e1	ENSMUST00000019354.8	ATPase, H+ transporting, lysosomal V1 subunit E1
3847	Asb16	ENSMUST00000036467.4	ankyrin repeat and SOCS box-containing 16
3848	Mfn1	ENSMUST00000091257.5	mitofusin 1
3849	Thumpd3	ENSMUST00000032398.9	THUMP domain containing 3
3850	Aagab	ENSMUST00000041551.7	alpha- and gamma-adaptin binding protein
3851	Bcat1	ENSMUST00000032402.6	branched chain aminotransferase 1, cytosolic

3852	Olfr630	ENSMUST00000106880.1	olfactory receptor 630
3853	Polh	ENSMUST00000024749.7	polymerase (DNA directed), eta (RAD 30 related)
3854	Sept7	ENSMUST00000115272.3	septin 7
3855	Sumo3	ENSMUST00000020501.9	SMT3 suppressor of mif two 3 homolog 3 (yeast)
3856	Lphn3	ENSMUST00000121707.2	latrophilin 3
3857	Mbip	ENSMUST00000021416.7	MAP3K12 binding inhibitory protein 1
3858	Lgmn	ENSMUST00000110020.2	legumain
3859	Serpina3c	ENSMUST00000085050.3	serine (or cysteine) peptidase inhibitor, clade A, member 3C
3860	Copg1	ENSMUST00000113607.4	coatomer protein complex, subunit gamma 1
3861	Prorsd1	ENSMUST00000133103.1	prolyl-tRNA synthetase domain containing 1
3862	Hspa5	ENSMUST00000028222.7	heat shock protein 5
3863	Ptp4a2	ENSMUST00000165853.1	protein tyrosine phosphatase 4a2
3864	Syt10	ENSMUST00000029441.3	synaptotagmin X
3865	Pspc1	ENSMUST00000022507.6	paraspeckle protein 1
3866	Chd1	ENSMUST00000024620.6	chromodomain helicase DNA binding protein 1
3867	Sdf4	ENSMUST00000050078.7	stromal cell derived factor 4
3868	Mrs2	ENSMUST00000021772.2	MRS2 magnesium homeostasis factor homolog (S. cerevisiae)
3869	Erp44	ENSMUST00000030028.4	endoplasmic reticulum protein 44
3870	Rrp15	ENSMUST00000001339.5	ribosomal RNA processing 15 homolog (S. cerevisiae)
3871	Sf3b4	ENSMUST00000076372.4	splicing factor 3b, subunit 4
3872	Ankrd42	ENSMUST00000118157.2	ankyrin repeat domain 42
3873	Hcfc2	ENSMUST00000020478.8	host cell factor C2
3874	Notum	ENSMUST00000106177.2	notum pectinacetylesterase homolog (Drosophila)
3875	Eif3e	ENSMUST00000022960.2	eukaryotic translation initiation factor 3, subunit E
3876	Ndufv2	ENSMUST00000143987.2	NADH dehydrogenase (ubiquinone) flavoprotein 2
3877	S100a10	ENSMUST00000045756.8	S100 calcium binding protein A10 (calpactin)
3878	Dysf	ENSMUST00000113821.2	dysferlin
3879	Zbtb3	ENSMUST00000172175.1	zinc finger and BTB domain containing 3
3880	Traf4	ENSMUST00000017530.3	TNF receptor associated factor 4
3881	Des	ENSMUST00000027409.9	desmin
3882	Ran	ENSMUST00000031383.8	RAN, member RAS oncogene family
3883	Exoc7	ENSMUST00000106411.4	exocyst complex component 7
3884	Fan1	ENSMUST00000177443.2	FANCD2/FANCI-associated nuclease 1
3885	Psmd1	ENSMUST00000027432.8	proteasome (prosome, macropain) 26S subunit, non-ATPase, 1
3886	Sord	ENSMUST00000110551.3	sorbitol dehydrogenase
3887	Numbl	ENSMUST00000079258.6	numb-like
3888	Med6	ENSMUST00000161598.2	mediator complex subunit 6
3889	Ptdss1	ENSMUST00000021990.3	phosphatidylserine synthase 1
3890	Lilra5	ENSMUST00000117550.1	leukocyte immunoglobulin-like receptor, subfamily A (with TM domain), member 5
3891	Emc10	ENSMUST00000118515.2	ER membrane protein complex subunit 10
3892	Pcdh10	ENSMUST00000171554.2	protocadherin 10
3893	Amz2	ENSMUST00000092500.5	archaelysin family metallopeptidase 2
3894	0610009L18Rik	ENSMUST00000143813.1	RIKEN cDNA 0610009L18 gene
3895	Pnisr	ENSMUST00000098238.3	PNN interacting serine/arginine-rich
3896	Afap1l1	ENSMUST00000120472.1	actin filament associated protein 1-like 1
3897	Mgst3	ENSMUST00000028005.2	microsomal glutathione S-transferase 3

3898	Lancl1	ENSMUST00000027149.6	LanC (bacterial lantibiotic synthetase component C)-like 1
3899	Smg7	ENSMUST00000073441.7	Smg-7 homolog, nonsense mediated mRNA decay factor (C. elegans)
3900	Epn3	ENSMUST00000127305.1	epsin 3
3901	Dtnb	ENSMUST00000164578.3	dystrobrevin, beta
3902	Tmem60	ENSMUST00000115259.2	transmembrane protein 60
3903	Med23	ENSMUST00000092646.7	mediator complex subunit 23
3904	Tmem176b	ENSMUST00000166247.2	transmembrane protein 176B
3905	Col3a1	ENSMUST00000087883.7	collagen, type III, alpha 1
3906	Abra	ENSMUST00000054742.5	actin-binding Rho activating protein
3907	Ccdc80	ENSMUST00000061050.5	coiled-coil domain containing 80
3908	Ttc5	ENSMUST00000006451.6	tetratricopeptide repeat domain 5
3909	Hnrnpu	ENSMUST00000037748.8	heterogeneous nuclear ribonucleoprotein U
3910	S1pr3	ENSMUST00000087978.3	sphingosine-1-phosphate receptor 3
3911	Frmd8	ENSMUST00000025728.7	FERM domain containing 8
3912	Radil	ENSMUST00000063635.9	Ras association and DIL domains
3913	Dpf2	ENSMUST00000136983.2	D4, zinc and double PHD fingers family 2
3914	Cdh12	ENSMUST00000075132.6	cadherin 12
3915	Rnf215	ENSMUST00000003677.5	ring finger protein 215
3916	Rtn4ip1	ENSMUST00000054418.6	reticulon 4 interacting protein 1
3917	Leo1	ENSMUST00000048937.4	Leo1, Paf1/RNA polymerase II complex component, homolog (S. cerevisiae)
3918	Fbxo46	ENSMUST00000053109.3	F-box protein 46
3919	Aars	ENSMUST00000034441.7	alanyl-tRNA synthetase
3920	C1ql2	ENSMUST00000037286.9	complement component 1, q subcomponent-like 2
3921	Nphs2	ENSMUST00000027896.4	nephrosis 2, podocin
3922	Ndufb2	ENSMUST00000135671.2	NADH dehydrogenase (ubiquinone) 1 beta subcomplex, 2
3923	Las1l	ENSMUST00000079987.7	LAS1-like (S. cerevisiae)
3924	Lpar6	ENSMUST00000044405.7	lysophosphatidic acid receptor 6
3925	Polr3k	ENSMUST00000039551.8	polymerase (RNA) III (DNA directed) polypeptide K
3926	Fam187b	ENSMUST00000058093.4	family with sequence similarity 187, member B
3927	Gimap4	ENSMUST00000090070.5	GTPase, IMAP family member 4
3928	Tcf20	ENSMUST00000109510.3	transcription factor 20
3929	Ccdc163	ENSMUST00000030452.7	coiled-coil domain containing 163
3930	Ephb4	ENSMUST00000111054.1	Eph receptor B4
3931	Man2b1	ENSMUST00000034121.9	mannosidase 2, alpha B1
3932	G0s2	ENSMUST00000009777.2	G0/G1 switch gene 2
3933	Hspa2	ENSMUST00000080449.5	heat shock protein 2
3934	Gzf1	ENSMUST00000028928.7	GDNF-inducible zinc finger protein 1
3935	Dhx36	ENSMUST00000029336.4	DEAH (Asp-Glu-Ala-His) box polypeptide 36
3936	Cpt1a	ENSMUST00000025835.4	carnitine palmitoyltransferase 1a, liver
3937	Erich5	ENSMUST00000060894.7	glutamate rich 5
3938	L3mbtl4	ENSMUST00000093007.4	l(3)mbt-like 4 (Drosophila)
3939	D830039M14Rik	ENSMUST00000051330.4	RIKEN cDNA D830039M14 gene
3940	Incenp	ENSMUST00000025562.7	inner centromere protein
3941	Tmbim4	ENSMUST00000020446.5	transmembrane BAX inhibitor motif containing 4
3942	Rfk	ENSMUST00000025617.3	riboflavin kinase
3943	Asic5	ENSMUST00000029641.4	acid-sensing (proton-gated) ion channel family member 5
3944	Ctps2	ENSMUST00000033727.8	cytidine 5'-triphosphate synthase 2

3945	Pik3ap1	ENSMUST00000059672.7	phosphoinositide-3-kinase adaptor protein 1
3946	Cyp4b1	ENSMUST00000102707.4	cytochrome P450, family 4, subfamily b, polypeptide 1
3947	Pih1d1	ENSMUST00000107813.2	PIH1 domain containing 1
3948	Lgals12	ENSMUST00000159983.2	lectin, galactose binding, soluble 12
3949	Akt3	ENSMUST00000111160.3	thymoma viral proto-oncogene 3
3950	Utp3	ENSMUST00000090413.4	UTP3, small subunit (SSU) processome component, homolog (S. cerevisiae)
3951	Emc2	ENSMUST00000022962.6	ER membrane protein complex subunit 2
3952	Shfm1	ENSMUST00000041111.9	split hand/foot malformation (ectrodactyly) type 1
3953	4921524J17Rik	ENSMUST00000047749.5	RIKEN cDNA 4921524J17 gene
3954	Polr3a	ENSMUST00000026322.7	polymerase (RNA) III (DNA directed) polypeptide A
3955	Cnot10	ENSMUST00000070117.6	CCR4-NOT transcription complex, subunit 10
3956	Prpsap1	ENSMUST00000106391.2	phosphoribosyl pyrophosphate synthetase-associated protein 1
3957	Gm14440	ENSMUST00000109051.2	predicted gene 14440
3958	Srgap2	ENSMUST00000097588.3	SLIT-ROBO Rho GTPase activating protein 2
3959	Dcn	ENSMUST00000105287.4	decorin
3960	Gatad1	ENSMUST00000007559.9	GATA zinc finger domain containing 1
3961	Emc6	ENSMUST00000054952.3	ER membrane protein complex subunit 6
3962	Arhgef40	ENSMUST00000093813.6	Rho guanine nucleotide exchange factor (GEF) 40
3963	Pfdn1	ENSMUST00000025204.5	prefoldin 1
3964	Pex14	ENSMUST00000103217.5	peroxisomal biogenesis factor 14
3965	Rasgef1b	ENSMUST00000031276.9	RasGEF domain family, member 1B
3966	Snapc5	ENSMUST00000034965.6	small nuclear RNA activating complex, polypeptide 5
3967	Rab24	ENSMUST00000035242.7	RAB24, member RAS oncogene family
3968	Txn1	ENSMUST00000030051.5	thioredoxin 1
3969	Ugt2b37	ENSMUST00000075858.3	UDP glucuronosyltransferase 2 family, polypeptide B37
3970	Myl4	ENSMUST00000018800.3	myosin, light polypeptide 4
3971	Klhl5	ENSMUST00000101192.3	kelch-like 5
3972	Ippk	ENSMUST00000021817.8	inositol 1,3,4,5,6-pentakisphosphate 2-kinase
3973	Gm14391	ENSMUST00000099028.4	predicted gene 14391
3974	Elmo2	ENSMUST00000103091.3	engulfment and cell motility 2
3975	Dock9	ENSMUST00000100299.4	dedicator of cytokinesis 9
3976	Slc16a3	ENSMUST00000168579.2	solute carrier family 16 (monocarboxylic acid transporters), member 3
3977	Pet112	ENSMUST00000127348.2	PET112 homolog (S. cerevisiae)
3978	Dock11	ENSMUST00000115264.1	dedicator of cytokinesis 11
3979	Them4	ENSMUST00000049822.7	thioesterase superfamily member 4
3980	Ppl	ENSMUST00000035672.3	periplakin
3981	Gpr12	ENSMUST00000036211.6	G-protein coupled receptor 12
3982	Phlpp1	ENSMUST00000061047.6	PH domain and leucine rich repeat protein phosphatase 1
3983	Slc9a1	ENSMUST00000030669.7	solute carrier family 9 (sodium/hydrogen exchanger), member 1
3984	Vps26a	ENSMUST00000105447.4	vacuolar protein sorting 26 homolog A (yeast)
3985	Atp8a1	ENSMUST00000037380.9	ATPase, aminophospholipid transporter (APLT), class I, type 8A, member 1
3986	Zmiz2	ENSMUST00000109787.2	zinc finger, MIZ-type containing 2
3987	Uroc1	ENSMUST00000046128.9	urocanase domain containing 1

3988	Uqcr11	ENSMUST00000020372.5	ubiquinol-cytochrome c reductase, complex III subunit XI
3989	Aldob	ENSMUST00000029987.9	aldolase B, fructose-bisphosphate
3990	Polr3d	ENSMUST00000000793.6	polymerase (RNA) III (DNA directed) polypeptide D
3991	Exoc5	ENSMUST00000162175.2	exocyst complex component 5
3992	Arpc3	ENSMUST00000102525.5	actin related protein 2/3 complex, subunit 3
3993	Apol6	ENSMUST00000149569.2	apolipoprotein L 6
3994	Paqr8	ENSMUST00000189400.1	progestin and adipoQ receptor family member VIII
3995	Eml1	ENSMUST00000054955.8	echinoderm microtubule associated protein like 1
3996	Actn1	ENSMUST00000021554.9	actinin, alpha 1
3997	Prmt7	ENSMUST00000071592.6	protein arginine N-methyltransferase 7
3998	Ppp1r35	ENSMUST00000031739.4	protein phosphatase 1, regulatory subunit 35
3999	Fgf23	ENSMUST00000000186.6	fibroblast growth factor 23
4000	Paics	ENSMUST00000120912.2	phosphoribosylaminoimidazole carboxylase, phosphoribosylaminoribosylaminoimidazole, succinocarboxamide synthetase
4001	Cyb5r4	ENSMUST00000168529.3	cytochrome b5 reductase 4
4002	Dgki	ENSMUST00000101532.4	diacylglycerol kinase, iota
4003	Ces1g	ENSMUST00000044602.7	carboxylesterase 1G
4004	Maf	ENSMUST00000109104.1	avian musculoaponeurotic fibrosarcoma (v-maf) AS42 oncogene homolog
4005	Def8	ENSMUST00000065534.4	differentially expressed in FDCP 8
4006	Fkbp9	ENSMUST00000031795.7	FK506 binding protein 9
4007	Cyp2j5	ENSMUST00000030299.7	cytochrome P450, family 2, subfamily j, polypeptide 5
4008	Psmb1	ENSMUST00000014913.9	proteasome (prosome, macropain) subunit, beta type 1
4009	Gm14306	ENSMUST00000108985.3	predicted gene 14306
4010	Ms4a1	ENSMUST00000169159.2	membrane-spanning 4-domains, subfamily A, member 1
4011	Urb2	ENSMUST00000034457.8	URB2 ribosome biogenesis 2 homolog (S. cerevisiae)
4012	Stab2	ENSMUST00000035288.9	stabilin 2
4013	Mtr	ENSMUST00000099856.4	5-methyltetrahydrofolate-homocysteine methyltransferase
4014	Rsu1	ENSMUST00000028059.3	Ras suppressor protein 1
4015	Chst12	ENSMUST00000043050.8	carbohydrate sulfotransferase 12
4016	Vbp1	ENSMUST00000033540.5	von Hippel-Lindau binding protein 1
4017	Mtx2	ENSMUST00000028511.7	metaxin 2
4018	Smc1a	ENSMUST00000045312.5	structural maintenance of chromosomes 1A
4019	Rsl1d1	ENSMUST00000119953.1	ribosomal L1 domain containing 1
4020	Arl6ip5	ENSMUST00000044681.6	ADP-ribosylation factor-like 6 interacting protein 5
4021	Syt15	ENSMUST00000035351.5	synaptotagmin XV
4022	Gm2026	ENSMUST00000174416.2	predicted gene 2026
4023	Cnnm3	ENSMUST00000097776.3	cyclin M3
4024	Cxcl16	ENSMUST00000019064.3	chemokine (C-X-C motif) ligand 16
4025	Mrps16	ENSMUST00000061444.3	mitochondrial ribosomal protein S16
4026	Ccdc85b	ENSMUST00000179549.1	coiled-coil domain containing 85B
4027	Gm4788	ENSMUST00000027612.9	predicted gene 4788
4028	Neurl2	ENSMUST00000042775.4	neuralized-like 2 (Drosophila)
4029	Slitrk3	ENSMUST00000059407.7	SLIT and NTRK-like family, member 3
4030	Tmem165	ENSMUST00000031144.8	transmembrane protein 165
4031	Urgcp	ENSMUST00000093362.6	upregulator of cell proliferation

4032	Myo9b	ENSMUST00000168839.2	myosin IXb
4033	Top2b	ENSMUST00000017629.5	topoisomerase (DNA) II beta
4034	Kdm6a	ENSMUST00000044484.7	lysine (K)-specific demethylase 6A
4035	Snx5	ENSMUST00000110030.4	sorting nexin 5
4036	Cyp2b13	ENSMUST00000005669.7	cytochrome P450, family 2, subfamily b, polypeptide 13
4037	Rapgef1	ENSMUST00000102872.5	Rap guanine nucleotide exchange factor (GEF) 1
4038	Exog	ENSMUST00000035094.7	endo/exonuclease (5'-3'), endonuclease G-like
4039	Anapc10	ENSMUST00000048147.7	anaphase promoting complex subunit 10
4040	Nelfa	ENSMUST00000030993.6	negative elongation factor complex member A, Whsc2
4041	Top3b	ENSMUST00000023465.8	topoisomerase (DNA) III beta
4042	Cox6a1	ENSMUST00000040154.8	cytochrome c oxidase subunit VIa polypeptide 1
4043	Nop16	ENSMUST00000026987.6	NOP16 nucleolar protein
4044	Krtcap2	ENSMUST00000040888.6	keratinocyte associated protein 2
4045	Ubxn6	ENSMUST00000019722.6	UBX domain protein 6
4046	Nxph1	ENSMUST00000160300.1	neurexophilin 1
4047	Htr2a	ENSMUST00000036653.3	5-hydroxytryptamine (serotonin) receptor 2A
4048	Nr1h4	ENSMUST00000058126.9	nuclear receptor subfamily 1, group H, member 4
4049	Dynlt1a	ENSMUST00000169415.1	dynein light chain Tctex-type 1A
4050	Megf8	ENSMUST00000128119.1	multiple EGF-like-domains 8
4051	Sp110	ENSMUST00000093508.6	Sp110 nuclear body protein
4052	Slmo2	ENSMUST00000016401.9	slowmo homolog 2 (Drosophila)
4053	Tcp11l2	ENSMUST00000020223.7	t-complex 11 (mouse) like 2
4054	Epha6	ENSMUST00000068860.7	Eph receptor A6
4055	Gm4631	ENSMUST00000121393.3	predicted gene 4631
4056	Cenpb	ENSMUST00000089510.4	centromere protein B
4057	Gm14399	ENSMUST00000109062.2	predicted gene 14399
4058	Rpf2	ENSMUST00000045114.7	ribosome production factor 2 homolog (S. cerevisiae)
4059	Itih4	ENSMUST00000006703.7	inter alpha-trypsin inhibitor, heavy chain 4
4060	Mfsd2b	ENSMUST00000137337.2	major facilitator superfamily domain containing 2B
4061	Vcl	ENSMUST00000022369.7	vinculin
4062	Jsrp1	ENSMUST00000020435.5	junctional sarcoplasmic reticulum protein 1
4063	Dock7	ENSMUST00000075836.6	dedicator of cytokinesis 7
4064	Cyp2c40	ENSMUST00000160476.2	cytochrome P450, family 2, subfamily c, polypeptide 40
4065	Gm14326	ENSMUST00000108932.2	predicted gene 14326
4066	H2-Aa	ENSMUST00000040655.7	histocompatibility 2, class II antigen A, alpha
4067	Top1mt	ENSMUST00000000958.8	DNA topoisomerase 1, mitochondrial
4068	Hspg2	ENSMUST00000030547.9	perlecan (heparan sulfate proteoglycan 2)
4069	Cpn1	ENSMUST00000026210.4	carboxypeptidase N, polypeptide 1
4070	Slc7a14	ENSMUST00000091259.3	solute carrier family 7 (cationic amino acid transporter, y+ system), member 14
4071	Tbc1d10a	ENSMUST00000041042.7	TBC1 domain family, member 10a
4072	Dnajc25	ENSMUST00000095070.3	DnaJ (Hsp40) homolog, subfamily C, member 25
4073	Atp2c1	ENSMUST00000112558.3	ATPase, Ca++-sequestering
4074	Inpp5k	ENSMUST00000006286.3	inositol polyphosphate 5-phosphatase K
4075	Srp19	ENSMUST00000072576.4	signal recognition particle 19
4076	Cfhr2	ENSMUST00000094489.3	complement factor H-related 2
4077	Aldh1a7	ENSMUST00000025656.3	aldehyde dehydrogenase family 1, subfamily A7
4078	N4bp3	ENSMUST00000001080.10	NEDD4 binding protein 3
4079	9130401M01Rik	ENSMUST00000100655.4	RIKEN cDNA 9130401M01 gene

4080	Scyl2	ENSMUST00000174252.2	SCY1-like 2 (S. cerevisiae)
4081	Acadl	ENSMUST00000027153.5	acyl-Coenzyme A dehydrogenase, long-chain
4082	Avl9	ENSMUST00000031805.8	AVL9 homolog (S. cerevisiase)
4083	Ptpra	ENSMUST00000028769.7	protein tyrosine phosphatase, receptor type, A
4084	Cdkl1	ENSMUST00000021377.4	cyclin-dependent kinase-like 1 (CDC2-related kinase)
4085	Cd300lg	ENSMUST00000017453.6	CD300 antigen like family member G
4086	Gtpbp2	ENSMUST00000024748.8	GTP binding protein 2
4087	Prss16	ENSMUST00000006341.2	protease, serine 16 (thymus)
4088	Slc30a5	ENSMUST00000067246.4	solute carrier family 30 (zinc transporter), member 5
4089	Qsox1	ENSMUST00000035325.9	quiescin Q6 sulfhydryl oxidase 1
4090	Taf1b	ENSMUST00000075954.7	TATA box binding protein (Tbp)-associated factor, RNA polymerase I, B
4091	Rchy1	ENSMUST00000031345.9	ring finger and CHY zinc finger domain containing 1
4092	Sult1c2	ENSMUST00000023886.6	sulfotransferase family, cytosolic, 1C, member 2
4093	Gm20878	ENSMUST00000153997.2	predicted gene, 20878
4094	Rpl26	ENSMUST00000073471.7	ribosomal protein L26
4095	Wbp4	ENSMUST00000022601.5	WW domain binding protein 4
4096	Slitrk5	ENSMUST00000042767.7	SLIT and NTRK-like family, member 5
4097	BC022687	ENSMUST00000037014.4	cDNA sequence BC022687
4098	Qprt	ENSMUST00000032912.5	quinolinate phosphoribosyltransferase
4099	Il6st	ENSMUST00000183663.2	interleukin 6 signal transducer
4100	Hint1	ENSMUST00000020504.5	histidine triad nucleotide binding protein 1
4101	Ubl5	ENSMUST00000161486.2	ubiquitin-like 5
4102	Myoz1	ENSMUST00000090469.6	myozenin 1
4103	Farp1	ENSMUST00000026635.7	FERM, RhoGEF (Arhgef) and pleckstrin domain protein 1 (chondrocyte-derived)
4104	Ephx2	ENSMUST00000070515.1	epoxide hydrolase 2, cytoplasmic
4105	Rab11fip3	ENSMUST00000118828.2	RAB11 family interacting protein 3 (class II)
4106	Banp	ENSMUST00000170857.2	BTG3 associated nuclear protein
4107	Tceb3	ENSMUST00000030427.5	transcription elongation factor B (SIII), polypeptide 3
4108	A1bg	ENSMUST00000096418.3	alpha-1-B glycoprotein
4109	Spcs2	ENSMUST00000036274.6	signal peptidase complex subunit 2 homolog (S. cerevisiae)
4110	Kti12	ENSMUST00000102738.3	KTI12 homolog, chromatin associated (S. cerevisiae)
4111	Timm23	ENSMUST00000170331.2	translocase of inner mitochondrial membrane 23
4112	Dis3	ENSMUST00000042471.9	DIS3 mitotic control homolog (S. cerevisiae)
4113	Ugp2	ENSMUST00000102875.5	UDP-glucose pyrophosphorylase 2
4114	Dennd4b	ENSMUST00000129564.2	DENN/MADD domain containing 4B
4115	Slfn8	ENSMUST00000038141.9	schlafen 8
4116	Mesdc2	ENSMUST00000094215.4	mesoderm development candidate 2
4117	Gm4723	ENSMUST00000109048.2	predicted gene 4723
4118	Nt5c3	ENSMUST00000101367.3	5'-nucleotidase, cytosolic III
4119	Psmd7	ENSMUST00000044106.4	proteasome (prosome, macropain) 26S subunit, non-ATPase, 7
4120	Brd7	ENSMUST00000034085.7	bromodomain containing 7
4121	2010107E04Rik	ENSMUST00000021719.5	RIKEN cDNA 2010107E04 gene
4122	Gbe1	ENSMUST00000163832.2	glucan (1,4-alpha-), branching enzyme 1
4123	Kxd1	ENSMUST00000093456.6	KxDL motif containing 1
4124	Lsm4	ENSMUST00000034311.9	LSM4 homolog, U6 small nuclear RNA associated (S. cerevisiae)
4125	Abcc3	ENSMUST00000178136.2	ATP-binding cassette, sub-family C (CFTR/MRP), member 3

4126	Sike1	ENSMUST00000029447.6	suppressor of IKBKE 1
4127	Rbm7	ENSMUST00000170000.2	RNA binding motif protein 7
4128	Rbm47	ENSMUST00000113726.2	RNA binding motif protein 47
4129	Pole	ENSMUST00000007296.6	polymerase (DNA directed), epsilon
4130	Cyfip1	ENSMUST00000032629.10	cytoplasmic FMR1 interacting protein 1
4131	Uqcrfs1	ENSMUST00000042834.3	ubiquinol-cytochrome c reductase, Rieske iron-sulfur polypeptide 1
4132	Acox1	ENSMUST00000066587.6	acyl-Coenzyme A oxidase 1, palmitoyl
4133	Zfp651	ENSMUST00000093772.3	zinc finger protein 651
4134	Ywhaz	ENSMUST00000022894.8	tyrosine 3-monooxygenase/tryptophan 5-monooxygenase activation protein, zeta polypeptide
4135	Havcr2	ENSMUST00000020668.9	hepatitis A virus cellular receptor 2
4136	BC027231	ENSMUST00000048788.8	cDNA sequence BC027231
4137	Scrib	ENSMUST00000002603.6	scribbled homolog (Drosophila)
4138	Aldh2	ENSMUST00000031411.9	aldehyde dehydrogenase 2, mitochondrial
4139	Phtf1	ENSMUST00000055425.9	putative homeodomain transcription factor 1
4140	Nlgn1	ENSMUST00000108308.4	neuroligin 1
4141	Dnajc10	ENSMUST00000028392.7	DnaJ (Hsp40) homolog, subfamily C, member 10
4142	Anapc4	ENSMUST00000031072.8	anaphase promoting complex subunit 4
4143	Ctnnal1	ENSMUST00000045142.9	catenin (cadherin associated protein), alpha-like 1
4144	Dcc	ENSMUST00000114943.4	deleted in colorectal carcinoma
4145	Snrnp70	ENSMUST00000074575.7	small nuclear ribonucleoprotein 70 (U1)
4146	Kmt2e	ENSMUST00000094962.3	lysine (K)-specific methyltransferase 2E
4147	Dph6	ENSMUST00000028640.8	diphthamine biosynthesis 6
4148	Stx8	ENSMUST00000021285.8	syntaxin 8
4149	Aste1	ENSMUST00000035181.4	asteroid homolog 1 (Drosophila)
4150	Sftpb	ENSMUST00000183018.2	surfactant associated protein B
4151	Hdac7	ENSMUST00000079838.8	histone deacetylase 7
4152	Arhgap42	ENSMUST00000093893.6	Rho GTPase activating protein 42
4153	Pkp2	ENSMUST00000039408.2	plakophilin 2
4154	Rpl8	ENSMUST00000004072.8	ribosomal protein L8
4155	Imp3	ENSMUST00000034827.9	IMP3, U3 small nucleolar ribonucleoprotein, homolog (yeast)
4156	2900011O08Rik	ENSMUST00000056521.6	RIKEN cDNA 2900011O08 gene
4157	Otud7a	ENSMUST00000058476.8	OTU domain containing 7A
4158	Colq	ENSMUST00000112027.3	collagen-like tail subunit (single strand of homotrimer) of asymmetric acetylcholinesterase
4159	Alb	ENSMUST00000031314.8	albumin
4160	Slc17a1	ENSMUST00000006785.7	solute carrier family 17 (sodium phosphate), member 1
4161	Mei4	ENSMUST00000057067.4	meiosis-specific, MEI4 homolog (S. cerevisiae)
4162	Zfp467	ENSMUST00000114566.2	zinc finger protein 467
4163	Ugt8a	ENSMUST00000057944.7	UDP galactosyltransferase 8A
4164	Slc16a4	ENSMUST00000029502.8	solute carrier family 16 (monocarboxylic acid transporters), member 4
4165	Sdha	ENSMUST00000022062.7	succinate dehydrogenase complex, subunit A, flavoprotein (Fp)
4166	Calm3	ENSMUST00000019514.9	calmodulin 3
4167	Aplp2	ENSMUST00000072634.8	amyloid beta (A4) precursor-like protein 2
4168	Aldh1l1	ENSMUST00000130418.2	aldehyde dehydrogenase 1 family, member L1
4169	F830016B08Rik	ENSMUST00000171297.1	RIKEN cDNA F830016B08 gene
4170	Lyrm2	ENSMUST00000062802.4	LYR motif containing 2

4171	Ffar1	ENSMUST00000052700.4	free fatty acid receptor 1
4172	Hoxd11	ENSMUST00000142312.2	homeobox D11
4173	Gdf2	ENSMUST00000100720.1	growth differentiation factor 2
4174	Wdr36	ENSMUST00000053663.9	WD repeat domain 36
4175	Mt1	ENSMUST00000034215.6	metallothionein 1
4176	Ppp2r3a	ENSMUST00000075941.6	protein phosphatase 2, regulatory subunit B", alpha
4177	Cp	ENSMUST00000091309.6	ceruloplasmin
4178	Zfp385c	ENSMUST00000103119.4	zinc finger protein 385C
4179	Gm8898	ENSMUST00000109018.2	predicted gene 8898
4180	B2m	ENSMUST00000102476.4	beta-2 microglobulin
4181	Fkbp8	ENSMUST00000119353.3	FK506 binding protein 8
4182	Nme1	ENSMUST00000135884.2	NME/NM23 nucleoside diphosphate kinase 1
4183	Bbc3	ENSMUST00000002152.7	BCL2 binding component 3
4184	Pln	ENSMUST00000163319.2	phospholamban
4185	Slc25a46	ENSMUST00000060396.6	solute carrier family 25, member 46
4186	Aamdc	ENSMUST00000178078.2	adipogenesis associated Mth938 domain containing
4187	Wdr12	ENSMUST00000027173.9	WD repeat domain 12
4188	Nid1	ENSMUST00000005532.7	nidogen 1
4189	Acot13	ENSMUST00000006900.6	acyl-CoA thioesterase 13
4190	Plcxd1	ENSMUST00000086687.4	phosphatidylinositol-specific phospholipase C, X domain containing 1
4191	2610001J05Rik	ENSMUST00000139231.1	RIKEN cDNA 2610001J05 gene
4192	Mrc2	ENSMUST00000100335.4	mannose receptor, C type 2
4193	Pawr	ENSMUST00000095313.3	PRKC, apoptosis, WT1, regulator
4194	1110037F02Rik	ENSMUST00000059914.7	RIKEN cDNA 1110037F02 gene
4195	Hace1	ENSMUST00000037044.7	HECT domain and ankyrin repeat containing, E3 ubiquitin protein ligase 1
4196	Cox7a2	ENSMUST00000034881.6	cytochrome c oxidase subunit VIIa 2
4197	Tusc3	ENSMUST00000167992.2	tumor suppressor candidate 3
4198	Dlgap2	ENSMUST00000133298.2	discs, large (Drosophila) homolog-associated protein 2
4199	4932438A13Rik	ENSMUST00000057272.9	RIKEN cDNA 4932438A13 gene
4200	Acsf3	ENSMUST00000015160.5	acyl-CoA synthetase family member 3
4201	Proz	ENSMUST00000033822.2	protein Z, vitamin K-dependent plasma glycoprotein
4202	Slain2	ENSMUST00000144843.2	SLAIN motif family, member 2
4203	Rtn4rl1	ENSMUST00000102514.3	reticulon 4 receptor-like 1
4204	Sec62	ENSMUST00000029256.7	SEC62 homolog (S. cerevisiae)
4205	0610007P14Rik	ENSMUST00000021676.6	RIKEN cDNA 0610007P14 gene
4206	Sdr9c7	ENSMUST00000047134.7	4short chain dehydrogenase/reductase family 9C, member 7
4207	Gja8	ENSMUST00000062944.5	gap junction protein, alpha 8
4208	Nudt2	ENSMUST00000030154.6	nudix (nucleoside diphosphate linked moiety X)-type motif 2
4209	Hs3st1	ENSMUST00000117944.1	heparan sulfate (glucosamine) 3-O-sulfotransferase 1
4210	Chmp2a	ENSMUST00000005711.4	charged multivesicular body protein 2A
4211	Cd99l2	ENSMUST00000037391.6	CD99 antigen-like 2
4212	Rpsa	ENSMUST00000035105.5	ribosomal protein SA
4213	Slamf7	ENSMUST00000111276.3	SLAM family member 7
4214	Grik2	ENSMUST00000105487.2	glutamate receptor, ionotropic, kainate 2 (beta 2)
4215	Oaf	ENSMUST00000034512.5	OAF homolog (Drosophila)
4216	Mrpl24	ENSMUST00000019854.7	mitochondrial ribosomal protein L24

4217	Ppp2cb	ENSMUST00000009774.9	protein phosphatase 2 (formerly 2A), catalytic subunit, beta isoform
4218	Tiprl	ENSMUST00000043235.5	TIP41, TOR signalling pathway regulator-like (S. cerevisiae)
4219	Sars	ENSMUST00000090553.6	seryl-aminoacyl-tRNA synthetase
4220	Gyg	ENSMUST00000118015.3	glycogenin
4221	Fech	ENSMUST00000025484.7	ferrochelatase
4222	Moap1	ENSMUST00000173760.3	modulator of apoptosis 1
4223	Bnip3	ENSMUST00000106112.1	BCL2/adenovirus E1B interacting protein 3
4224	Gpm6a	ENSMUST00000033915.7	glycoprotein m6a
4225	Vps35	ENSMUST00000034131.8	vacuolar protein sorting 35
4226	Ugdh	ENSMUST00000117542.2	UDP-glucose dehydrogenase
4227	Rpp21	ENSMUST00000025319.6	ribonuclease P 21 subunit
4228	AA986860	ENSMUST00000039323.7	expressed sequence AA986860
4229	Gm20604	ENSMUST00000174651.1	predicted gene 20604
4230	Sqrdl	ENSMUST00000005953.5	sulfide quinone reductase-like (yeast)
4231	Calml4	ENSMUST00000034777.7	calmodulin-like 4
4232	Gnptab	ENSMUST00000020251.8	N-acetylglucosamine-1-phosphate transferase, alpha and beta subunits
4233	Wwp2	ENSMUST00000166615.1	WW domain containing E3 ubiquitin protein ligase 2
4234	Zfp804a	ENSMUST00000047527.7	zinc finger protein 804A
4235	Alyref	ENSMUST00000026125.2	Aly/REF export factor
4236	Lphn2	ENSMUST00000106128.2	latrophilin 2
4237	Txnl4a	ENSMUST00000145963.2	thioredoxin-like 4A
4238	Itfg1	ENSMUST00000034140.7	integrin alpha FG-GAP repeat containing 1
4239	Hivep1	ENSMUST00000060148.5	human immunodeficiency virus type I enhancer binding protein 1
4240	Tfr2	ENSMUST00000031729.8	transferrin receptor 2
4241	Hmgcr	ENSMUST00000022176.9	3-hydroxy-3-methylglutaryl-Coenzyme A reductase
4242	Adat2	ENSMUST00000019944.8	adenosine deaminase, tRNA-specific 2
4243	Mthfsl	ENSMUST00000113110.4	5, 10-methenyltetrahydrofolate synthetase-like
4244	Chd1l	ENSMUST00000029730.4	chromodomain helicase DNA binding protein 1-like
4245	Rab4b	ENSMUST00000093040.7	RAB4B, member RAS oncogene family
4246	Elmod1	ENSMUST00000048409.8	ELMO/CED-12 domain containing 1
4247	Ndrg1	ENSMUST00000005256.7	N-myc downstream regulated gene 1
4248	Plaa	ENSMUST00000107107.3	phospholipase A2, activating protein
4249	B630005N14Rik	ENSMUST00000045235.5	RIKEN cDNA B630005N14 gene
4250	Tdo2	ENSMUST00000029645.8	tryptophan 2,3-dioxygenase
4251	Cep170	ENSMUST00000057037.7	centrosomal protein 170
4252	Slc1a1	ENSMUST00000025875.4	solute carrier family 1 (neuronal/epithelial high affinity glutamate transporter, system Xag), member 1
4253	Sh3bp5l	ENSMUST00000116376.3	SH3 binding domain protein 5 like
4254	Xirp2	ENSMUST00000112347.2	xin actin-binding repeat containing 2
4255	Adipoq	ENSMUST00000023593.5	adiponectin, C1Q and collagen domain containing
4256	Slc25a10	ENSMUST00000026899.3	solute carrier family 25 (mitochondrial carrier, dicarboxylate transporter), member 10
4257	Mrpl14	ENSMUST00000024734.7	mitochondrial ribosomal protein L14
4258	Agtpbp1	ENSMUST00000022040.7	ATP/GTP binding protein 1
4259	Uaca	ENSMUST00000050183.6	uveal autoantigen with coiled-coil domains and ankyrin repeats
4260	Ptpn9	ENSMUST00000034832.6	protein tyrosine phosphatase, non-receptor type 9

4261	9930111J21Rik1	ENSMUST00000093153.1	RIKEN cDNA 9930111J21 gene 1
4262	Lrrc4c	ENSMUST00000135431.2	leucine rich repeat containing 4C
4263	Cd3d	ENSMUST00000034602.7	CD3 antigen, delta polypeptide
4264	Ndufaf7	ENSMUST00000024887.4	NADH dehydrogenase (ubiquinone) 1 alpha subcomplex assembly factor 7
4265	Plk2	ENSMUST00000022212.7	polo-like kinase 2
4266	Tmem37	ENSMUST00000056089.7	transmembrane protein 37
4267	Usp28	ENSMUST00000047349.6	ubiquitin specific peptidase 28
4268	Ikbkb	ENSMUST00000063401.4	inhibitor of kappaB kinase beta
4269	Ap5b1	ENSMUST00000096318.3	adaptor-related protein complex 5, beta 1 subunit
4270	Srpk1	ENSMUST00000130643.2	serine/arginine-rich protein specific kinase 1
4271	Grk6	ENSMUST00000001115.9	G protein-coupled receptor kinase 6
4272	1700024G13Rik	ENSMUST00000100723.2	RIKEN cDNA 1700024G13 gene
4273	Sfpq	ENSMUST00000030623.7	splicing factor proline/glutamine rich (polypyrimidine tract binding protein associated)
4274	Rbbp4	ENSMUST00000102598.3	retinoblastoma binding protein 4
4275	Tmem45a	ENSMUST00000023435.5	transmembrane protein 45a
4276	Cd8b1	ENSMUST00000065248.7	CD8 antigen, beta chain 1
4277	Adig	ENSMUST00000059889.3	adipogenin
4278	Zfp444	ENSMUST00000054680.6	zinc finger protein 444
4279	Wars2	ENSMUST00000004343.2	tryptophanyl tRNA synthetase 2 (mitochondrial)
4280	Erich1	ENSMUST00000110813.3	glutamate rich 1
4281	Lgi1	ENSMUST00000087252.6	leucine-rich repeat LGI family, member 1
4282	Nop10	ENSMUST00000028553.3	NOP10 ribonucleoprotein
4283	Amfr	ENSMUST00000053766.7	autocrine motility factor receptor
4284	Tmem184c	ENSMUST00000034030.9	transmembrane protein 184C
4285	Acy1	ENSMUST00000024031.7	aminoacylase 1
4286	Aven	ENSMUST00000099588.3	apoptosis, caspase activation inhibitor
4287	Lmo7	ENSMUST00000100337.4	LIM domain only 7
4288	Ndufb9	ENSMUST00000022980.3	NADH dehydrogenase (ubiquinone) 1 beta subcomplex, 9
4289	Elovl1	ENSMUST00000006557.7	elongation of very long chain fatty acids (FEN1/Elo2, SUR4/Elo3, yeast)-like 1
4290	Zbtb8os	ENSMUST00000119480.1	zinc finger and BTB domain containing 8 opposite strand
4291	Rab1	ENSMUST00000020358.6	RAB1, member RAS oncogene family
4292	Srsf5	ENSMUST00000094693.5	serine/arginine-rich splicing factor 5
4293	Pcnt	ENSMUST00000001179.5	pericentrin (kendrin)
4294	Luzp2	ENSMUST00000082373.6	leucine zipper protein 2
4295	Rab12	ENSMUST00000070538.5	RAB12, member RAS oncogene family
4296	Fbp1	ENSMUST00000092888.5	fructose bisphosphatase 1
4297	Sod2	ENSMUST00000007012.4	superoxide dismutase 2, mitochondrial
4298	AW549877	ENSMUST00000046633.8	expressed sequence AW549877
4299	Apip	ENSMUST00000011055.6	APAF1 interacting protein
4300	Ctbp1	ENSMUST00000079746.6	C-terminal binding protein 1
4301	Tmem9b	ENSMUST00000033333.7	TMEM9 domain family, member B
4302	Sdc1	ENSMUST00000020911.8	syndecan 1
4303	Zfp169	ENSMUST00000176176.2	zinc finger protein 169
4304	Dpf3	ENSMUST00000178756.2	D4, zinc and double PHD fingers, family 3
4305	Prkrir	ENSMUST00000033009.10	protein-kinase, interferon-inducible double stranded RNA dependent inhibitor, repressor of (P58 repressor)
4306	Fgf16	ENSMUST00000033581.3	fibroblast growth factor 16

4307	Mep1a	ENSMUST00000117137.2	meprin 1 alpha
4308	Slc26a9	ENSMUST00000049027.4	solute carrier family 26, member 9
4309	Asb18	ENSMUST00000086882.2	ankyrin repeat and SOCS box-containing 18
4310	Slc26a5	ENSMUST00000115176.2	solute carrier family 26, member 5
4311	H3f3b	ENSMUST00000106454.2	H3 histone, family 3B
4312	Nup214	ENSMUST00000065398.7	nucleoporin 214
4313	Neu3	ENSMUST00000036331.6	neuraminidase 3
4314	Ddx47	ENSMUST00000032326.5	DEAD (Asp-Glu-Ala-Asp) box polypeptide 47
4315	0610009O20Rik	ENSMUST00000025314.5	RIKEN cDNA 0610009O20 gene
4316	Gm6685	ENSMUST00000042595.6	predicted pseudogene 6685
4317	Kcnj4	ENSMUST00000057801.6	potassium inwardly-rectifying channel, subfamily J, member 4
4318	Ttc9c	ENSMUST00000096751.5	tetratricopeptide repeat domain 9C
4319	Slc7a13	ENSMUST00000035890.7	solute carrier family 7, (cationic amino acid transporter, y+ system) member 13
4320	Drosha	ENSMUST00000169061.2	drosha, ribonuclease type III
4321	P4hb	ENSMUST00000026122.5	prolyl 4-hydroxylase, beta polypeptide
4322	Slc13a1	ENSMUST00000031713.8	solute carrier family 13 (sodium/sulfate symporters), member 1
4323	Itih1	ENSMUST00000006704.9	inter-alpha trypsin inhibitor, heavy chain 1
4324	Ywhaq	ENSMUST00000103002.2	tyrosine 3-monooxygenase/tryptophan 5-monooxygenase activation protein, theta polypeptide
4325	Ccne1	ENSMUST00000108023.4	cyclin E1
4326	Cxcl9	ENSMUST00000113093.3	chemokine (C-X-C motif) ligand 9
4327	Mmp8	ENSMUST00000018765.2	matrix metallopeptidase 8
4328	Mamstr	ENSMUST00000148532.1	MEF2 activating motif and SAP domain containing transcriptional regulator
4329	Tctn3	ENSMUST00000025981.9	tectonic family member 3
4330	Gpnmb	ENSMUST00000031840.7	glycoprotein (transmembrane) nmb
4331	Ncaph2	ENSMUST00000036987.6	non-SMC condensin II complex, subunit H2
4332	Slc22a19	ENSMUST00000025666.7	solute carrier family 22 (organic anion transporter), member 19
4333	Sptlc1	ENSMUST00000021920.6	serine palmitoyltransferase, long chain base subunit 1
4334	Patz1	ENSMUST00000134089.2	POZ (BTB) and AT hook containing zinc finger 1
